# Functional characterization of CreA, ZnT, and MTase as key regulators of cadmium resistance in *Paecilomyces lilacinus*

**DOI:** 10.3389/fmicb.2026.1792636

**Published:** 2026-05-14

**Authors:** FangMei Wang, WenTao Zhong, Qiu Wang, Yue Xu, Hui Chen, ShuHao Wang, ChangZheng Fan, Zheng Li, BaiYu Li, XiaoFeng Li, XiaoFeng Ding, ShuangLin Xiang

**Affiliations:** 1 Hunan Provincial Institute of Product and Goods Quality Inspection, Changsha, China; 2 Key Laboratory of Protein Chemistry and Development Biology of State Education Ministry of China, College of Life Science, Hunan Normal University, Changsha, China; 3 Department of Laboratory Medicine, Peking University Shenzhen Hospital, Shenzhen, China; 4 College of Environment Science and Engineering, Hunan Normal University, Changsha, China

**Keywords:** cadmium (Cd) resistance mechanisms, CREA, heavy metal detoxification processes, MTase, *Paecilomyces lilacinus*, ZnT

## Abstract

**Introduction:**

Following the implementation of the Heavy Metal Pollution Control Program in the *Xiangjiang River Basin*, significant progress has been achieved in the effective treatment and remediation of heavy metal contamination in the region. This study aims to identify and investigate microbial species that have adapted to long-term heavy metal contamination in soil environments, with a particular focus on their cadmium (Cd) tolerance mechanisms under stress conditions.

**Methods:**

Using high-throughput Illumina HiSeq sequencing, differentially expressed genes were identified, annotated, and classified through comprehensive bioinformatics analyses. The associated signaling pathways were systematically examined in detail for functional interpretation.

**Results:**

This study highlights novel Cd-resistant genes exhibiting pronounced differential expression, thereby offering valuable insights into the Cd-tolerance mechanisms of filamentous fungi under heavy metal stress.

**Discussion:**

These findings provide a solid foundation for improving fungal tolerance through targeted genetic modifications, such as small interfering RNA (siRNA) interference or gene overexpression, with potential applications in environmental bioremediation strategies.

## 1 Introduction

Heavy metal pollution, particularly Cd contamination, poses a significant and persistent threat to both the environment and public health due to its persistence and high toxicity (Lu et al., 2022). Cd and its compounds, which are highly toxic and widely used in various industrial applications, significantly contribute to long-term soil contamination and ecological degradation (Wang and Chadwick, 2024). In the *Xiangjiang River Basin*, the substantial accumulation of heavy metals in sediment layers not only seriously jeopardizes ecosystem stability but also poses a significant risk to human health, primarily through bioaccumulation within the food chain. To effectively address these challenges, researchers have developed various advanced methods to analyze the structure, diversity, and functional activity of microbial communities in contaminated environments (Vinay et al., 2025; Iddrisu et al., 2024; Badeenezhad et al., 2023; Singh et al., 2024).

Traditional physicochemical methods for heavy metal remediation, such as soil washing and chemical stabilization techniques, are often costly, resource-intensive, and environmentally invasive in practical applications. In contrast, microbial remediation offers a more sustainable and environmentally friendly alternative through processes such as biosorption, bioaccumulation, and biotransformation mechanisms. Filamentous fungi possess functional groups (e.g., hydroxyl, carboxyl, and amino groups) that can effectively complex with heavy metal ions, thereby reducing cellular toxicity through mechanisms such as precipitation, complexation, and adsorption processes (Kumar et al., 2025; Xia et al., 2024; Abbas and Rafatullah, 2021). Microbial remediation, which capitalizes on interactions between microorganisms and heavy metals, has emerged as a cost-effective, environmentally friendly, and highly efficient strategy for mitigating heavy metal contamination (Yin et al., 2019; Das et al., 2022). For example, the oxalate-producing filamentous fungus Penicillium oxalicum ZP6 efficiently sequesters Cd in phosphate mine spoils at a rapid rate, thereby providing a low-cost and environmentally sustainable remediation solution. However, the molecular mechanisms governing fungal Cd tolerance remain incompletely understood, underscoring the urgent need for further systematic investigation (Zheng et al., 2022; Maroušek et al., 2022). Furthermore, the molecular mechanisms underlying Cd tolerance in fungi remain poorly understood and warrant further detailed investigation to elucidate their functional roles (Qu and Zheng, 2024).

CreA, a key regulatory transcription factor involved in carbon catabolite repression (CCR), has been shown to significantly influence fungal growth and metabolic processes under diverse environmental and stress conditions (Tudzynski et al., 2000; Kayikci and Nielsen, 2015). In *Aspergillus nidulans*, CreA has been demonstrated to play a crucial regulatory role in CCR pathways governing carbon metabolism. Conversely, its deletion in *Beauveria bassiana* has been associated with increased amino acid toxicity, impaired cellular development, and reduced virulence under various stress conditions (Fasoyin et al., 2018; Luo et al., 2014). Similarly, DNA methyltransferases (MTases) are known to regulate gene expression and have been implicated in the toxicological effects of heavy metals, including cadmium Cd and zinc (Zn) ions (Genchi et al., 2020; Poirier and Vlasova, 2002; Shafiq et al., 2019). Zinc transporter proteins (ZnT), which are essential for maintaining intracellular zinc homeostasis, have also been directly linked to Cd resistance mechanisms in fungi. Homologous ZnT genes have been shown to confer Cd tolerance in yeast species under various metal stress conditions (Kamizono et al., 1989; Paulsen and Saier, 1997; MacDiarmid et al., 2003). Using enrichment and continuous culture techniques, we successfully isolated four Cd-tolerant fungal strains from Cd-contaminated soil samples collected from heavily contaminated sites. Among these strains, *Paecilomyces lilacinus* (PLN) exhibited the highest Cd tolerance, as determined by growth rate and dry weight analyses under varying Cd^2+^concentrations in culture conditions. Through RNA-Seq and RT-qPCR analyses, 19 differentially expressed genes related to Cd tolerance were identified, including CreA, ZnT, and MTase genes involved in regulatory pathways. These genes are involved in critical cellular processes, including apoptosis, stress response, immune modulation, cell cycle regulation, and intracellular protein transport mechanisms. Second-generation sequencing facilitated the identification of differentially expressed genes within mitochondria and other cellular organelles, thereby providing deeper insights into mechanisms that mitigate oxidative damage and reduce Cd sensitivity in fungal cells. By integrating soil science, environmental toxicology, microbial transcriptomics, molecular biology, and advanced bioinformatics approaches, this interdisciplinary study aims to enhance fungal bioremediation strategies, improve food and agricultural product safety, and provide microbial strain resources for the effective treatment of Cd-contaminated sediments. By identifying and characterizing key Cd-resistant genes, this study lays the groundwork for targeted genetic and biotechnological interventions to enhance fungal Cd resistance, thereby advancing our understanding of Cd tolerance mechanisms in filamentous fungi, particularly PLN strains. Ultimately, these findings significantly contribute to the development of sustainable bioremediation strategies for effectively mitigating heavy metal pollution and improving both environmental and food safety outcomes.

## 2 Materials and methods

### 2.1 Sample preparation

Surface sediment samples (*n* = 10) were collected from different regions in the Changsha section of the *Xiangjiang River Basin* (JZZ, HZS, and SCJ locations). The collected samples were promptly transported to the laboratory for further processing and analysis. One portion of each sample was evenly spread in a clean, well-ventilated area for natural air-drying under ambient conditions. Subsequently, plant residues, gravel, and other debris were carefully removed, followed by grinding and sieving through a 100-mesh sieve for the analysis of heavy metal content in the samples.

Another portion of the samples consisted of three independent 20 g subsamples of fresh sediment collected from JZZ, and each subsample was aseptically transferred into a 250 mL blue reagent bottle for subsequent enrichment and cultivation of microbial strains. The Paecilomyces lilacinus strain used in this study was isolated from Sanchaji (SCJ), in the Changsha section of the Xiangjiang River Basin (N 28°16′57.540″, E 112°57′43.330″), and subsequently deposited at Hunan Normal University. After incubation on potato dextrose agar (PDA) at 28 °C for 7 days, this strain formed velvety colonies ranging in color from light purple to pale pink, with colony diameters ranging from 5.2 to 6.8 cm. Phylogenetic analysis based on ITS and GAPDH gene sequences revealed 93% sequence homology with the type strain P. lilacinus ATCC 10114.

The Changsha section of the Xiangjiang River Basin represents a typical region experiencing severe soil cadmium (Cd) contamination under long-term anthropogenic influence. Purpureocillium lilacinum is a prevalent Cd-tolerant fungus species in this region; however, it is not the dominant species. According to ITS high-throughput sequencing analysis, its relative abundance was predominantly within the range of 0.1%–3% and exhibited a trend of an initial increase followed by a gradual decrease with increasing Cd concentrations. As a stress-tolerant saprophytic and parasitic fungus, it occupies a multifunctional ecological niche in Cd-contaminated soils, integrating Cd resistance, organic matter decomposition, and nematode control functions.

### 2.2 Total Cd determination

Total cadmium was extracted using a tetra-acid digestion system (HCl-HNO_3_-HF-HClO_4_), and its concentration was subsequently determined using inductively coupled plasma mass spectrometry (ICP-MS; Agilent 7700x instrument). All acids used were of trace-metal grade to minimize potential contamination, and all aqueous solutions were prepared using ultrapure water (18.2 MΩ·cm; Milli-Q purification system). Method blanks were included in each digestion batch to monitor background contamination and to ensure analytical accuracy and overall reliability of the measurements. The analytical method for soil cadmium was validated using the national certified reference material (GBW07405); the sample pretreatment and analytical procedures were identical to those employed for the experimental samples, and the relative error between the measured and certified cadmium concentrations was less than 5%, thereby confirming that the method is accurate, precise, and reliable.

Briefly, 0.5 g of air-dried sediment was accurately weighed into a polytetrafluoroethylene (PTFE) crucible and subsequently wetted with a small volume of ultrapure water. Subsequently, 10 mL of concentrated HCl was added, and the mixture was heated to reduce the volume to approximately 5 mL. After cooling, 15 mL of concentrated HNO_3_ was added, and the mixture was heated until the sample solution became viscous. Subsequently, 10 mL of HF was added with frequent agitation to facilitate the removal of silica. Finally, 5 mL HClO_4_ was added, and the mixture was heated until white fumes completely disappeared. The resulting residue was dissolved in 16.7% HNO_3_ solution and subsequently diluted to a final volume of 25 mL.

### 2.3 Available Cd determination

Available Cd was extracted in accordance with the technical specification HJ/T 166-2004 (Kamizono et al., 1989; Paulsen and Saier, 1997; MacDiarmid et al., 2003). Briefly, 5 g of air-dried sediment was accurately weighed into a 100 mL conical flask, and 25 mL of DTPA extracting solution was subsequently added. The mixture was shaken at 25 °C for 2 h under controlled conditions. The supernatant was subsequently filtered and analyzed for Cd concentration using standard analytical procedures. The concentration of available Cd was determined using inductively coupled plasma mass spectrometry (ICP-MS) in accordance with the National Food Safety Standard—Determination of Multi-elements in Foods (GB 5009.268-2016) (Chen et al., 2018).

### 2.4 Screening of Cd-tolerant microorganisms

**Figure 1:**
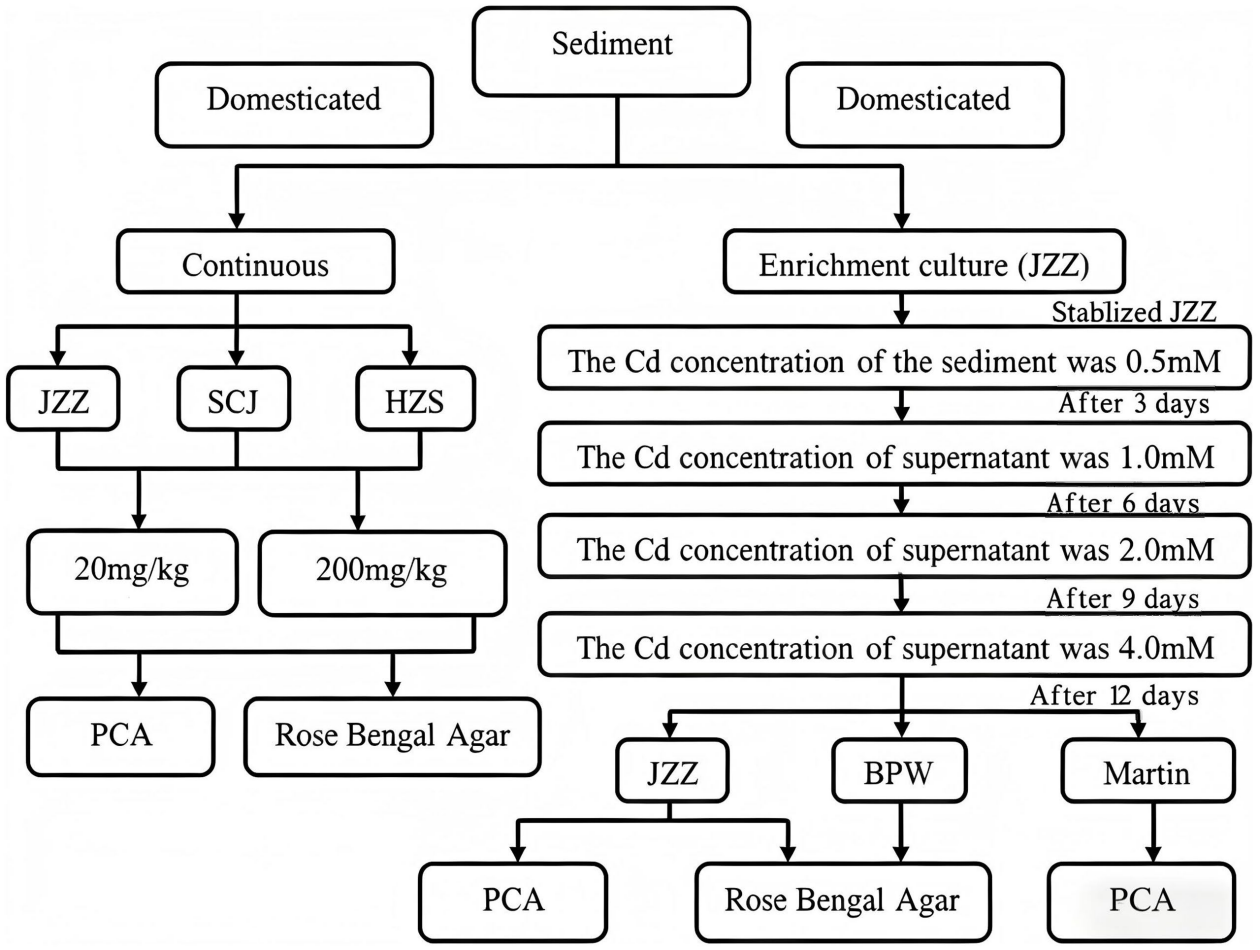
Schematic diagram of the screening process for Cd-tolerant microorganisms.

Enrichment and continuous culture techniques were employed to isolate Cd-tolerant microorganisms under controlled laboratory conditions. Culture screening, purification, and isolation procedures were performed in accordance with the workflow illustrated in Figure 1.

### 2.5 Growth of fungi

The fungi strains were activated by placing the preserved beads into 3 mL of potato dextrose broth (PDB) culture medium and incubating them for 3 days. A volume of 100 μL of each fungal suspension was inoculated into PDB medium containing 0, 2, 4, 6, and 8 mM Cd, followed by incubation for 7 days, after which the samples were filtered, dried, and weighed at a constant weight.

### 2.6 Determination of microbial biomass and culture fluid

Microbial screening was performed using enrichment and continuous culture techniques under controlled laboratory conditions. Selected fungal strains were cultured in potato dextrose broth (PDB) supplemented with varying Cd^2+^ concentrations (0.5, 1, 2, and 4 mM) for 12 days under controlled incubation conditions. The fungal biomass was collected, subsequently filtered, oven-dried, and weighed to a constant weight for accurate quantification.

### 2.7 RT-qPCR experiments

Fungal samples exposed to different Cd concentrations were collected, washed several times with sterile saline solution, and 20 mg of dried mycelial biomass was ground into a fine powder using liquid nitrogen. Total RNA was extracted using the RNeasy Plant Mini Kit (Qiagen, Cat. No. 74904) and complementary DNA (cDNA) was subsequently synthesized using a reverse transcription kit (Promega, United States). The PCR reaction conditions were as follows: 95 °C for 30 s; 40 cycles of 95 °C for 5 s and 60 °C for 34 s. Primer 5.0 software was used to design gene-specific primers based on the identified gene sequences. PCR primers were synthesized by Shanghai Sangon Biotech Co., Ltd. The relative gene expression levels of the target genes were calculated using the 2^−ΔΔCt^ method, and log2 values were used to represent relative difference compared with the control group; *β*-tubulin was used as the internal reference gene. The primer sequences are provided in Supplementary Table 1 for reference.

### 2.8 Plasmid construction and siRNA synthesis

The CreA (1,172 bp), ZnT (1,109 bp), and MTase (941 bp) genes derived from PLN were cloned into the pCMV-HA expression vectors (Clontech, Palo Alto, CA, United States). The pCMV-HA expression vector was purchased from Clontech Laboratories (Palo Alto, CA, United States) for plasmid construction. Restriction enzymes, including KpnI and NotI, were obtained from Fermentas (Thermo Fisher Scientific, Vilnius, Lithuania) and used according to the manufacturer’s instructions. Ex Taq DNA polymerase was purchased from TaKaRa Bio Inc. (Shiga, Japan) and used for PCR amplification according to standard protocols. Monoclonal and polyclonal anti-HA antibodies were purchased from Sigma-Aldrich (St. Louis, MO, United States) and used for protein detection assays. A monoclonal antibody against *β*-tubulin was obtained from Boster Biological Technology Co., Ltd. (Wuhan, China) and used as an internal control for protein expression analysis. Horseradish peroxidase (HRP)-conjugated goat anti-rabbit secondary antibodies were purchased from Jackson ImmunoResearch Laboratories (West Grove, PA, United States) and used for immunodetection. These sequences were validated by Sagon sequencing. Based on the gene-specific sequences of CreA, ZnT, and MTase from *Paecilomyces lilacinus* in the GenBank database, the mRNA initiation codon was selected, corresponding siRNAs were designed, and three siRNAs per gene were synthesized by Guangzhou RiboBio Co., Ltd. (Guangzhou, China).

### 2.9 Cell culture and transfection

HEK293 cells were cultured in high-glucose Dulbecco’s modified Eagle’s medium (DMEM) supplemented with 10% fetal bovine serum (FBS), 2 mmol/L glutamine, 100 U/mL penicillin, and 100 μg/mL streptomycin and maintained at 37 °C in a humidified incubator with 5% CO_2_. When the cells reached 80–90% confluence in 6-well plates, they were washed once with serum- and antibiotic-free DMEM medium prior to transfection. Recombinant plasmids (pCMV-HA-CreA, pCMV-HA-ZnT, and pCMV-HA-MTase) were individually transfected into the cells using Lipofectamine 2000 (Invitrogen, Carlsbad, CA, United States) at a concentration of 2 μg per well, in accordance with the manufacturer’s instructions. After 4 h of transfection, the culture medium was removed and replaced with fresh complete DMEM supplemented with 10% FBS.

### 2.10 Cell proliferation assays

For cell viability assays, 3,000 cells per well were seeded in octuplicate in 48-well plates under standard cell culture conditions. Cells were treated with 1 mg/mL 3-(4,5-dimethylthiazol-2-yl)-2,5-diphenyltetrazolium bromide (MTT) at 37 °C for 4 h at 0, 12, and 24 h under standard incubation conditions. Subsequently, 100 μL of dimethyl sulfoxide (DMSO) was added to completely dissolve the resulting formazan crystals. The absorbance at 490 nm was measured using a calibrated spectrophotometer (UV-2102C; Hangzhou, China) under standardized measurement conditions. For cell survival assays, 50,000 cells per week were seeded in triplicate in 6-well plates containing complete culture medium, and cell numbers were subsequently determined using a hemocytometer at the indicated time points following trypan blue staining.

### 2.11 Fungal cell counting

The preserved fungal strains were inoculated into PDB medium and cultured for 5 days under controlled incubation conditions. After filtration through three layers of sterile gauze, the fungal spores were resuspended in sterile water treated with diethyl pyrocarbonate (DEPC). A 10 μL aliquot of the suspension was taken, and a hemocytometer was used to observe and count the cells under a light microscope. According to the standard calculation formula, the number of cells in the suspension (cells/mL) was calculated as (number of cells counted in five large squares/5) × 25 × 10^4^ × dilution factor.

### 2.12 Electroporation

The spore concentration was adjusted to 1 × 10^7^ spores/mL, and the suspension was inoculated into 200 mL of PDB and incubated with shaking at 300 rpm at 37 °C for 3 h. The swollen conidia were collected and resuspended in 200 mL of cold DEPC-treated water to wash and prepare the spores for electroporation. The spores were then resuspended in l mL of pre-cooled 10% glycerol solution. The conidial suspension was mixed with the corresponding siRNAs and incubated on ice for 30 min to facilitate uptake. The mixture was transferred into a 0.2 cm electroporation cuvette, and electroporation was performed using an electroporator at 1 kV and 200 Ω with appropriate capacitance settings. The transfected fungi were cultured on plate count agar (PCA) supplemented with 4 mM Cd^2+^ for 5 days at 28 °C prior to RNA extraction.

### 2.13 Statistical analysis

Data are presented as the mean ± standard deviation (SD) derived from at least three independent experiments. The statistical significance of differences between groups was assessed using Student’s *t*-test. Values of *p* < 0.05 were considered statistically significant.

## 3 Results

### 3.1 Cd pollution in Xiangjiang river sediments

According to the soil heavy metal standards established in India, the maximum allowable concentrations of cadmium (Cd), copper (Cu), nickel (Ni), lead (Pb), and zinc (Zn) are 3–6 mg/kg, 135–270 mg/kg, 75–150 mg/kg, 250–500 mg/kg and 300–600 mg/kg, respectively (Nagajyoti et al., 2010). To ensure appropriate soil quality for agricultural production and normal plant growth, pollution levels were classified as light, moderate, or severe according to the three-level soil quality standards implemented in China (Chen et al., 2018).

To determine the sampling sites, 10 different locations within the Changsha section of the *Xiangjiang River Basin* were selected and labeled as JZZ_1-4_, SCJ_1-4_, and HZS_1-2_, as shown in Table 1. To assess the heavy metal content in sediments of the *Xiangjiang River Basin*, the total concentrations of four heavy metals—Cd, copper, lead, and zinc—as well as their bioavailable fractions were determined at the 10 sampling sites. Analysis of sediment samples collected from different locations within the Changsha section of the *Xiangjiang River Basin* revealed significant Cd contamination. Cd concentrations exceeded the national three-level soil quality standard (1 mg/kg) at all sampling sites, with values ranging from 2.02 mg/kg to 23.4 mg/kg. The highest Cd concentration (23.4 mg/kg) was detected at sampling site JZZ1. Three samples exhibiting a wide concentration gradient and a high range ratio were subsequently selected for further experimental were subsequently selected for further species. The highest Cd concentration (23.4 mg/kg) was detected at site JZZ_1_ (28°11′55.663″, 112°57′06.196″), whereas concentrations of 2.02 mg/kg and 10.5 mg/kg were observed at site SCJ_4_ (28°16′57.540″, 112°57′43.330″) and HZS_1_ (28°10′00.588″, 112°57′02.241″), respectively. The potential ecological risk index (RI) further confirmed that Cd posed the highest hazard among the analyzed heavy metals (Cd > Cu > Pb > Zn), with a risk factor ( $E_{r}^{i}$ ) of 1,478, significantly exceeding the national standard threshold. These findings underscore the severity of Cd pollution in the sediments, thereby justifying the focus on Cd-resistant microbial strains for further investigation.

**Table 1:** Results of potential ecological risk assessment.

Sample	North	East	Cd (mg/kg)	Cu (mg/kg)	Pb (mg/kg)	Zn (mg/kg)	$E_{r}^{i}$				RI
							Cd	Cu	Pb	Zn	
JZZ1	28°11′55.663″	112°57′06.196″	23.8	108.0	53.80	523.5	1,428	32.3	10.8	7.3	1478.4
JZZ2	28°12′06.849″	112°57′07.377″	6.90	93.80	48.40	319.9	414	28.1	9.70	4.5	456.3
JZZ3	28°11′56.048″	112°57′45.390″	14.6	130.9	73.30	554.5	876	39.2	14.7	7.7	937.6
JZZ4	28°12′05.956″	112°57′46.846″	6.70	134.1	136.5	490.0	402	40.1	27.3	6.8	476.2
SCJ1	28°16′36.007″	112°57′05.440″	2.90	71.00	11.41	256.4	174	21.3	2.30	3.6	201.2
SCJ2	28°16′37.524″	112°57′04.521″	10.9	86.70	26.00	346.5	654	26.0	5.20	4.8	690.0
SCJ3	28°16′44.120″	112°57′01.719″	21.2	126.5	105.9	571.2	1,272	37.9	21.2	8.0	1339.1
SCJ4	28°16′57.540″	112°57′43.330″	2.02	126.3	55.40	278.9	121.2	37.8	11.1	3.9	174.0
HZS1	28°10′00.588″	112°57′02.241″	10.5	111.3	105.9	516.4	630	33.3	21.2	7.2	691.7
HZS2	28°08′45.854″	112°56′51.969″	15.2	113.6	61.50	468.1	912	34.0	12.3	6.5	964.8
Average	28°11′55.663″	112°57′06.196″	11.5	110.2	67.80	432.5	690	33.0	13.6	6.0	742.6

### 3.2 Screening of Cd-tolerant microorganisms at three sampling sites

**Figure 2:**
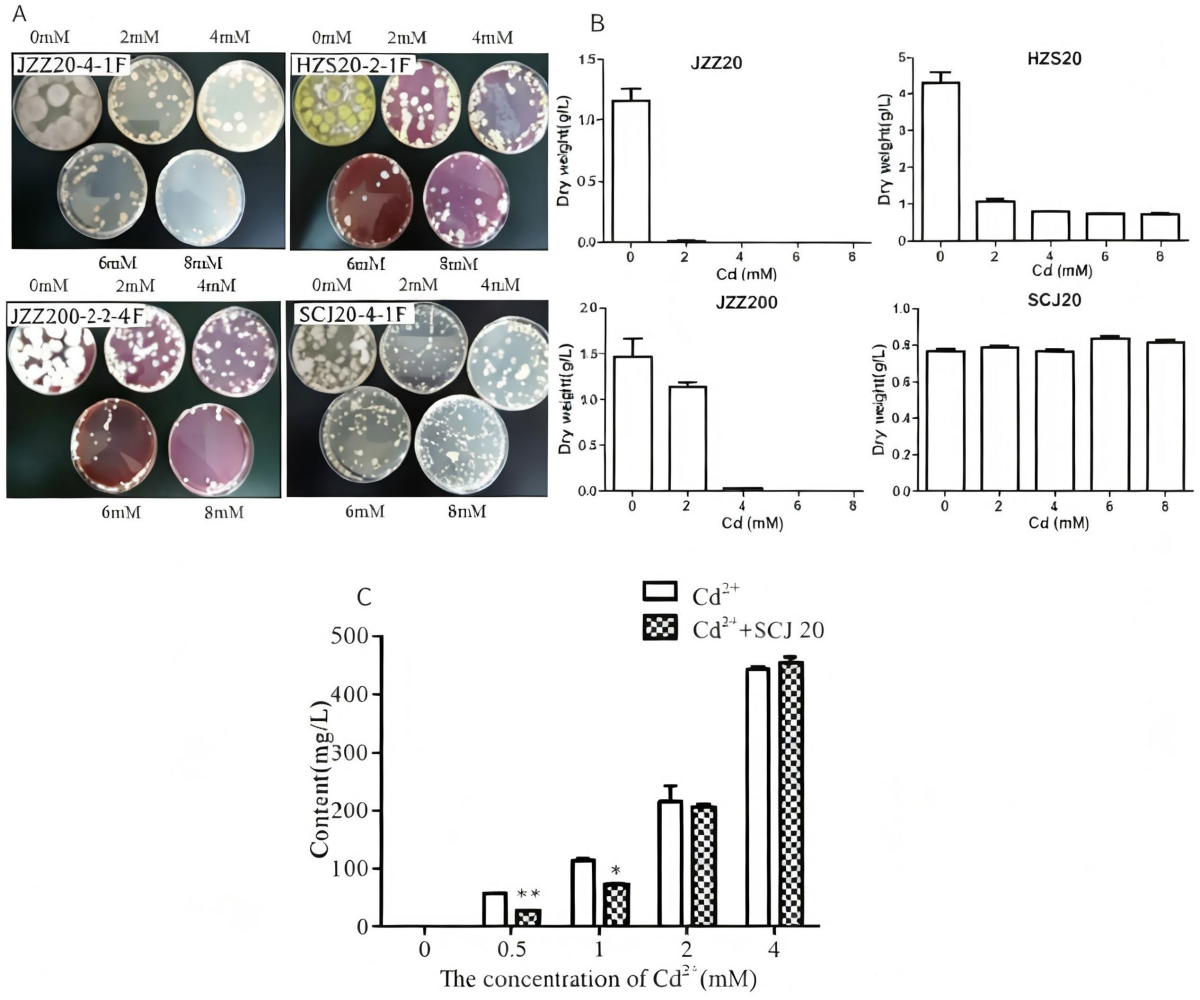
Growth of Cd-tolerant microorganisms under different Cd^2+^ concentrations. **(A)** Screening of microorganisms on Cd-containing plates. **(B)** Dry weight of four microbial strains. **(C)** Effects of different Cd concentrations in the medium on the Cd^2+^ content of PLN (*p < 0.05; **p < 0.01).

To screen microorganisms with high Cd tolerance, pure strains were selected following repeated continuous culture on plate count agar (PCA) and Rose Bengal agar media. The isolates were cultured on plates containing Cd at concentrations of 0, 2, 4, 6, and 8 mM, and those capable of growth at higher concentrations were selected for preservation and subsequently sent to Shanghai Sangon Biotech Co., Ltd. for sequencing (Figure 2A). To further investigate.

The Cd resistance of the selected microorganisms was determined by growing cultures in PDB medium containing Cd at concentrations of 0, 2, 4, 6, and 8 mM for 7 days at 30 °C, followed by filtration, drying, and weighing to a constant weight. The results showed that, with increasing Cd concentration, only strain SCJ_20_ maintained its growth at 8 mM, further indicating its strong tolerance under high Cd stress conditions. Taken together, these data indicate that SCJ_20_ exhibited the highest Cd tolerance (Figure 2B). Based on sequencing results, the internal transcribed spacer (ITS) sequence of SCJ20 was compared with known sequences in the GenBank database and identified as *Paecilomyces lilacinus* (PLN), which was selected for f further investigation. After 5 days of culture with 0.5 mM CdCl_2_, the Cd^2+^ concentration in the culture medium was significantly reduced by 52.2% compared with the control group (*p* < 0.01). When 1 mM CdCl_2_ was applied, the Cd^2+^ concentration in the culture medium was significantly reduced by 37.3% (*p* < 0.05). When the CdCl_2_ concentration exceeded 2 mM, the Cd^2+^ concentration in the culture medium remained unchanged. These data indicate that PLN reduces Cd^2+^ levels in the medium through adsorption at low CdCl_2_ concentrations, Cd^2+^ may be adsorbed and subsequently released, resulting in no net change in Cd^2+^ levels in the medium (Figure 2C).

### 3.3 Differentially expressed genes genes involved in the Cd tolerance mechanism of PLN

**Figure 3:**
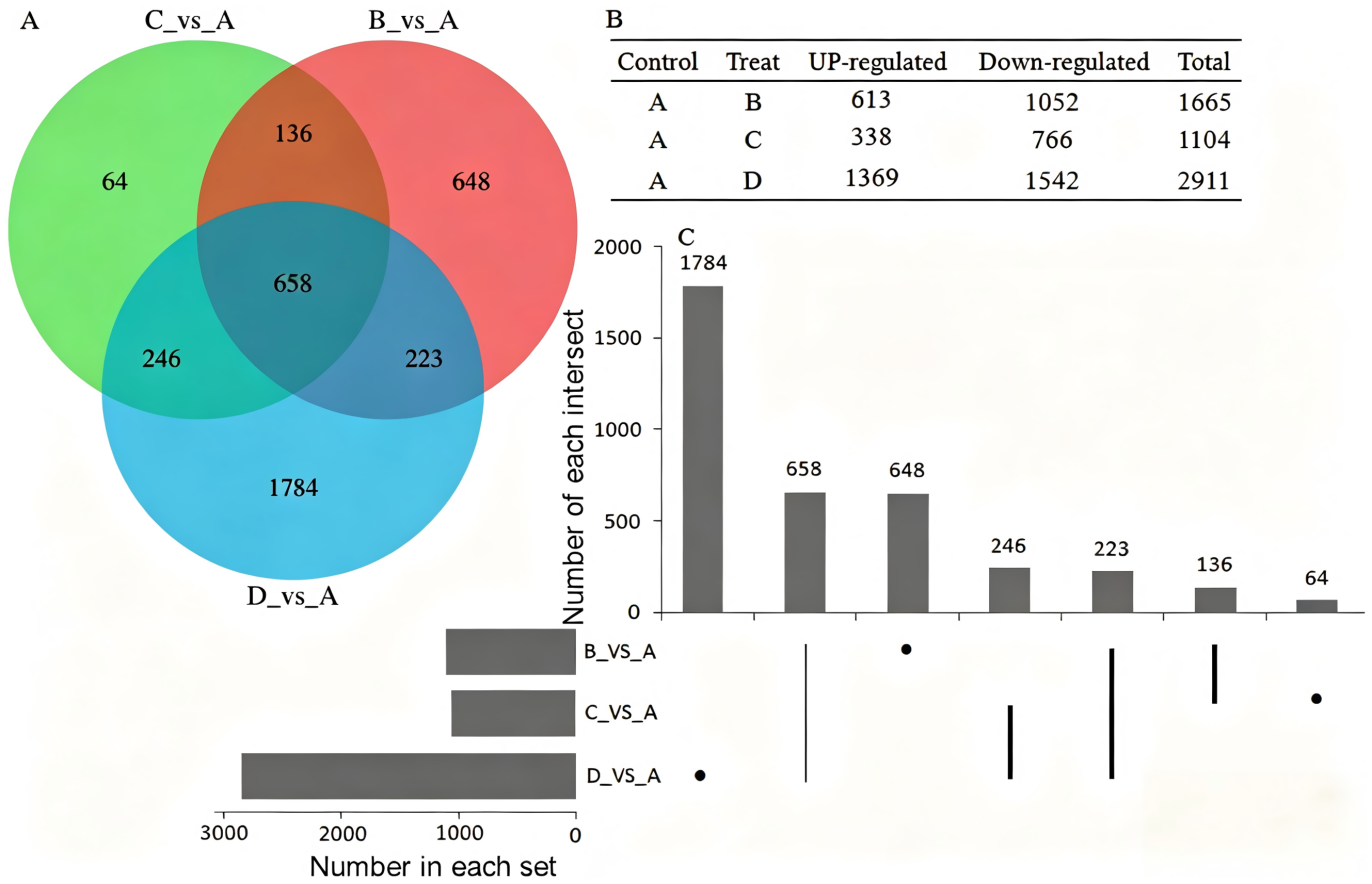
Statistical analysis of differential gene expression. **(A)** Overlap of differentially expressed genes among the treatment groups. **(B)** Quantitative distribution of differentially expressed genes in each group. **(C)** Group-specific differentially expressed genes were identified in each treatment group.

To elucidate the molecular mechanisms underlying Cd^2+^ tolerance in PLN, transcriptome sequencing was performed using the Illumina HiSeq platform on four groups of PLN samples treated with different Cd^2+^ concentrations (0 mM [A], 2 mM [B], 4 mM [C], and 8 mM [D]), with three biological replicates for each treatment group. Differential gene expression analysis was conducted using DESeq software (see Supplementary file detailed analysis). Compared with the control group (0 mM Cd^2+^), the experimental groups (2, 4, and 8 mM Cd^2+^) exhibited 1,665, 1,104, and 2,911 differentially expressed genes (DEGs), respectively (Supplementary Tables 2–4). Among these, 613, 338, and 1,369 genes were upregulated, whereas 1,052, 766, and 1,542 genes were downregulated in the 2, 4, and 8 mM Cd^2+^ treatment groups, respectively. A total of 658 DEGs were shared among all three treatment groups (Figure 3B). Further analysis revealed 648, 64, and 1,784 treatment-specific DEGs in the 2, 4, and 8 mM Cd^2+^ groups, respectively, with 658 DEGs overlapping among these groups (Figures 3A,C).

### 3.4 Functional annotation and pathway enrichment of differentially expressed genes involved in the Cd tolerance mechanism of PLN

**Figure 4:**
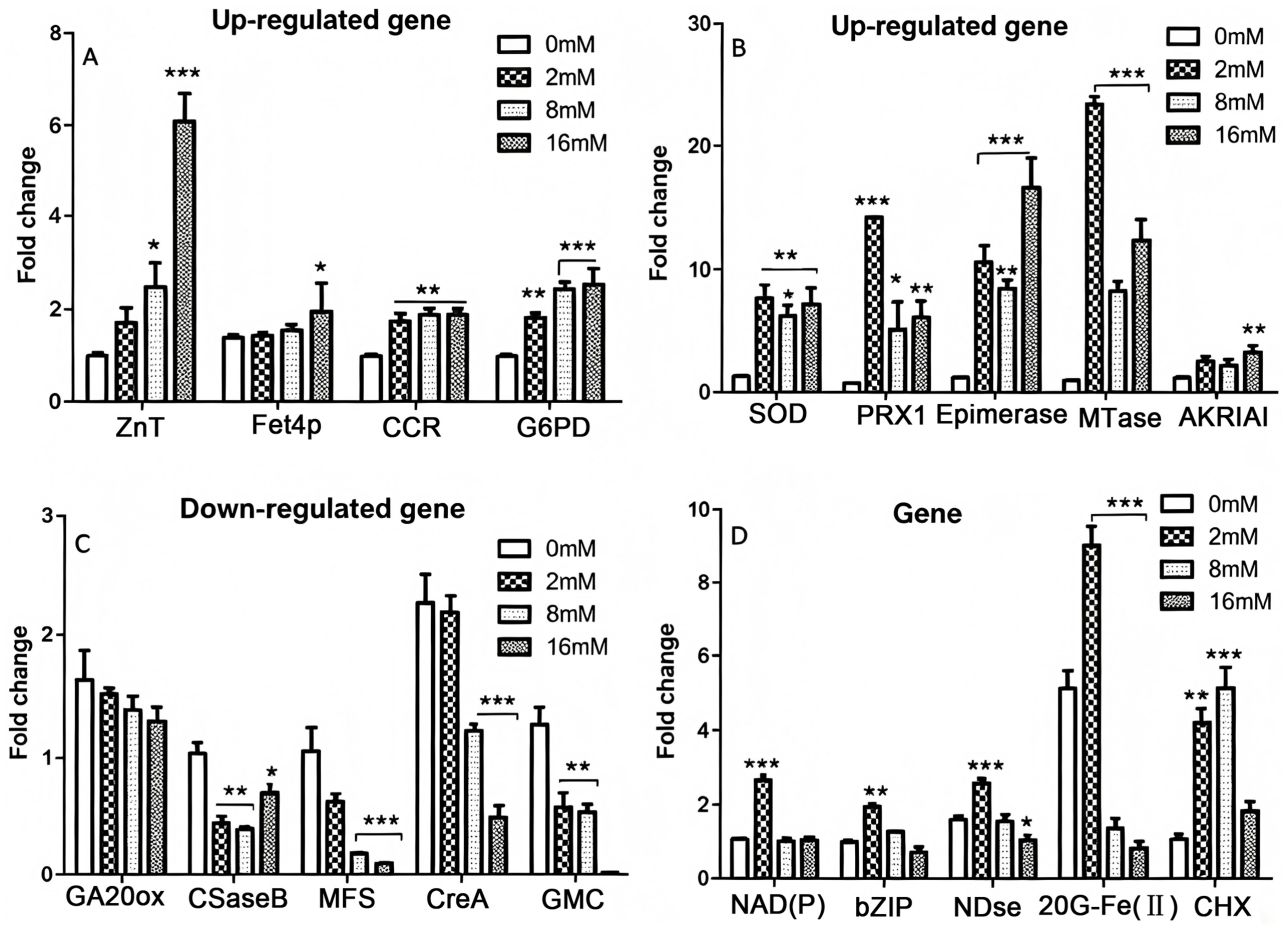
Changes in the expression of differentially expressed genes associated with Cd tolerance. **(A)** Upregulated genes with increasing Cd^2+^ concentrations. **(B)** Upregulated gene transcription levels in response to increasing Cd^2+^ concentrations. **(C)** Genes with downregulated transcription levels in response to increasing Cd^2+^ concentrations. **(D)** Genes whose transcriptional expression levels were initially upregulated and subsequently downregulated in response to increasing Cd^2+^ concentrations.

Based on the differential gene expression data, 19 significantly differentially expressed genes associated with stress resistance factor, antioxidant systems, Cd transport processes, and Cd toxicity were selected for further analysis. Real-time quantitative SYBR Green RT-qPCR was performed under different Cd treatment conditions to validate the magnitude of gene expression changes in PLN; the expression trends of the 19 selected genes were consistent, and their basic information and functional annotations are presented in Table 1. Blast_2_GO software was employed for functional annotation (Supplementary Tables 5–7), and pathway enrichment analysis of differentially expressed genes was conducted using KEGG and other databases (Supplementary Tables 8–10). The results indicated that the differentially expressed genes were closely associated with transmembrane transport, redox processes, carbohydrate metabolism, and detoxification mechanisms. Using a fold change ≥±2 and a false discovery rate (FDR) <0.01 as screening criteria, RT-qPCR was performed to validate the 19 differentially expressed genes; the observed gene expression trends were generally consistent with the transcriptome sequencing predictions, indicating that the transcriptomic data obtained in this study are reliable. Among these, the novel genes CreA, ZnT, and MTase were the most significantly differentially expressed (Figure 4). The correlation of gene expression fold changes between RNA-seq and RT-qPCR was assessed, revealing a strong positive correlation (*R*^2^ = 0.92, *p* < 0.001).

### 3.5 Effects of CreA, ZnT, and MTase on PLN growth under cd stress

**Figure 5:**
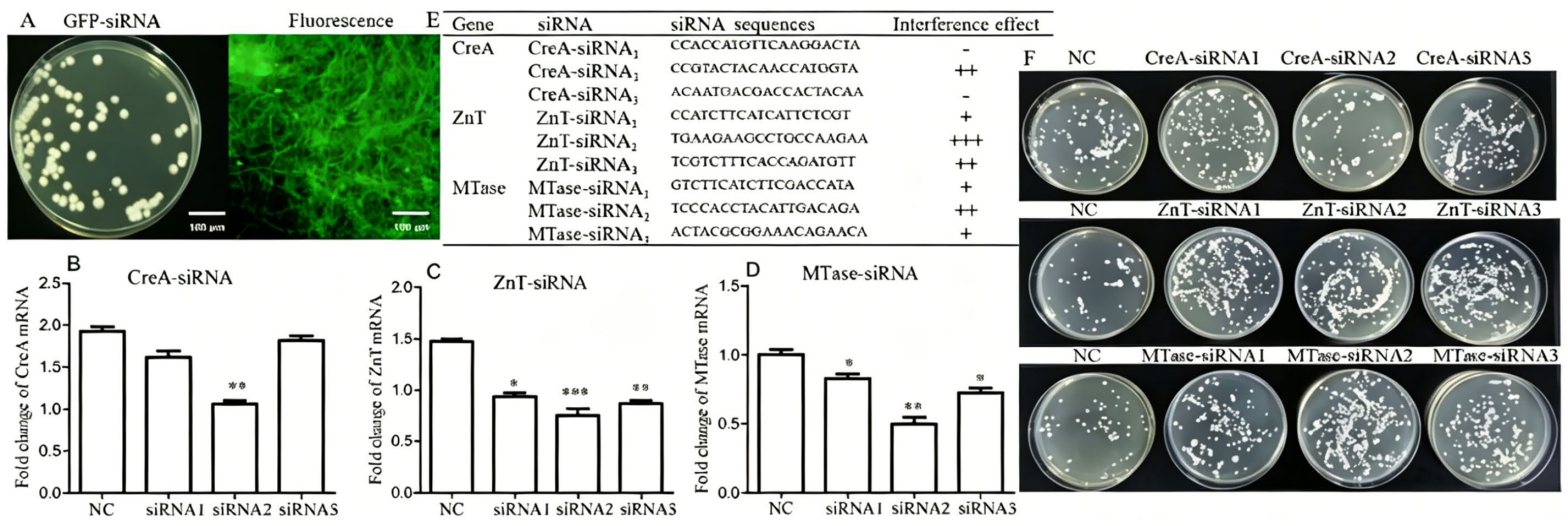
Effects of CreA-, ZnT-, and MTase-targeting siRNAs on PLN growth. **(A)** Positive control showing strong green fluorescent protein (GFP) fluorescence. **(B)** siRNA-mediated knockdown of CreA at the mRNA level. **(C)** siRNA-mediated knockdown of ZnT at the mRNA level. **(D)** siRNA-mediated knockdown of MTase at the mRNA level. **(E)** Quantitative analysis of siRNA interference efficiency for CreA, ZnT, and MTase. **(F)** Effects of CreA-, ZnT-, and MTase-targeting siRNAs on PLN growth (*p < 0.05; **p < 0.01; ***p < 0.001).

To investigate the functional roles of CreA, ZnT, and MTase in PLN growth under Cd^2+^ stress, gene-specific siRNAs targeting these genes were introduced into PLN cells via electroporation under optimized conditions (1 kV and 200 Ω). Successful siRNA transfection was confirmed by the presence of green fluorescence in the positive control group (Figure 5A). After 5 days of culture, RT-qPCR analysis revealed that the mRNA expression levels of CreA, ZnT, and MTase were significantly reduced following transfection with CreA-siRNA_2_, ZnT-siRNA_1/2/3,_ and MTase-siRNA_2_ (*p* < 0.01), thereby confirming the efficiency of the siRNA sequences targeting CreA, ZnT, and MTase genes (Figures 5B–E). PLN cells transfected with CreA-siRNA, ZnT-siRNA, and MTase-siRNA were cultured on PCA plates supplemented with 4 mM Cd^2+^. CreA-siRNA_2_ significantly inhibited PLN growth, whereas ZnT-siRNA_1/2/3_ and MTase-siRNA_2_ significantly promoted PLN growth compared with the negative control (NC-siRNA) (Figure 5F). Thus, under specific Cd^2+^concentrations, CreA-siRNA inhibits PLN growth, whereas ZnT-siRNA and MTase-siRNA promote its growth.

### 3.6 Western blot analysis of CreA, ZnT, and MTase overexpression

**Figure 6:**
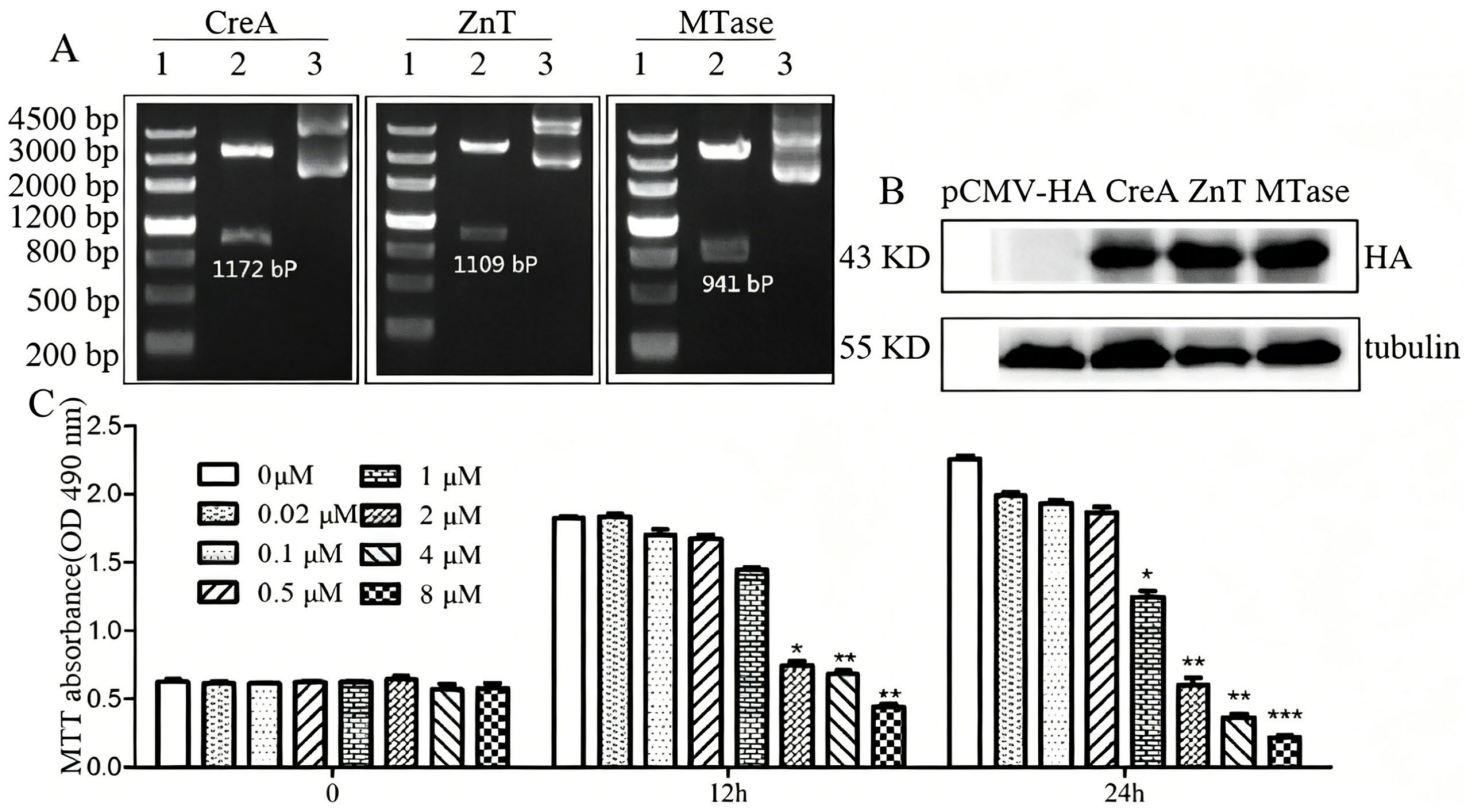
Expression of CreA, ZnT, and MTase proteins in HEK293 cells. **(A)** CreA, ZnT, and MTase genes were cloned into the pCMV-HA expression vector and confirmed by restriction enzyme digestion and agarose gel electrophoresis. **(B)** Protein expression was analyzed by western blot following the overexpression of CreA, ZnT, and MTase in HEK293 cells. **(C)** Effects of Cd^2+^ on HEK293 cell viability, as determined by MTT assays.

To further characterize the functional roles of CreA, ZnT, and MTase, these genes were cloned into the pCMV-HA expression vector and subsequently overexpressed in HEK293 cells (Figure 6A). Western blot analysis further confirmed the successful overexpression of CreA (46 kDa), ZnT (44 kDa), and MTase (40 kDa) proteins in HEK293 cells (Figure 6B). To determine the optimal Cd^2+^ concentration for evaluating its effects on HEK293 cell viability, MTT assays were subsequently conducted. HEK293 cells were treated with a range of Cd^2+^ concentrations (0, 0.02, 0.1, 0.5, 1, 2, 4, and 8 μM) at time points of 0, 12, and 24 h. The results demonstrated that Cd^2+^ inhibited HEK293 cell growth in a concentration- and time-dependent manner. Specifically, at Cd^2+^ concentrations below 2 μM, the inhibitory effect on cell growth was relatively minimal (Figure 6C). Therefore, a Cd^2+^ concentration of 2 μM was selected as the optimal condition for subsequent experiments.

### 3.7 Effects of CreA, ZnT, and MTase overexpression on HEK293 cell growth under defined Cd^2+^ conditions

**Figure 7:**
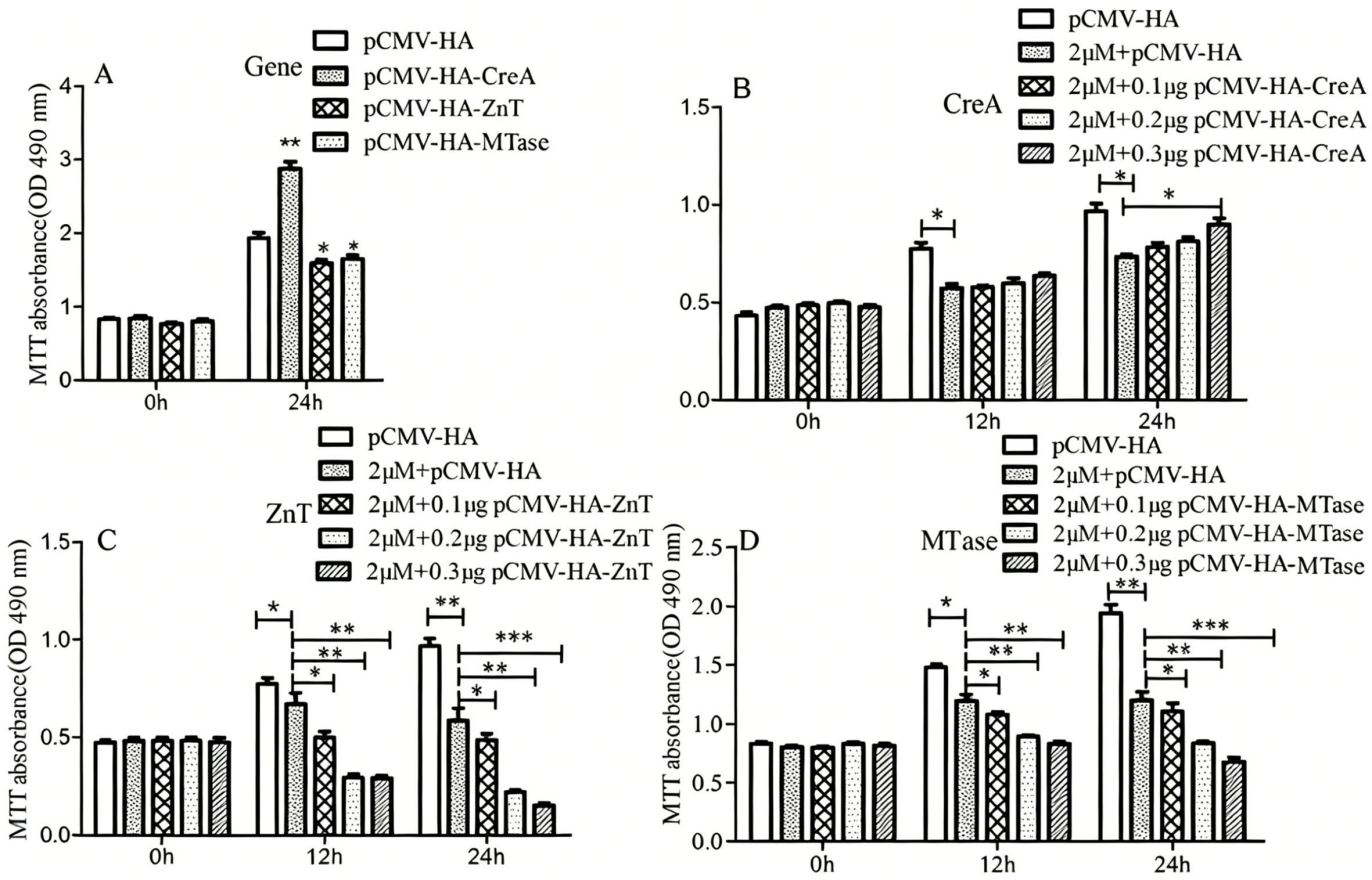
MTT assays showing that CreA overexpression enhances the effects of Cd^2+^ on HEK293 cell viability. **(A)** Cell viability following overexpression of CreA, ZnT, and MTase genes. **(B)** HEK293 cells were transfected with pCMV-HA-CreA and treated with a defined concentration of Cd^2+^, which exhibited increased cell viability. **(C)** HEK293 cells transfected with pCMV-HA-ZnT and treated with a defined concentration of Cd^2+^ exhibited reduced cell viability. **(D)** HEK293 cells transfected with pCMV-HA-MTase and treated with a defined concentration of Cd^2+^ exhibited reduced cell viability. (*p < 0.05; **p < 0.01; ***p < 0.001).

The effects of CreA, ZnT, and MTase overexpression on HEK293 cell viability under Cd^2+^ stress were evaluated using MTT assays. Compared with the control group, CreA overexpression significantly enhanced HEK293 cell viability over time (*p* < 0.01), indicating a substantial increase in cell growth under Cd^2+^ stress conditions. Conversely, overexpression of ZnT and MTase significantly reduced HEK293 cell viability (*p* < 0.05), suggesting a protective role in mitigating Cd^2+^-induced toxicity (Figure 7A). Specifically, under treatment with 2 μM Cd^2+^, CreA overexpression significantly increased HEK293 cell proliferation (*p* < 0.05), whereas overexpression of ZnT and MTase significantly decreased cell proliferation (*p* < 0.01) (Figures 7B–D). These results suggest that Cd^2+^ regulates the expression of CreA, ZnT, and MTase, which in turn modulate cellular Cd^2+^ tolerance mechanisms.

## 4 Discussion and conclusions

Cd-resistant microorganisms deploy a versatile array of mechanisms to counteract heavy metal stress, as extensively documented in previous studies (Tian et al., 2015; Zhai et al., 2014; Zhai et al., 2016; Cavalcanti Luna et al., 2015; Baptista et al., 2009; Chatterjee et al., 2024). Recent advances have further expanded this repertoire, including magnetic biochar–*Bacillus cereus* composites (Zhang Y. et al., 2025) and phosphate rock–microbe–plant synergistic systems (Wang et al., 2023). Although both strategies immobilize >70% of Cd under controlled conditions, the former lacks genetic-level elucidation, whereas the latter leaves microbial gene functions largely uncharacterized. Therefore, addressing this knowledge gap is imperative for advancing Cd bioremediation strategies (Shindhal et al., 2021). By integrating high-throughput transcriptomic sequencing with siRNA-mediated knockdown and targeted gene overexpression, we not only corroborate the exceptional efficacy of *Penicillium oxalicum* ZP6 reported by Zheng et al. (2022), but also identify CreA as a master transcriptional regulator that orchestrates the upregulation of metal efflux systems under Cd stress. More importantly, we demonstrate that the CreA-ZnT-MTase regulatory axis can be genetically modulated to sustain remediation performance under fluctuating soil conditions, thereby addressing a long-standing bottleneck that has limited the practical application of laboratory-scale MICP/MIPP technologies (Zheng et al., 2021).

Using state-of-the-art bioinformatics pipelines, we annotated and classified differentially expressed genes, thereby identifying CreA, ZnT, and MTase as key genetic determinants of Cd tolerance in PLN (Li et al., 2024). ZnT transporters alleviate Cd toxicity by regulating intracellular Zn^2+^ levels (Vaher et al., 2001), thereby mitigating ionic mimicry, a mechanism recently confirmed by studies on ZnT-mediated metal ion homeostasis under heavy metal stress (Firincă et al., 2025). In contrast, metallothioneins (MTase) sequester free Cd^2+^ into non-toxic thiolate clusters, a process further supported by recent spectroscopic analyses revealing the cooperative formation of Cd-containing MT complexes (Zhang Y. et al., 2025). These findings not only expand the functional landscape of Cd-resistance genes across taxa, as increasingly explored in cross-kingdom comparative studies, but also provide precise molecular targets for future engineering efforts aimed at enhancing microbial bioremediation efficacy.

This study systematically delineates a complex, multilayered transcriptional regulatory network underlying cadmium tolerance in *Paecilomyces lilacinus*, identifying the transcription factor CreA as a key central regulatory hub that modulates downstream ZnT transporter activity and MTase-mediated metallothionein gene expression. These findings not only further consolidate the remarkable bioremediation capacity of strain ZP6 reported previously but also, for the first time, comprehensively elucidate its underlying molecular genetic basis, thereby addressing a critical knowledge gap in current cadmium remediation strategies. Conventional approaches, such as magnetic biochar composites and phosphate rock-microbe-plant systems, often achieve measurable Cd removal efficiency without fully elucidating the underlying genetic or regulatory mechanisms (Zhang Y. et al., 2025, Kambe et al., 2015). By integrating full-transcriptome high-throughput sequencing with gene silencing and targeted gene overexpression approaches, we demonstrate that CreA alleviates carbon catabolite repression under Cd stress, thereby activating zinc efflux and cadmium sequestration pathways that collectively confer robust cadmium resistance. A key novel insight is that fine-tuning the CreA-ZnT-MTase regulatory axis stabilizes remediation efficiency under fluctuating soil conditions, thereby providing a feasible strategy to overcome the long-standing bottleneck that has limited the field-scale translation of laboratory-based MICP/MIPP bioremediation technologies. Beyond transcriptional regulation, we further characterized organelle-specific transcriptomic responses, particularly mitochondrial transcriptomic profiles, revealing conserved molecular circuits that alleviate oxidative stress and suppress apoptosis under cadmium-induced cytotoxic conditions. This high-resolution subcellular perspective significantly enhances the mechanistic understanding of cellular responses to heavy metal exposure, thereby enabling more accurate prediction, prevention, and management of cadmium toxicity. At the translational level, these findings extend beyond environmental bioremediation to potential biomedical applications. At the cellular level, CreA overexpression or ZnT/MTase silencing markedly mitigates Cd-induced damage in kidney and bone models, suggesting promising gene-targeted therapeutic strategies for Cd-associated osteomalacia and renal dysfunction. With the convergence of biotechnology and precision medicine, early diagnosis and targeted interventions for heavy metal poisoning are gradually transitioning from conceptual frameworks to clinically feasible strategies. Nevertheless, several important limitations warrant careful consideration. First, cadmium tolerance is a highly complex and multifactorial process involving not only transcriptional regulation but also non-coding RNA modulation, post-translational modifications, and metabolite-mediated feedback mechanisms. Our current understanding remains largely restricted to the transcriptomic level; therefore, integrating proteomic and metabolomic analyses will be essential for constructing a comprehensive multidimensional regulatory network. Second, although a genetic manipulation system for *Paecilomyces lilacinus* has been established, its ecological adaptability, competitive fitness, and long-term colonization capacity in complex soil environments remain poorly characterized. Laboratory efficiency does not necessarily guarantee field performance, as indigenous microbiota and abiotic factors (e.g., pH and organic matter) may disrupt the stability of engineered genetic circuits. From an ecological safety perspective, potential off-target effects also require rigorous and systematic evaluation. As a global transcriptional regulator, modification of CreA may induce unintended metabolic shifts or fitness costs that could alter soil microbial community structure and function. Long-term field trials and systematic ecological risk assessments are therefore essential to verify the stability, safety, and sustainability of genetically engineered strains. Despite these constraints, this study establishes a comprehensive interdisciplinary framework integrating soil science, environmental toxicology, microbial transcriptomics, molecular biology, and bioinformatics, thereby providing a holistic strategy for the source control of heavy metal contamination. Intervention at the base of the food chain enhances environmental security and agricultural product quality while providing genetically tractable microbial strains and gene cassettes for *in situ* remediation of Cd-contaminated soils and sediments.

In conclusion, this study elucidates the CreA-centered regulatory cascade governing cadmium tolerance in *Paecilomyces lilacinus*, thereby bridging the mechanistic gap between laboratory-scale remediation efficacy and field-level application, and opening new avenues for environmental bioremediation and gene-targeted therapeutic strategies. Further translation of this integrated strategy from bench to field requires sustained interdisciplinary collaboration, rigorous ecological assessment, and iterative technical optimization, ultimately contributing to sustainable agriculture development and long-term public health protection.

## Data Availability

The data presented in the study are deposited in the NCBI BioProject repository, accession number PRJNA1464123.
