# Supplementary figures and images for: Functional characterization of CreA, ZnT, and MTase as key regulators of cadmium resistance in Paecilomyces lilacinus (part 2 of 2)
Source: Front Microbiol. 2026 May 14;17:1792636. doi: 10.3389/fmicb.2026.1792636 (PMC13218079; doi:10.3389/fmicb.2026.1792636)

# Read Distribution

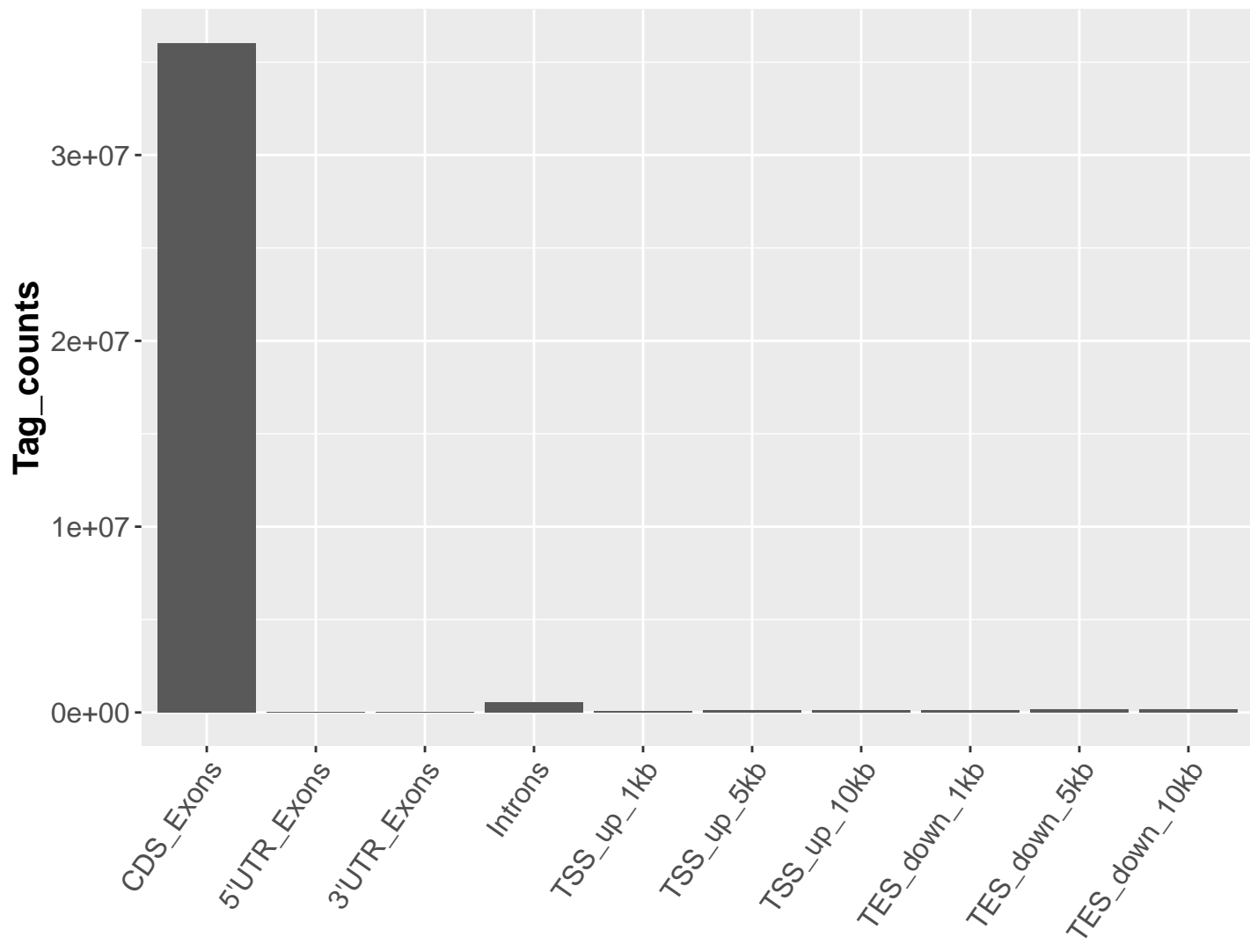

Supplement: Supplementary file 1 [file Data_Sheet_1.ZIP › 04_MapQC/read_distribution/B2.read_distribution.pdf]

# Read Distribution

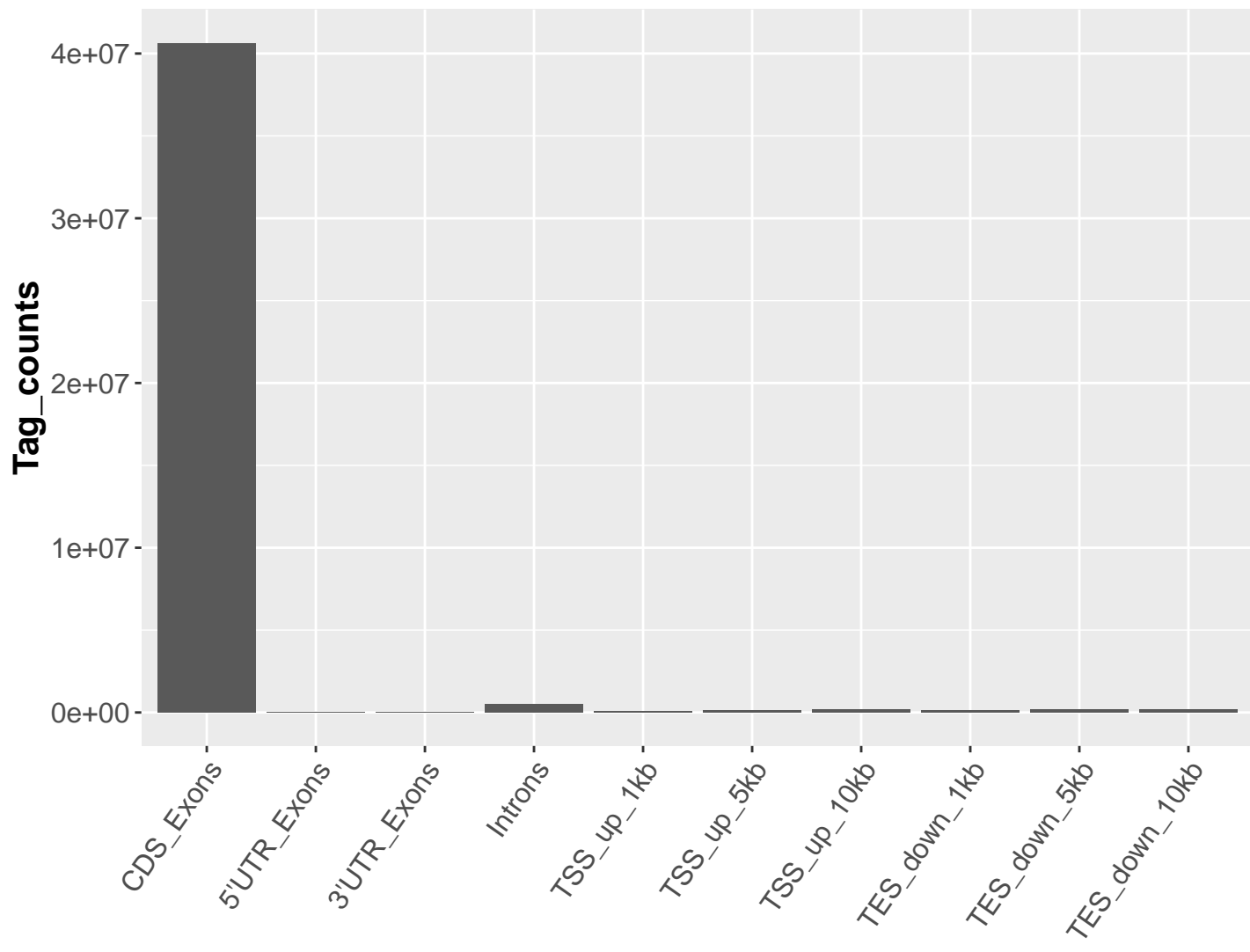

Supplement: Supplementary file 1 [file Data_Sheet_1.ZIP › 04_MapQC/read_distribution/B3.read_distribution.pdf]

# Read Distribution

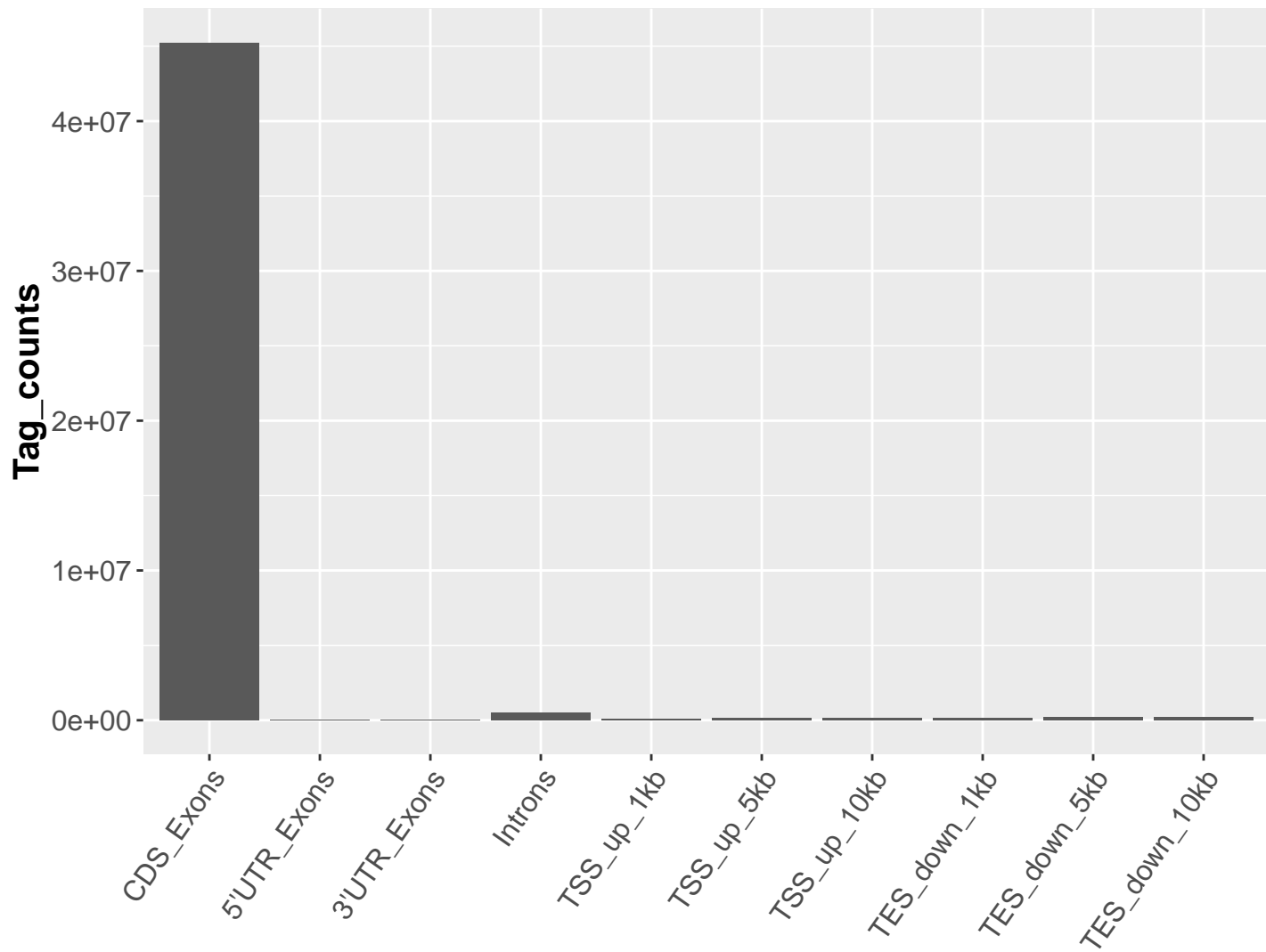

Supplement: Supplementary file 1 [file Data_Sheet_1.ZIP › 04_MapQC/read_distribution/C1.read_distribution.pdf]

# Read Distribution

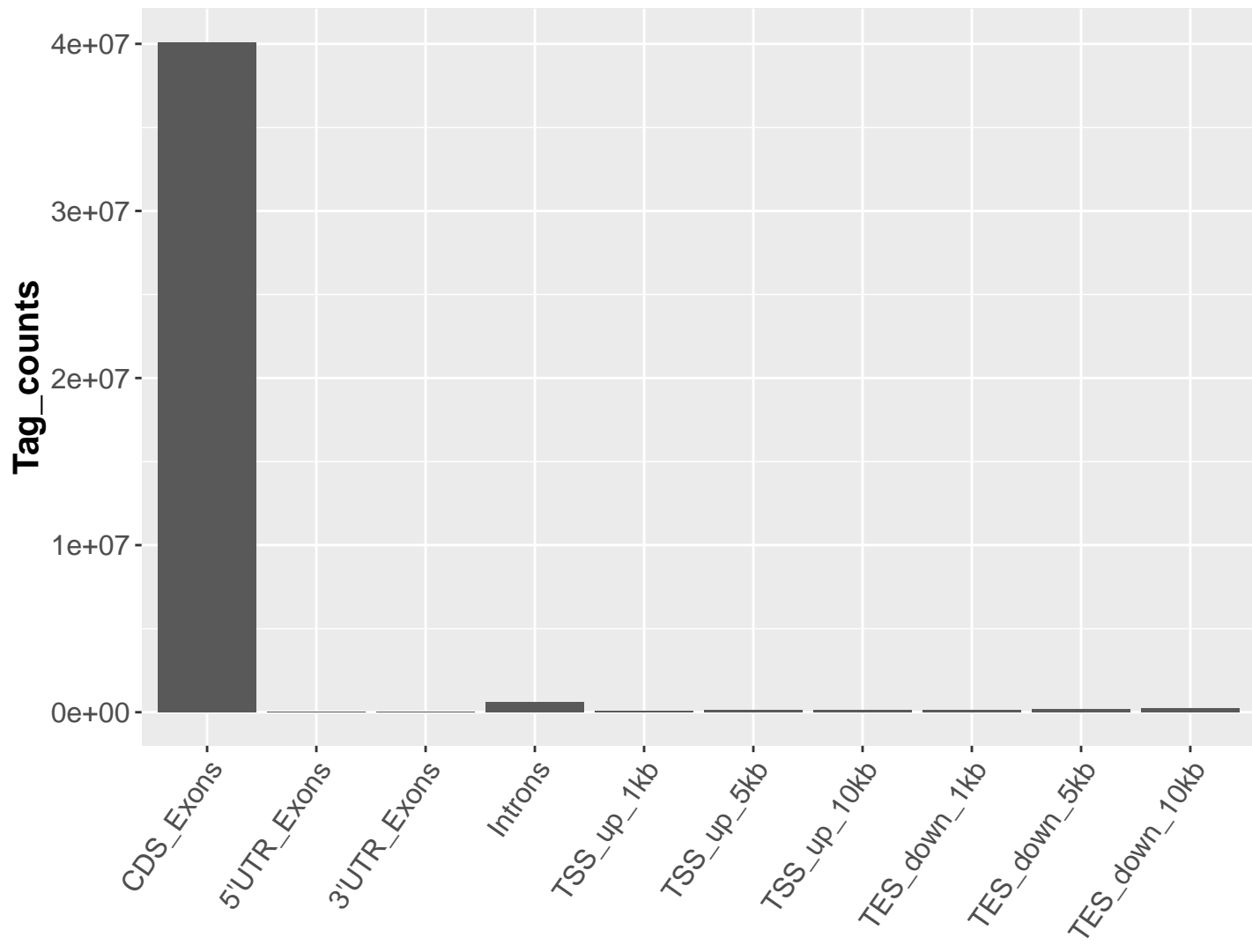

Supplement: Supplementary file 1 [file Data_Sheet_1.ZIP › 04_MapQC/read_distribution/C2.read_distribution.pdf]

# Read Distribution

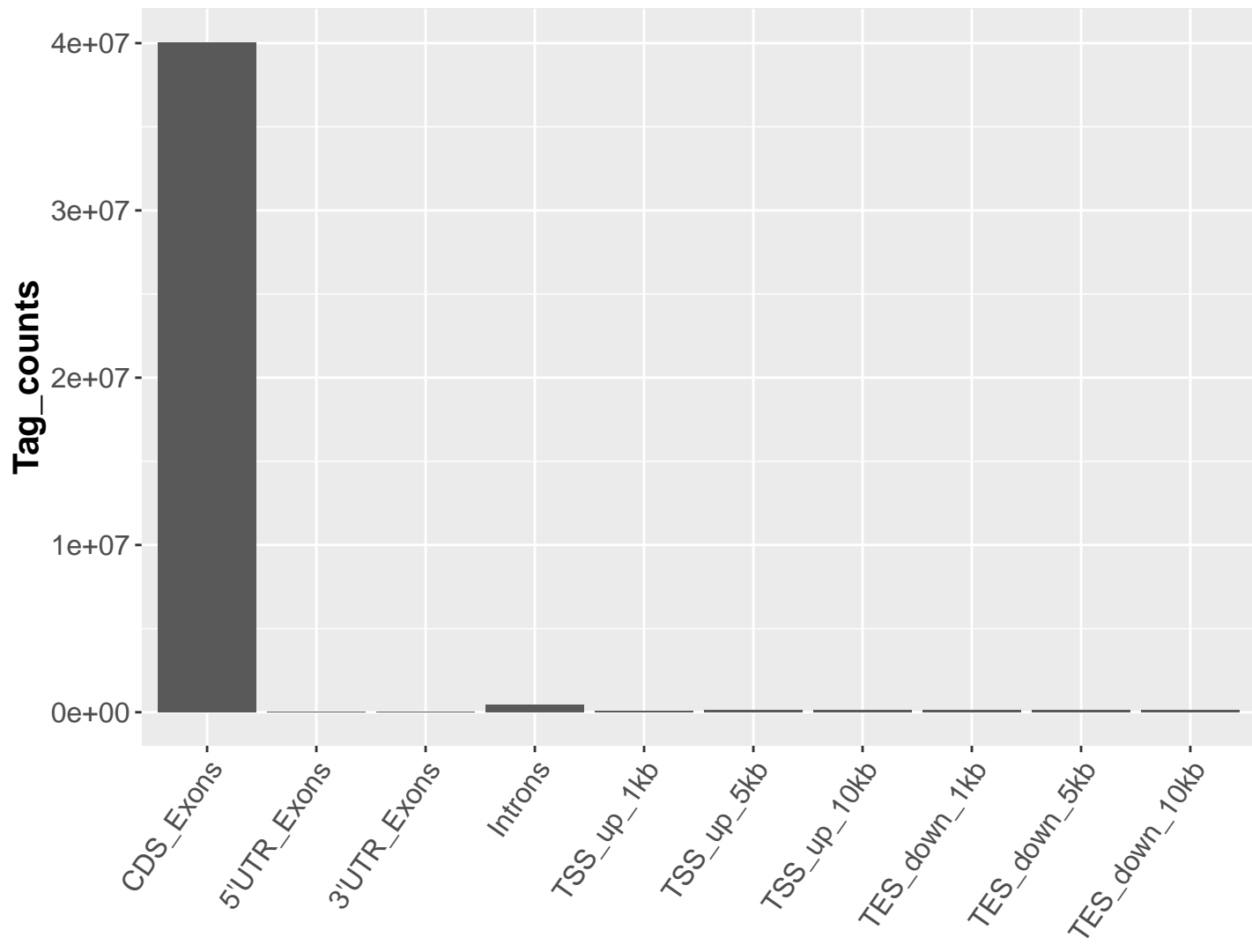

Supplement: Supplementary file 1 [file Data_Sheet_1.ZIP › 04_MapQC/read_distribution/C3.read_distribution.pdf]

# Read Distribution

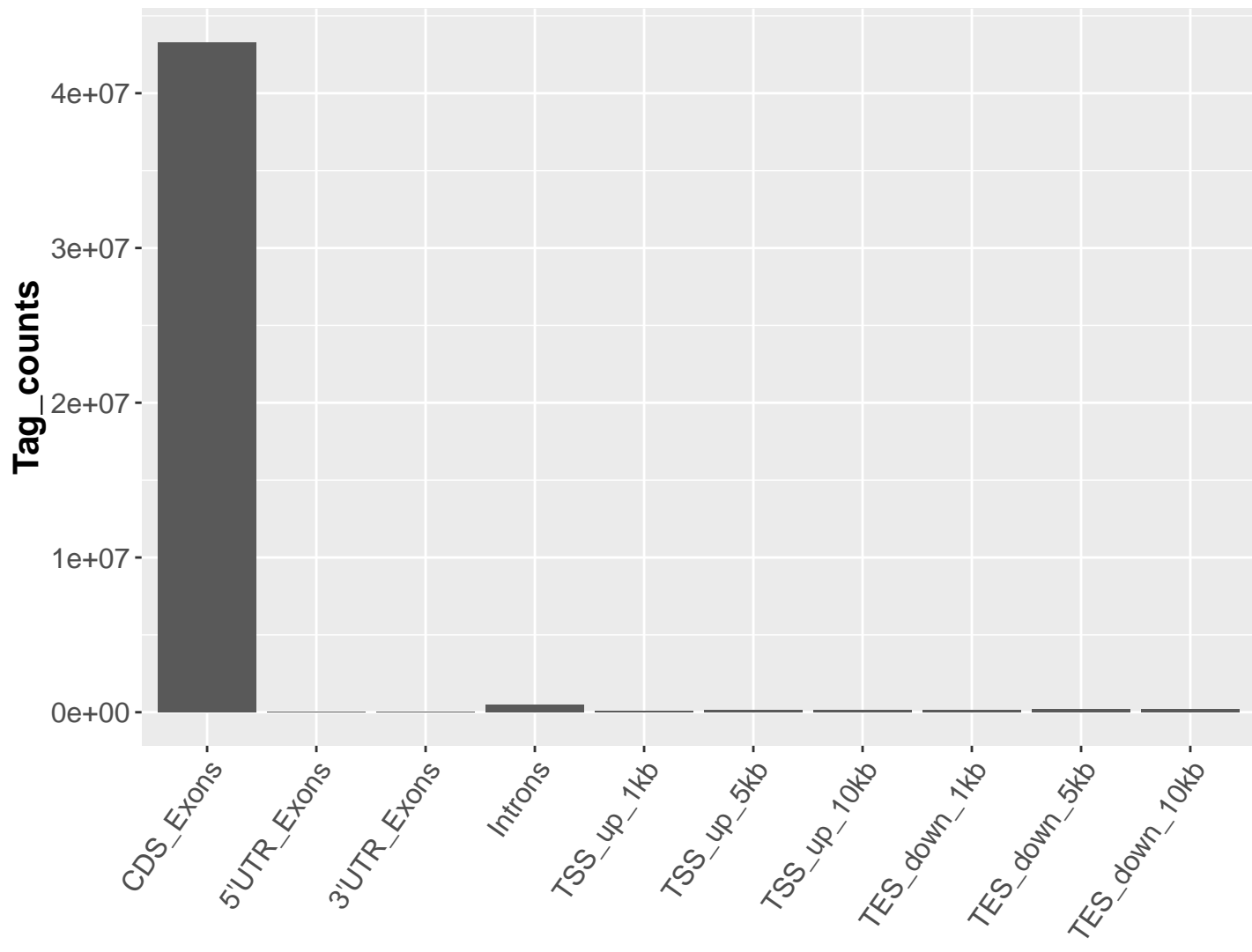

Supplement: Supplementary file 1 [file Data_Sheet_1.ZIP › 04_MapQC/read_distribution/D1.read_distribution.pdf]

# Read Distribution

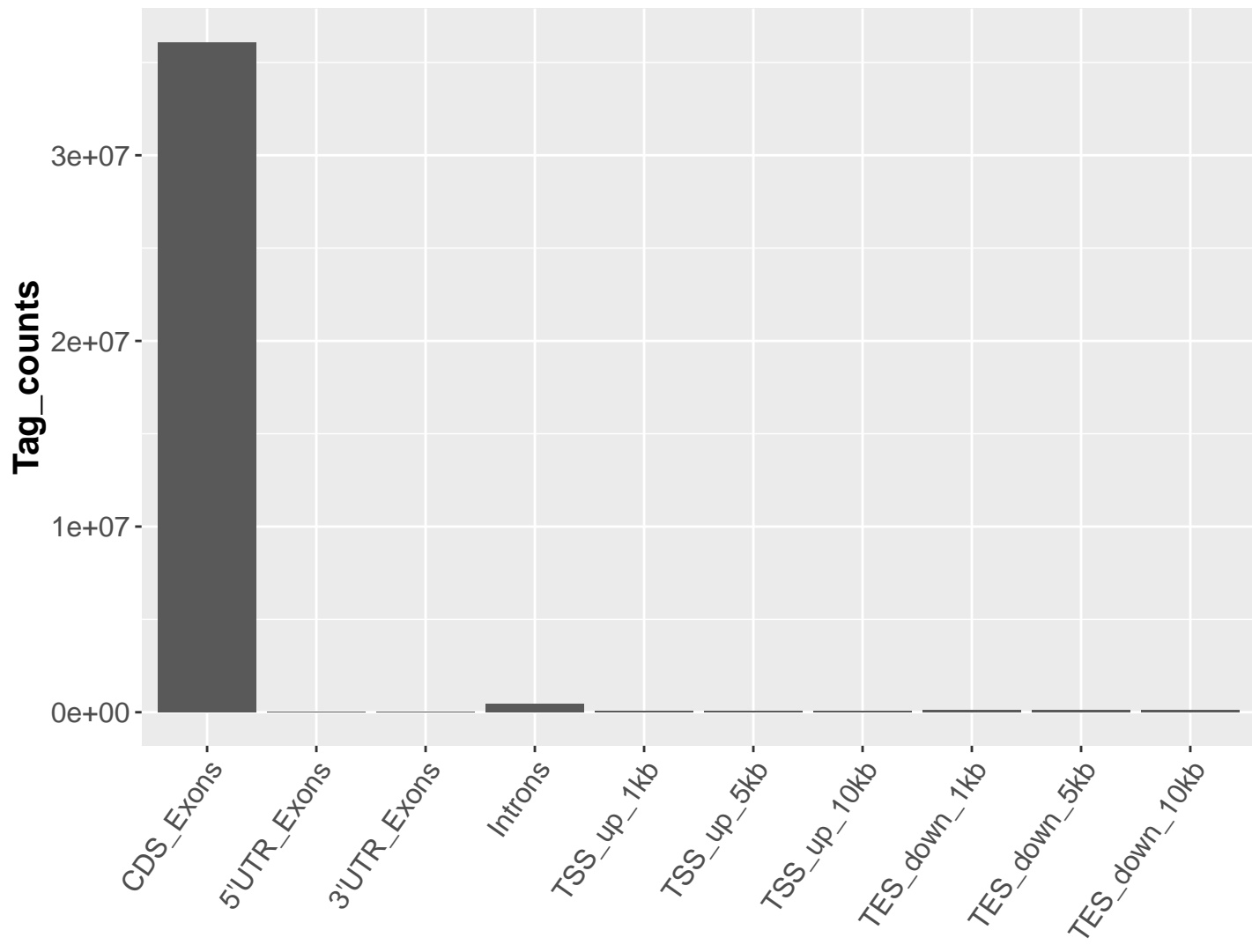

Supplement: Supplementary file 1 [file Data_Sheet_1.ZIP › 04_MapQC/read_distribution/D2.read_distribution.pdf]

# Read Distribution

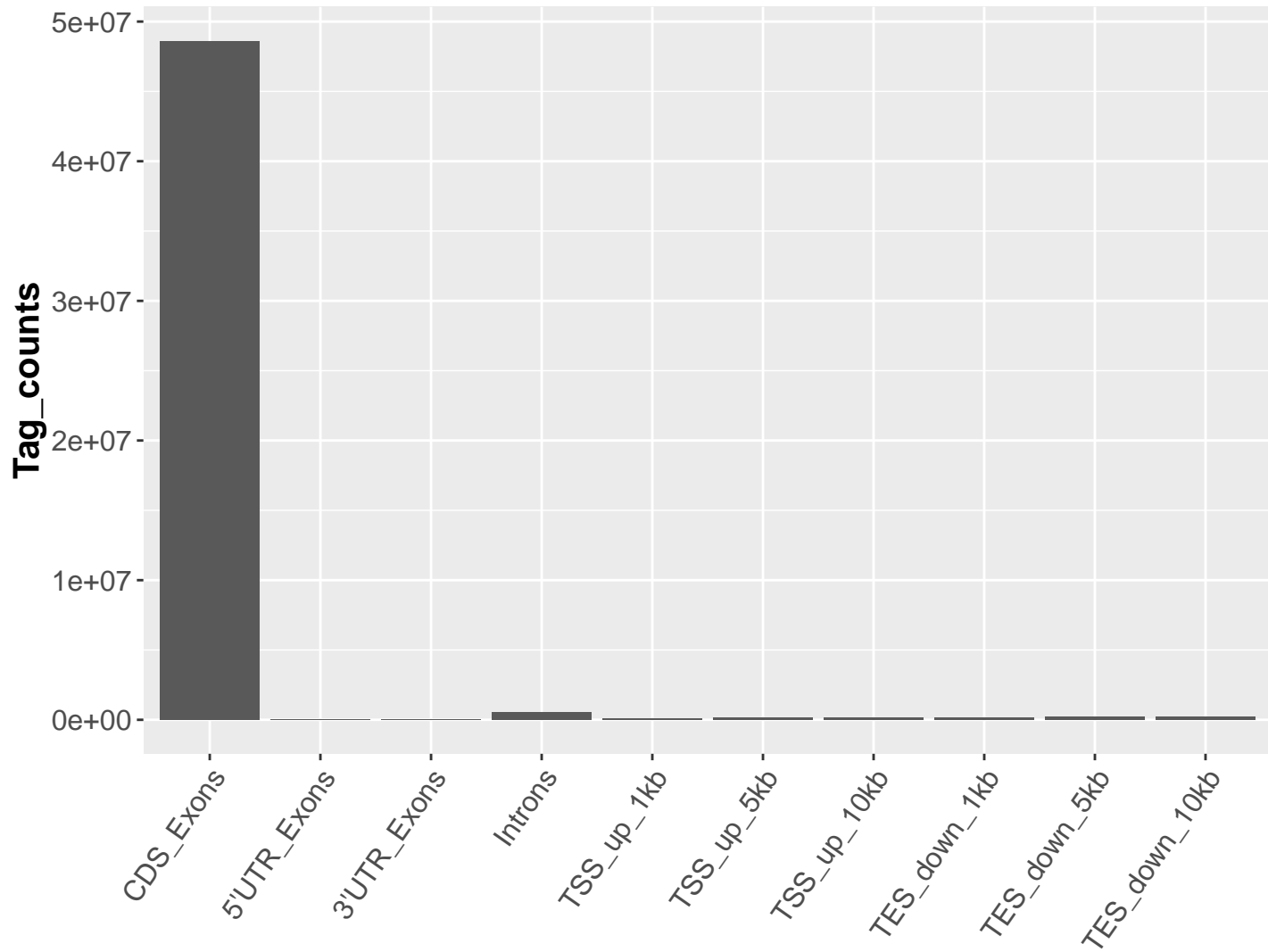

Supplement: Supplementary file 1 [file Data_Sheet_1.ZIP › 04_MapQC/read_distribution/D3.read_distribution.pdf]

**Q1**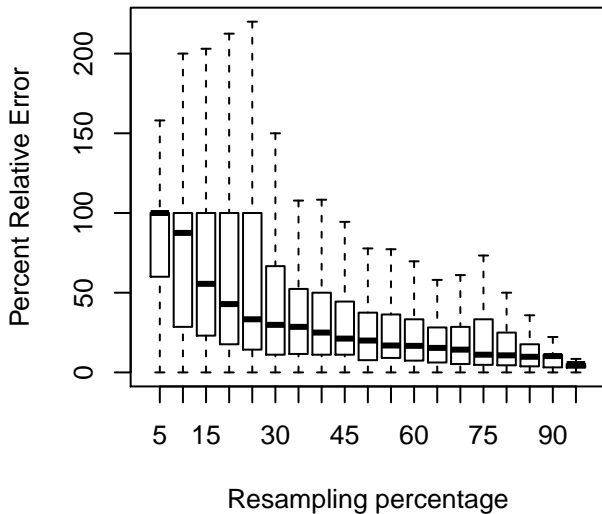**Q2**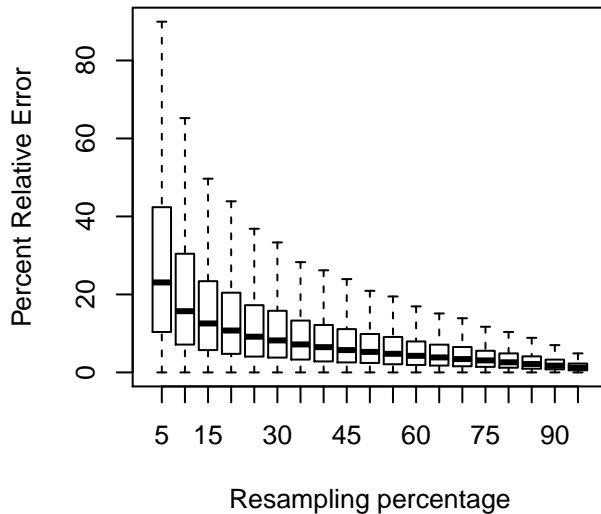**Q3**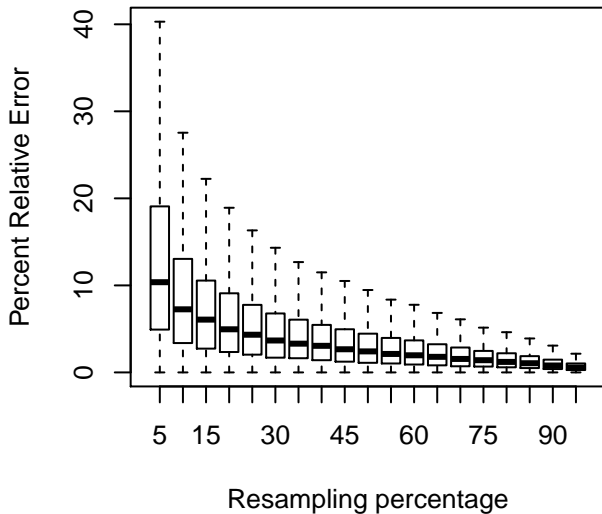**Q4**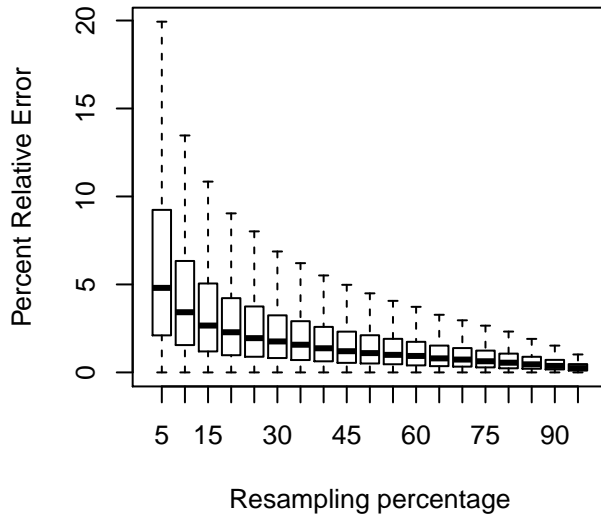

Supplement: Supplementary file 1 [file Data_Sheet_1.ZIP › 04_MapQC/RPKM_saturation/A1.saturation.pdf]

**Q1**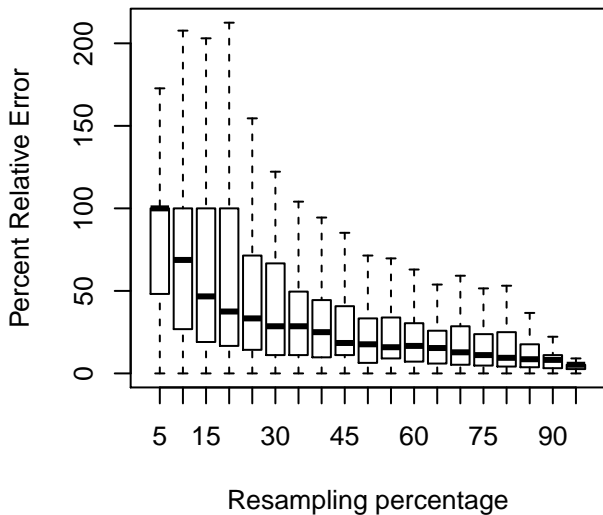**Q2**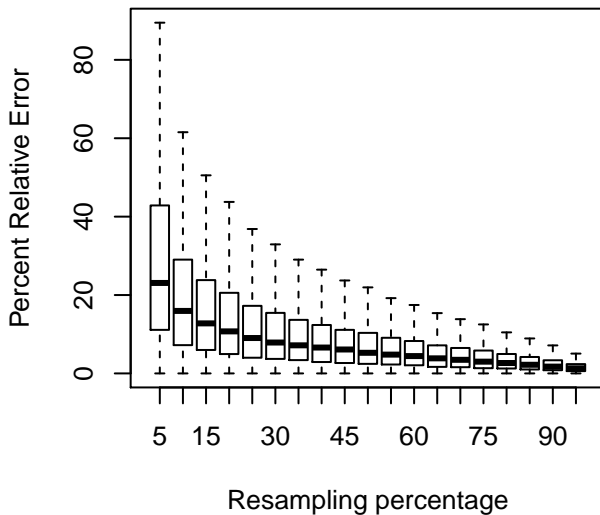**Q3**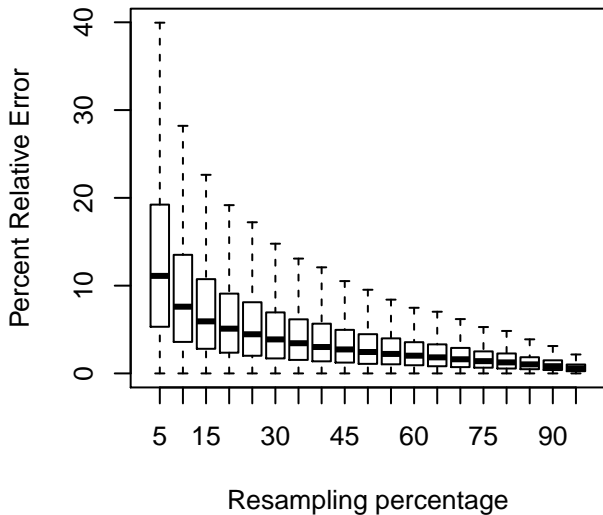**Q4**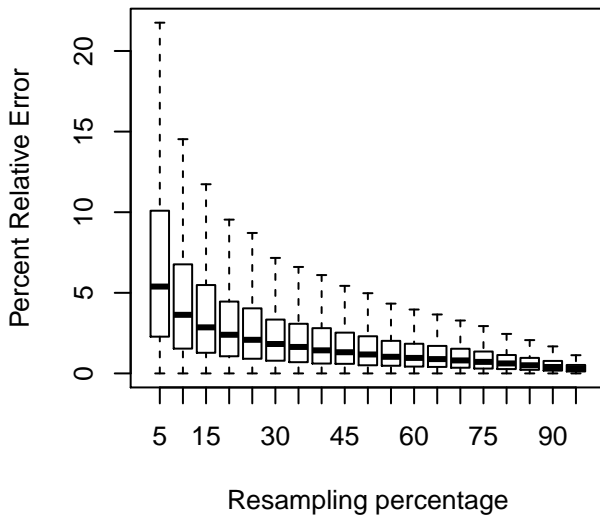

Supplement: Supplementary file 1 [file Data_Sheet_1.ZIP › 04_MapQC/RPKM_saturation/A2.saturation.pdf]

**Q1**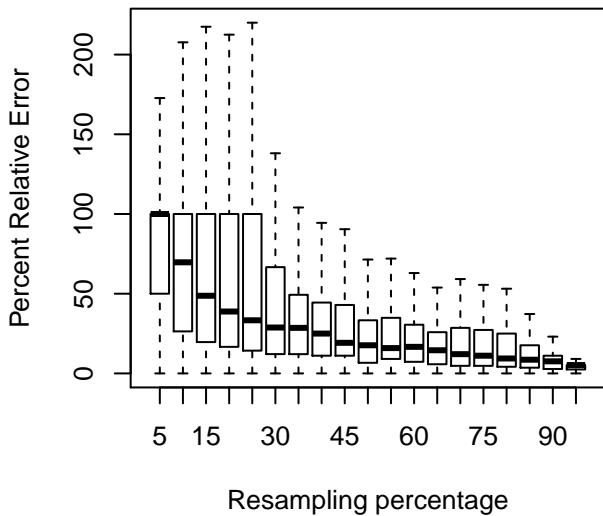**Q2**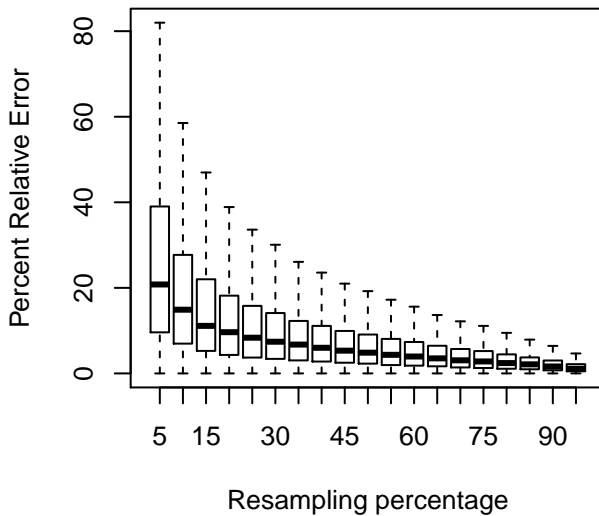**Q3**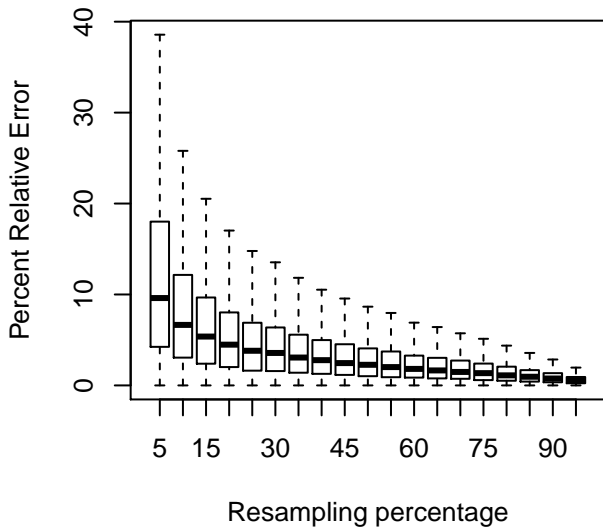**Q4**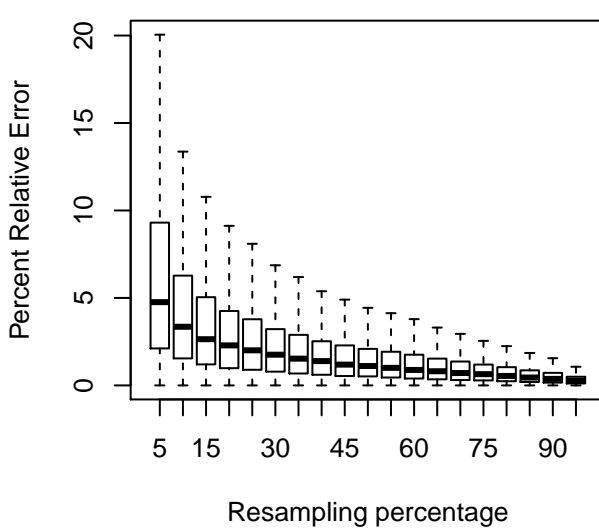

Supplement: Supplementary file 1 [file Data_Sheet_1.ZIP › 04_MapQC/RPKM_saturation/A3.saturation.pdf]

**Q1**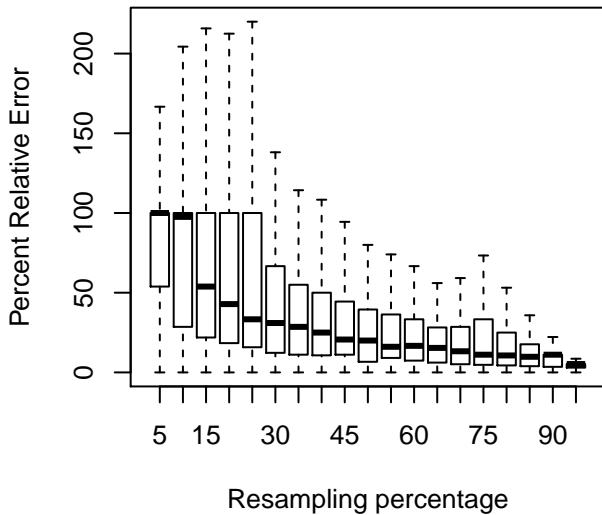**Q2**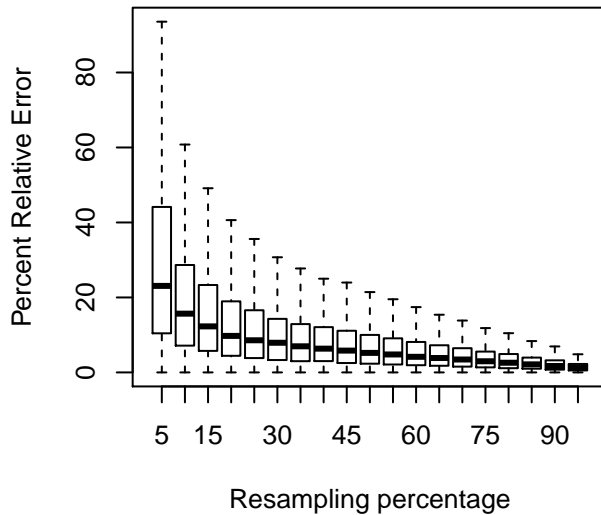**Q3**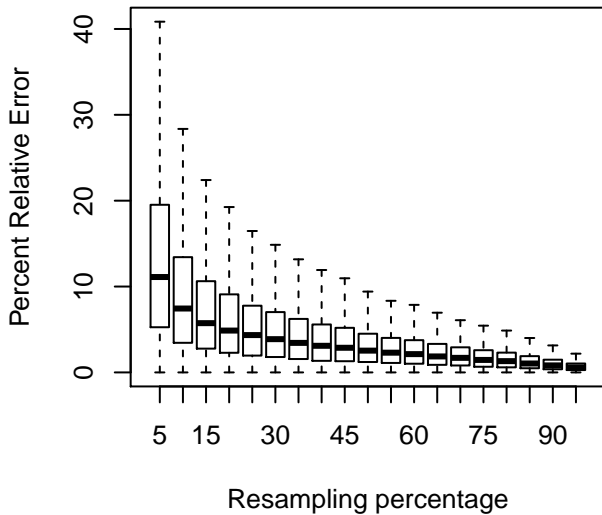**Q4**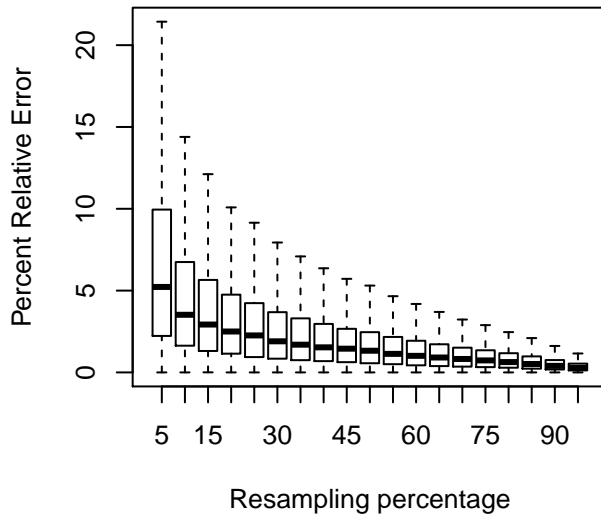

Supplement: Supplementary file 1 [file Data_Sheet_1.ZIP › 04_MapQC/RPKM_saturation/B1.saturation.pdf]

**Q1**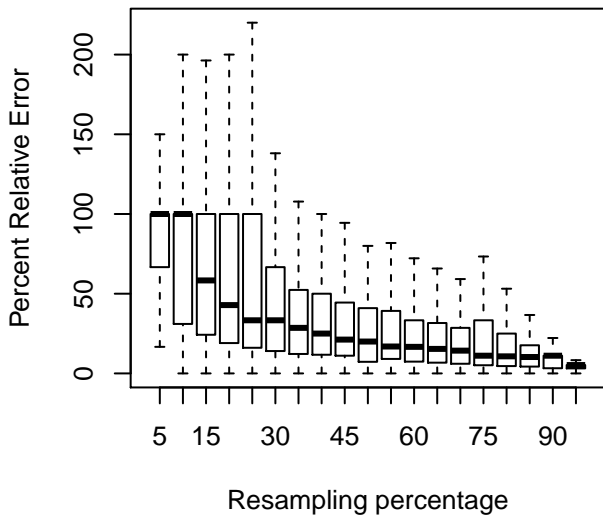**Q2**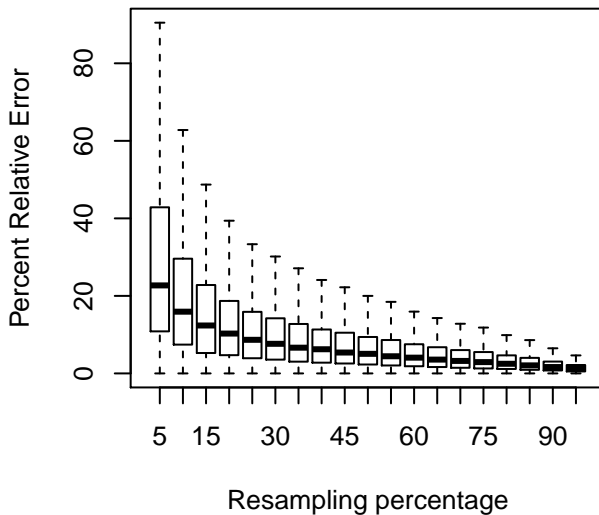**Q3**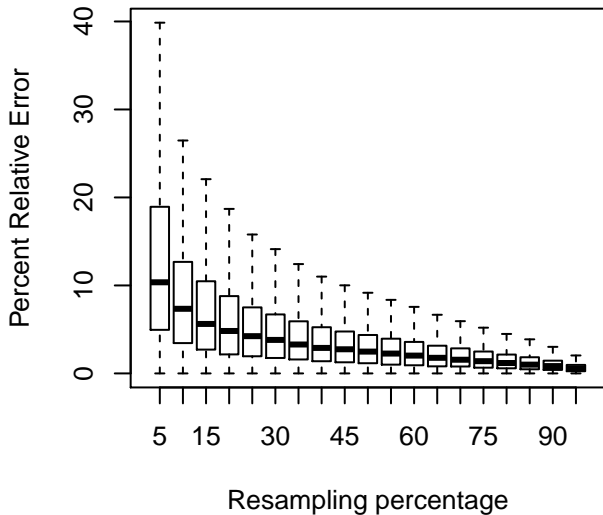**Q4**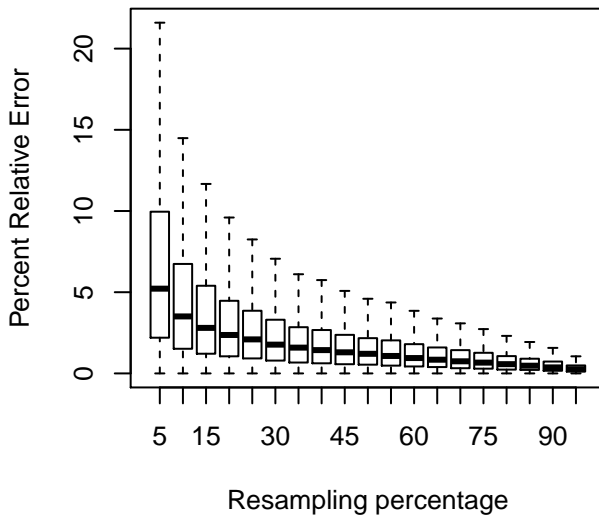

Supplement: Supplementary file 1 [file Data_Sheet_1.ZIP › 04_MapQC/RPKM_saturation/B2.saturation.pdf]

**Q1**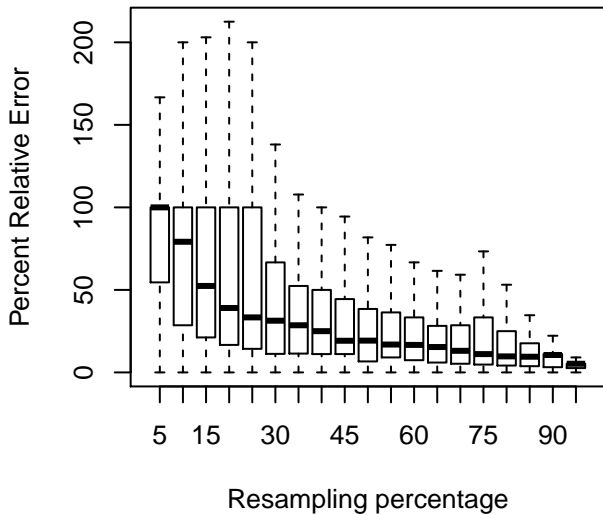**Q2**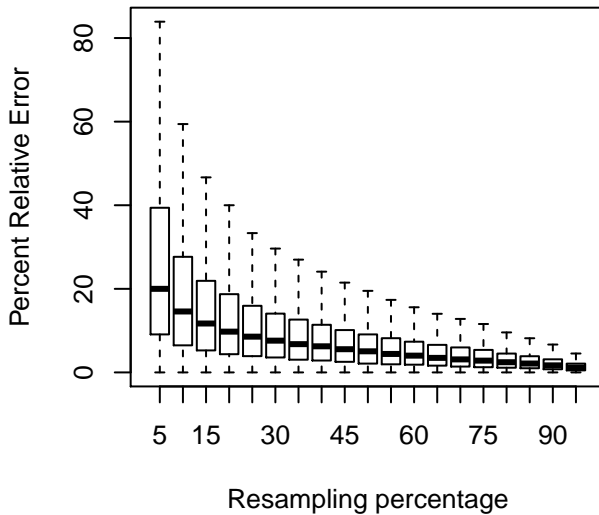**Q3**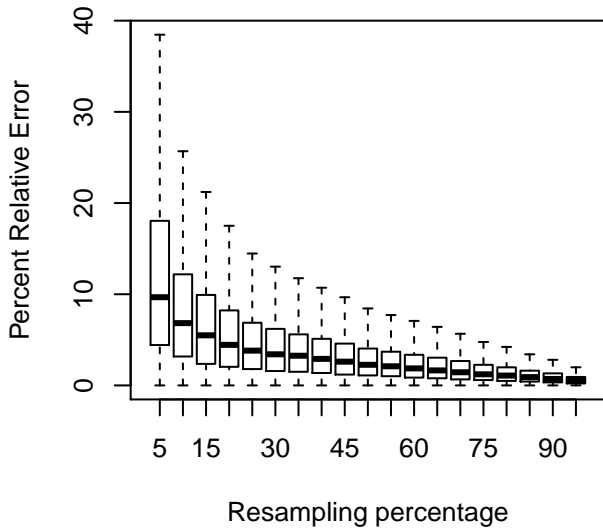**Q4**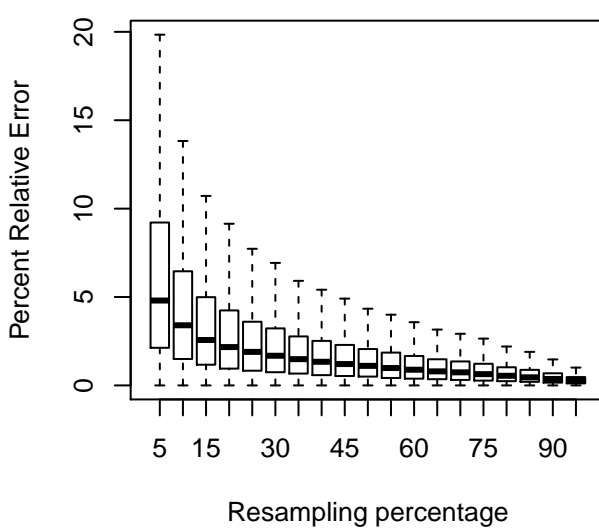

Supplement: Supplementary file 1 [file Data_Sheet_1.ZIP › 04_MapQC/RPKM_saturation/B3.saturation.pdf]

**Q1**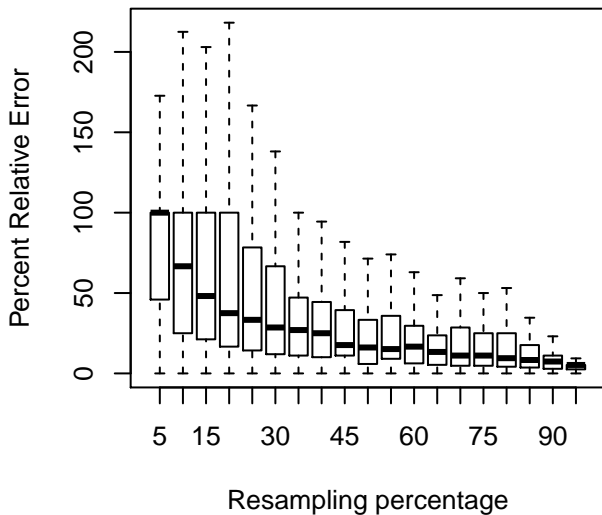**Q2**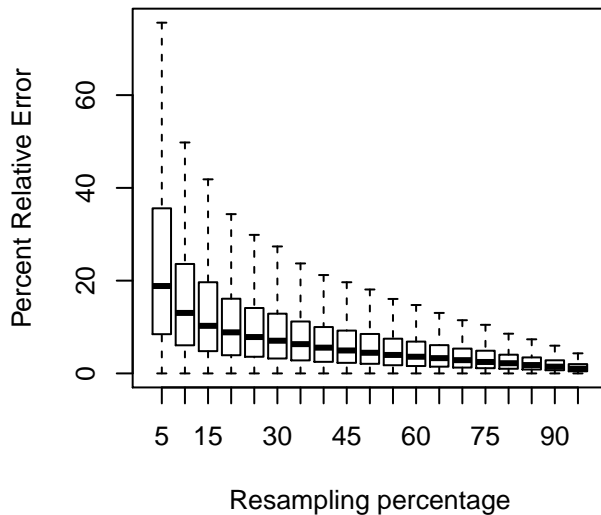**Q3**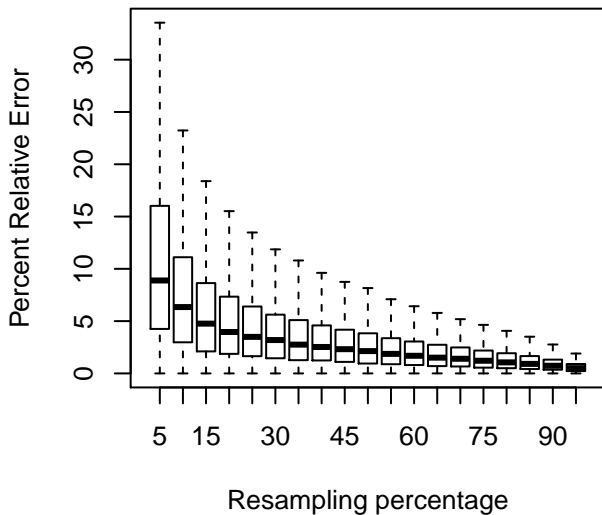**Q4**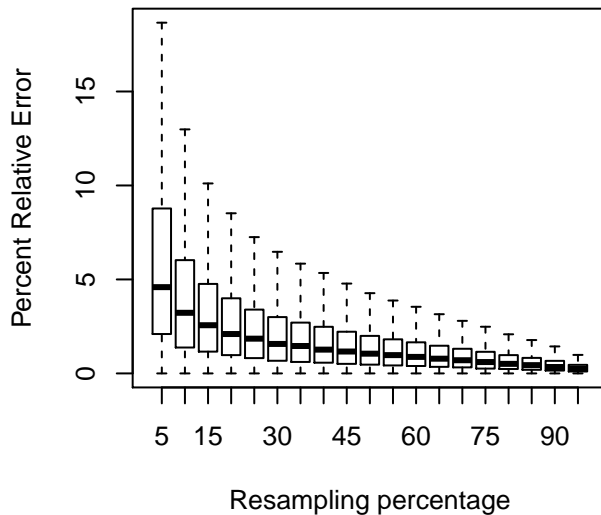

Supplement: Supplementary file 1 [file Data_Sheet_1.ZIP › 04_MapQC/RPKM_saturation/C1.saturation.pdf]

**Q1**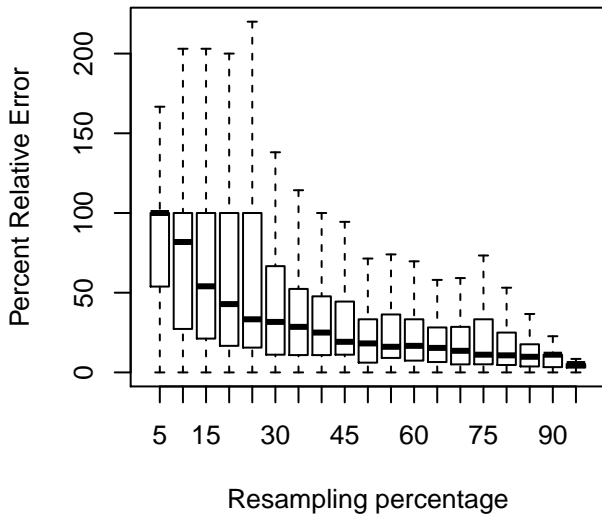**Q2**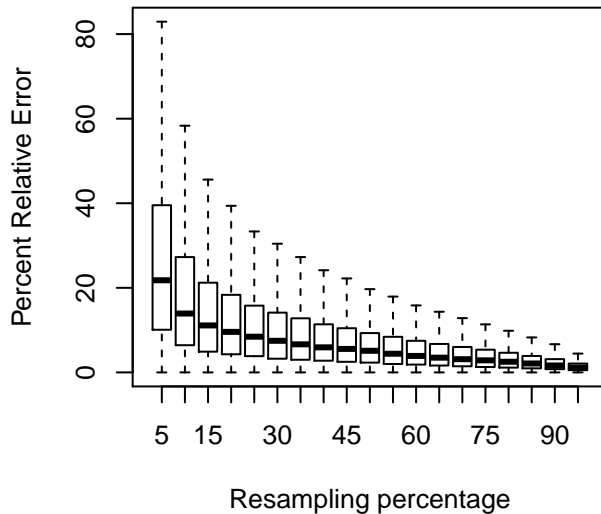**Q3**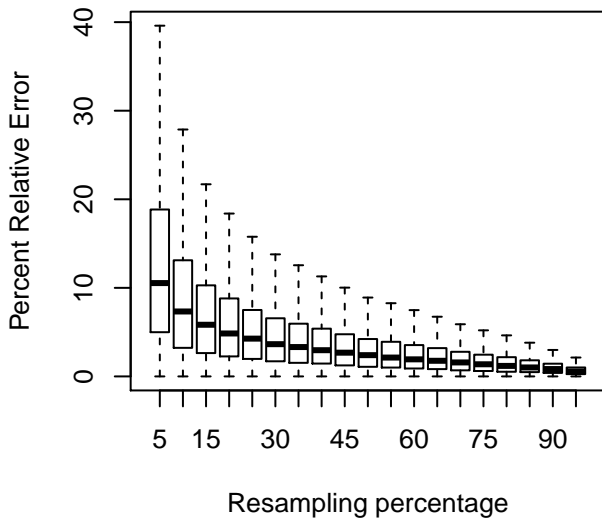**Q4**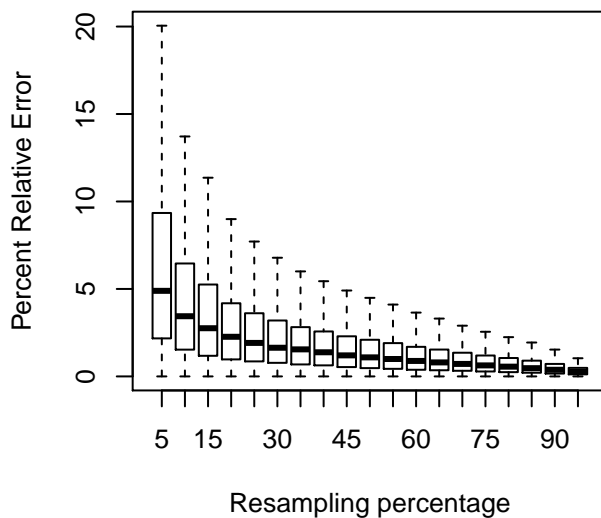

Supplement: Supplementary file 1 [file Data_Sheet_1.ZIP › 04_MapQC/RPKM_saturation/C2.saturation.pdf]

**Q1**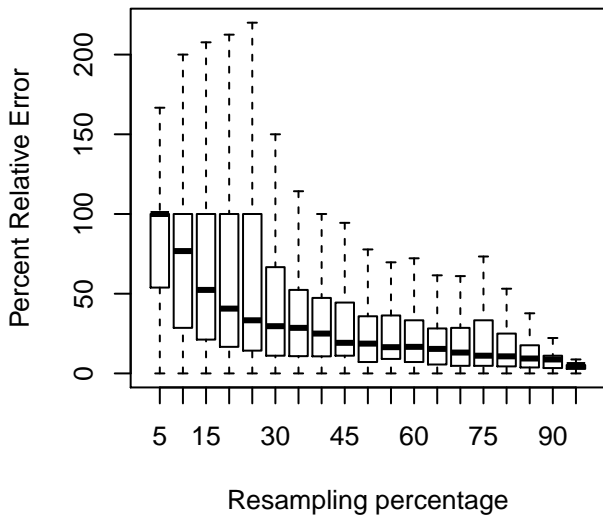**Q2**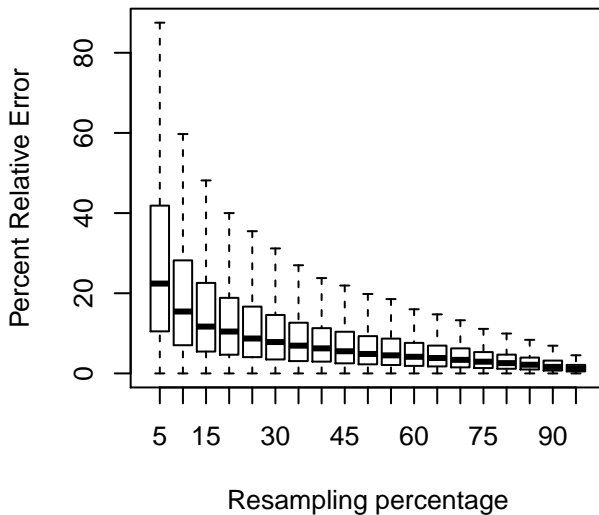**Q3**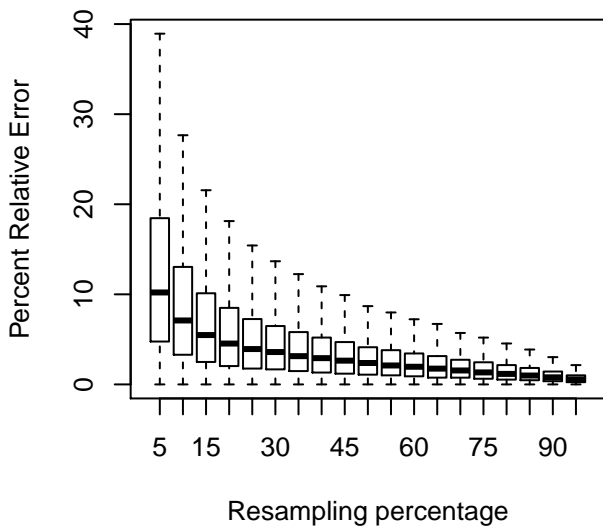**Q4**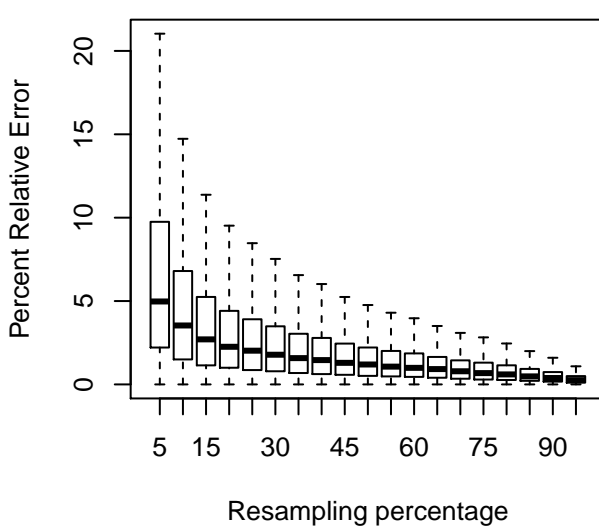

Supplement: Supplementary file 1 [file Data_Sheet_1.ZIP › 04_MapQC/RPKM_saturation/C3.saturation.pdf]

**Q1**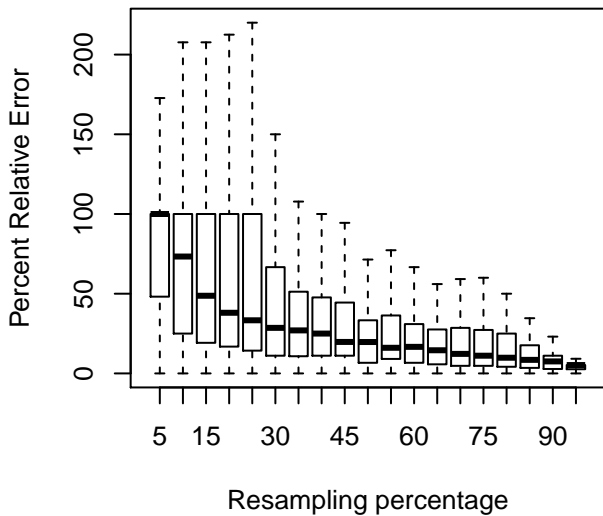**Q2**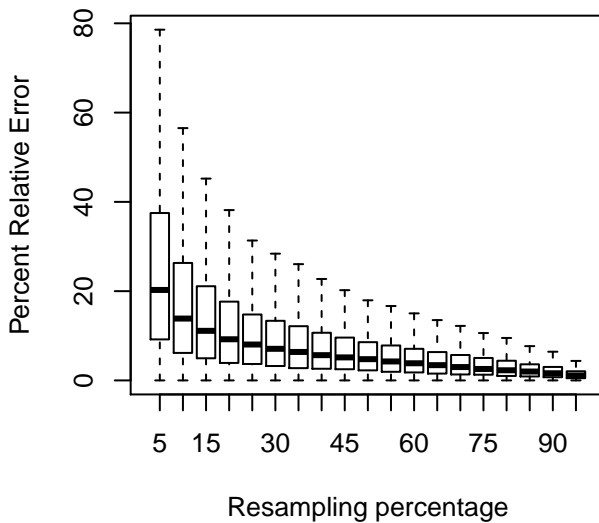**Q3**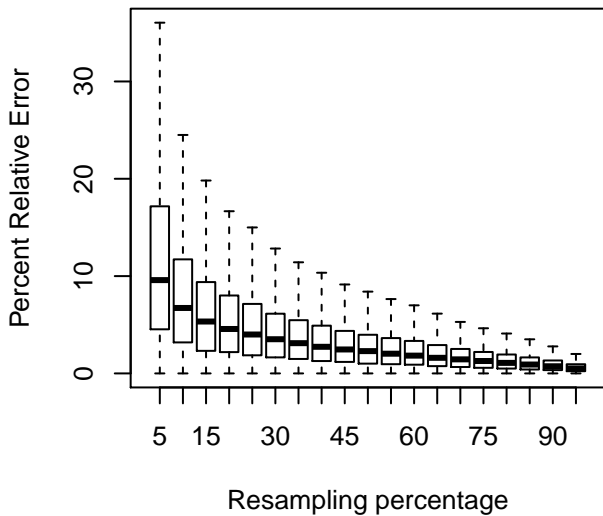**Q4**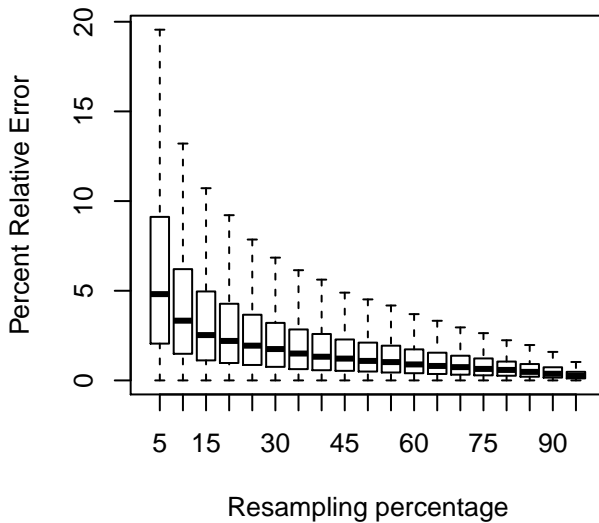

Supplement: Supplementary file 1 [file Data_Sheet_1.ZIP › 04_MapQC/RPKM_saturation/D1.saturation.pdf]

**Q1**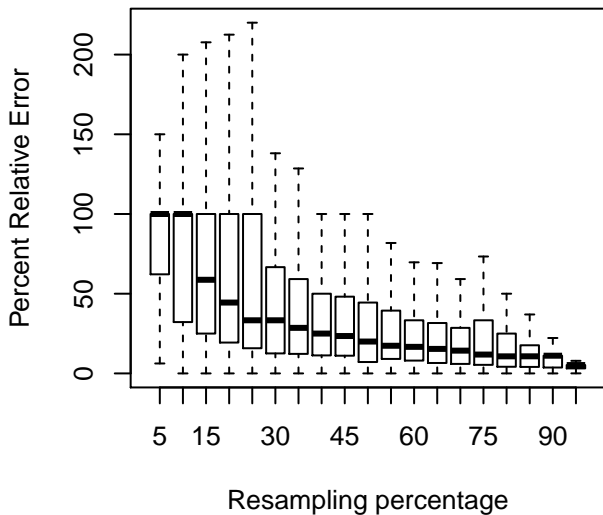**Q2**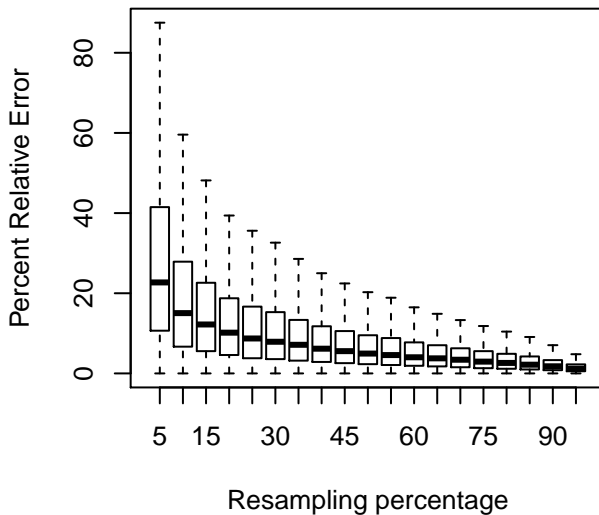**Q3**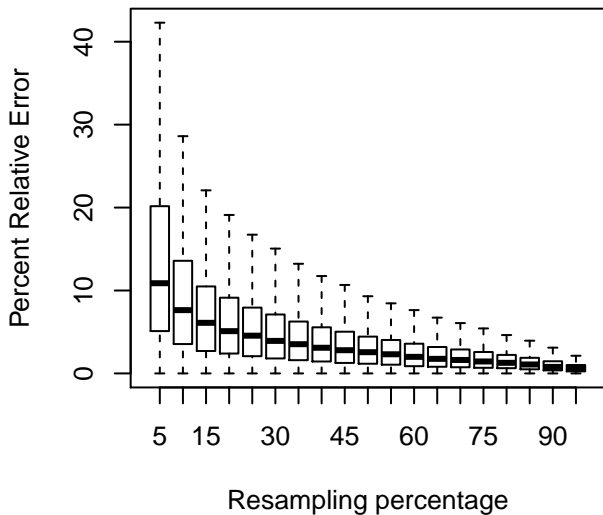**Q4**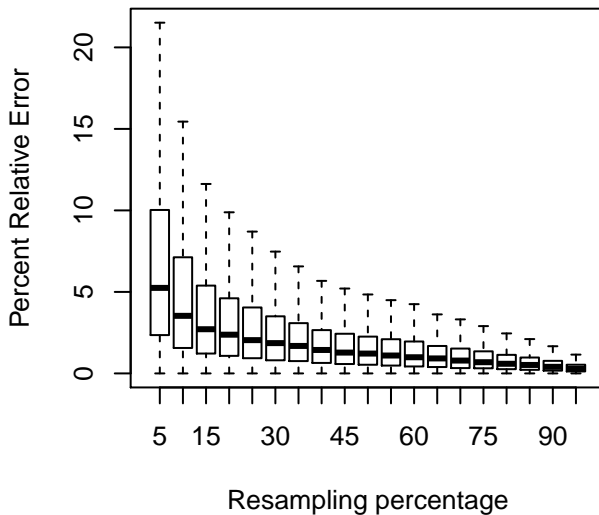

Supplement: Supplementary file 1 [file Data_Sheet_1.ZIP › 04_MapQC/RPKM_saturation/D2.saturation.pdf]

**Q1**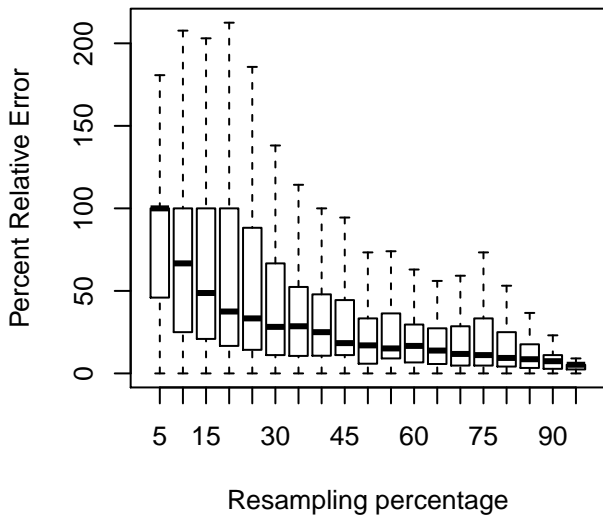**Q2**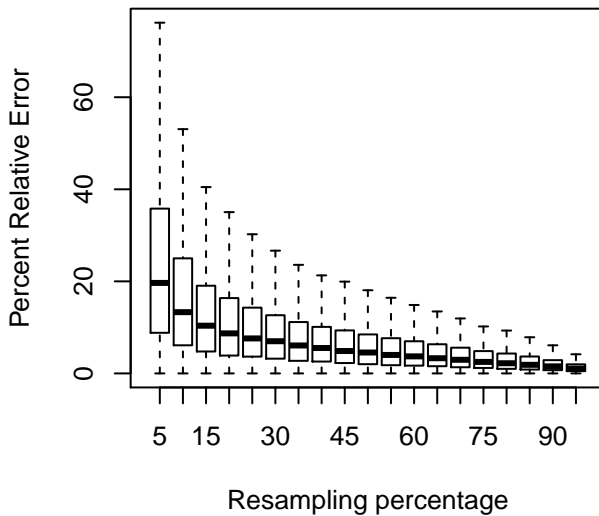**Q3**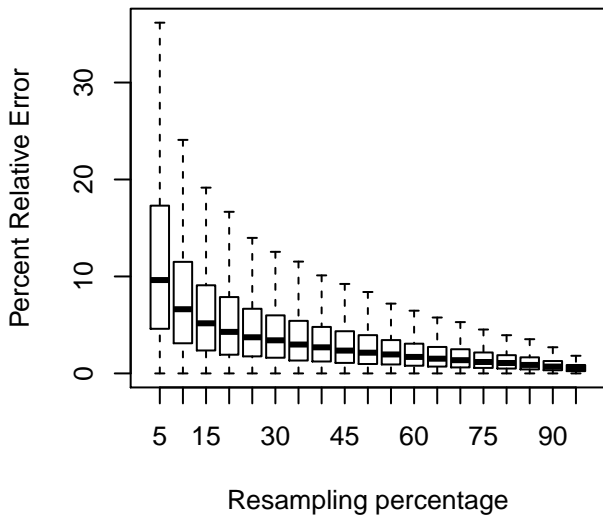**Q4**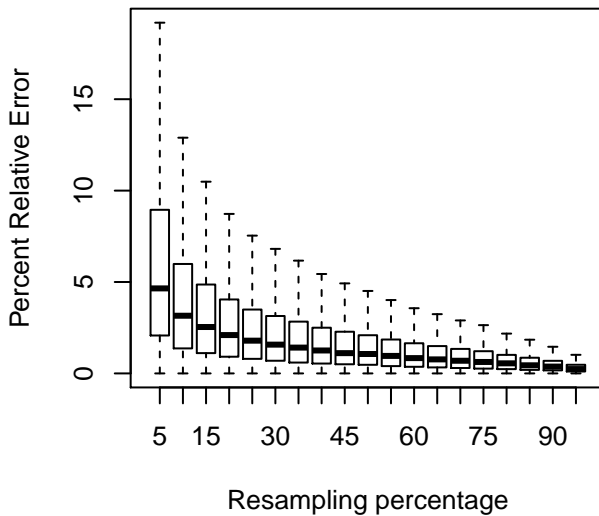

Supplement: Supplementary file 1 [file Data_Sheet_1.ZIP › 04_MapQC/RPKM_saturation/D3.saturation.pdf]

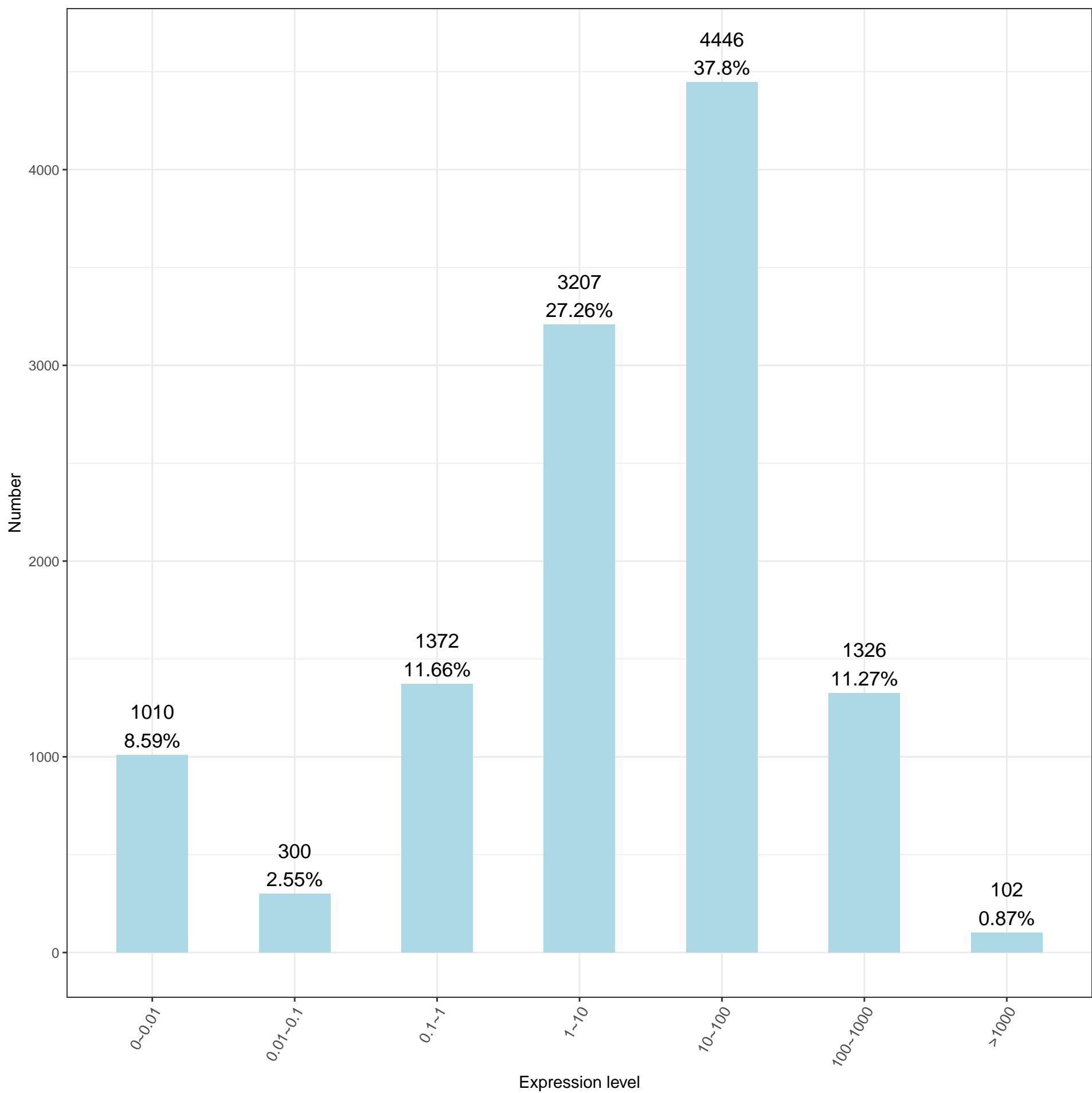

Supplement: Supplementary file 1 [file Data_Sheet_1.ZIP › mRNA/1_Expression/A1.fpkm_distribution.pdf]

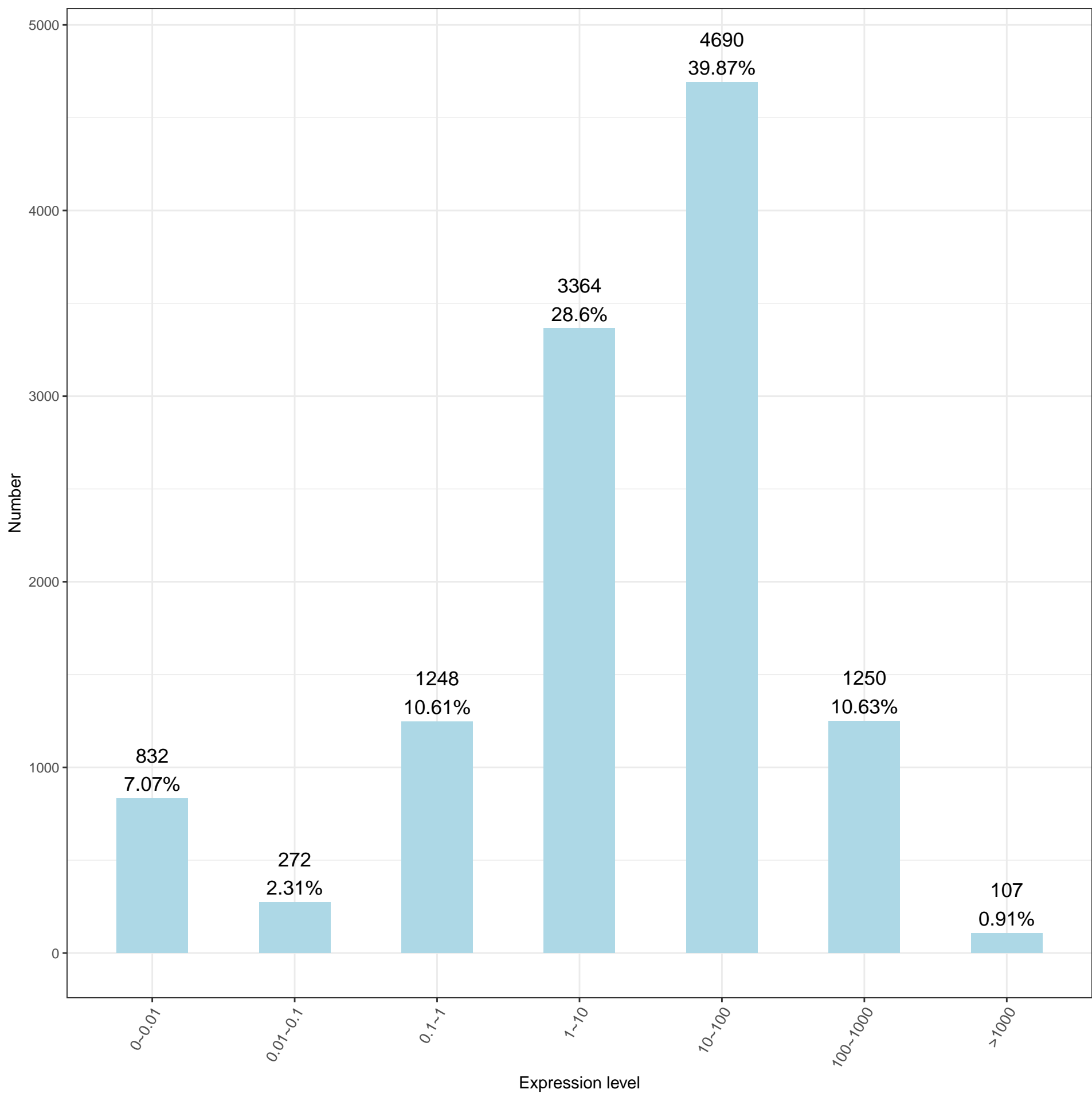

Supplement: Supplementary file 1 [file Data_Sheet_1.ZIP › mRNA/1_Expression/A2.fpkm_distribution.pdf]

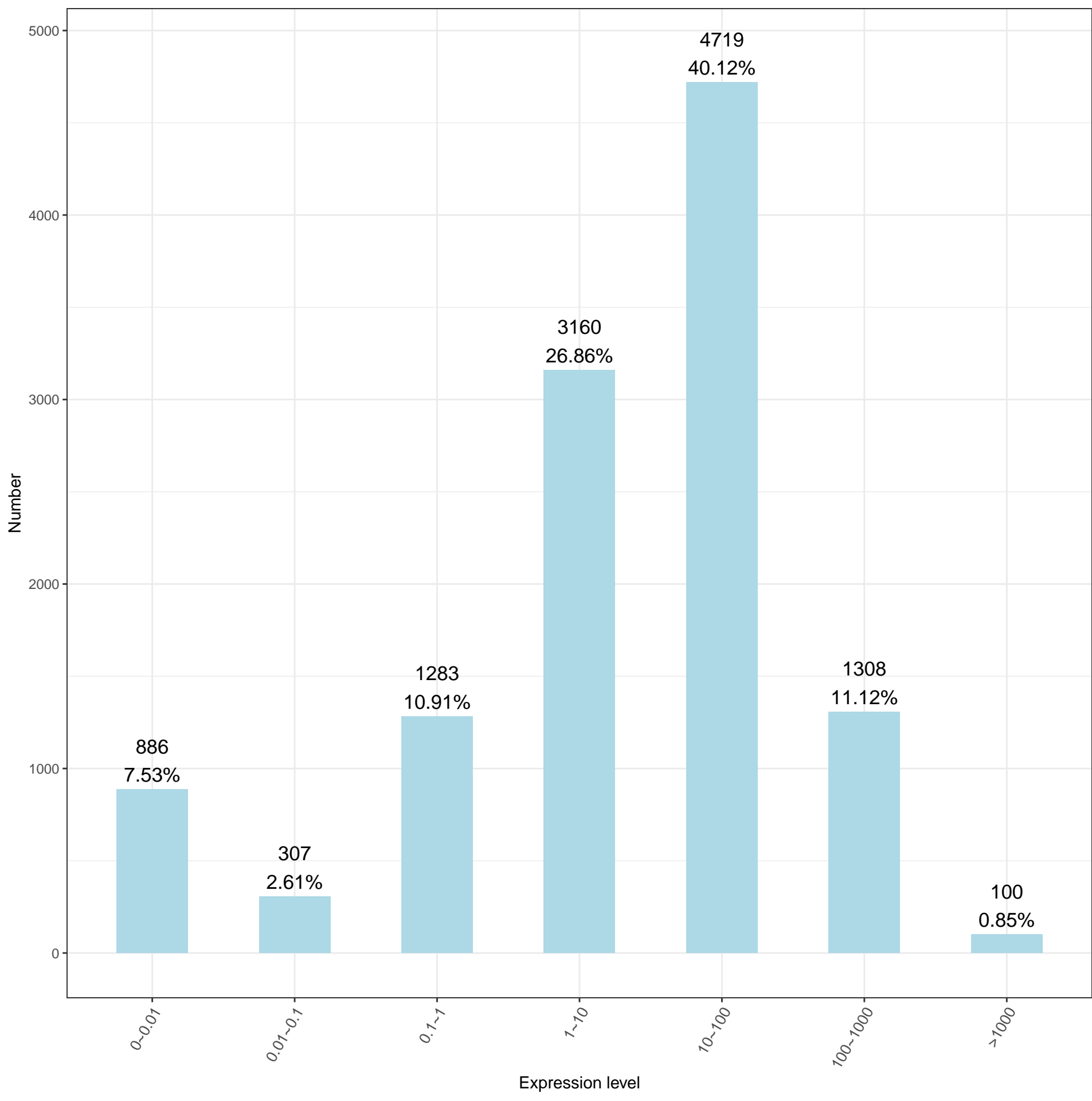

Supplement: Supplementary file 1 [file Data_Sheet_1.ZIP › mRNA/1_Expression/A3.fpkm_distribution.pdf]

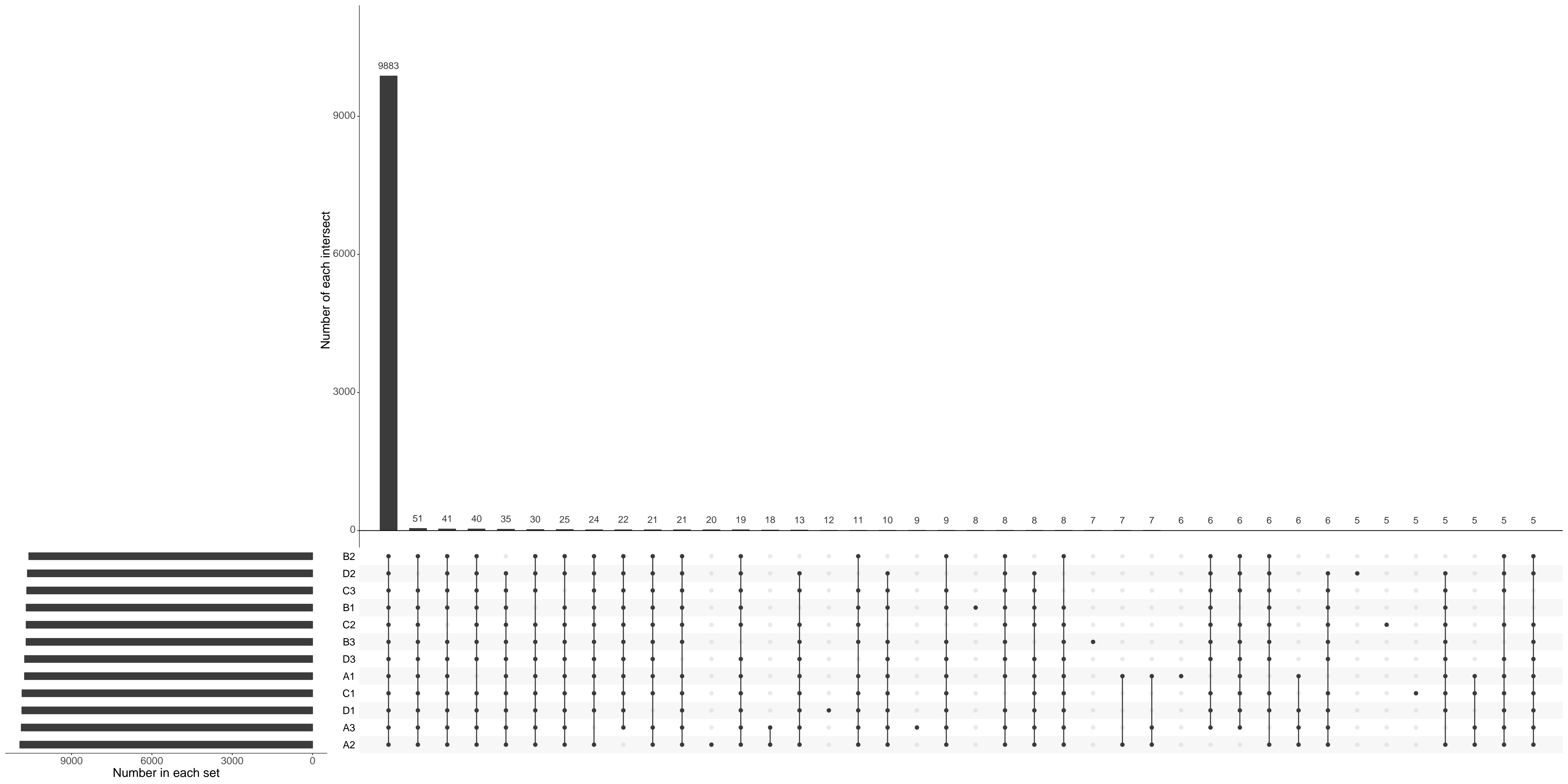

Supplement: Supplementary file 1 [file Data_Sheet_1.ZIP › mRNA/1_Expression/allsample.UpSet.pdf]

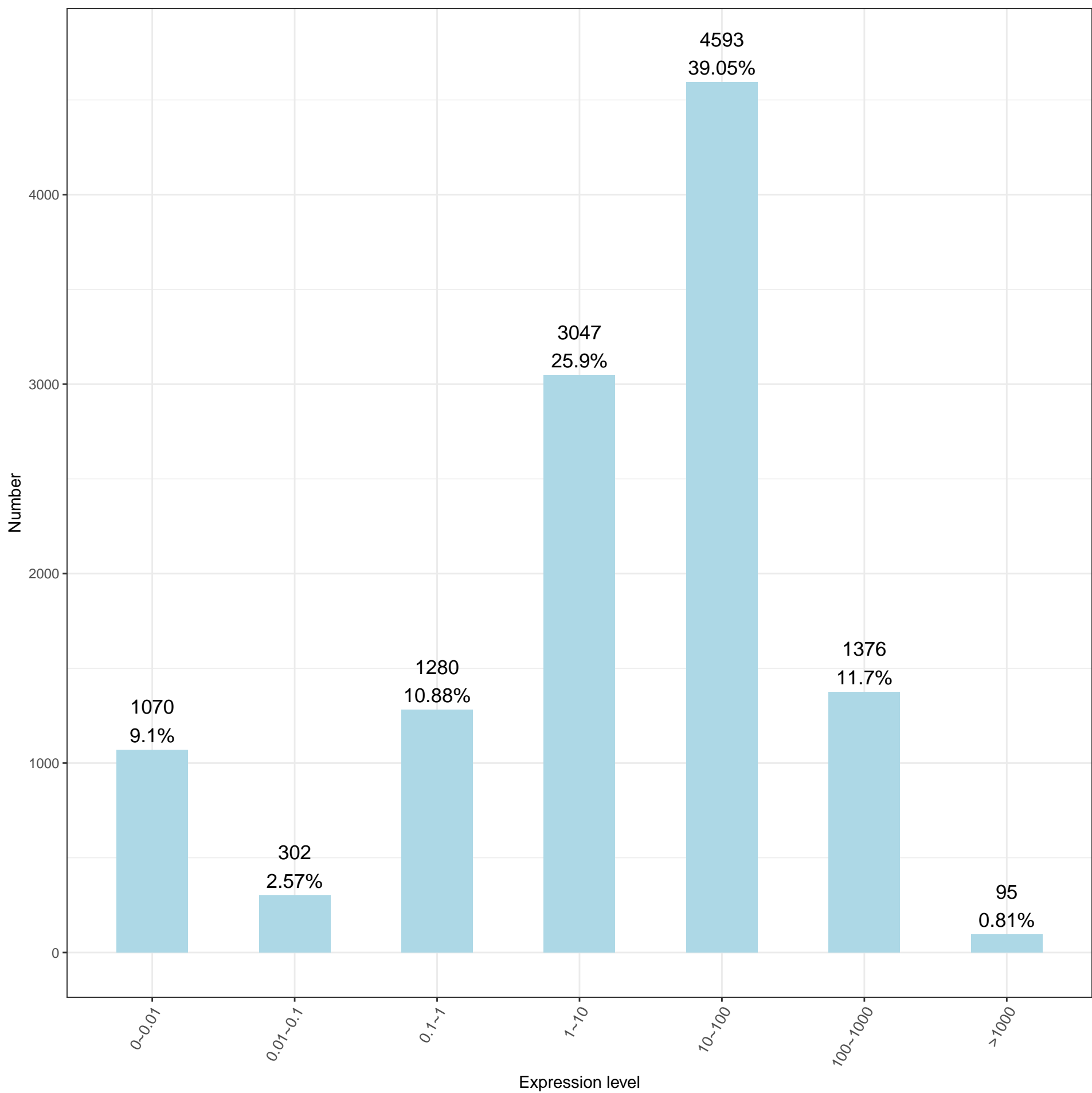

Supplement: Supplementary file 1 [file Data_Sheet_1.ZIP › mRNA/1_Expression/B1.fpkm_distribution.pdf]

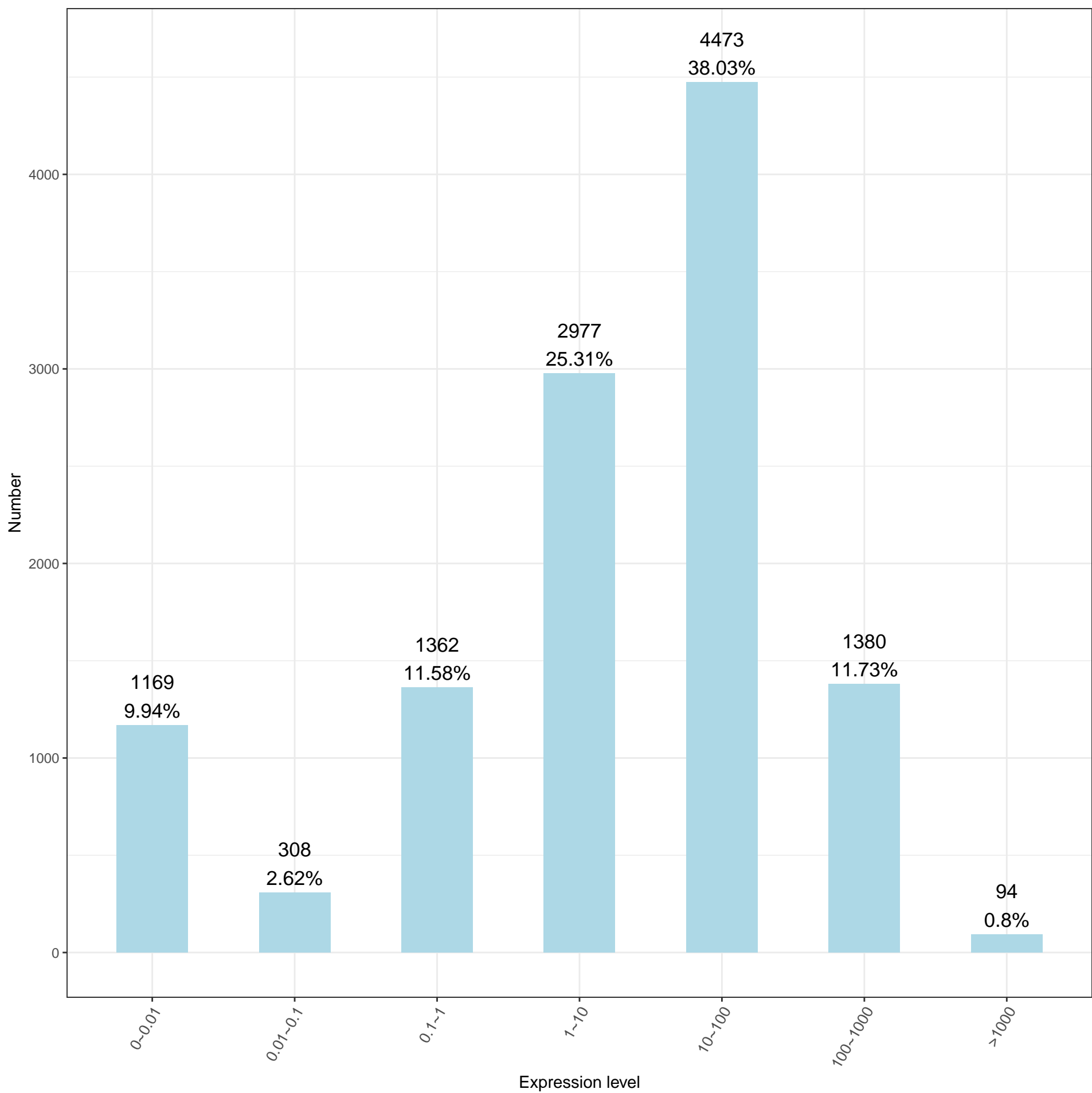

Supplement: Supplementary file 1 [file Data_Sheet_1.ZIP › mRNA/1_Expression/B2.fpkm_distribution.pdf]

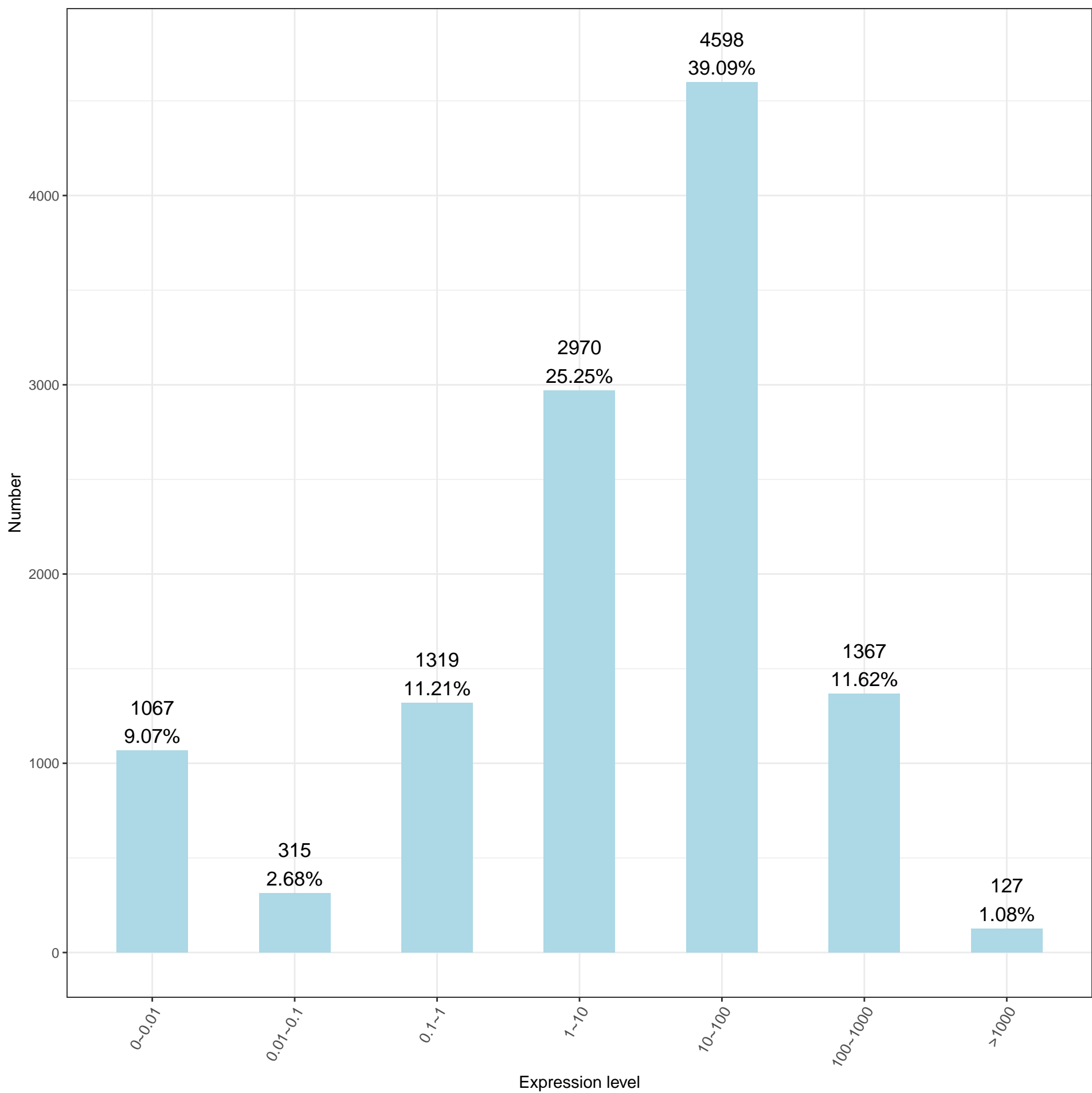

Supplement: Supplementary file 1 [file Data_Sheet_1.ZIP › mRNA/1_Expression/B3.fpkm_distribution.pdf]

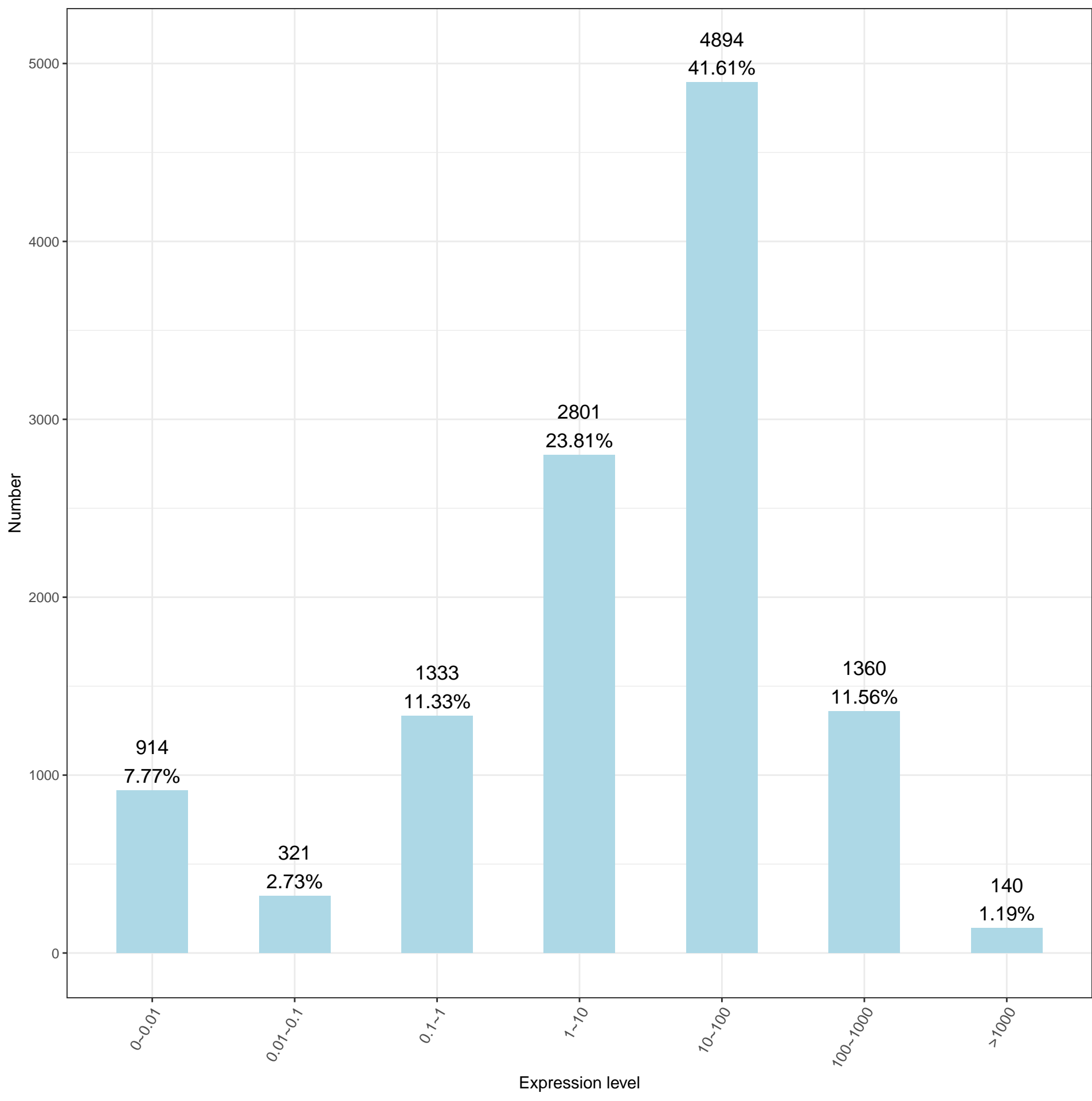

Supplement: Supplementary file 1 [file Data_Sheet_1.ZIP › mRNA/1_Expression/C1.fpkm_distribution.pdf]

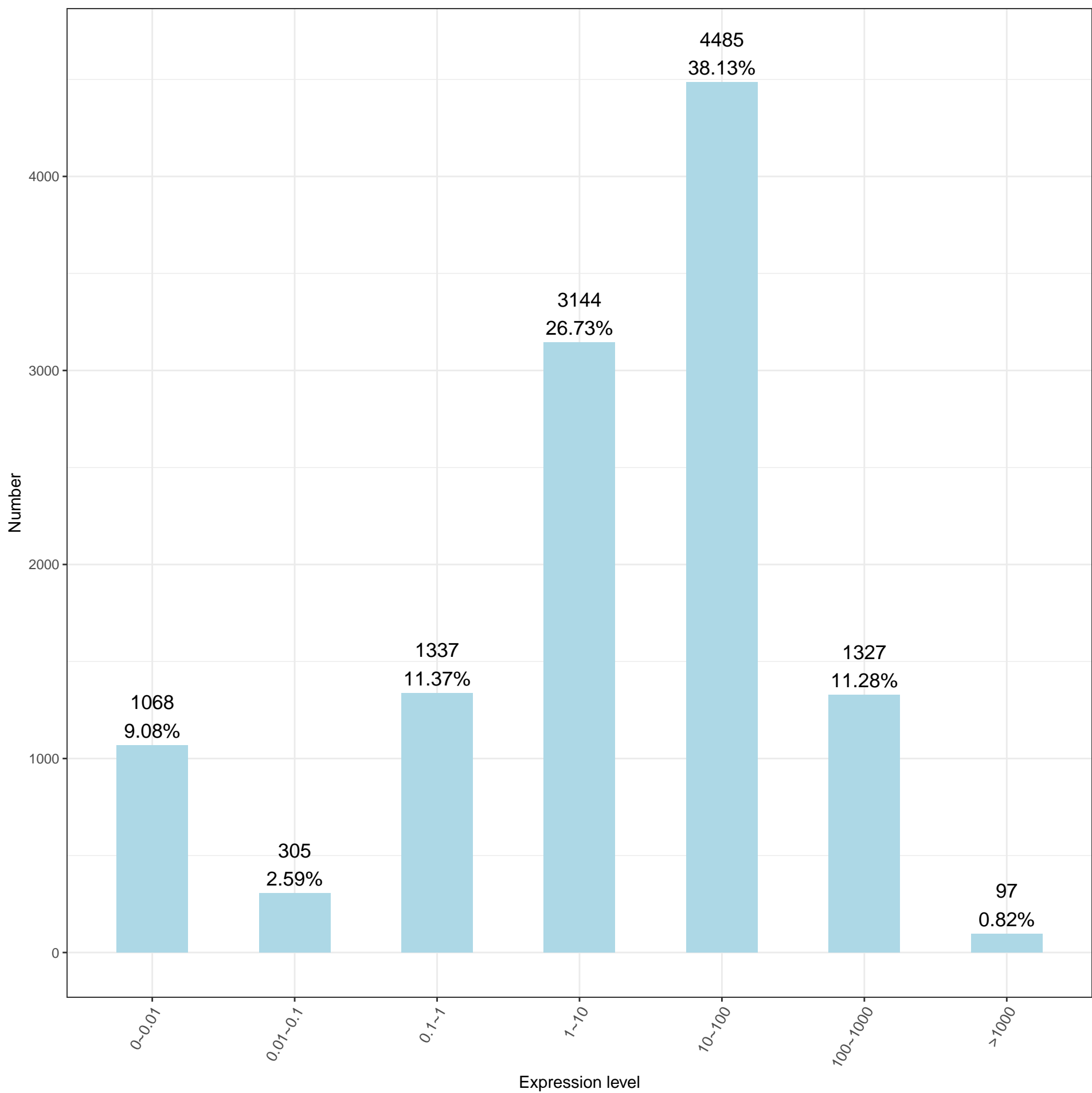

Supplement: Supplementary file 1 [file Data_Sheet_1.ZIP › mRNA/1_Expression/C2.fpkm_distribution.pdf]

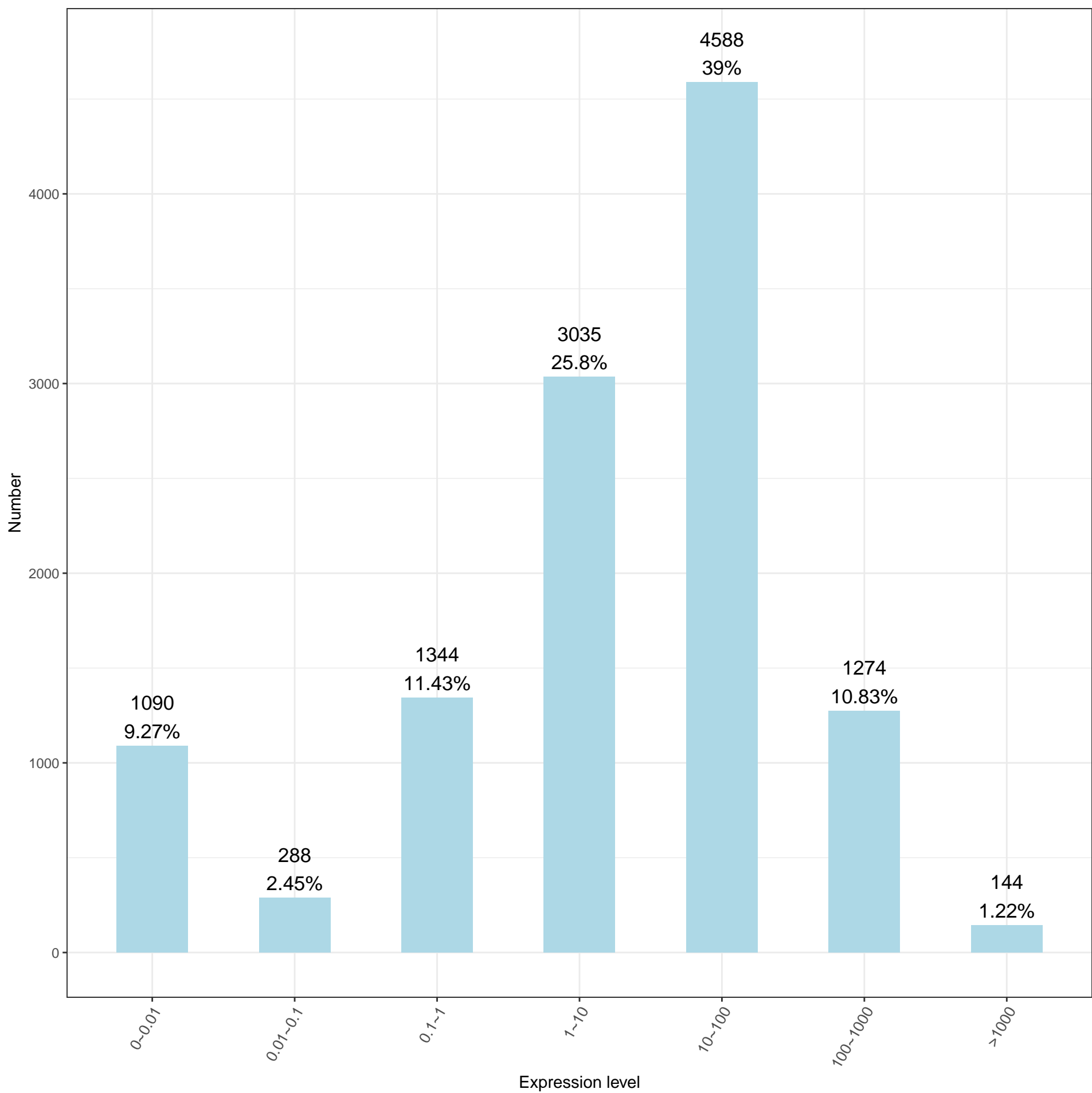

Supplement: Supplementary file 1 [file Data_Sheet_1.ZIP › mRNA/1_Expression/C3.fpkm_distribution.pdf]

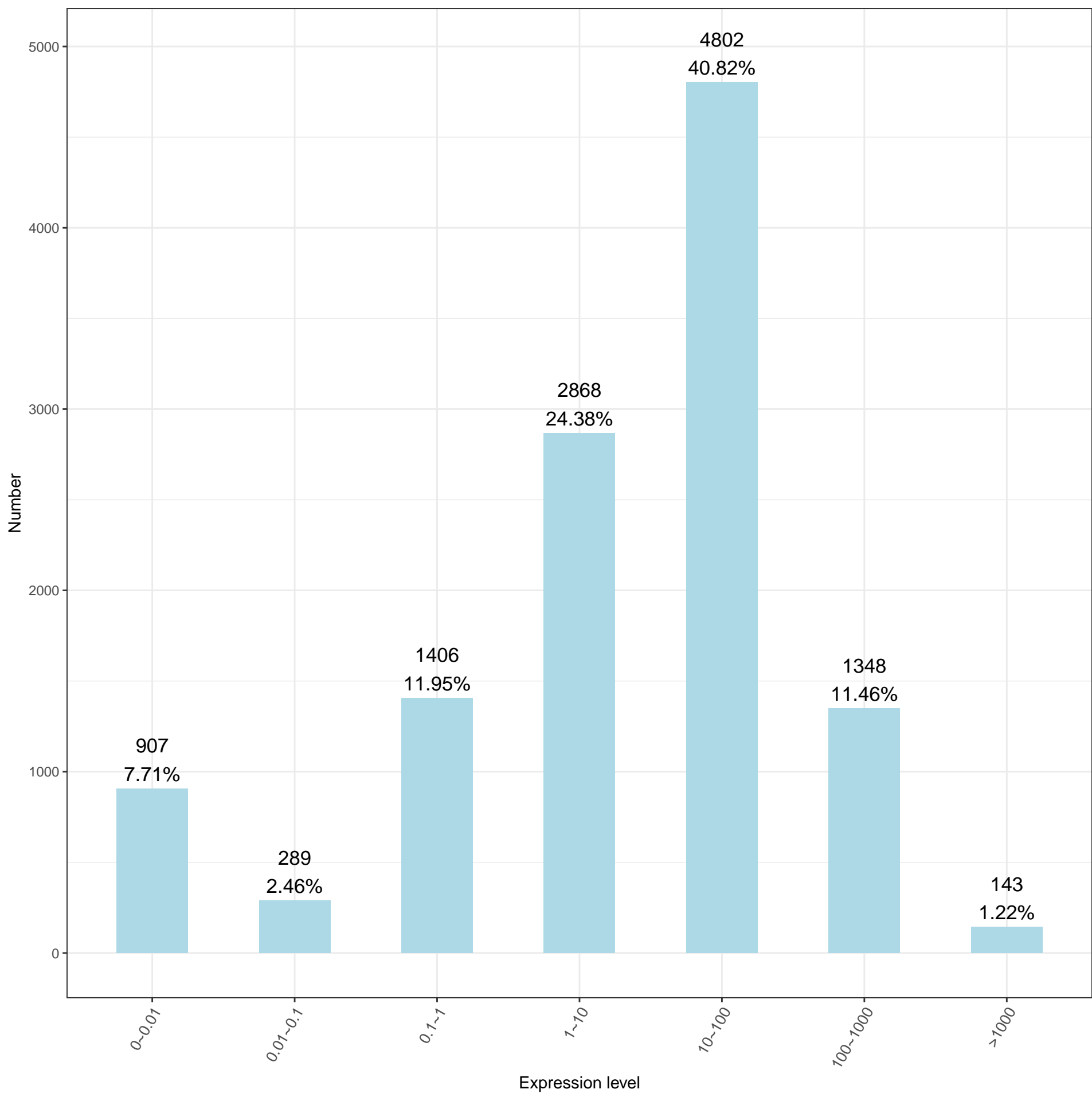

Supplement: Supplementary file 1 [file Data_Sheet_1.ZIP › mRNA/1_Expression/D1.fpkm_distribution.pdf]

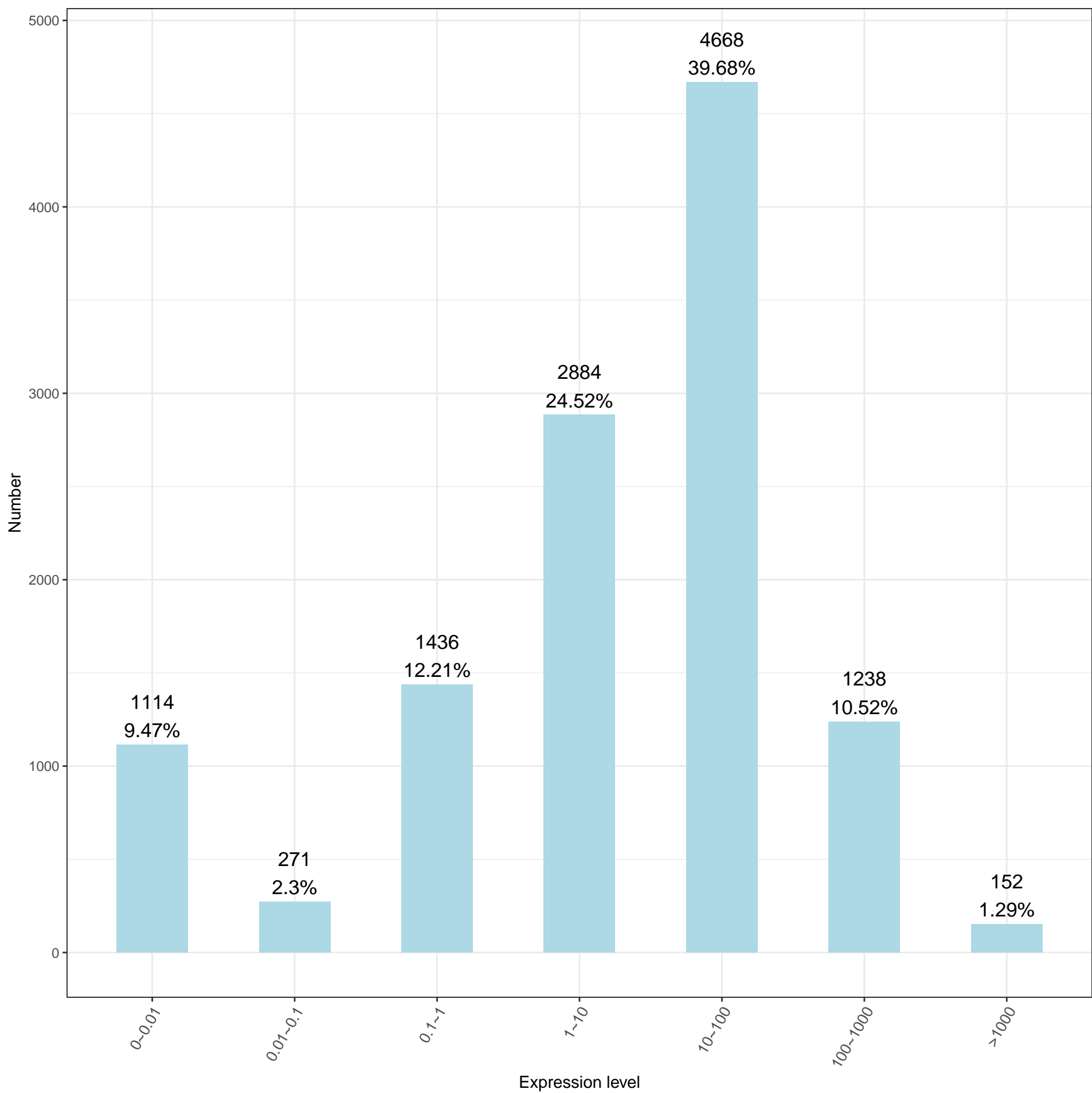

Supplement: Supplementary file 1 [file Data_Sheet_1.ZIP › mRNA/1_Expression/D2.fpkm_distribution.pdf]

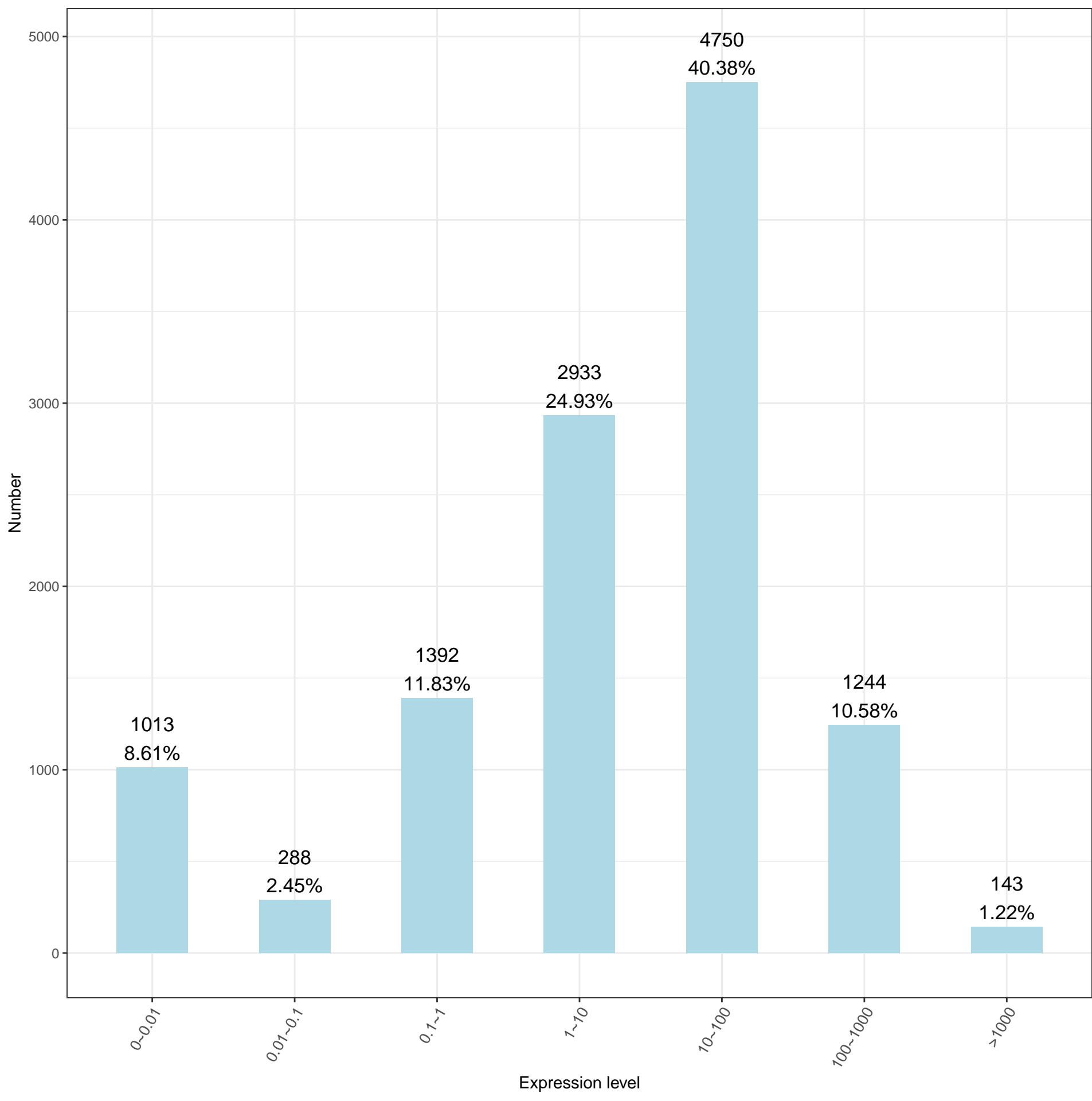

Supplement: Supplementary file 1 [file Data_Sheet_1.ZIP › mRNA/1_Expression/D3.fpkm_distribution.pdf]

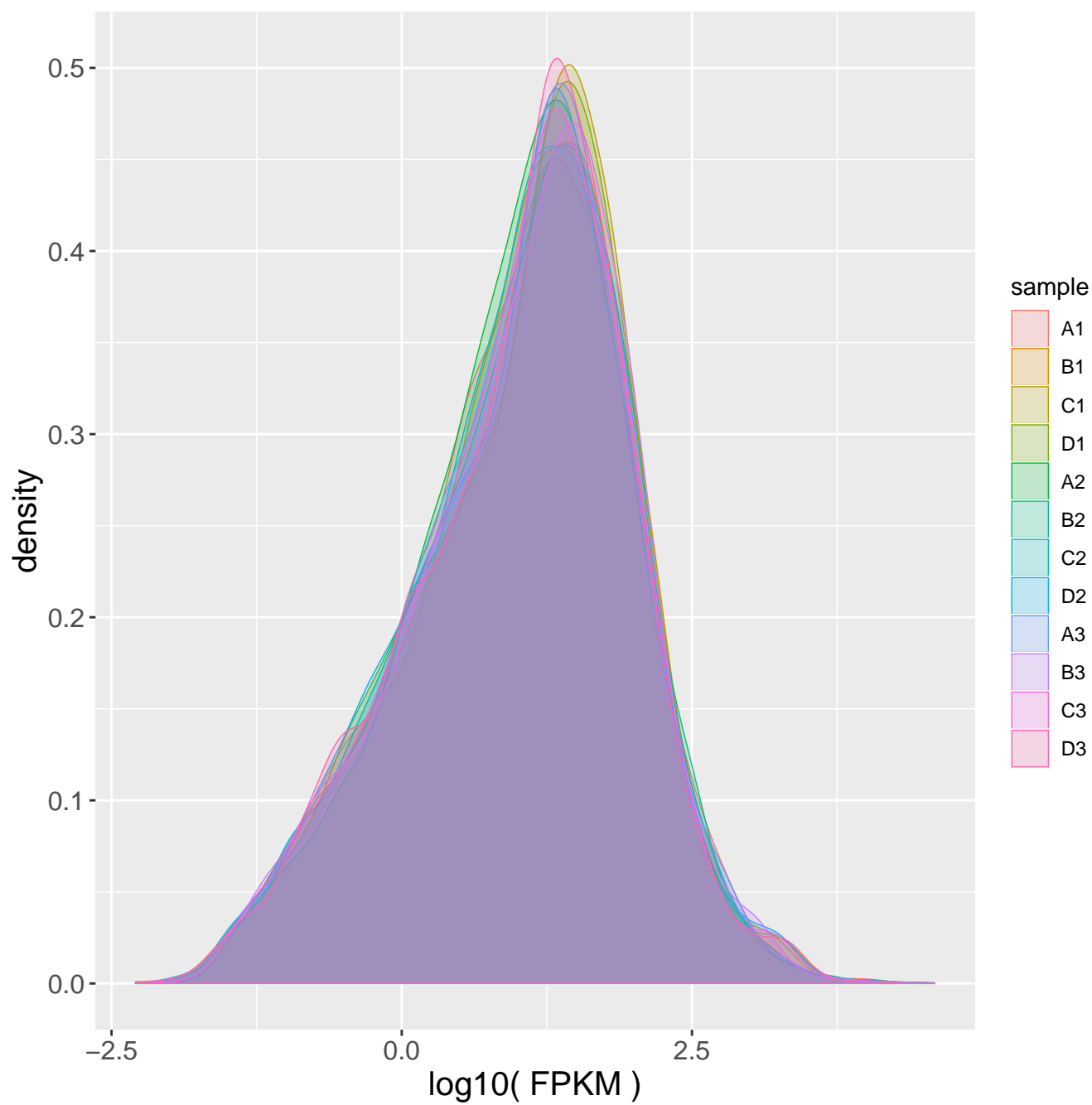

Supplement: Supplementary file 1 [file Data_Sheet_1.ZIP › mRNA/1_Expression/density.pdf]

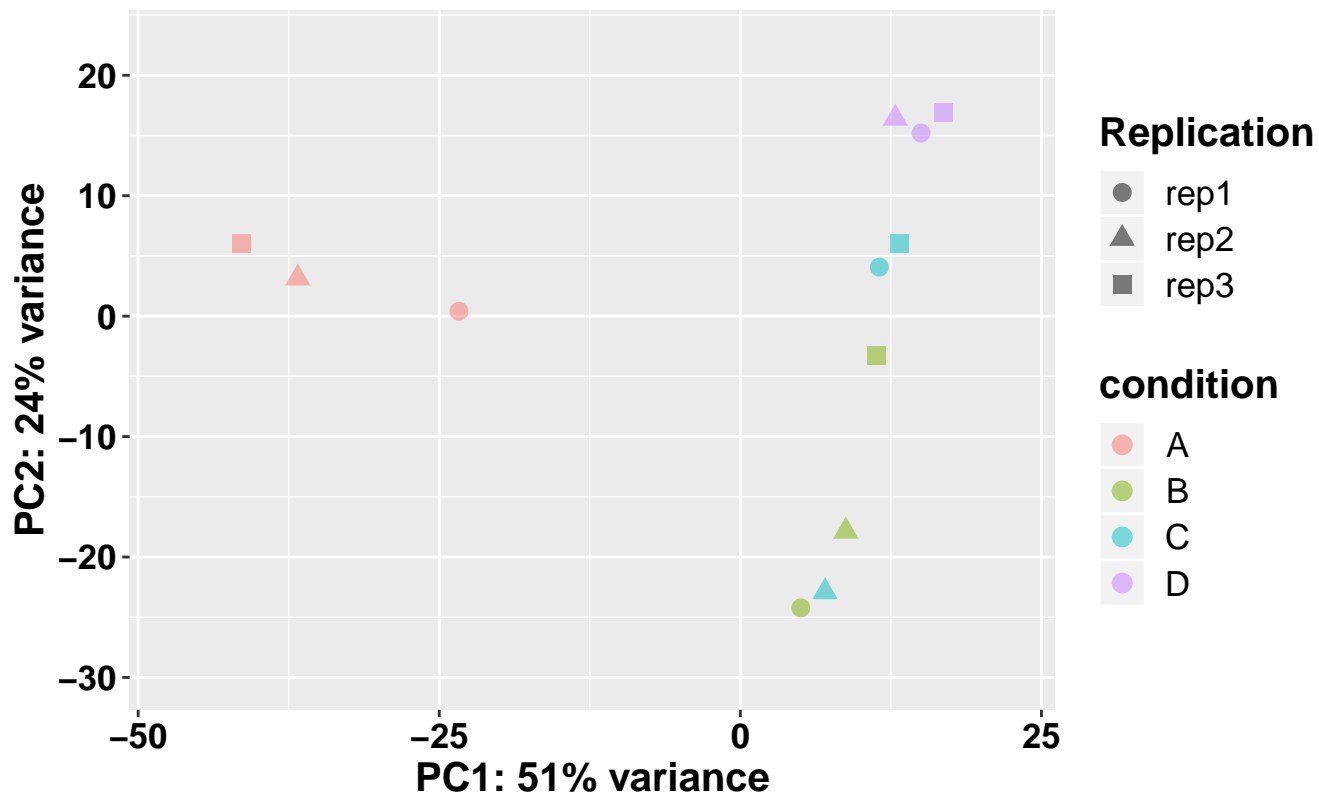

Supplement: Supplementary file 1 [file Data_Sheet_1.ZIP › mRNA/1_Expression/PCA.pdf]

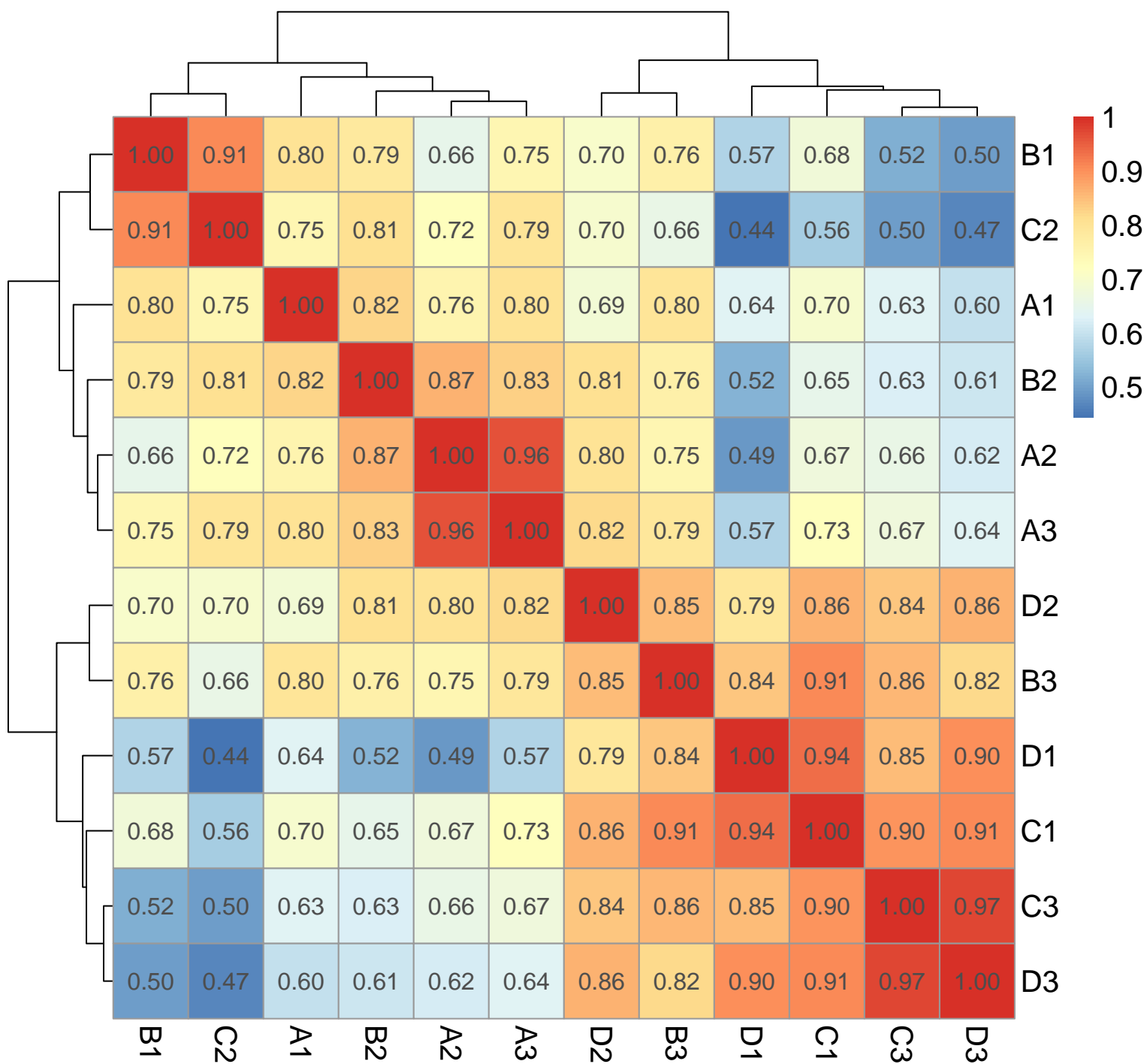

Supplement: Supplementary file 1 [file Data_Sheet_1.ZIP › mRNA/1_Expression/sample_cor_matrix.pdf]

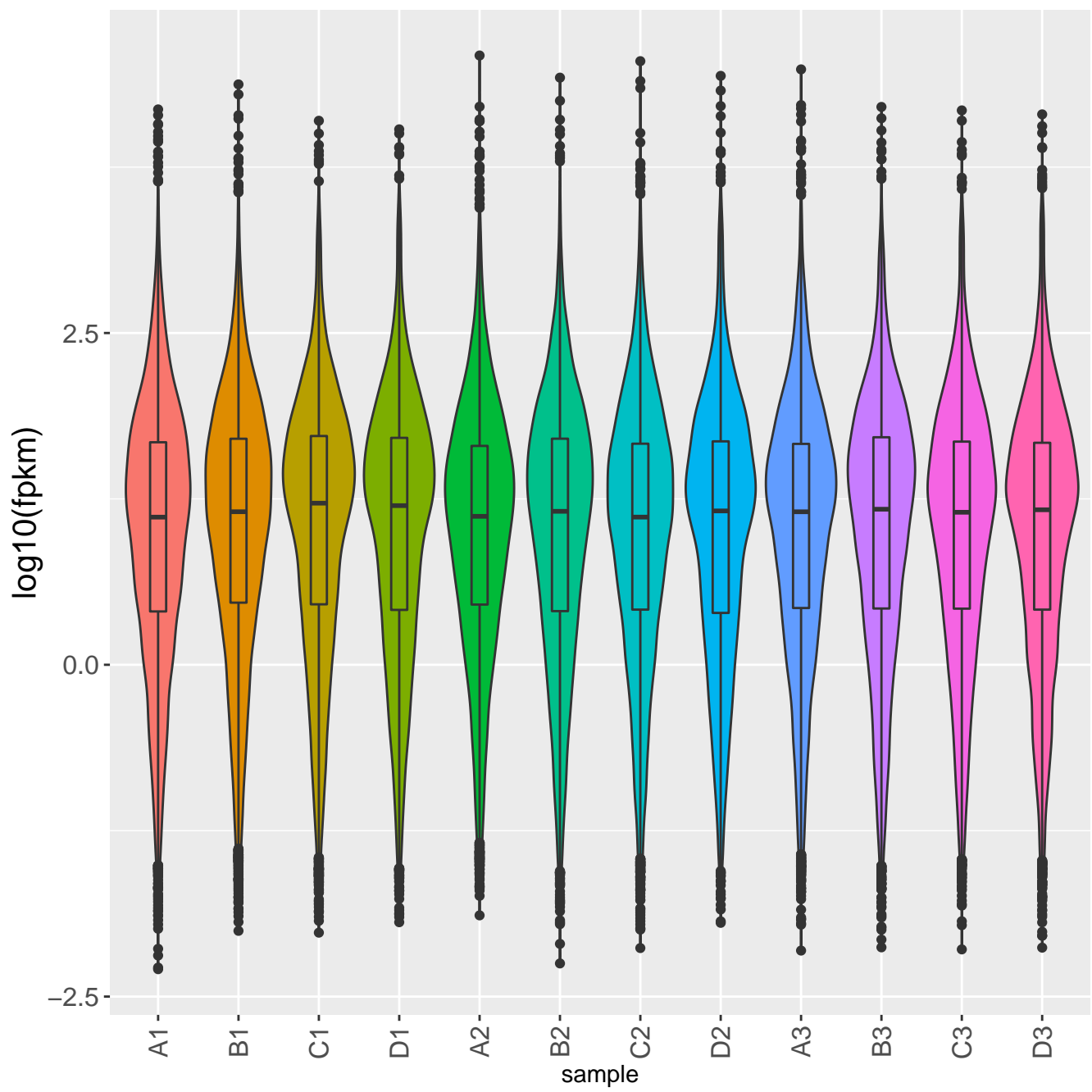

Supplement: Supplementary file 1 [file Data_Sheet_1.ZIP › mRNA/1_Expression/violin_plot.pdf]

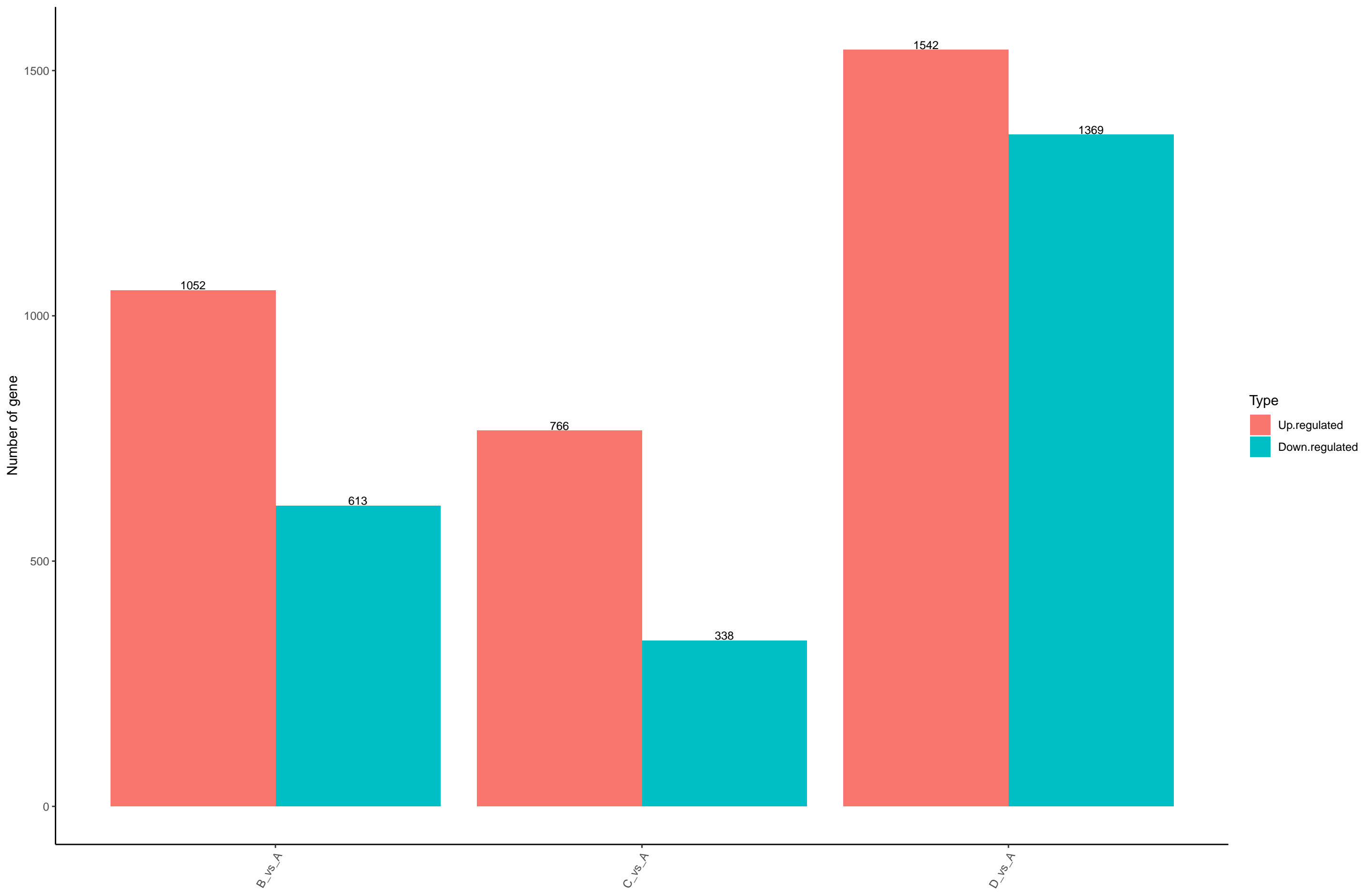

Supplement: Supplementary file 1 [file Data_Sheet_1.ZIP › mRNA/2_DEG/DESeq/DEG_stat.pdf]

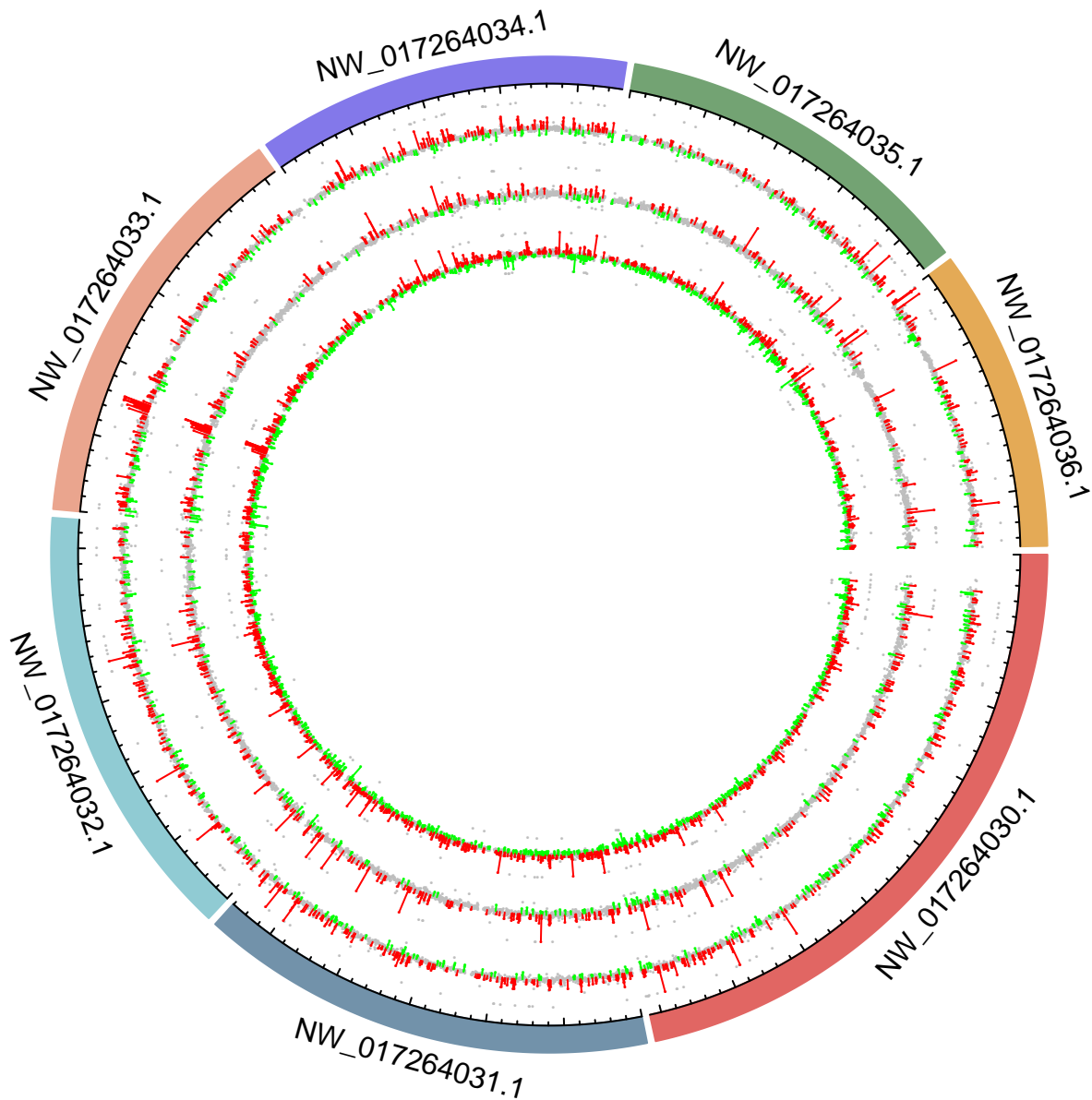

Supplement: Supplementary file 1 [file Data_Sheet_1.ZIP › mRNA/2_DEG/genomeCircos/genomeCircos.pdf]

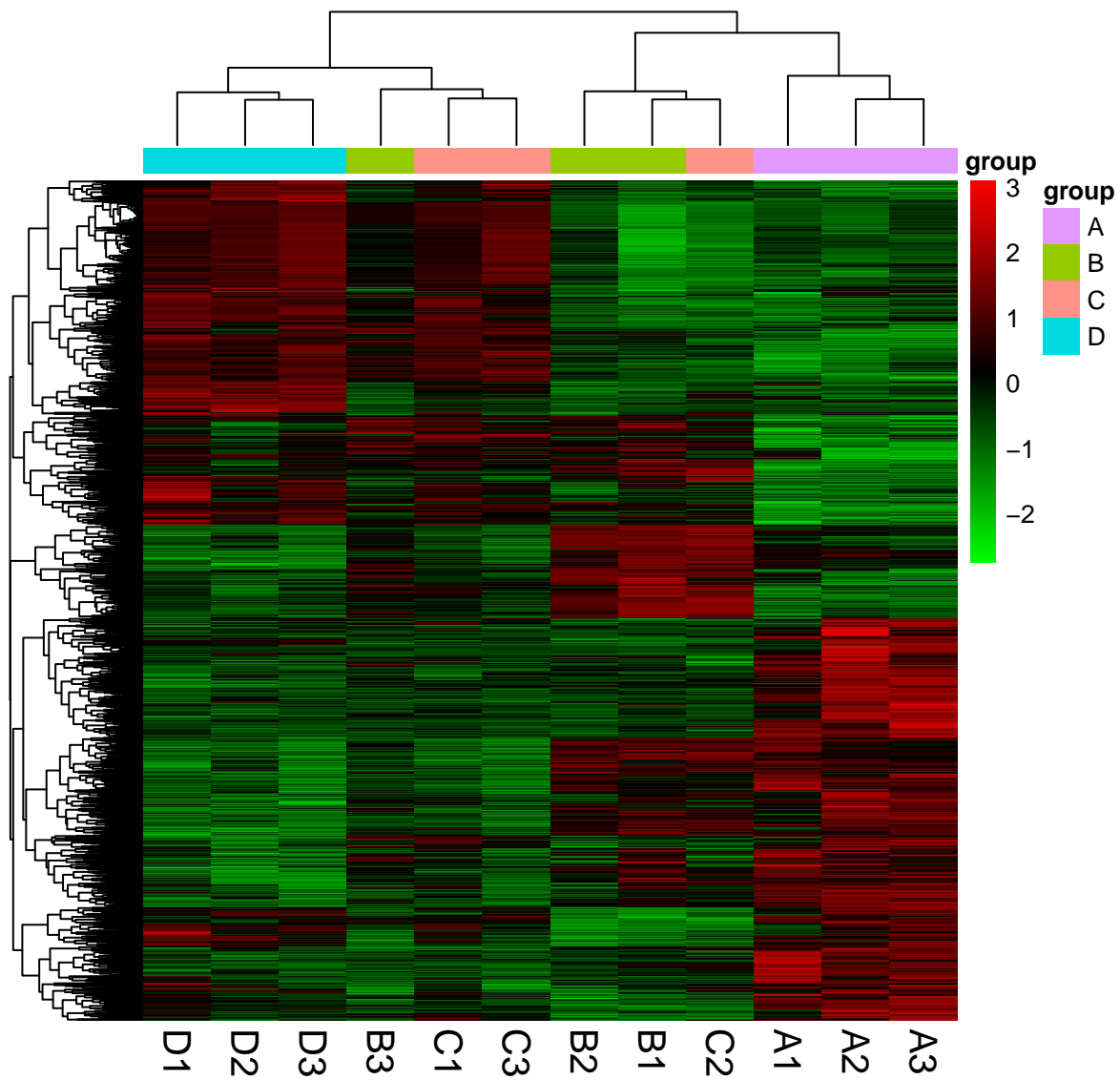

Supplement: Supplementary file 1 [file Data_Sheet_1.ZIP › mRNA/2_DEG/Heatmap/all.pdf]

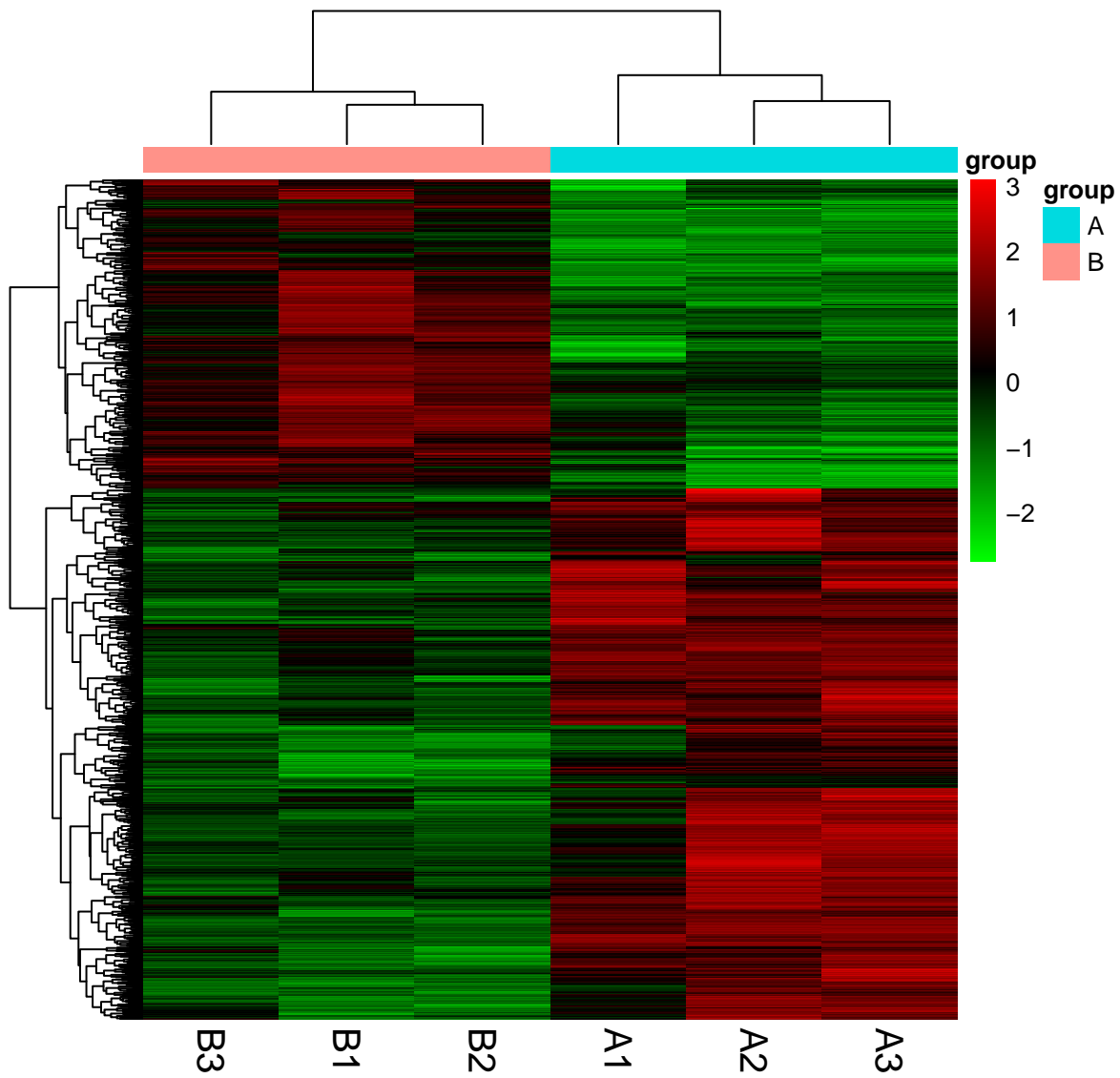

Supplement: Supplementary file 1 [file Data_Sheet_1.ZIP › mRNA/2_DEG/Heatmap/B_vs_A.pdf]

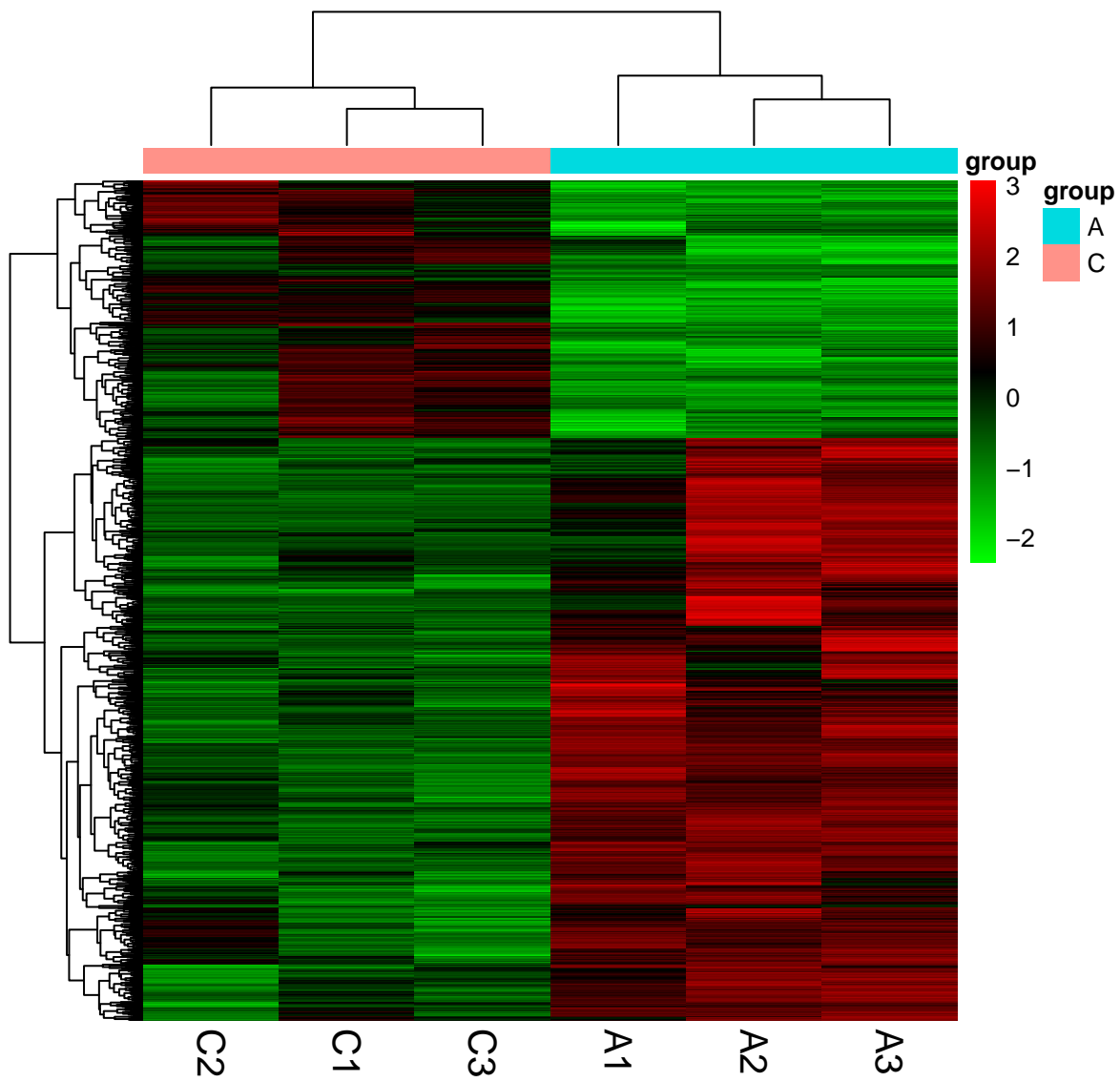

Supplement: Supplementary file 1 [file Data_Sheet_1.ZIP › mRNA/2_DEG/Heatmap/C_vs_A.pdf]

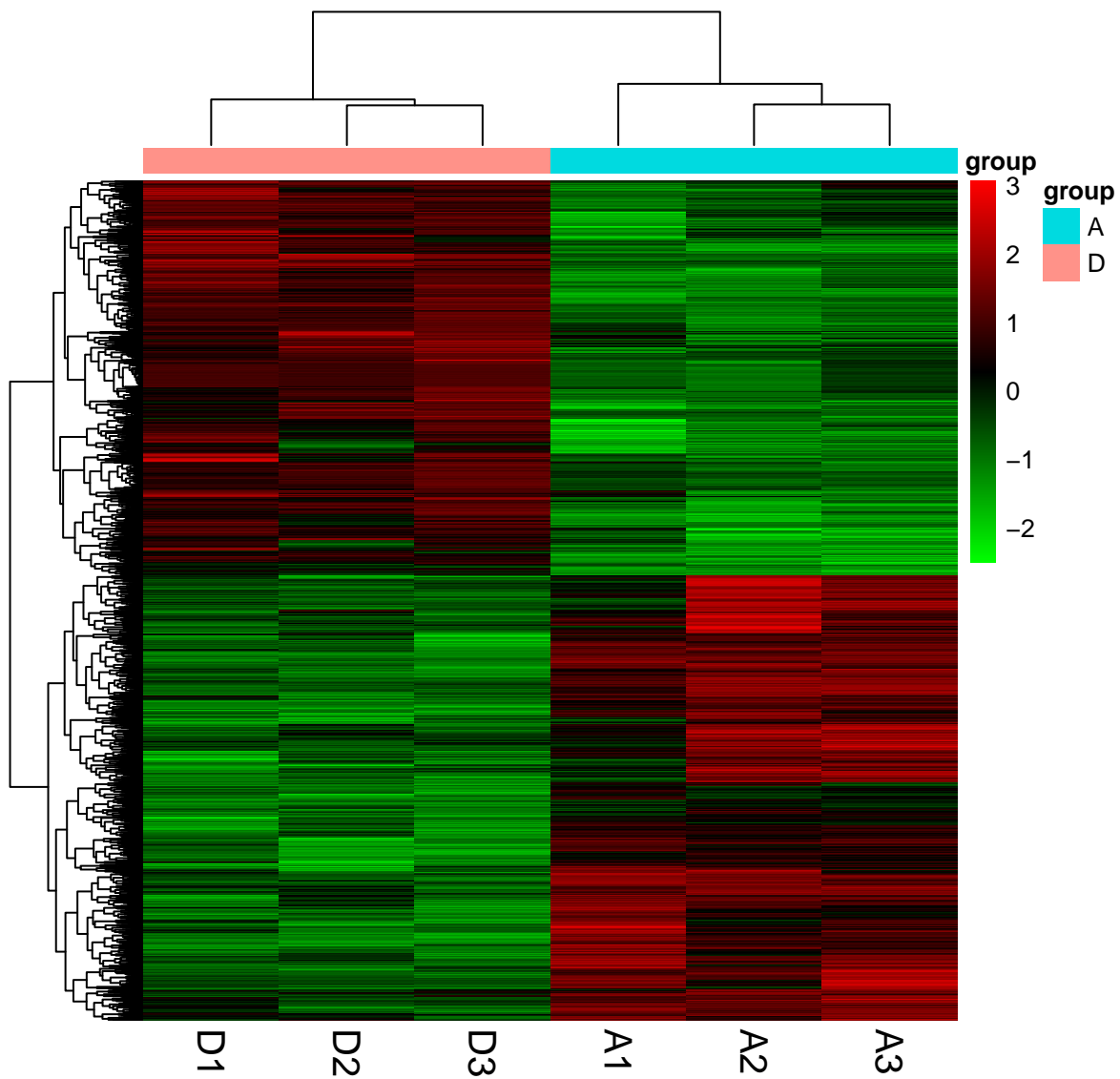

Supplement: Supplementary file 1 [file Data_Sheet_1.ZIP › mRNA/2_DEG/Heatmap/D_vs_A.pdf]

**clust1**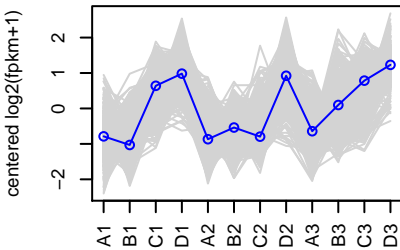**clust2**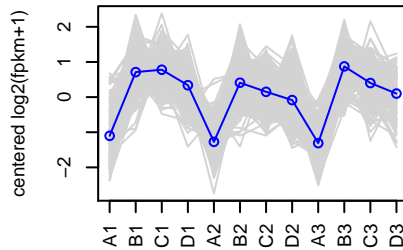**clust3**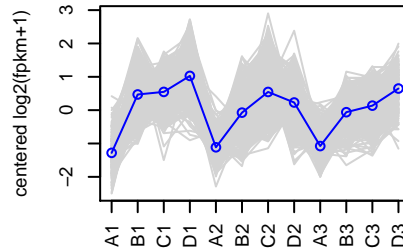**clust4**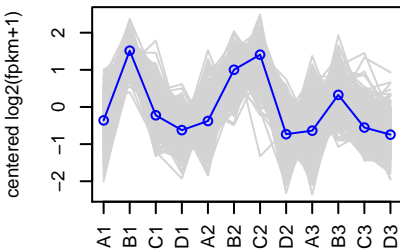**clust5**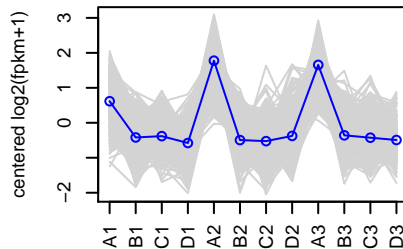**clust6**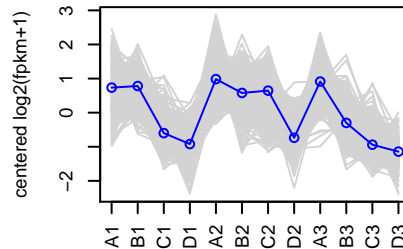**clust7**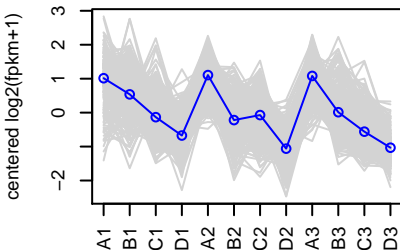**clust8**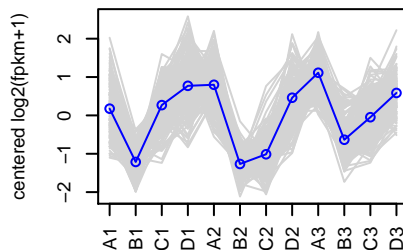**clust9**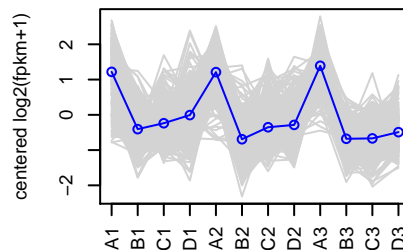

Supplement: Supplementary file 1 [file Data_Sheet_1.ZIP › mRNA/2_DEG/trend/all_cluster_plots.pdf]

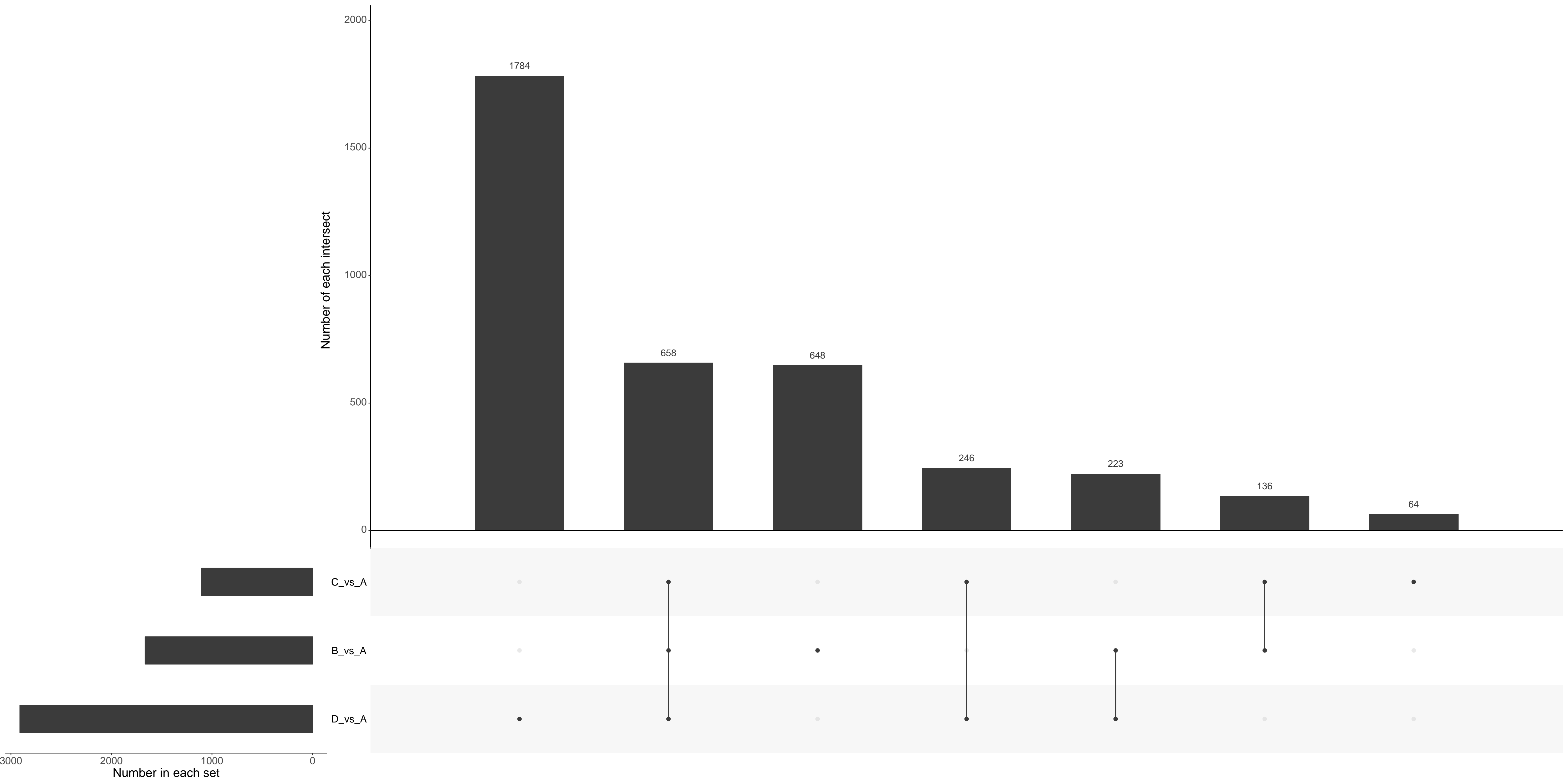

Supplement: Supplementary file 1 [file Data_Sheet_1.ZIP › mRNA/2_DEG/Venn/DEG_UpSet.pdf]

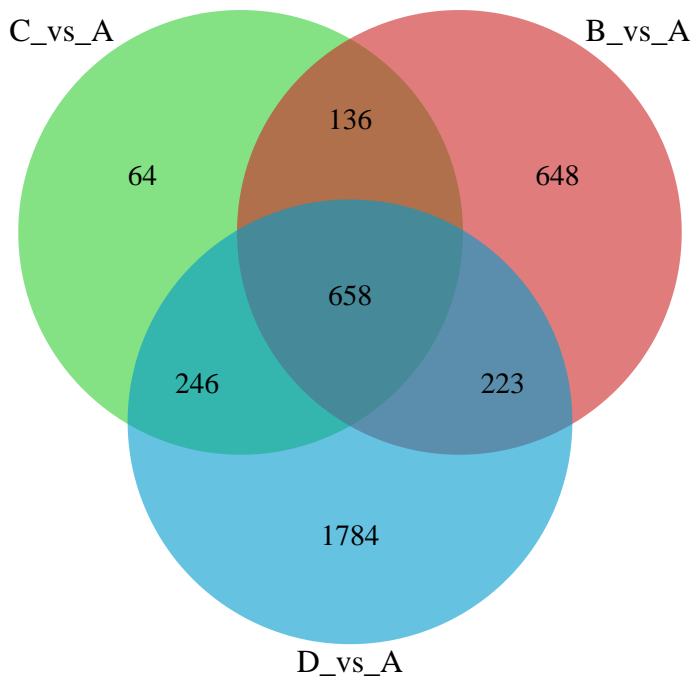

Supplement: Supplementary file 1 [file Data_Sheet_1.ZIP › mRNA/2_DEG/Venn/venn.pdf]

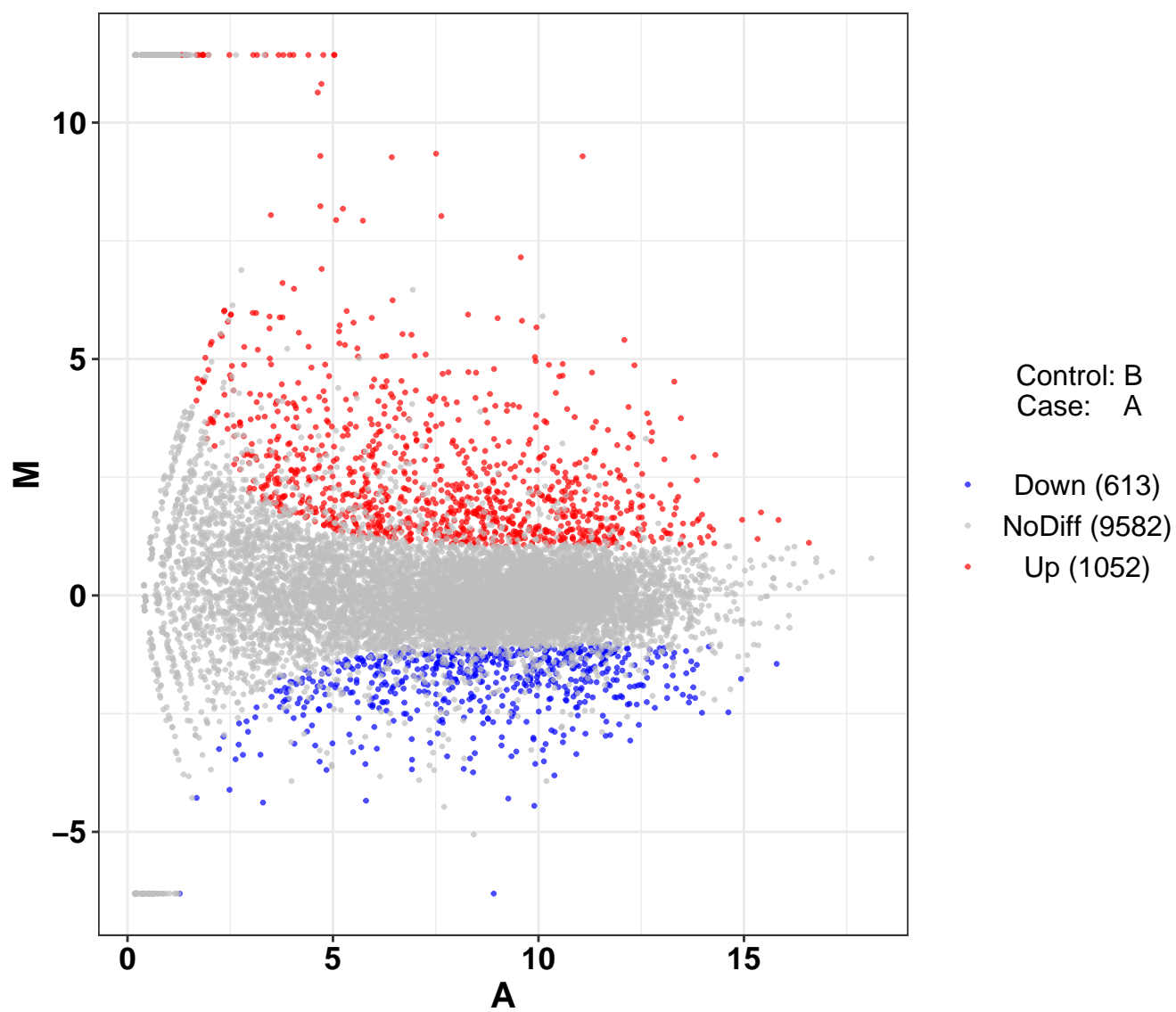

Supplement: Supplementary file 1 [file Data_Sheet_1.ZIP › mRNA/2_DEG/V_MA/B_vs_A.MA.pdf]

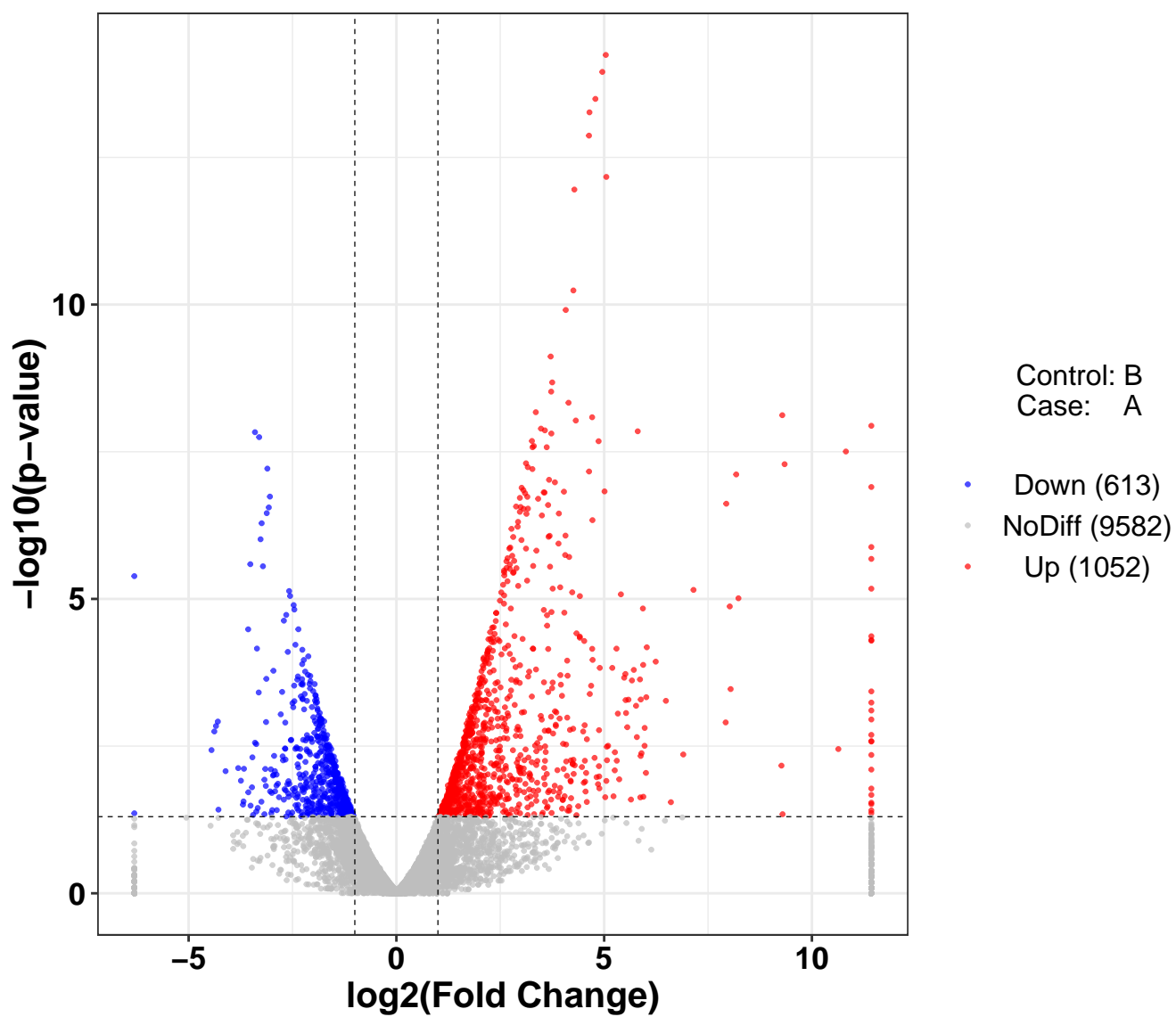

Supplement: Supplementary file 1 [file Data_Sheet_1.ZIP › mRNA/2_DEG/V_MA/B_vs_A.Volcano.pdf]

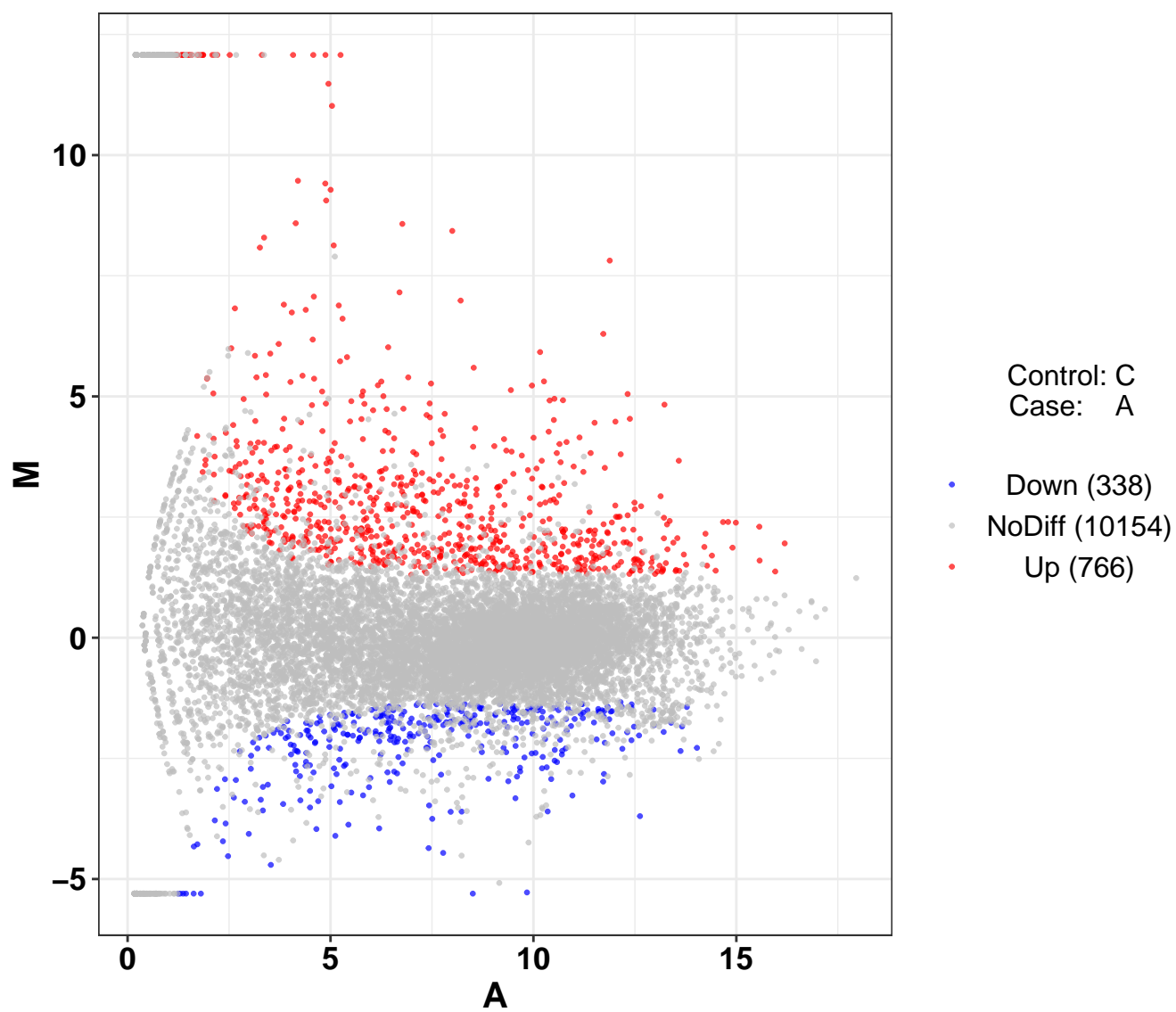

Supplement: Supplementary file 1 [file Data_Sheet_1.ZIP › mRNA/2_DEG/V_MA/C_vs_A.MA.pdf]

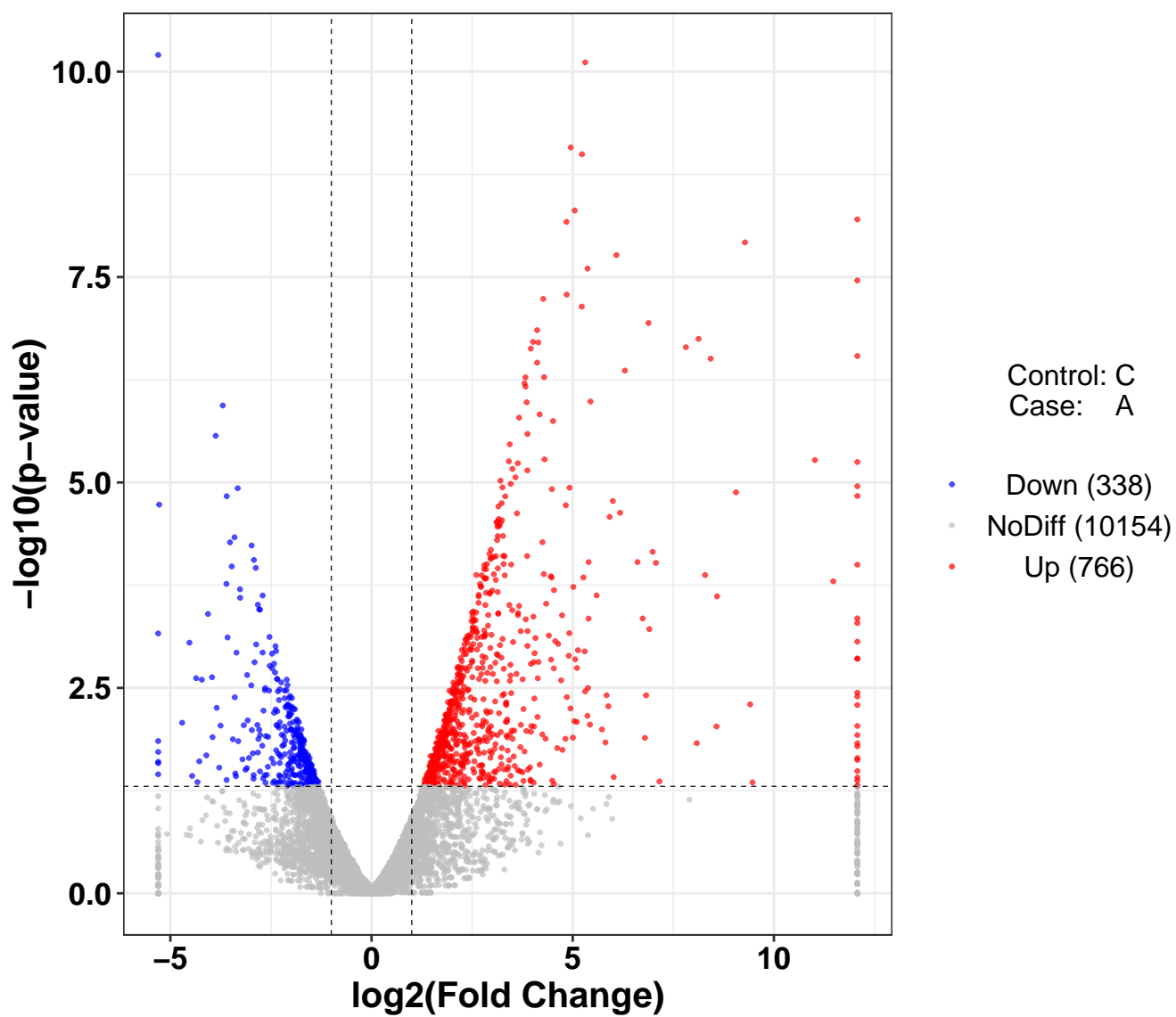

Supplement: Supplementary file 1 [file Data_Sheet_1.ZIP › mRNA/2_DEG/V_MA/C_vs_A.Volcano.pdf]

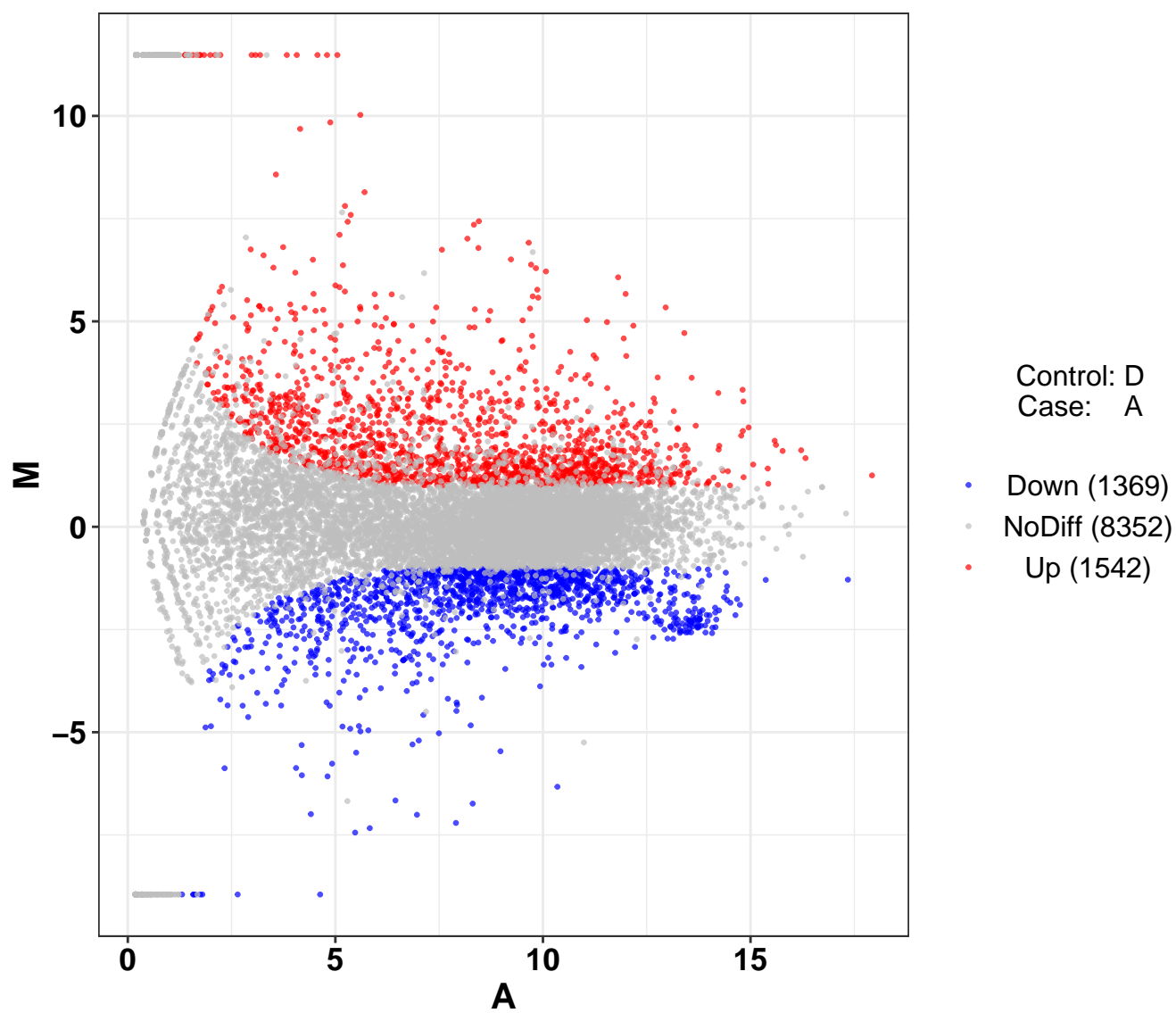

Supplement: Supplementary file 1 [file Data_Sheet_1.ZIP › mRNA/2_DEG/V_MA/D_vs_A.MA.pdf]

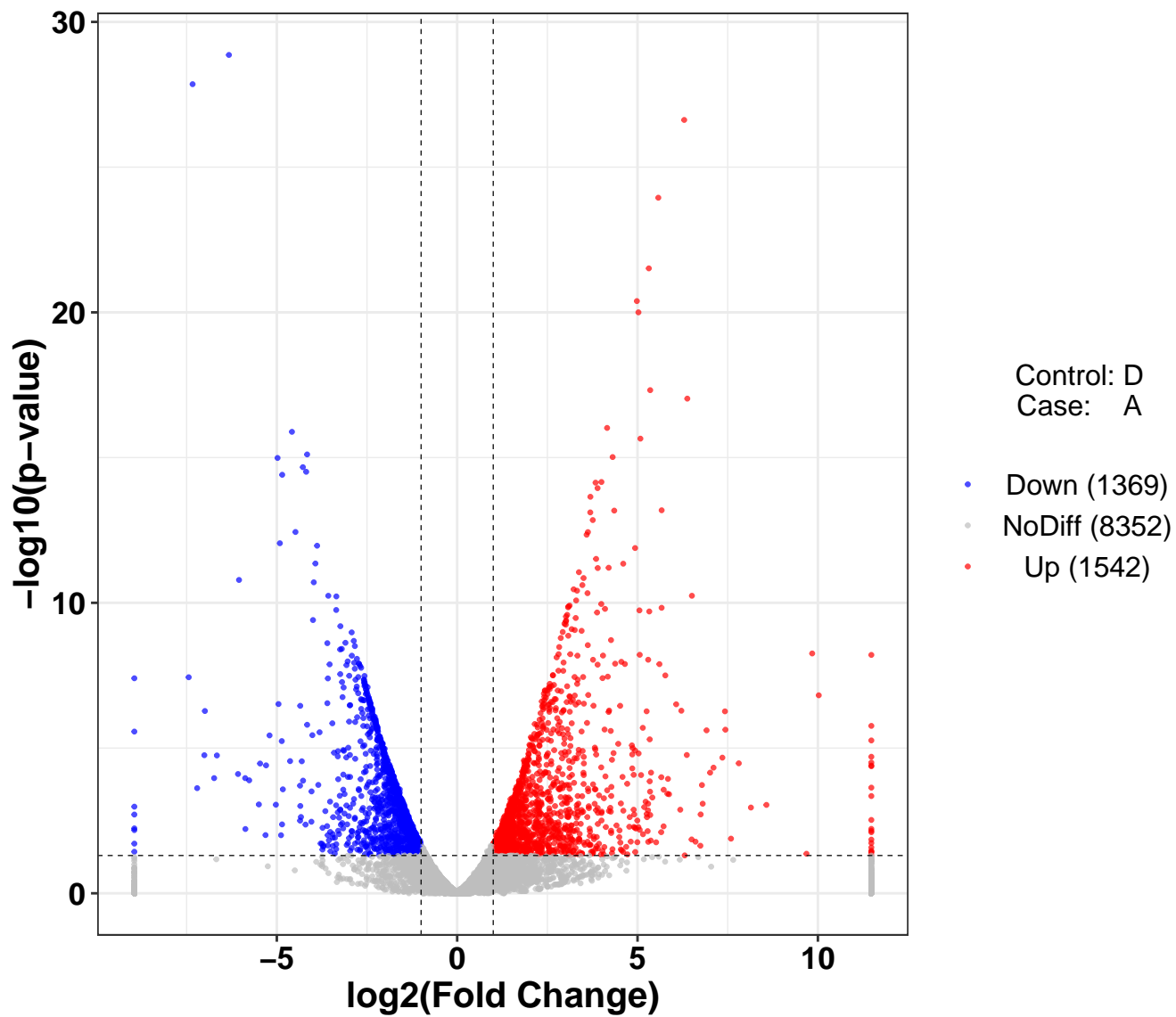

Supplement: Supplementary file 1 [file Data_Sheet_1.ZIP › mRNA/2_DEG/V_MA/D_vs_A.Volcano.pdf]

# GO Enrichment

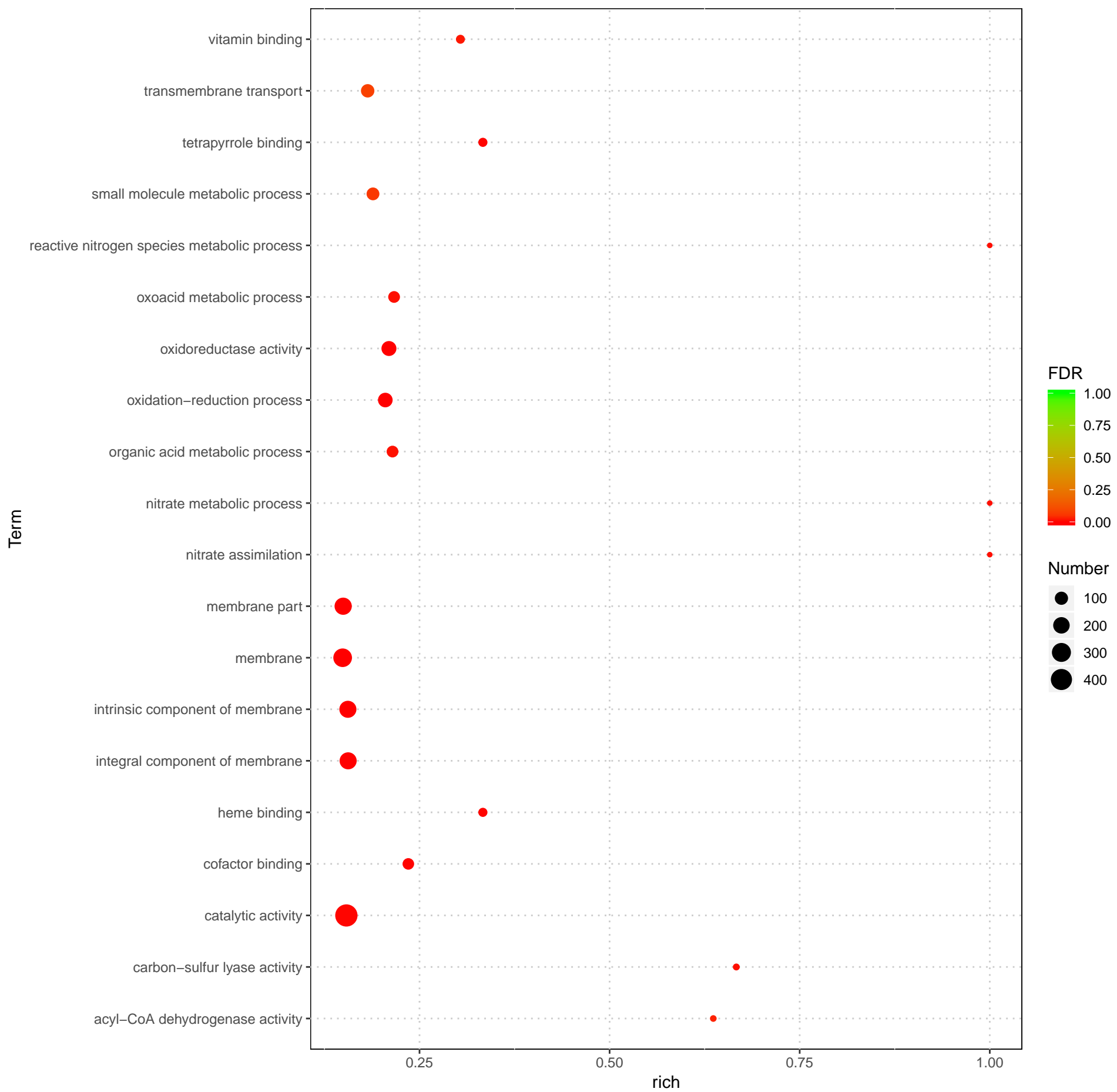

Supplement: Supplementary file 1 [file Data_Sheet_1.ZIP › mRNA/3_Enrichment/B_vs_A/GO.richfactor.pdf]

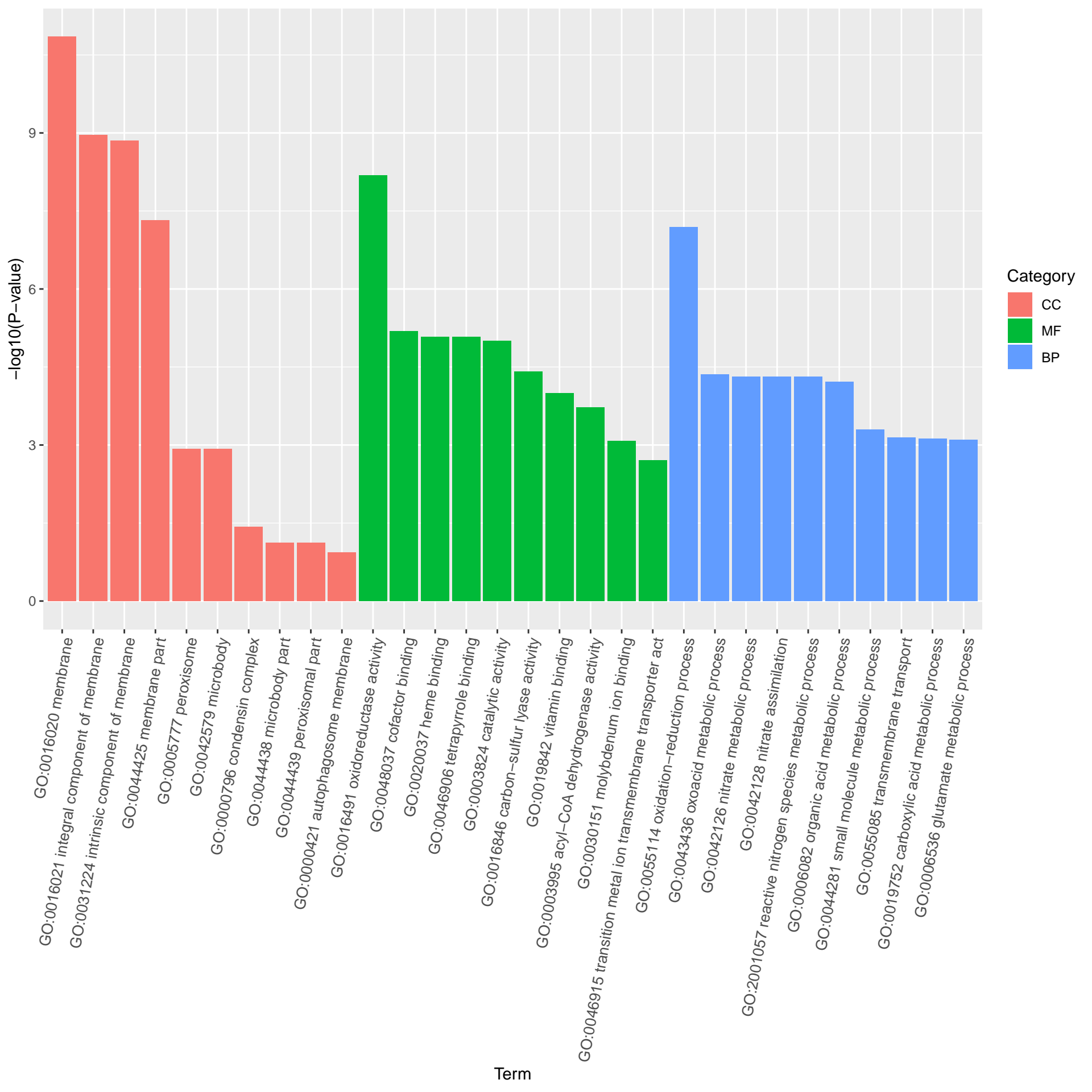

Supplement: Supplementary file 1 [file Data_Sheet_1.ZIP › mRNA/3_Enrichment/B_vs_A/GO_enrichment_pvalue_barplot.pdf]

# KEGG Pathway Enrichment

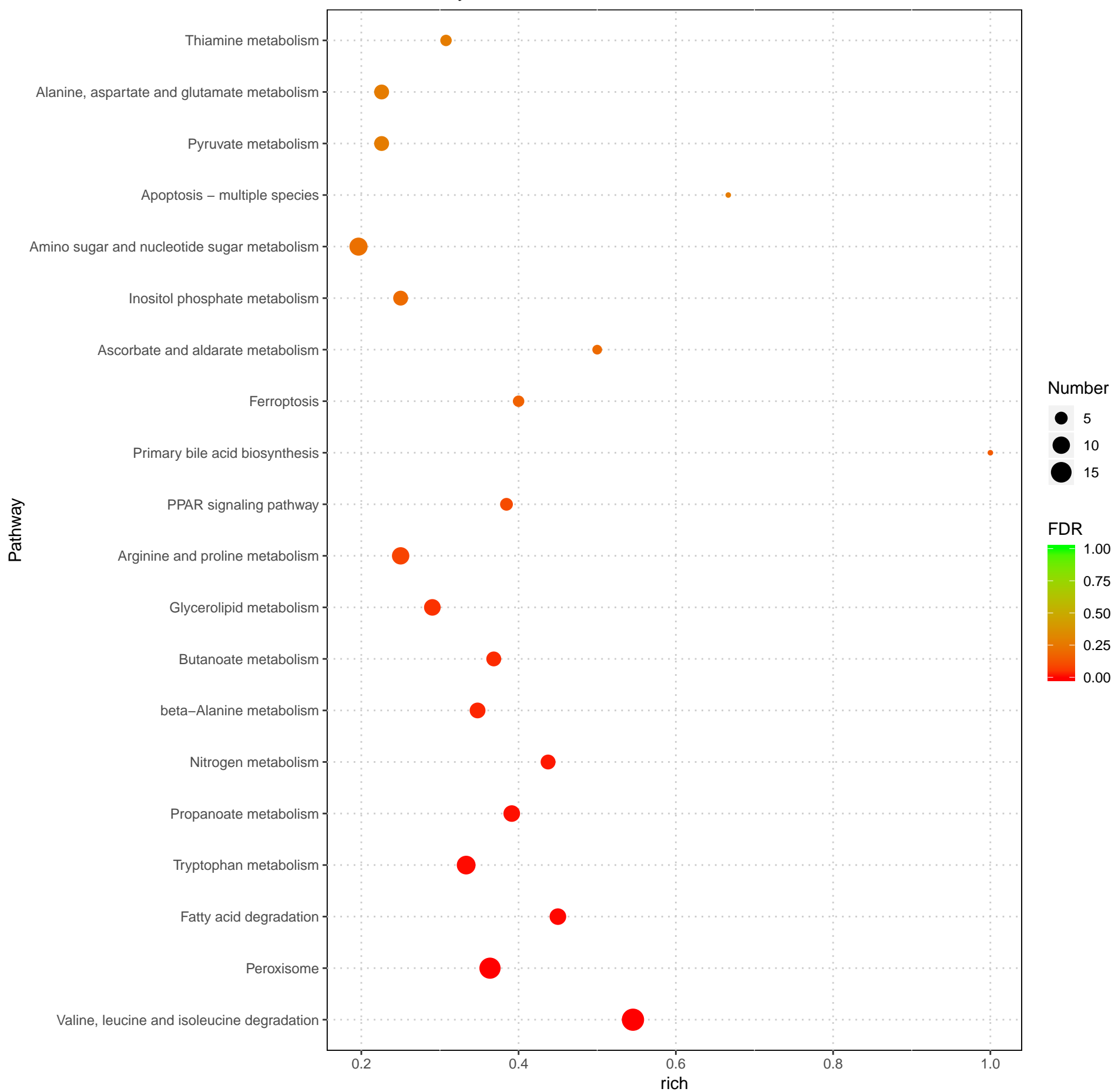

Supplement: Supplementary file 1 [file Data_Sheet_1.ZIP › mRNA/3_Enrichment/B_vs_A/KEGG.richfactor.pdf]

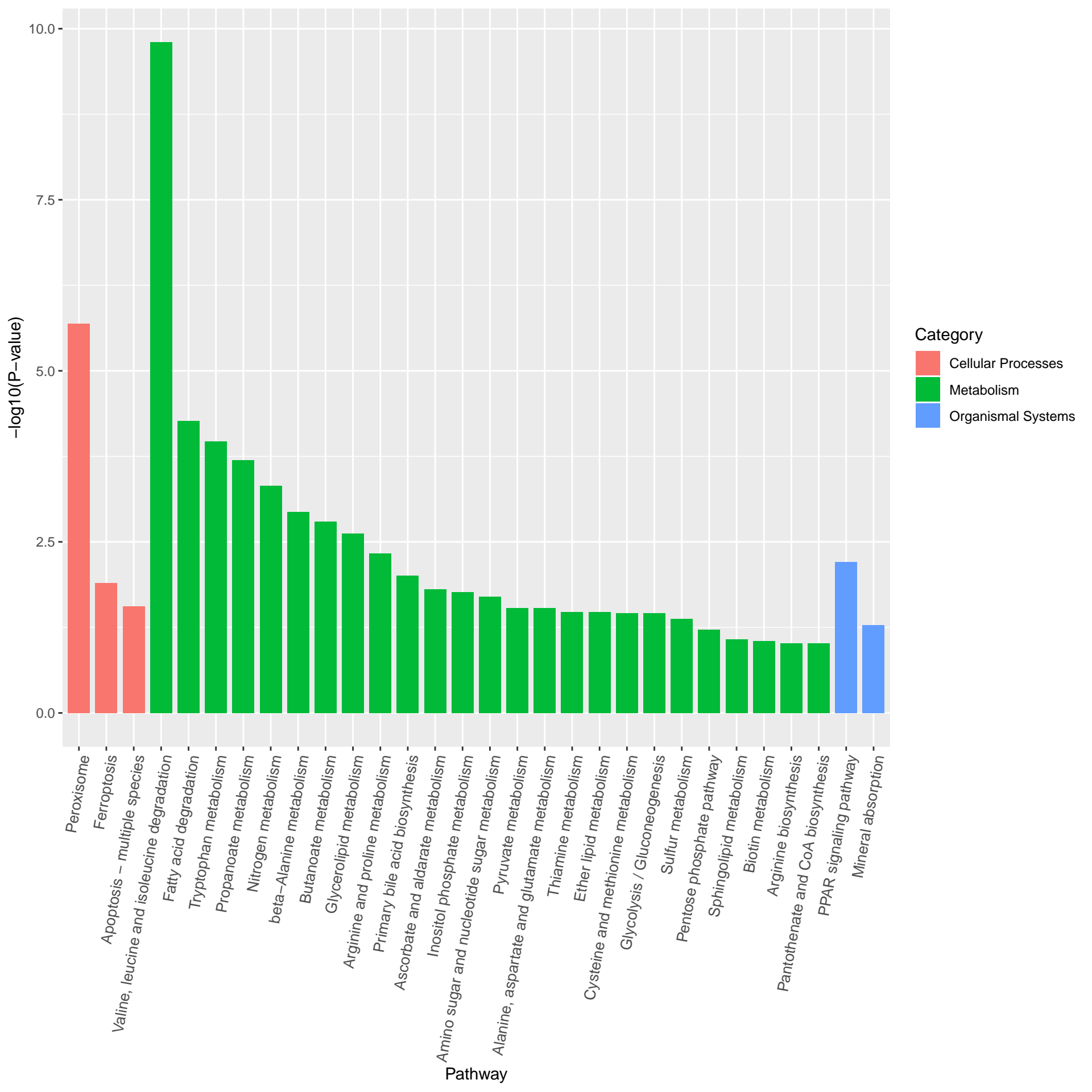

Supplement: Supplementary file 1 [file Data_Sheet_1.ZIP › mRNA/3_Enrichment/B_vs_A/KEGG_enrichment_pvalue_barplot.pdf]

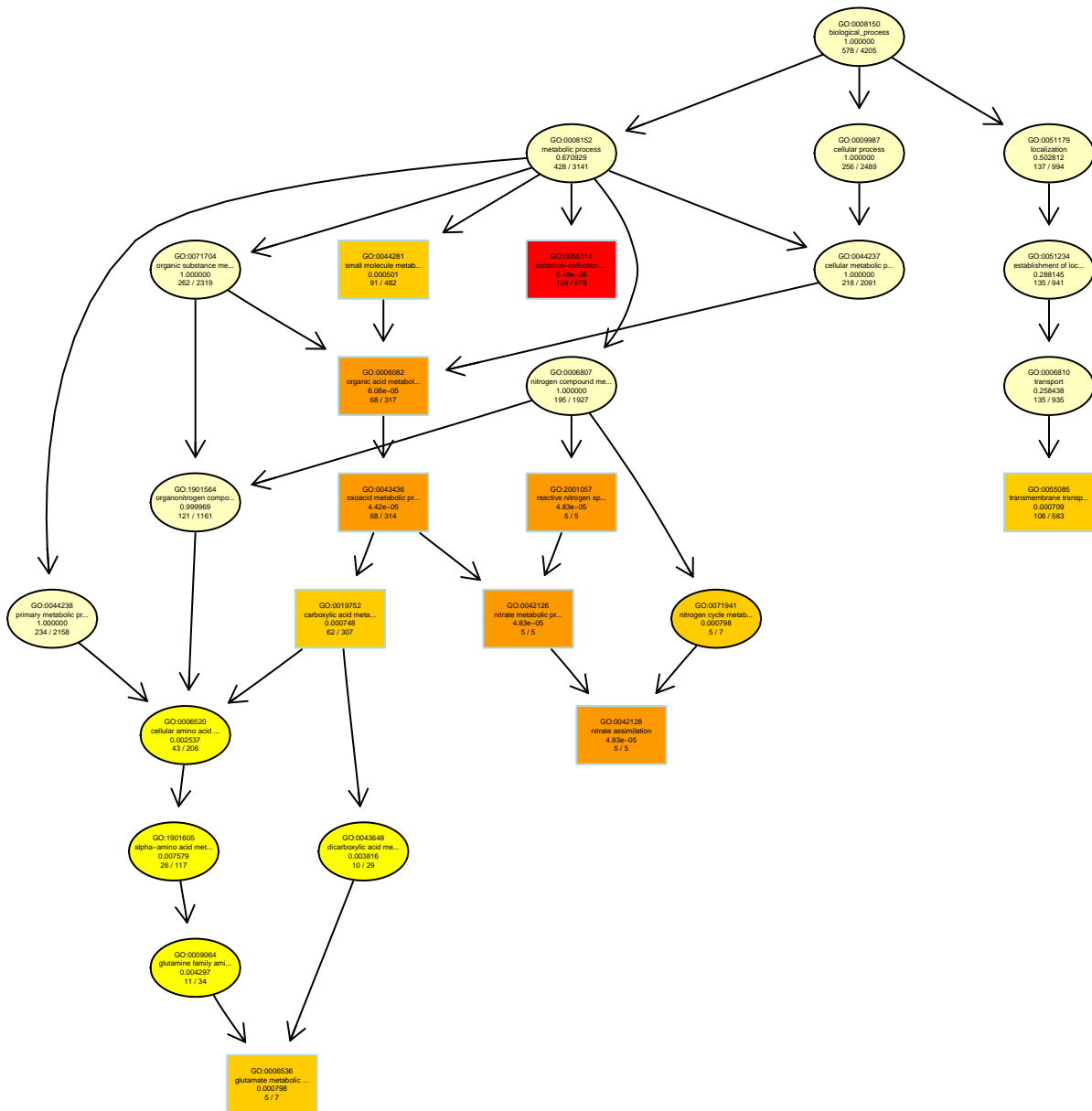

Supplement: Supplementary file 1 [file Data_Sheet_1.ZIP › mRNA/3_Enrichment/B_vs_A/topGO_BP_top10.pdf]

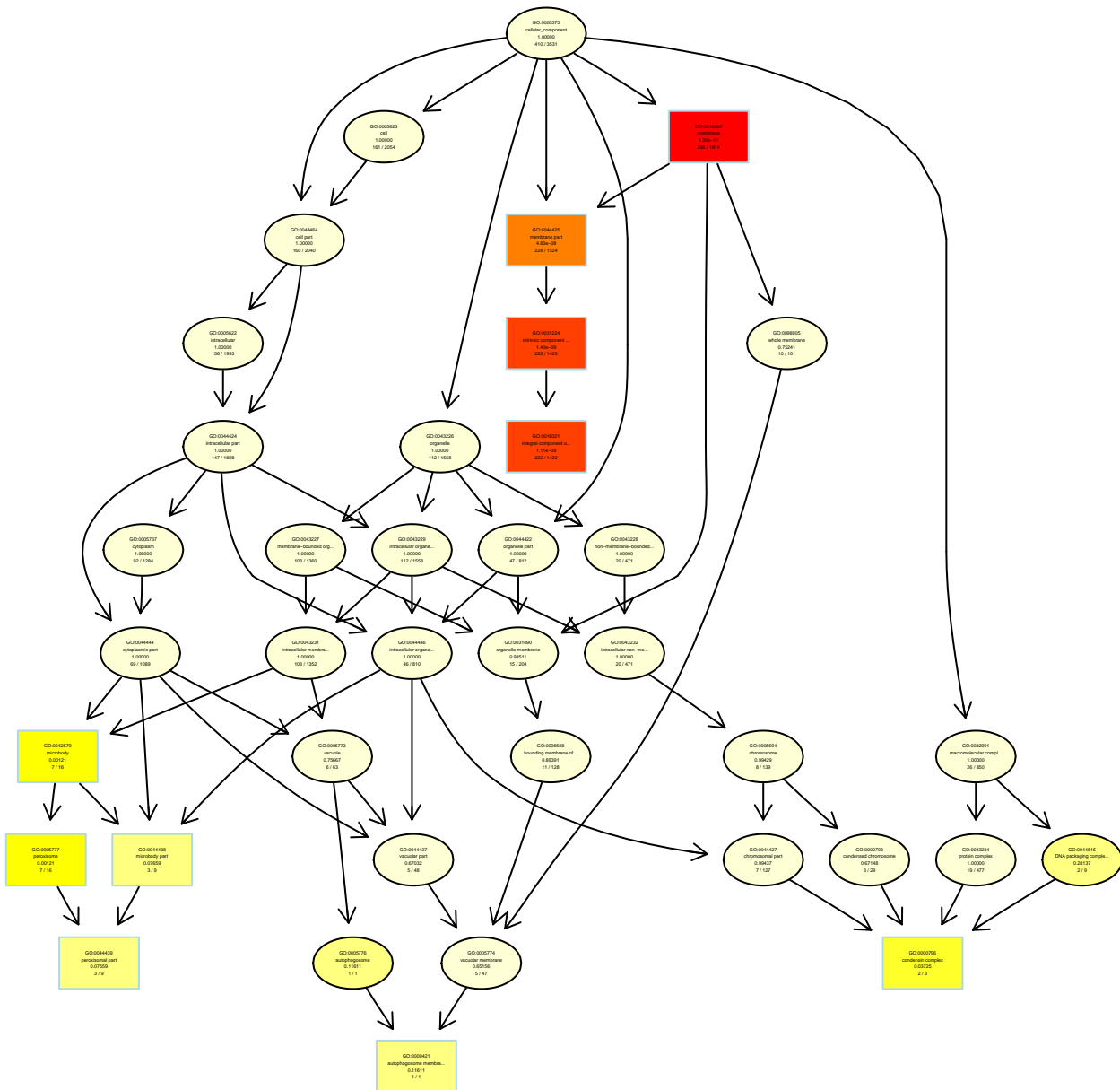

Supplement: Supplementary file 1 [file Data_Sheet_1.ZIP › mRNA/3_Enrichment/B_vs_A/topGO_CC_top10.pdf]

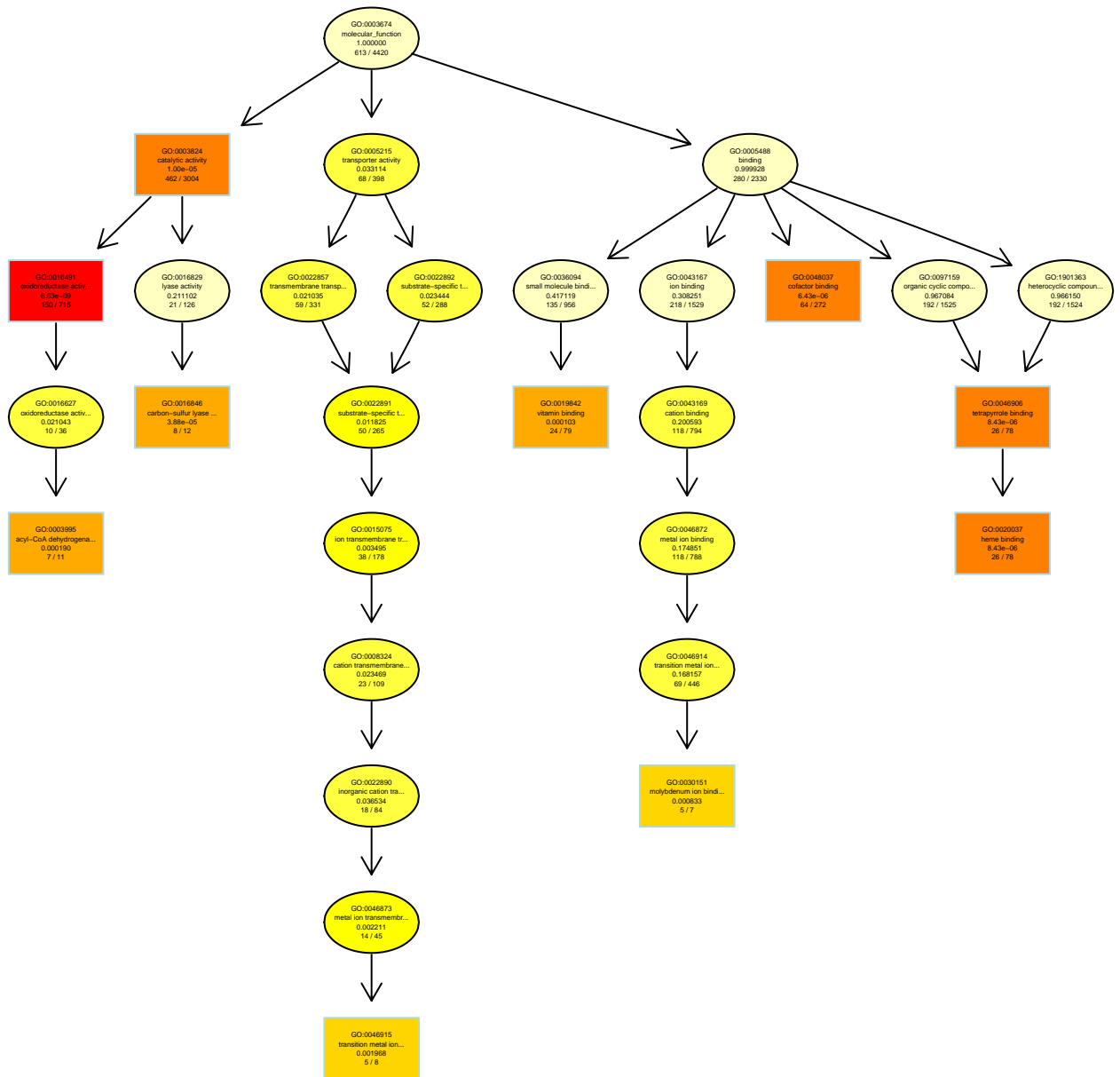

Supplement: Supplementary file 1 [file Data_Sheet_1.ZIP › mRNA/3_Enrichment/B_vs_A/topGO_MF_top10.pdf]

# GO Enrichment

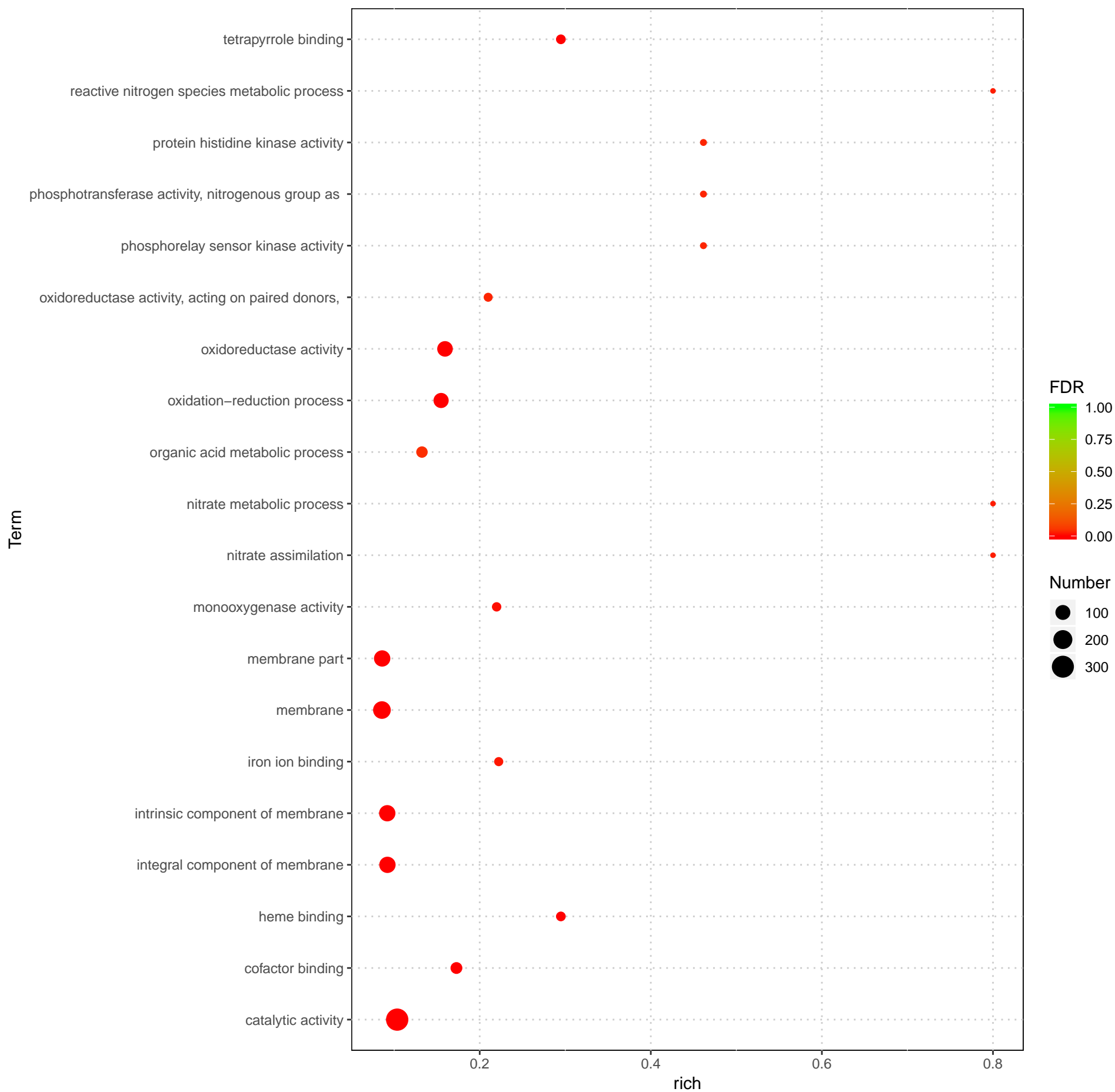

Supplement: Supplementary file 1 [file Data_Sheet_1.ZIP › mRNA/3_Enrichment/C_vs_A/GO.richfactor.pdf]

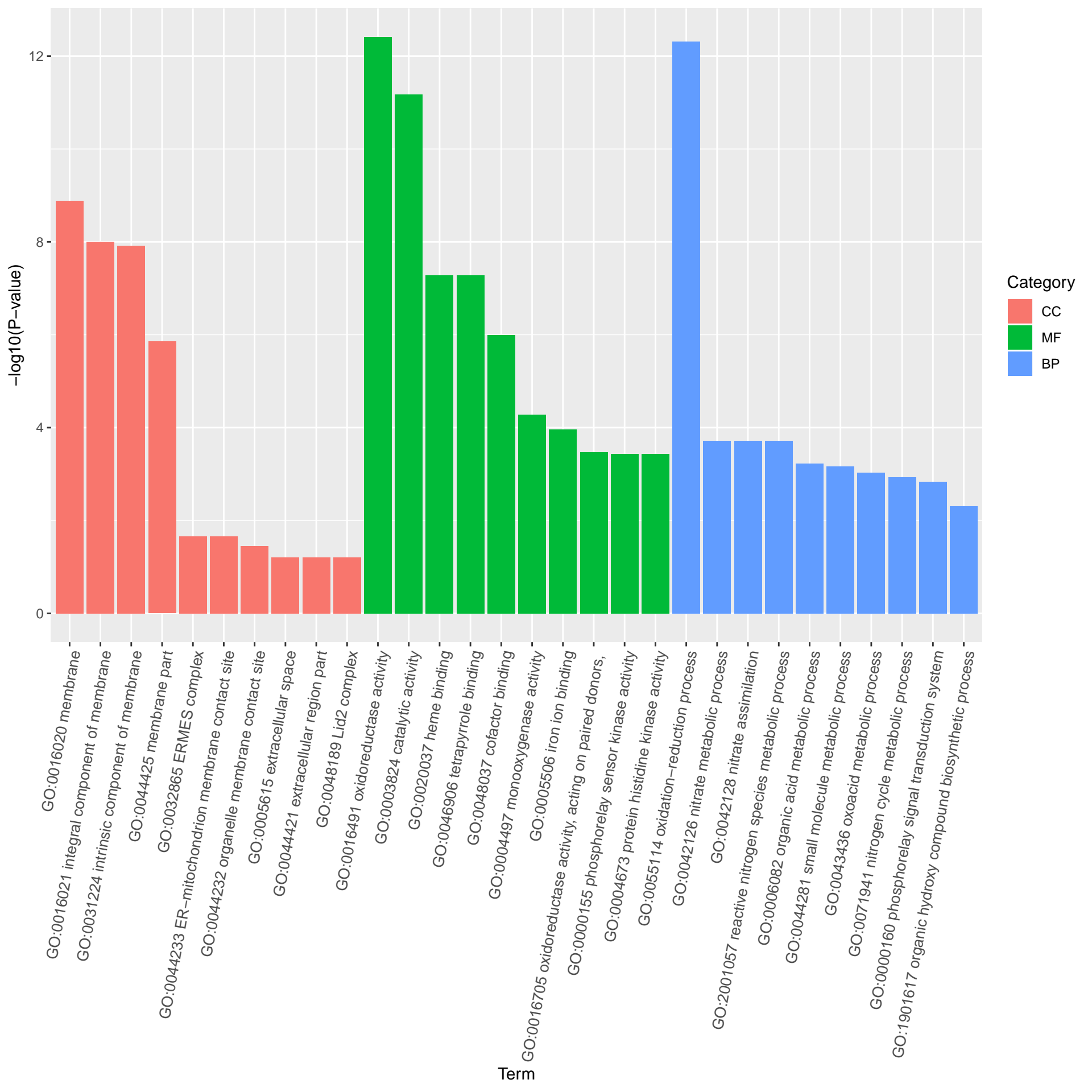

Supplement: Supplementary file 1 [file Data_Sheet_1.ZIP › mRNA/3_Enrichment/C_vs_A/GO_enrichment_pvalue_barplot.pdf]

# KEGG Pathway Enrichment

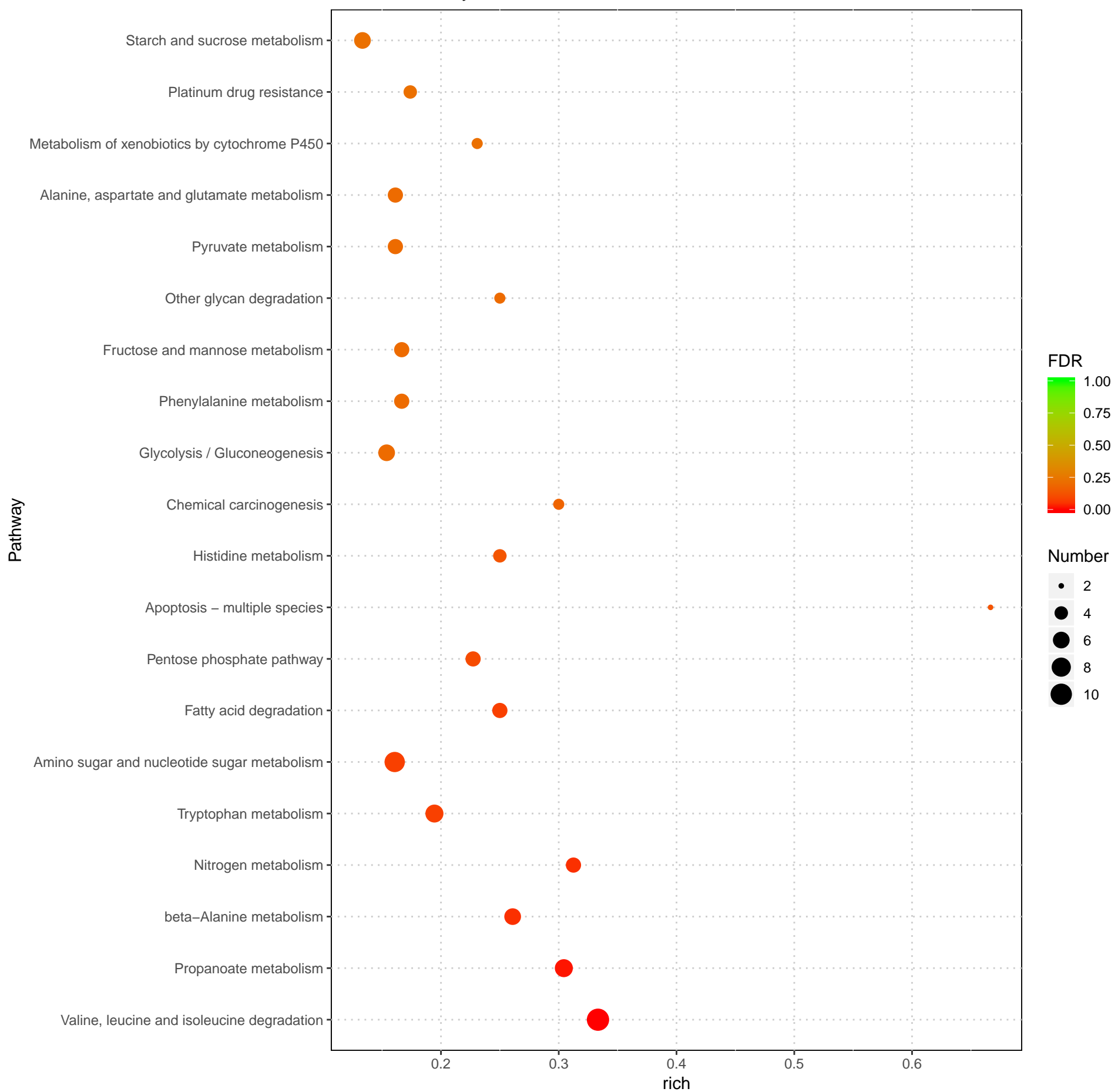

Supplement: Supplementary file 1 [file Data_Sheet_1.ZIP › mRNA/3_Enrichment/C_vs_A/KEGG.richfactor.pdf]

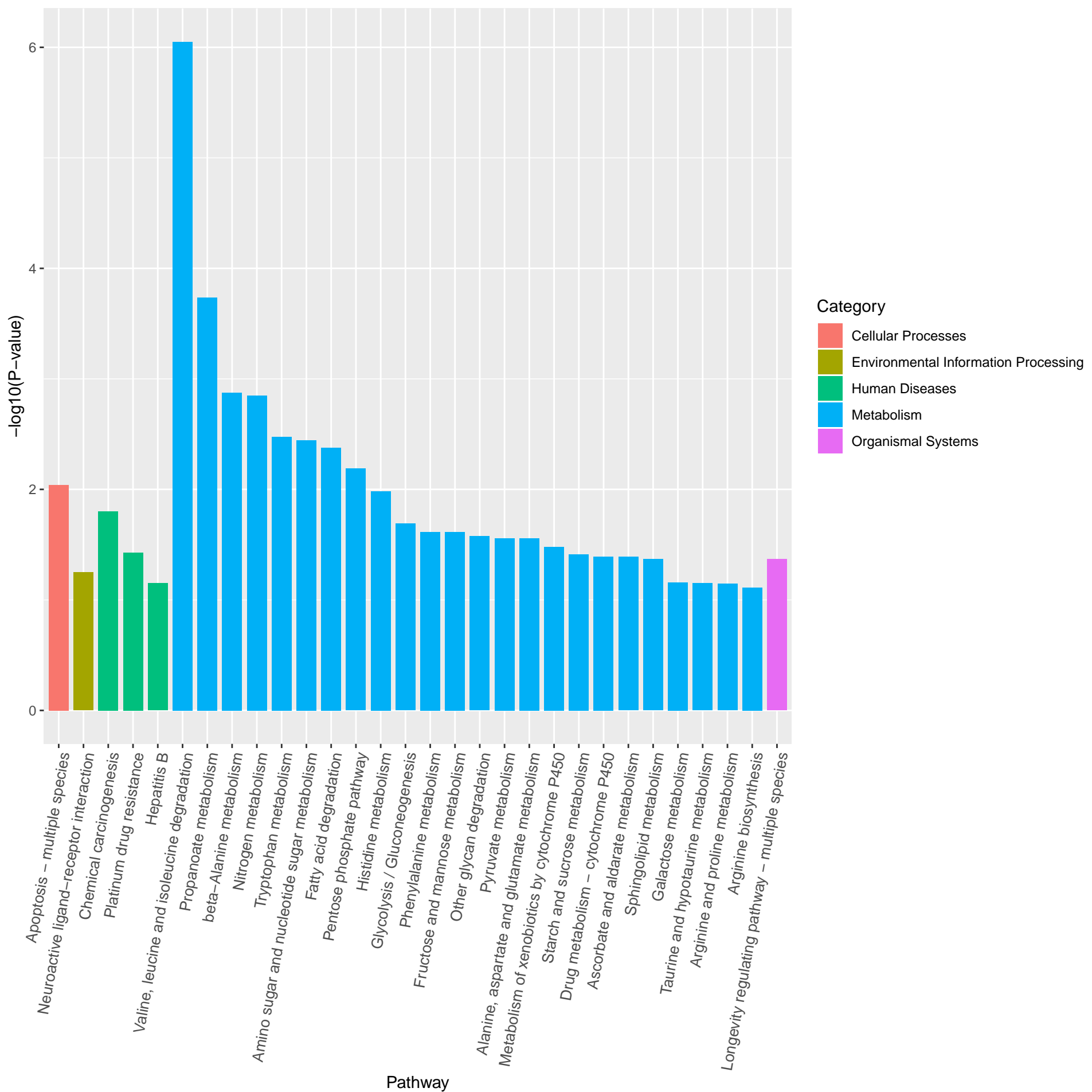

Supplement: Supplementary file 1 [file Data_Sheet_1.ZIP › mRNA/3_Enrichment/C_vs_A/KEGG_enrichment_pvalue_barplot.pdf]

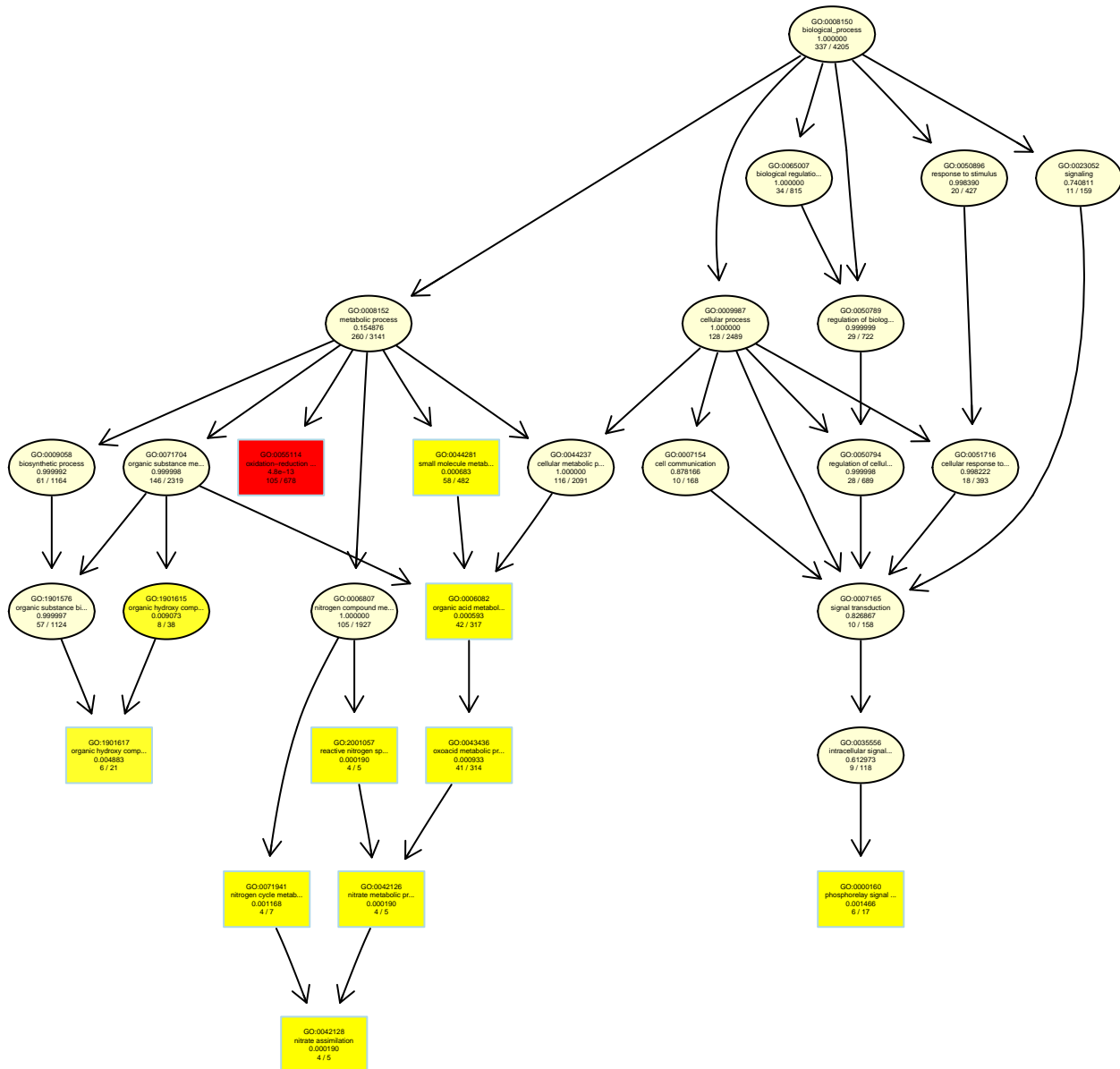

Supplement: Supplementary file 1 [file Data_Sheet_1.ZIP › mRNA/3_Enrichment/C_vs_A/topGO_BP_top10.pdf]

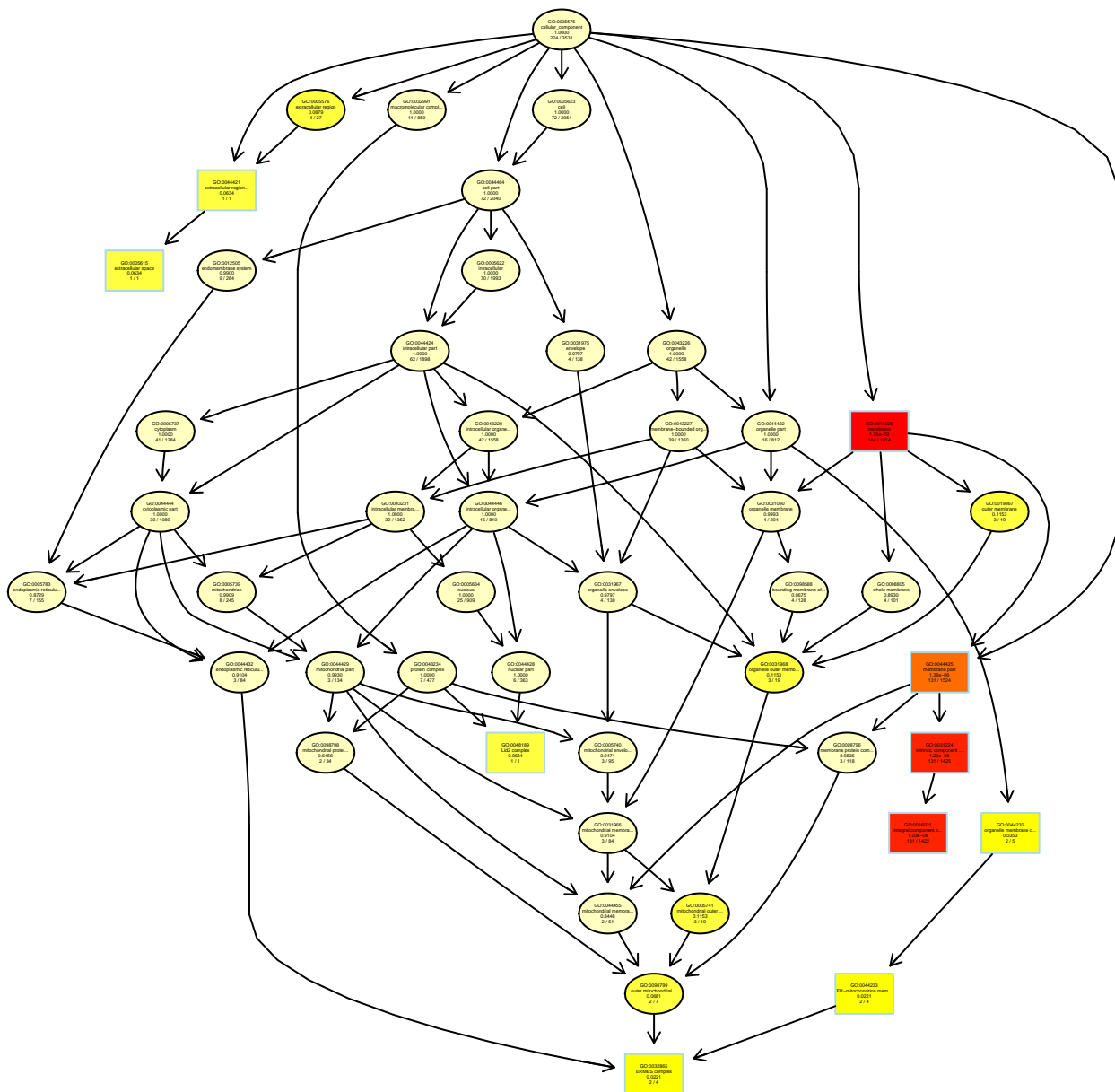

Supplement: Supplementary file 1 [file Data_Sheet_1.ZIP › mRNA/3_Enrichment/C_vs_A/topGO_CC_top10.pdf]

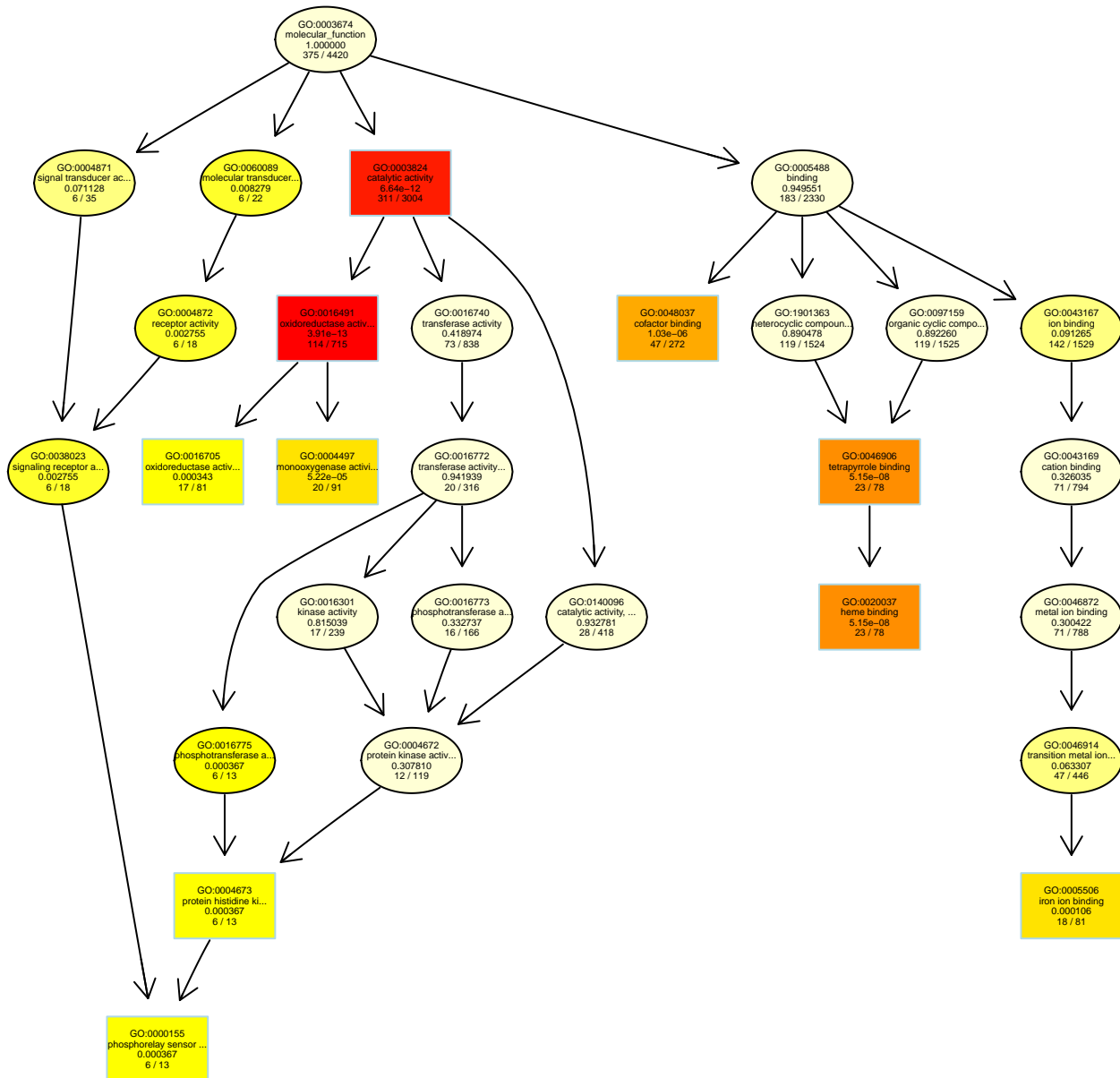

Supplement: Supplementary file 1 [file Data_Sheet_1.ZIP › mRNA/3_Enrichment/C_vs_A/topGO_MF_top10.pdf]

# GO Enrichment

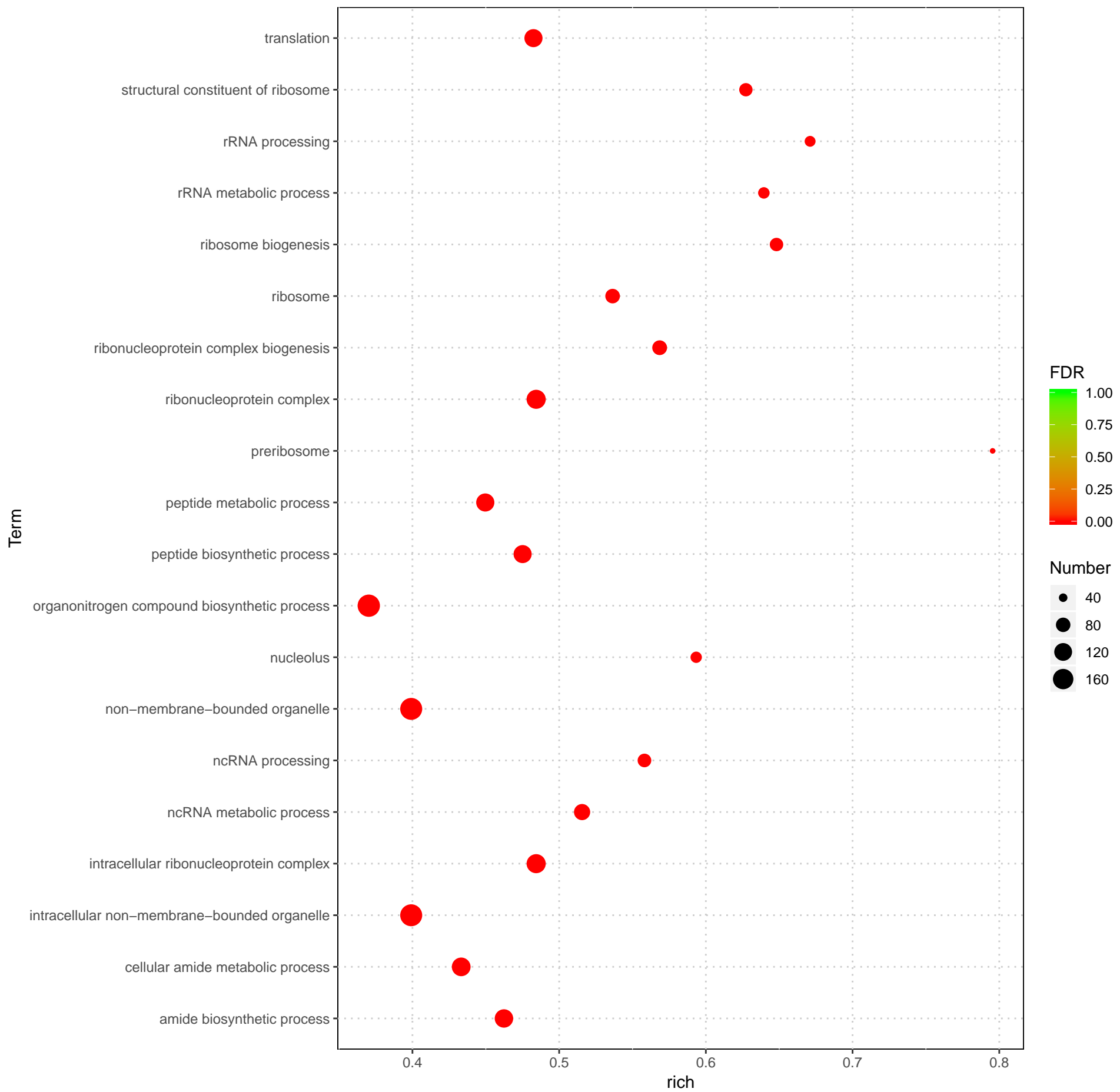

Supplement: Supplementary file 1 [file Data_Sheet_1.ZIP › mRNA/3_Enrichment/D_vs_A/GO.richfactor.pdf]

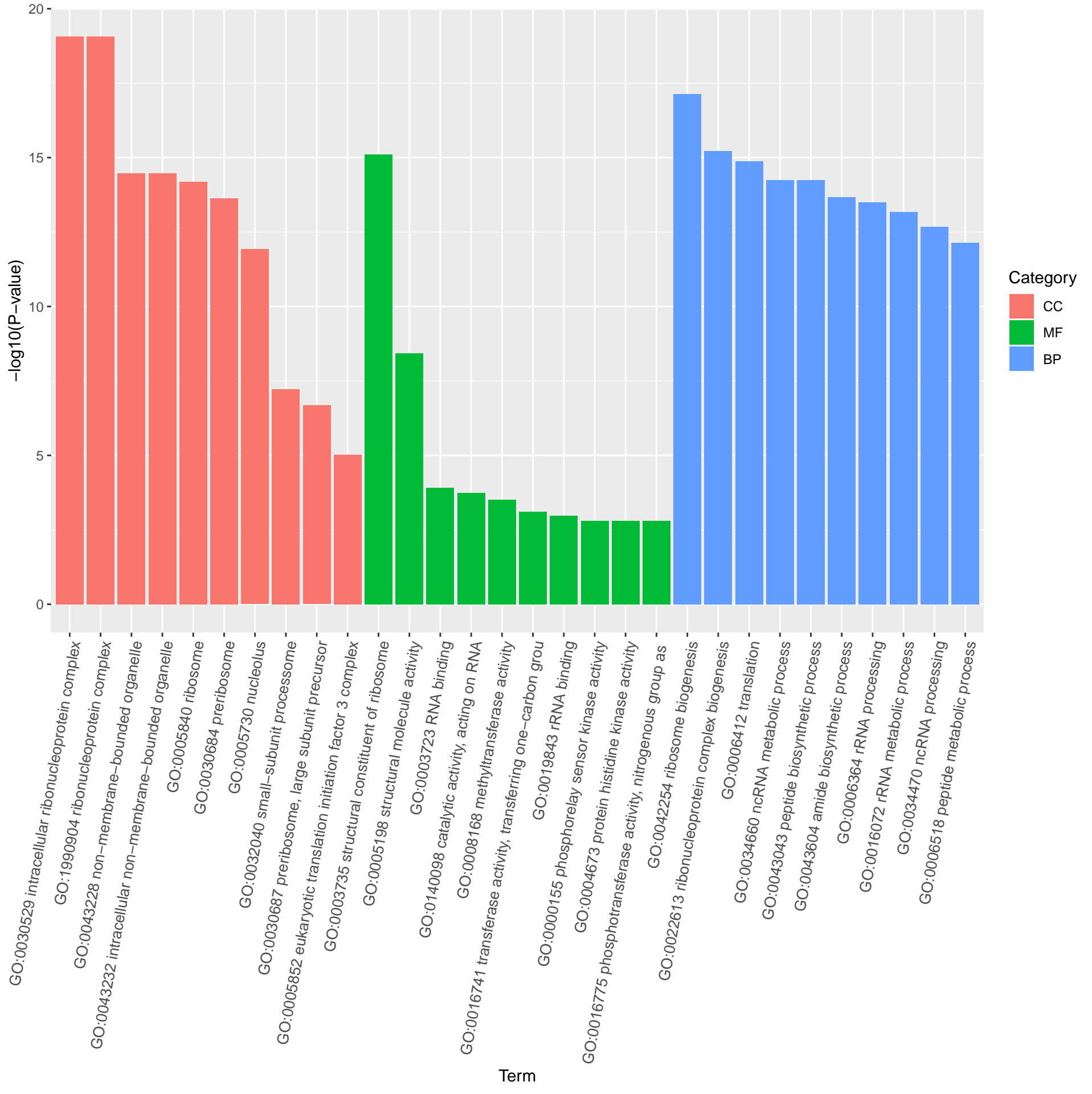

Supplement: Supplementary file 1 [file Data_Sheet_1.ZIP › mRNA/3_Enrichment/D_vs_A/GO_enrichment_pvalue_barplot.pdf]

# KEGG Pathway Enrichment

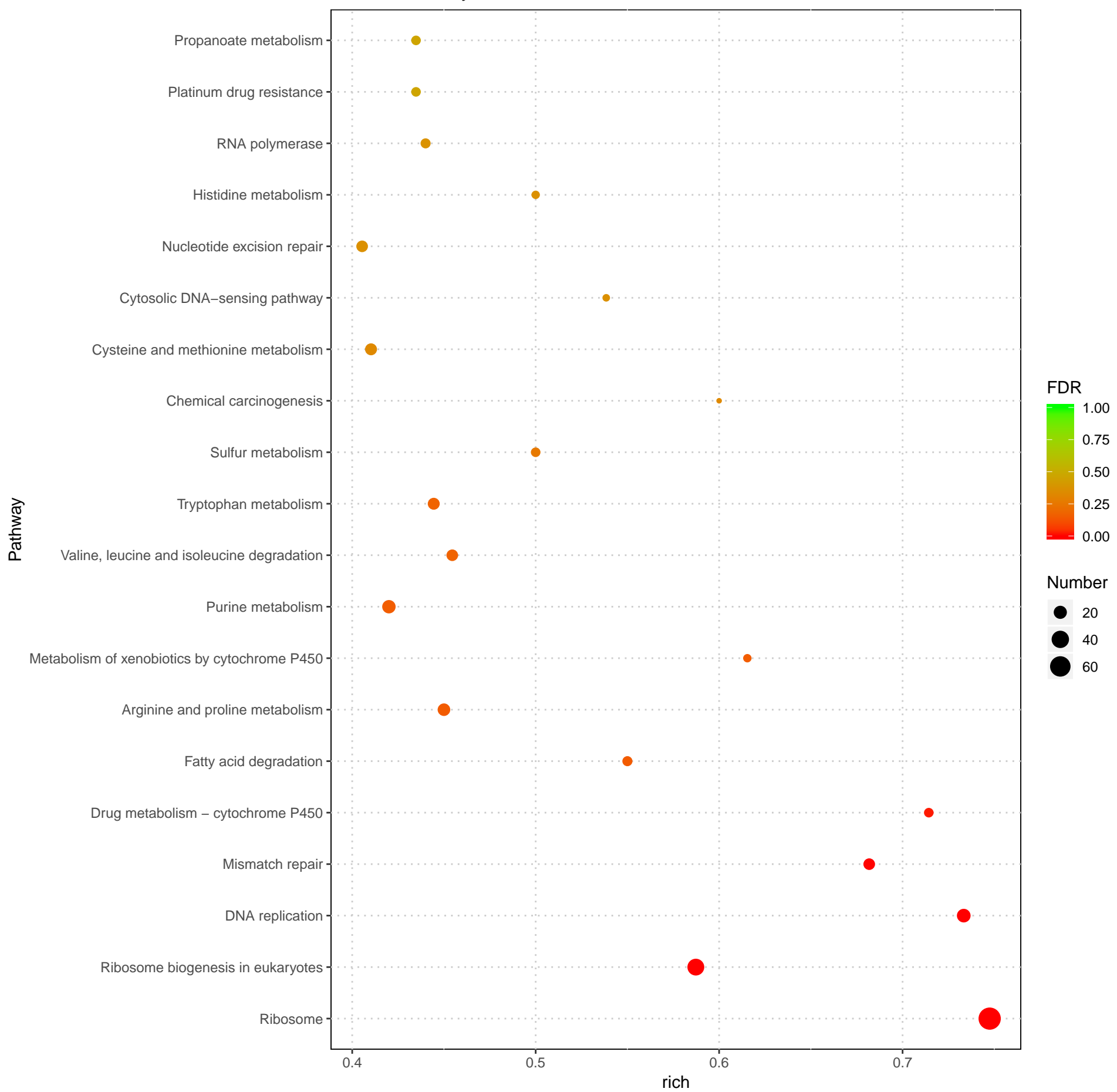

Supplement: Supplementary file 1 [file Data_Sheet_1.ZIP › mRNA/3_Enrichment/D_vs_A/KEGG.richfactor.pdf]

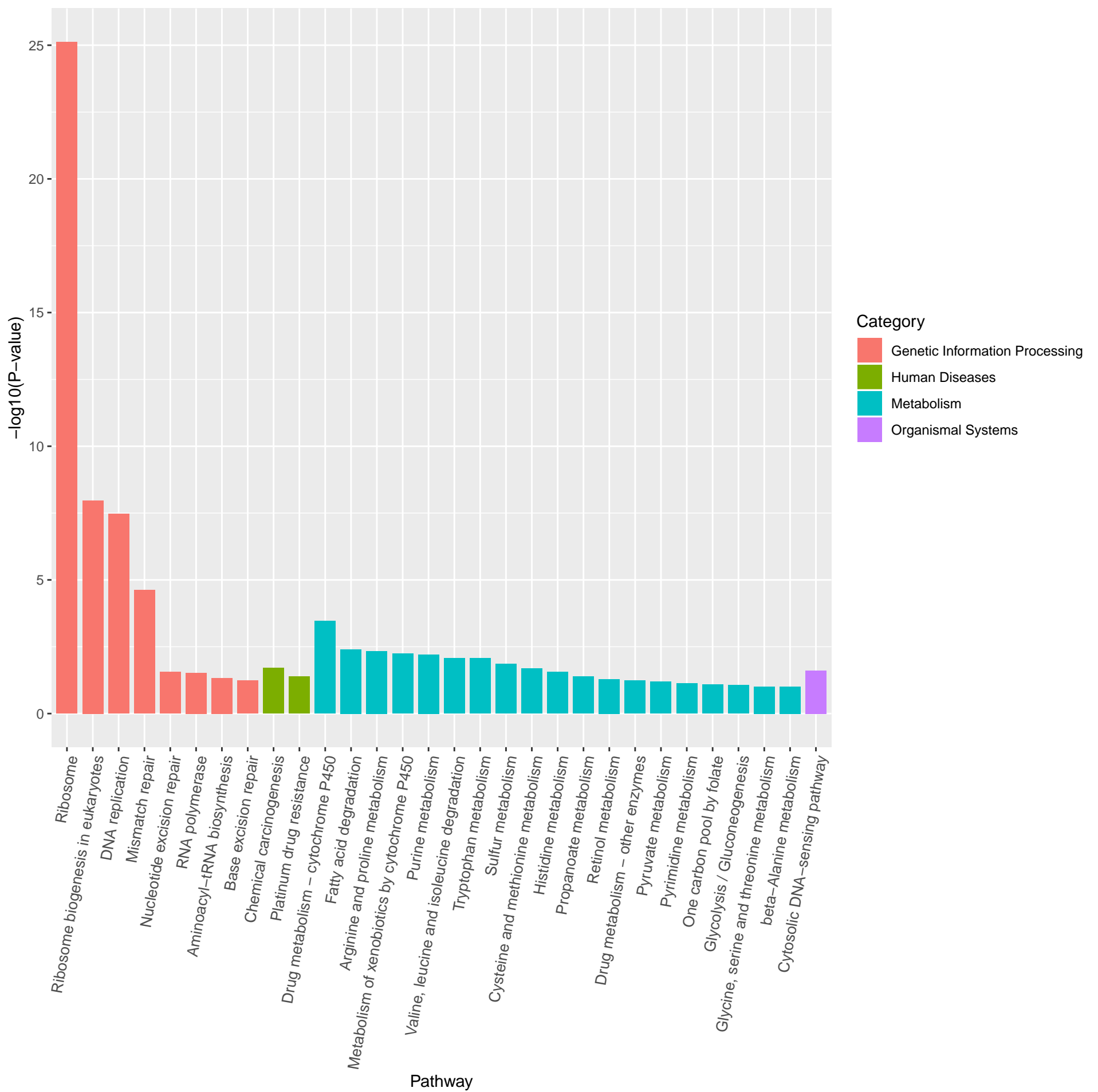

Supplement: Supplementary file 1 [file Data_Sheet_1.ZIP › mRNA/3_Enrichment/D_vs_A/KEGG_enrichment_pvalue_barplot.pdf]

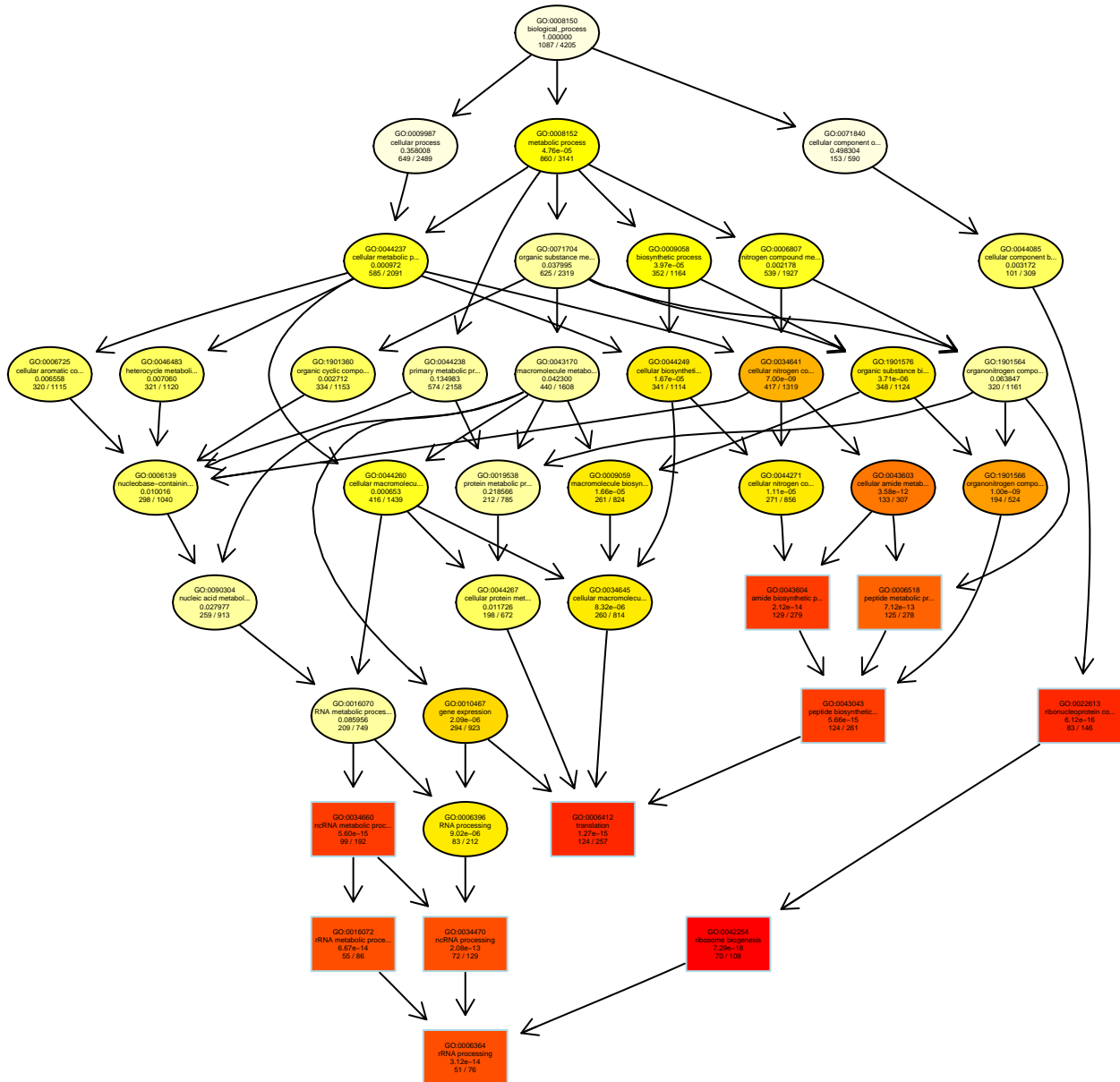

Supplement: Supplementary file 1 [file Data_Sheet_1.ZIP › mRNA/3_Enrichment/D_vs_A/topGO_BP_top10.pdf]

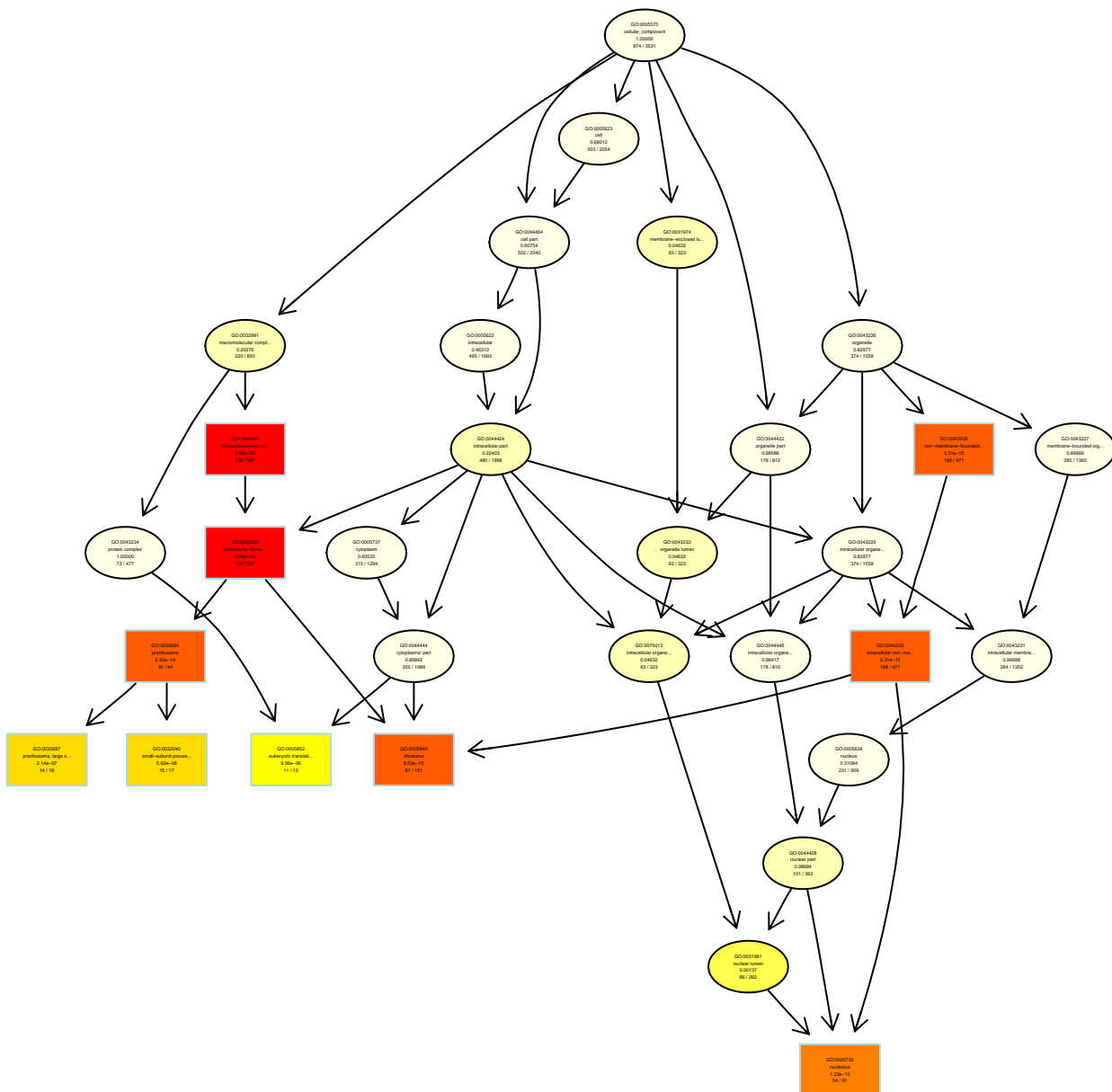

Supplement: Supplementary file 1 [file Data_Sheet_1.ZIP › mRNA/3_Enrichment/D_vs_A/topGO_CC_top10.pdf]
