# Supplementary figures and images for: Functional characterization of CreA, ZnT, and MTase as key regulators of cadmium resistance in Paecilomyces lilacinus (part 1 of 2)
Source: Front Microbiol. 2026 May 14;17:1792636. doi: 10.3389/fmicb.2026.1792636 (PMC13218079; doi:10.3389/fmicb.2026.1792636)

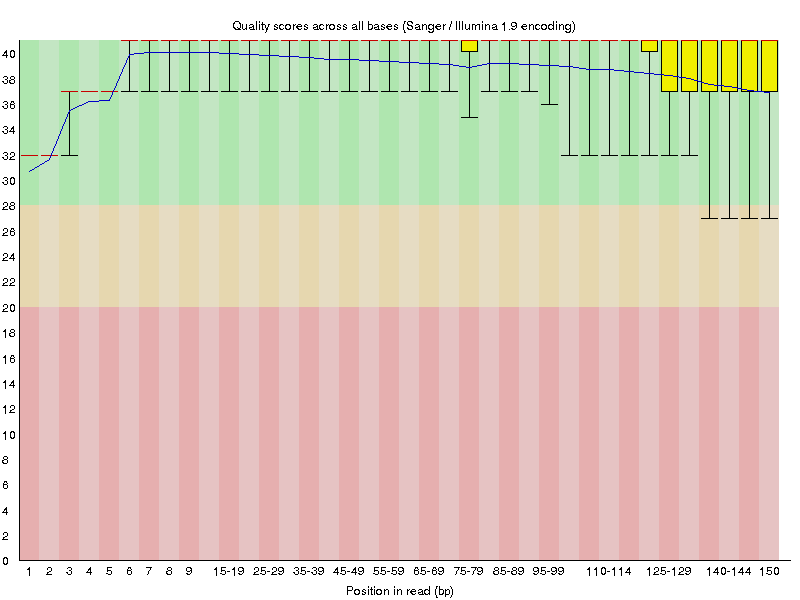

Supplement: Supplementary file 1 [file Data_Sheet_1.ZIP › 02_FastQC/A1_R1_fastqc/per_base_quality.png]

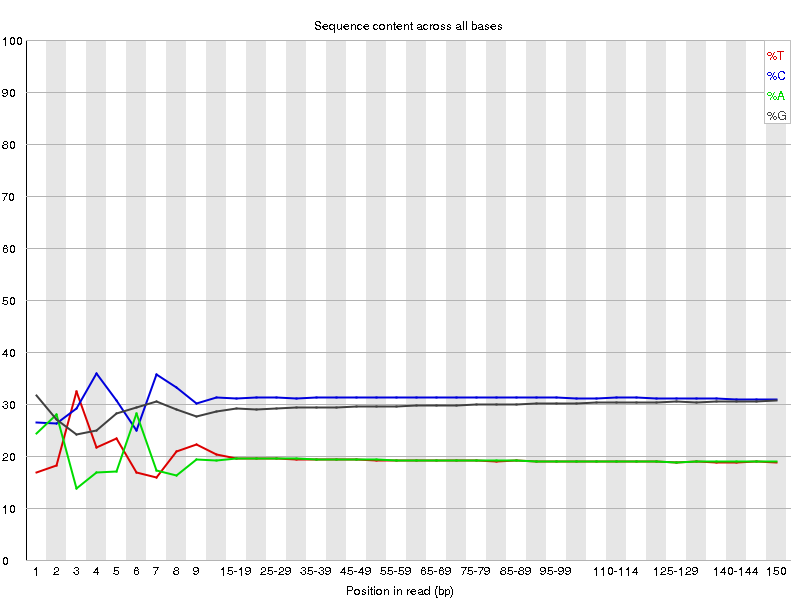

Supplement: Supplementary file 1 [file Data_Sheet_1.ZIP › 02_FastQC/A1_R1_fastqc/per_base_sequence_content.png]

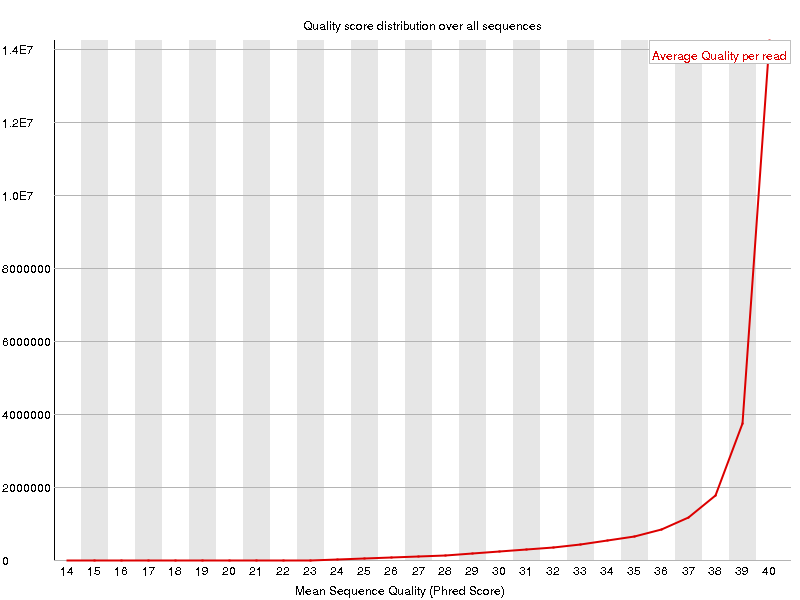

Supplement: Supplementary file 1 [file Data_Sheet_1.ZIP › 02_FastQC/A1_R1_fastqc/per_sequence_quality.png]

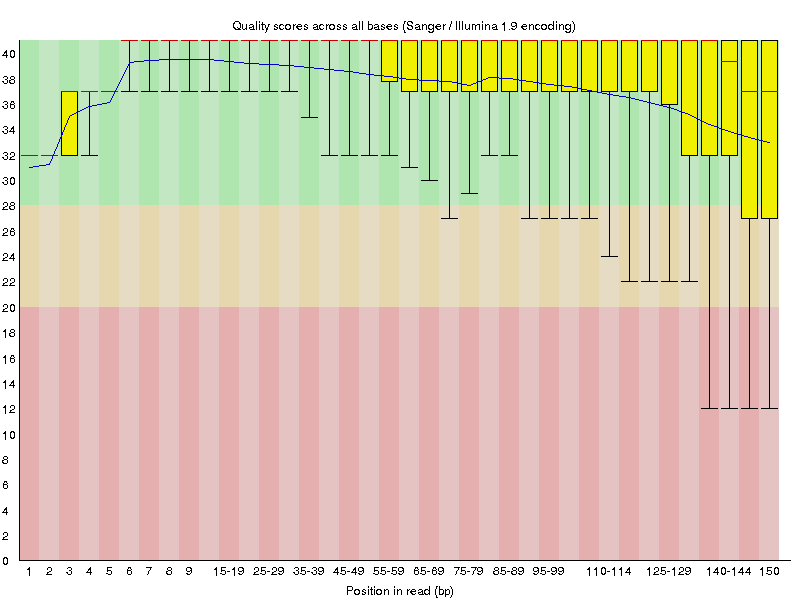

Supplement: Supplementary file 1 [file Data_Sheet_1.ZIP › 02_FastQC/A1_R2_fastqc/per_base_quality.png]

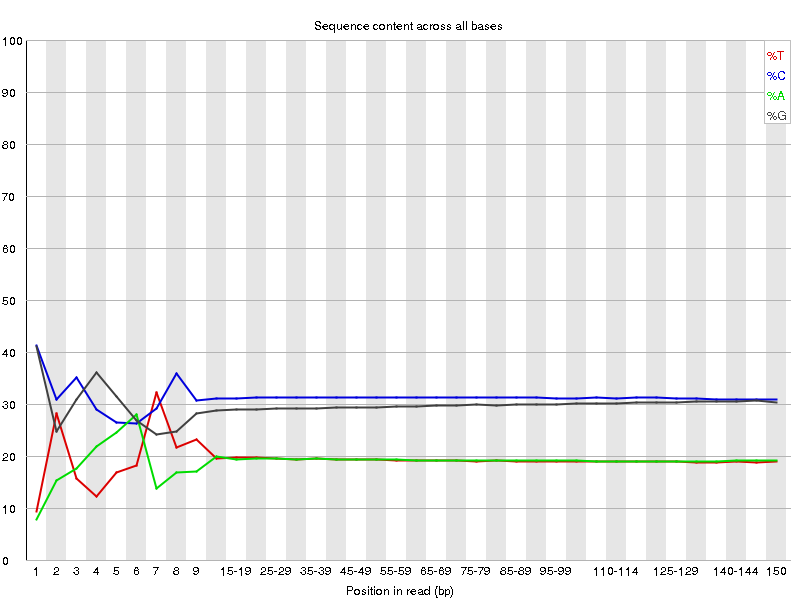

Supplement: Supplementary file 1 [file Data_Sheet_1.ZIP › 02_FastQC/A1_R2_fastqc/per_base_sequence_content.png]

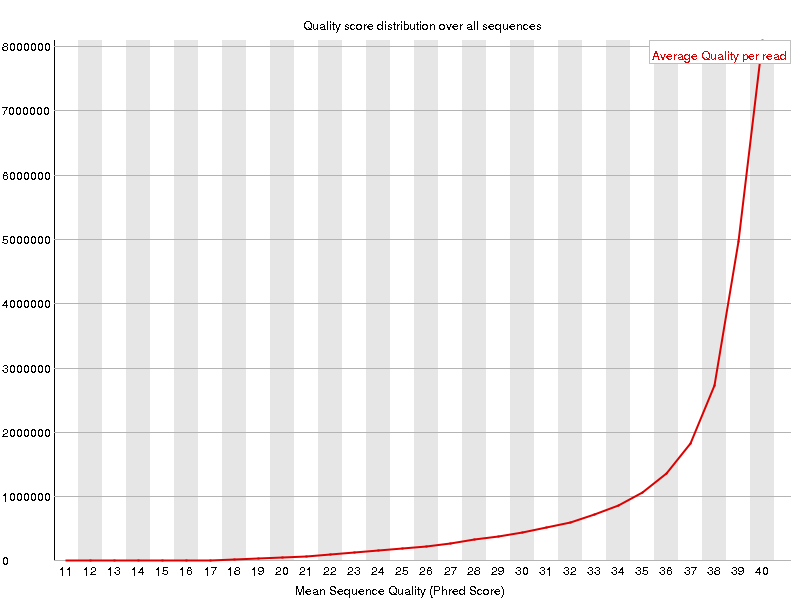

Supplement: Supplementary file 1 [file Data_Sheet_1.ZIP › 02_FastQC/A1_R2_fastqc/per_sequence_quality.png]

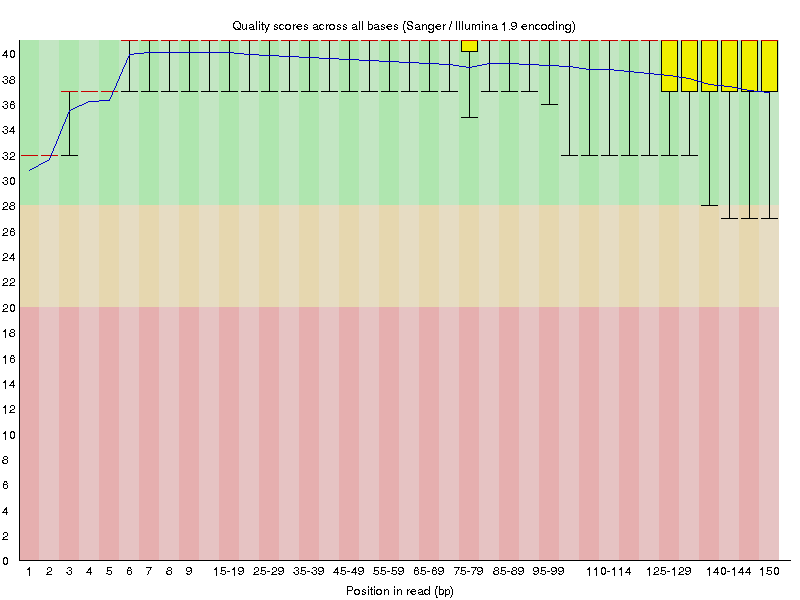

Supplement: Supplementary file 1 [file Data_Sheet_1.ZIP › 02_FastQC/A2_R1_fastqc/per_base_quality.png]

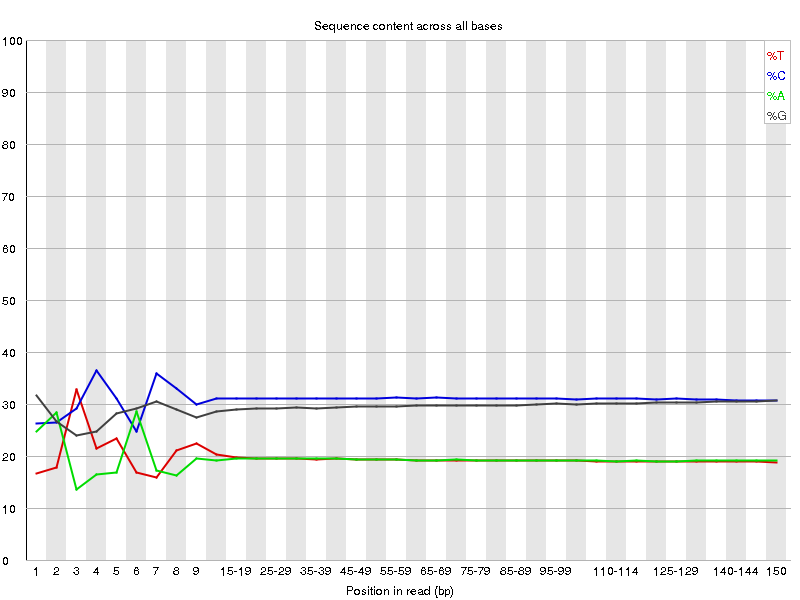

Supplement: Supplementary file 1 [file Data_Sheet_1.ZIP › 02_FastQC/A2_R1_fastqc/per_base_sequence_content.png]

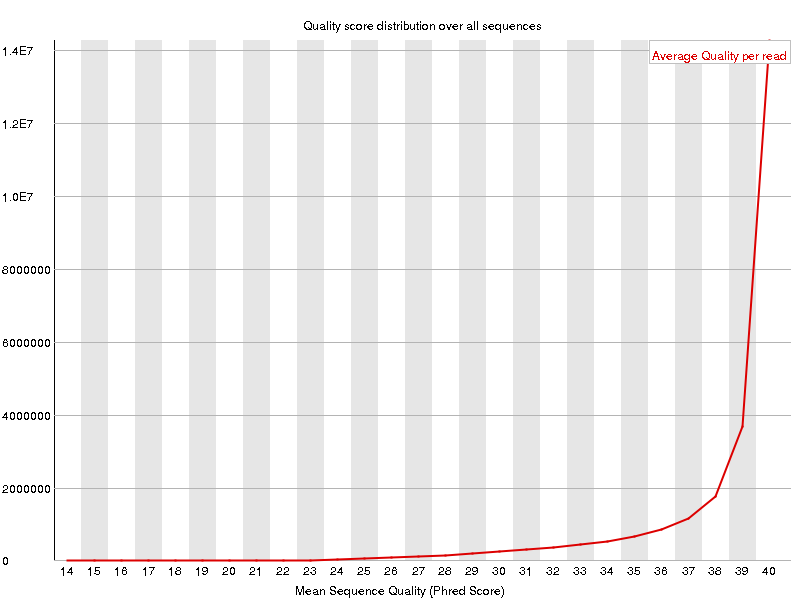

Supplement: Supplementary file 1 [file Data_Sheet_1.ZIP › 02_FastQC/A2_R1_fastqc/per_sequence_quality.png]

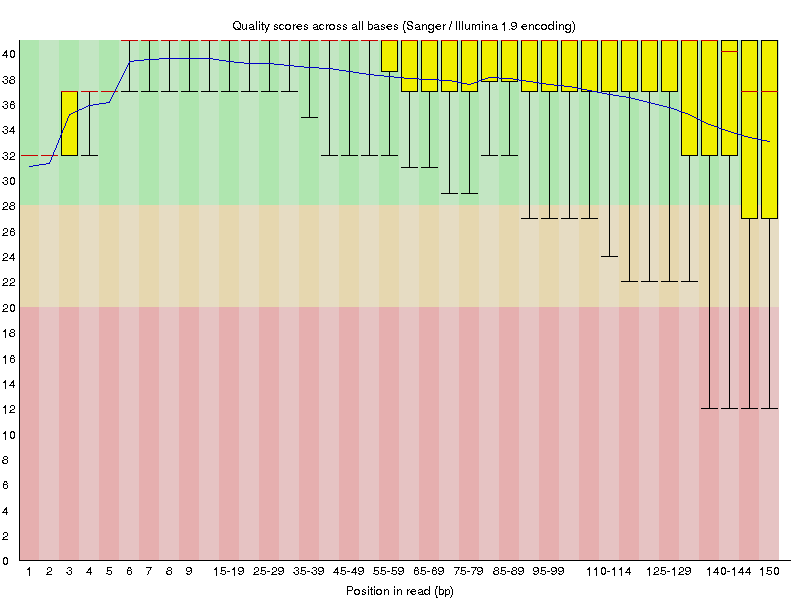

Supplement: Supplementary file 1 [file Data_Sheet_1.ZIP › 02_FastQC/A2_R2_fastqc/per_base_quality.png]

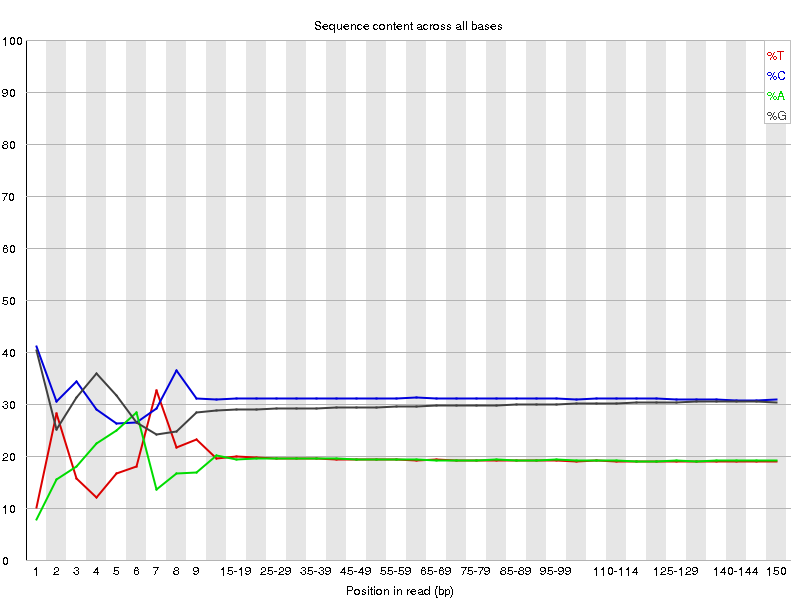

Supplement: Supplementary file 1 [file Data_Sheet_1.ZIP › 02_FastQC/A2_R2_fastqc/per_base_sequence_content.png]

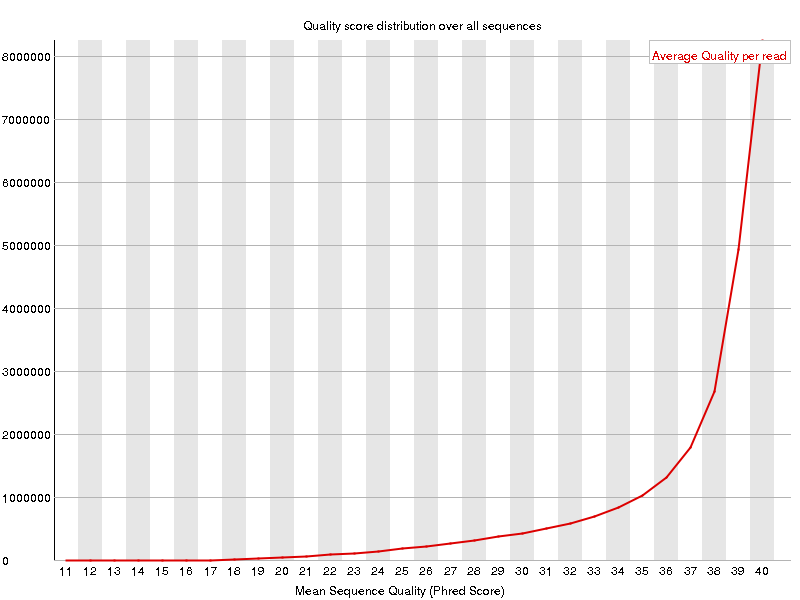

Supplement: Supplementary file 1 [file Data_Sheet_1.ZIP › 02_FastQC/A2_R2_fastqc/per_sequence_quality.png]

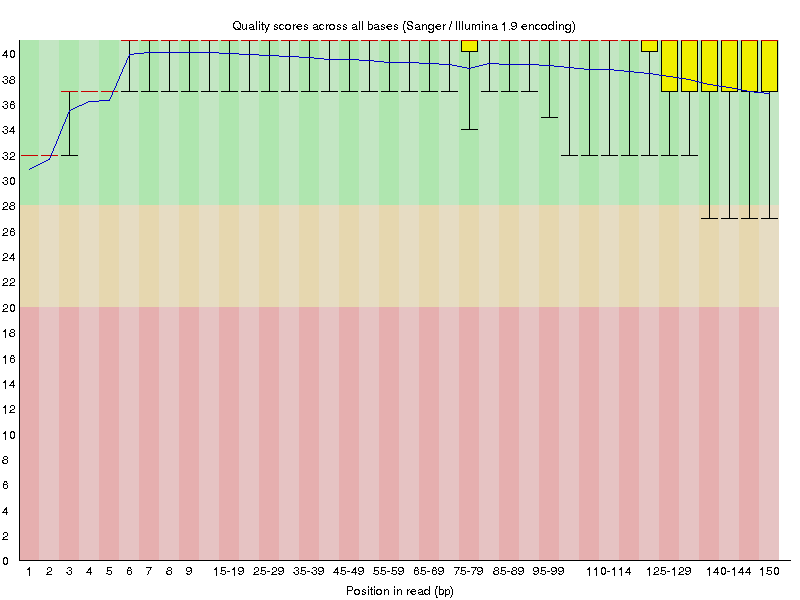

Supplement: Supplementary file 1 [file Data_Sheet_1.ZIP › 02_FastQC/A3_R1_fastqc/per_base_quality.png]

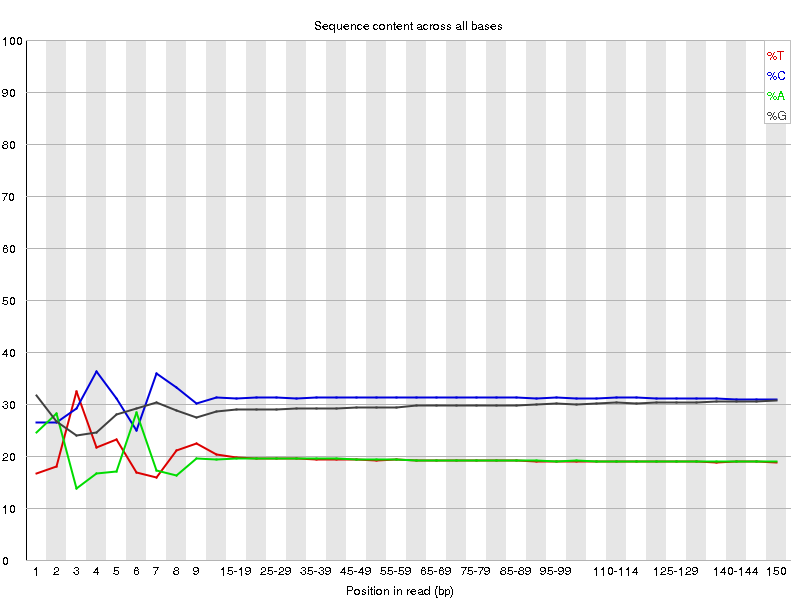

Supplement: Supplementary file 1 [file Data_Sheet_1.ZIP › 02_FastQC/A3_R1_fastqc/per_base_sequence_content.png]

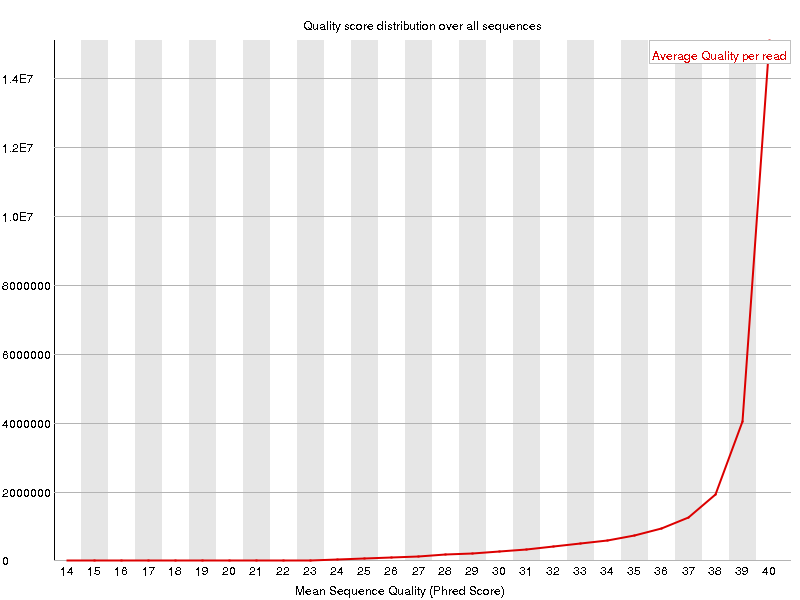

Supplement: Supplementary file 1 [file Data_Sheet_1.ZIP › 02_FastQC/A3_R1_fastqc/per_sequence_quality.png]

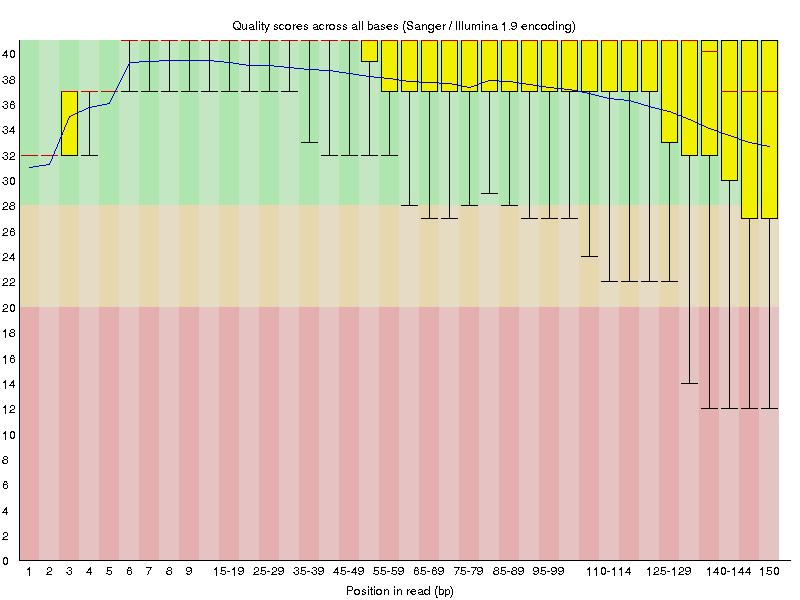

Supplement: Supplementary file 1 [file Data_Sheet_1.ZIP › 02_FastQC/A3_R2_fastqc/per_base_quality.png]

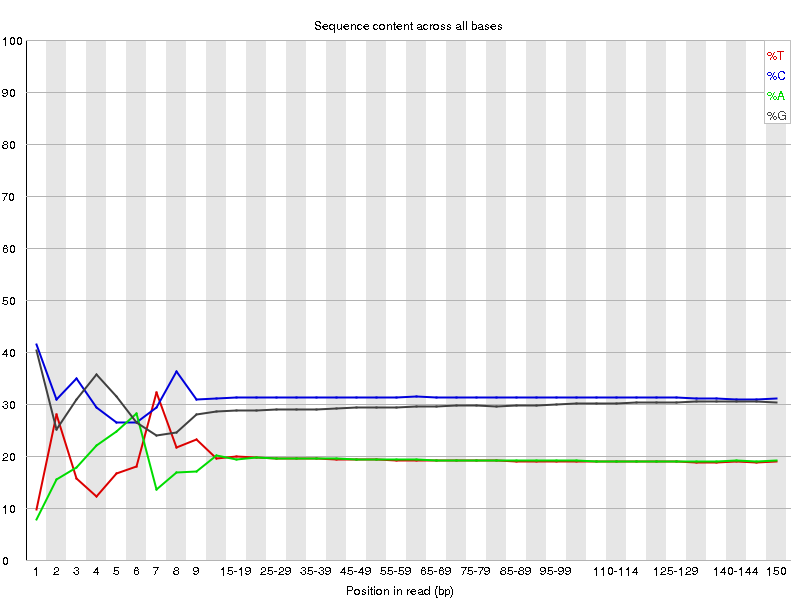

Supplement: Supplementary file 1 [file Data_Sheet_1.ZIP › 02_FastQC/A3_R2_fastqc/per_base_sequence_content.png]

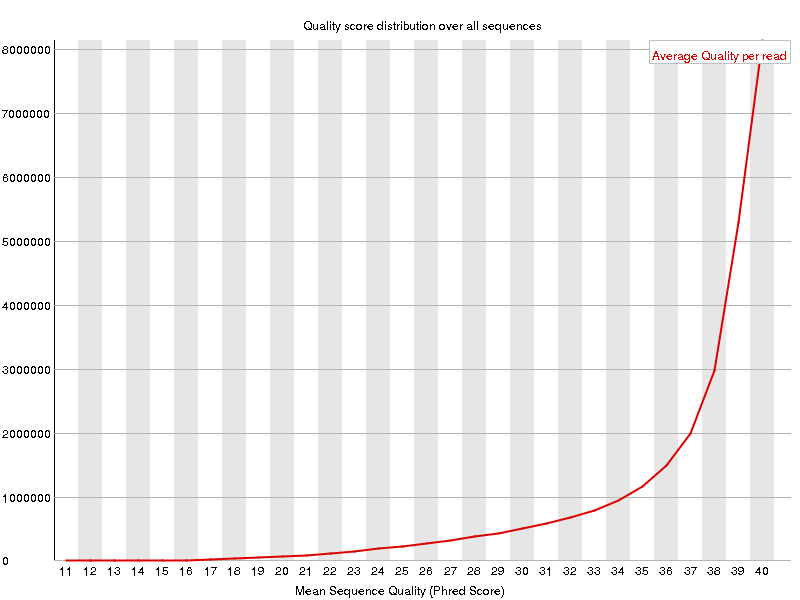

Supplement: Supplementary file 1 [file Data_Sheet_1.ZIP › 02_FastQC/A3_R2_fastqc/per_sequence_quality.png]

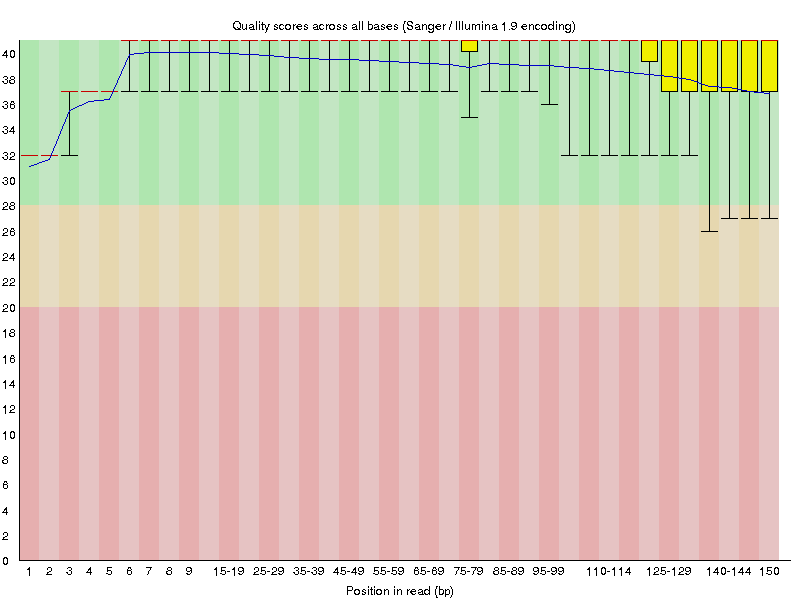

Supplement: Supplementary file 1 [file Data_Sheet_1.ZIP › 02_FastQC/B1_R1_fastqc/per_base_quality.png]

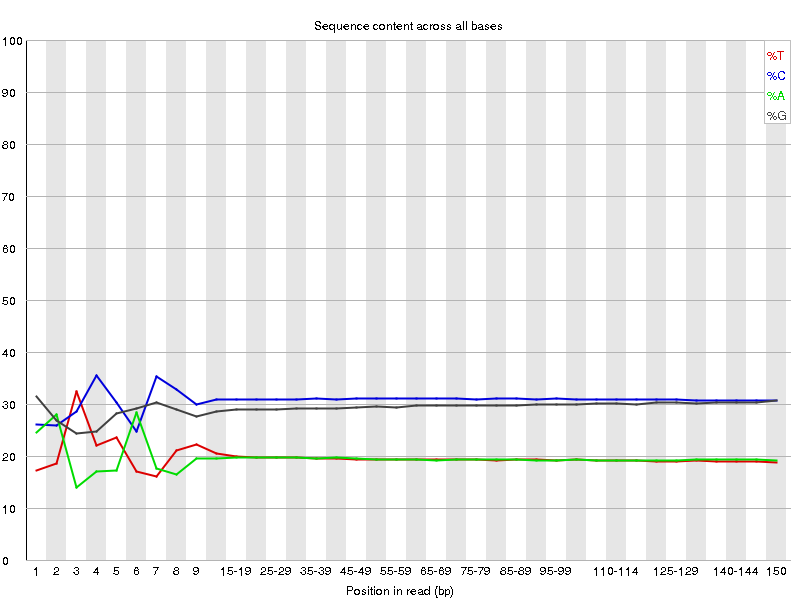

Supplement: Supplementary file 1 [file Data_Sheet_1.ZIP › 02_FastQC/B1_R1_fastqc/per_base_sequence_content.png]

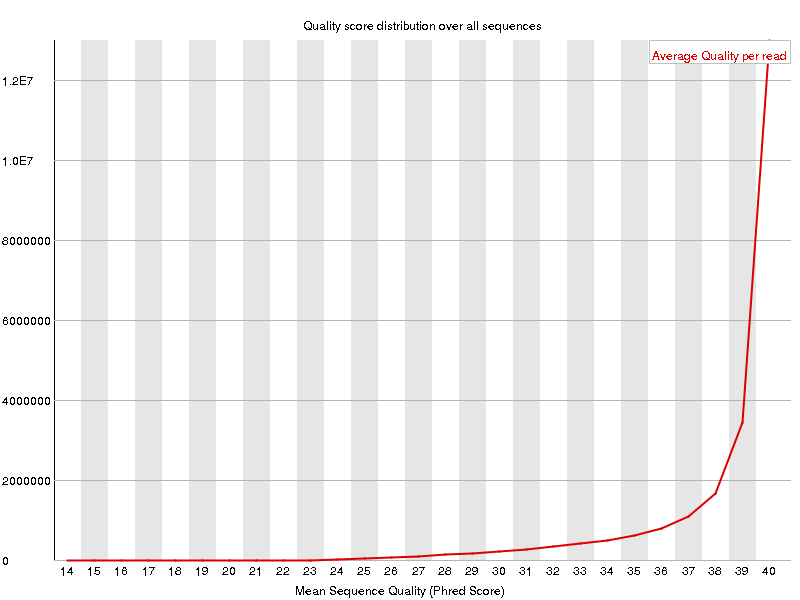

Supplement: Supplementary file 1 [file Data_Sheet_1.ZIP › 02_FastQC/B1_R1_fastqc/per_sequence_quality.png]

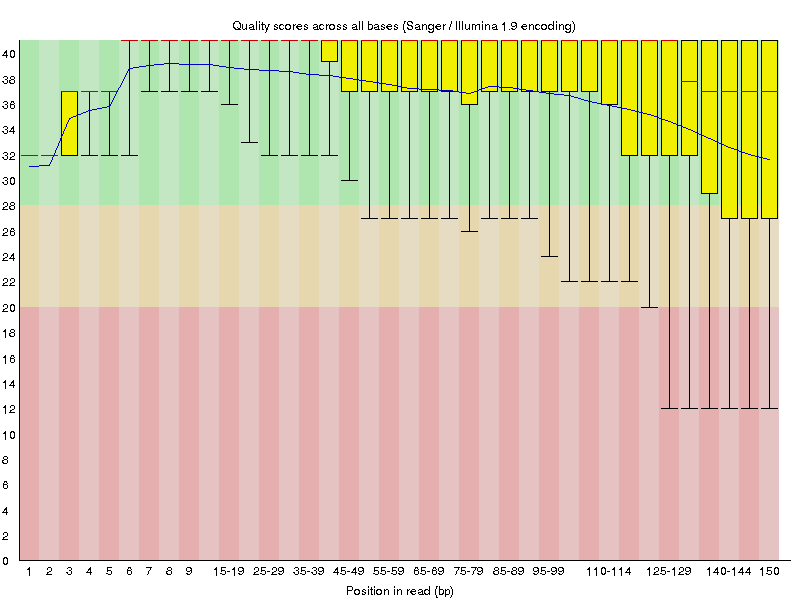

Supplement: Supplementary file 1 [file Data_Sheet_1.ZIP › 02_FastQC/B1_R2_fastqc/per_base_quality.png]

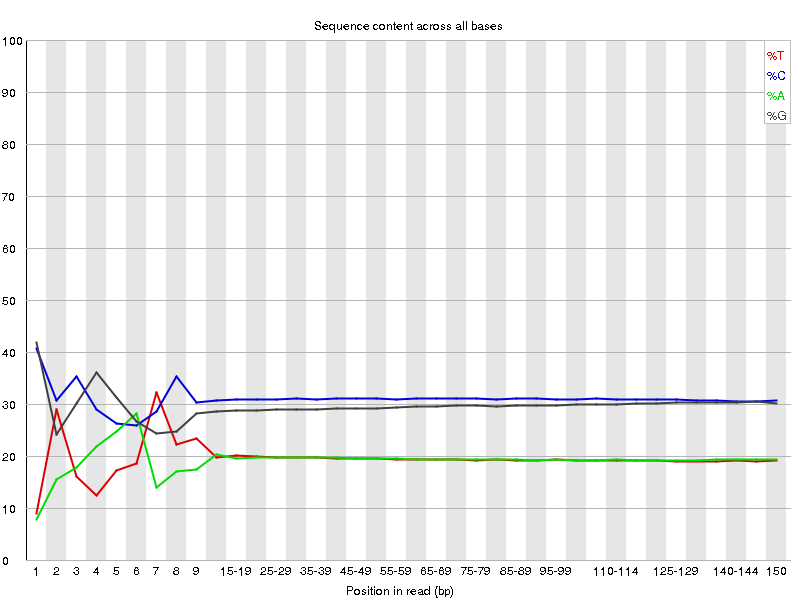

Supplement: Supplementary file 1 [file Data_Sheet_1.ZIP › 02_FastQC/B1_R2_fastqc/per_base_sequence_content.png]

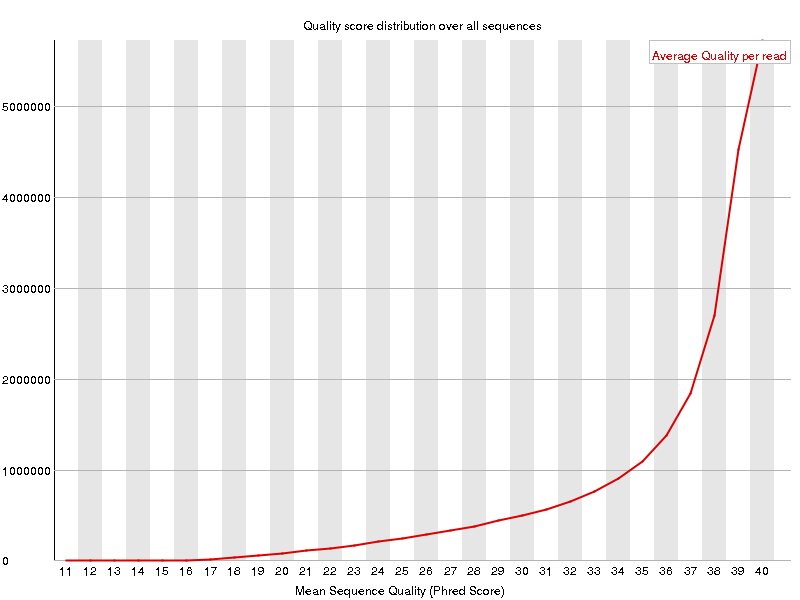

Supplement: Supplementary file 1 [file Data_Sheet_1.ZIP › 02_FastQC/B1_R2_fastqc/per_sequence_quality.png]

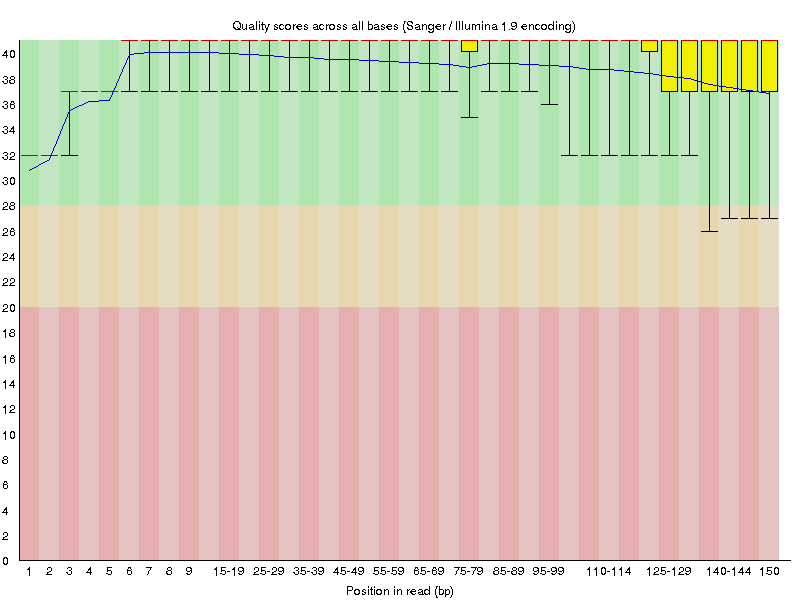

Supplement: Supplementary file 1 [file Data_Sheet_1.ZIP › 02_FastQC/B2_R1_fastqc/per_base_quality.png]

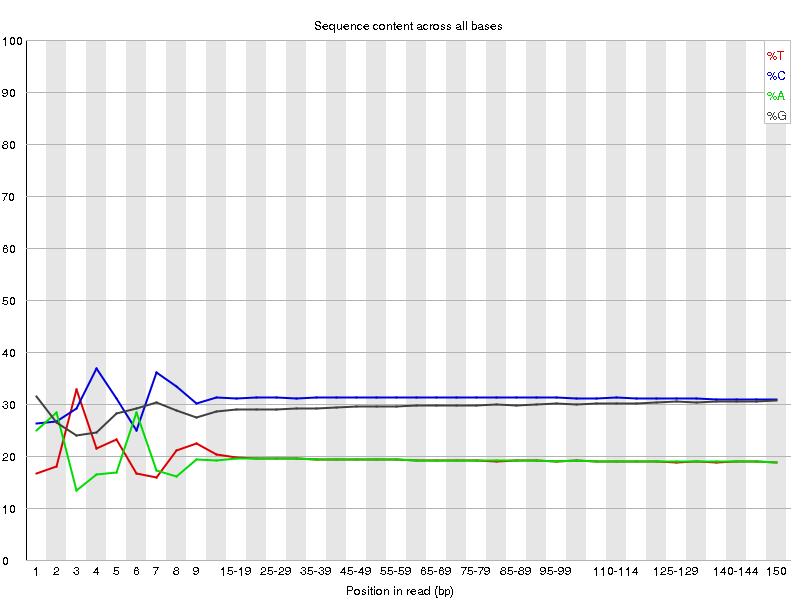

Supplement: Supplementary file 1 [file Data_Sheet_1.ZIP › 02_FastQC/B2_R1_fastqc/per_base_sequence_content.png]

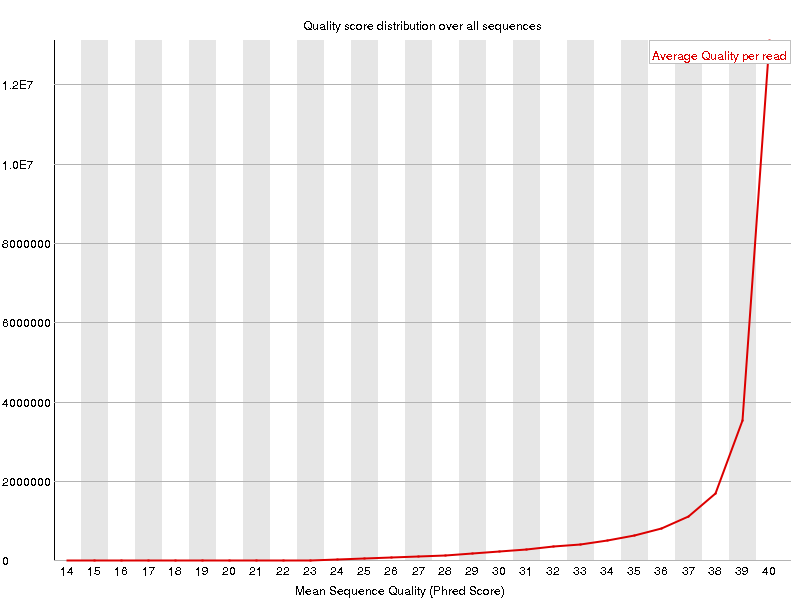

Supplement: Supplementary file 1 [file Data_Sheet_1.ZIP › 02_FastQC/B2_R1_fastqc/per_sequence_quality.png]

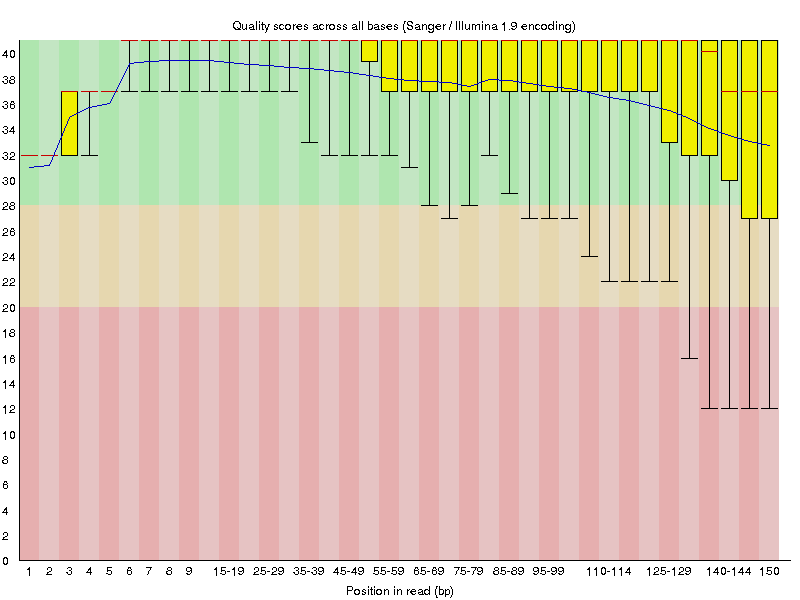

Supplement: Supplementary file 1 [file Data_Sheet_1.ZIP › 02_FastQC/B2_R2_fastqc/per_base_quality.png]

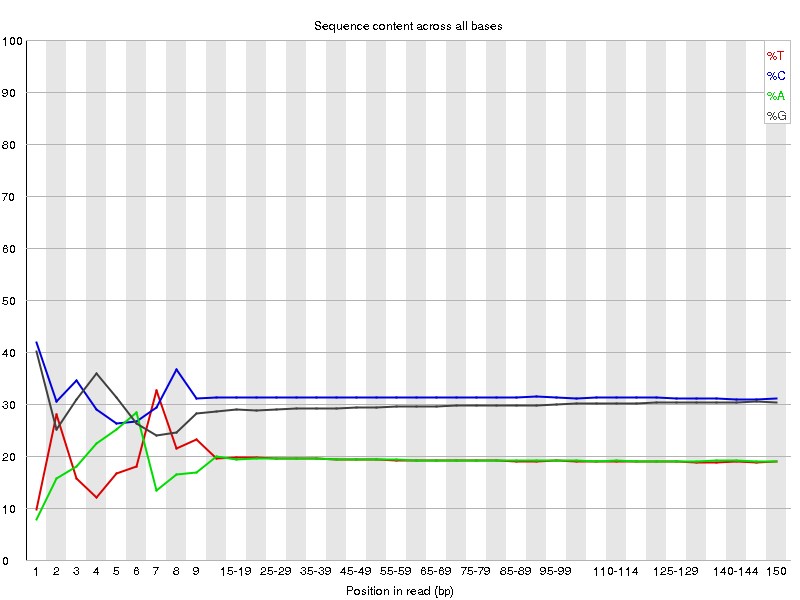

Supplement: Supplementary file 1 [file Data_Sheet_1.ZIP › 02_FastQC/B2_R2_fastqc/per_base_sequence_content.png]

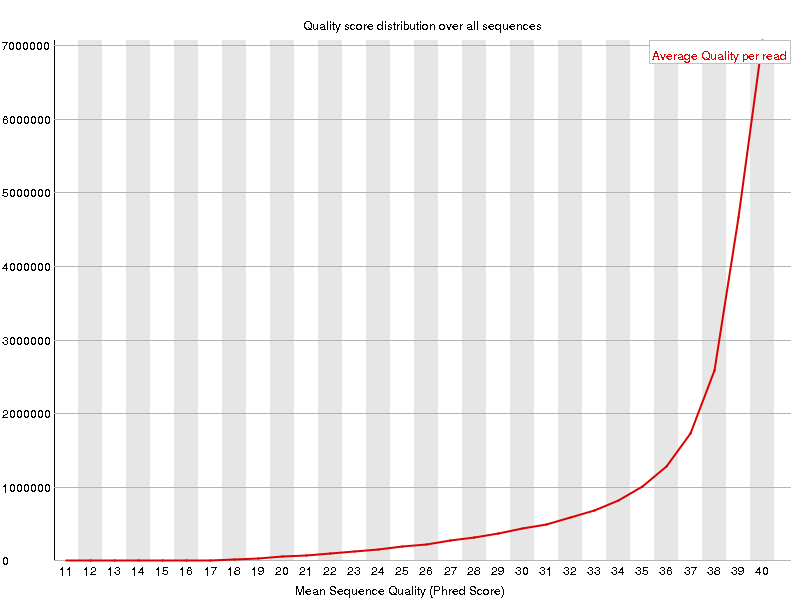

Supplement: Supplementary file 1 [file Data_Sheet_1.ZIP › 02_FastQC/B2_R2_fastqc/per_sequence_quality.png]

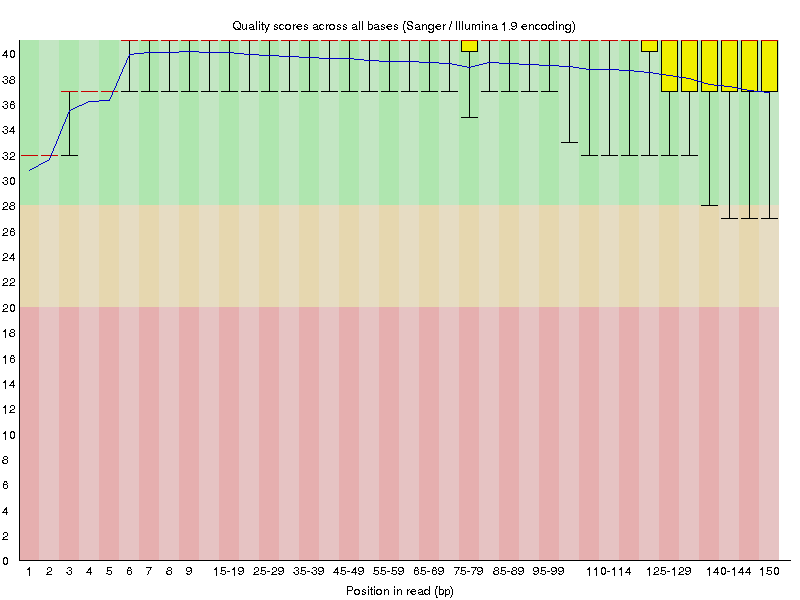

Supplement: Supplementary file 1 [file Data_Sheet_1.ZIP › 02_FastQC/B3_R1_fastqc/per_base_quality.png]

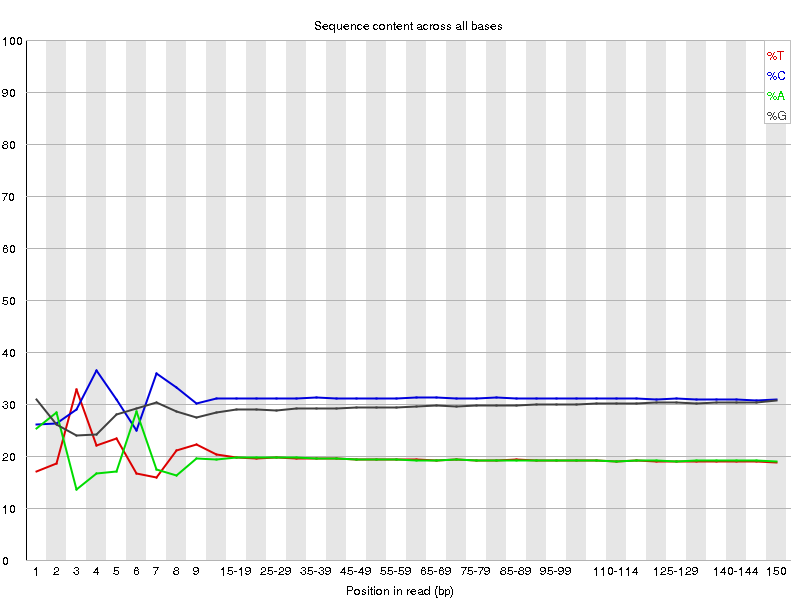

Supplement: Supplementary file 1 [file Data_Sheet_1.ZIP › 02_FastQC/B3_R1_fastqc/per_base_sequence_content.png]

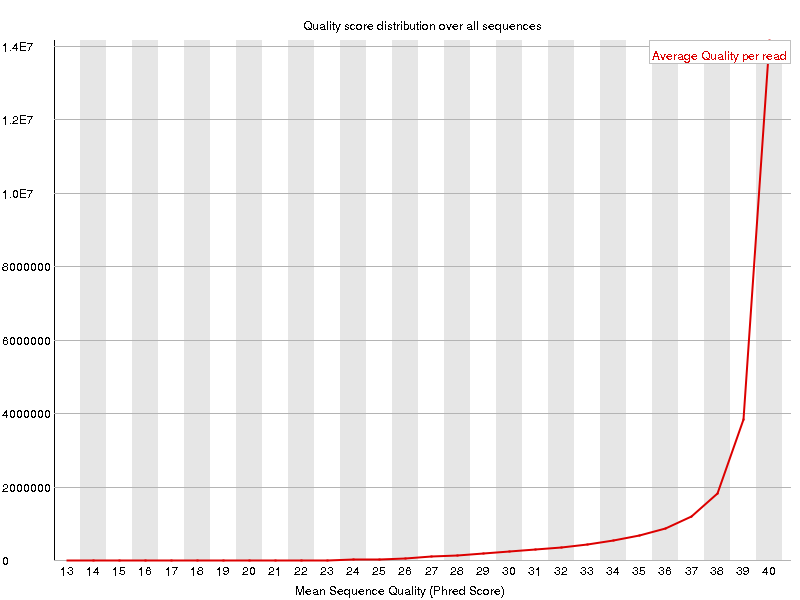

Supplement: Supplementary file 1 [file Data_Sheet_1.ZIP › 02_FastQC/B3_R1_fastqc/per_sequence_quality.png]

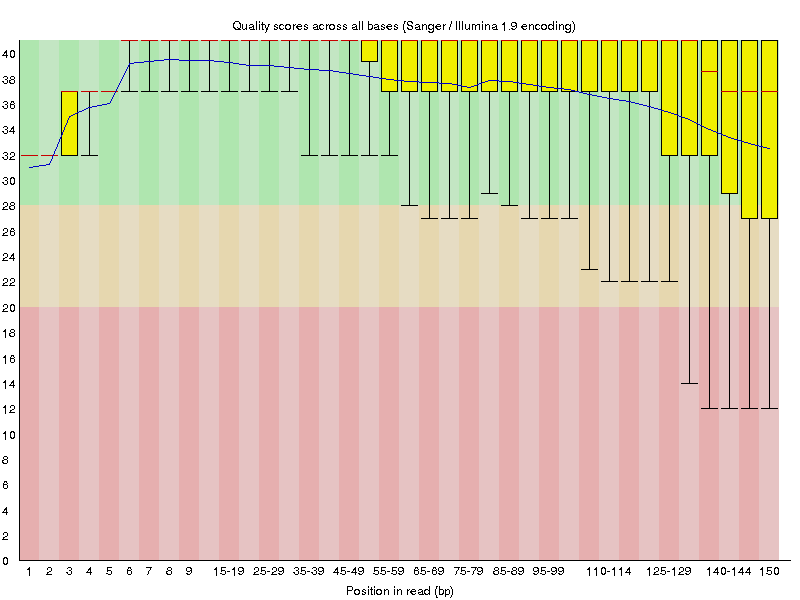

Supplement: Supplementary file 1 [file Data_Sheet_1.ZIP › 02_FastQC/B3_R2_fastqc/per_base_quality.png]

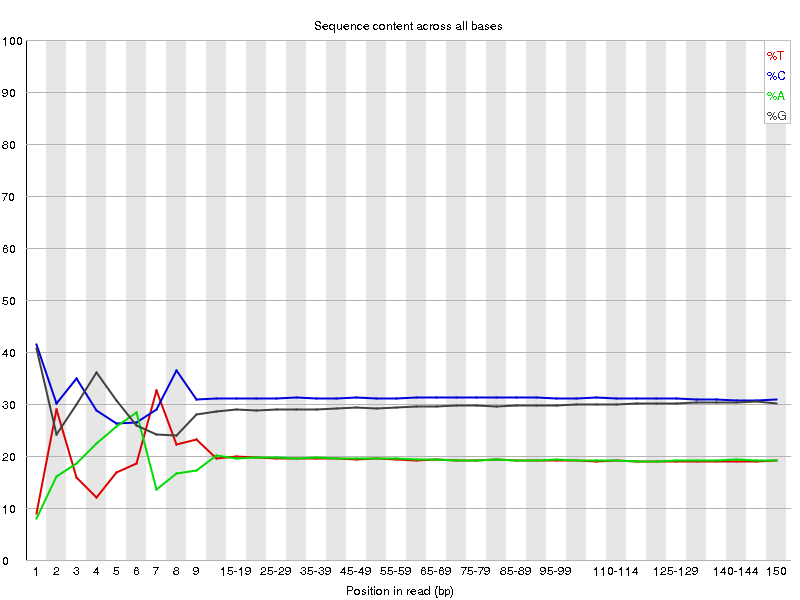

Supplement: Supplementary file 1 [file Data_Sheet_1.ZIP › 02_FastQC/B3_R2_fastqc/per_base_sequence_content.png]

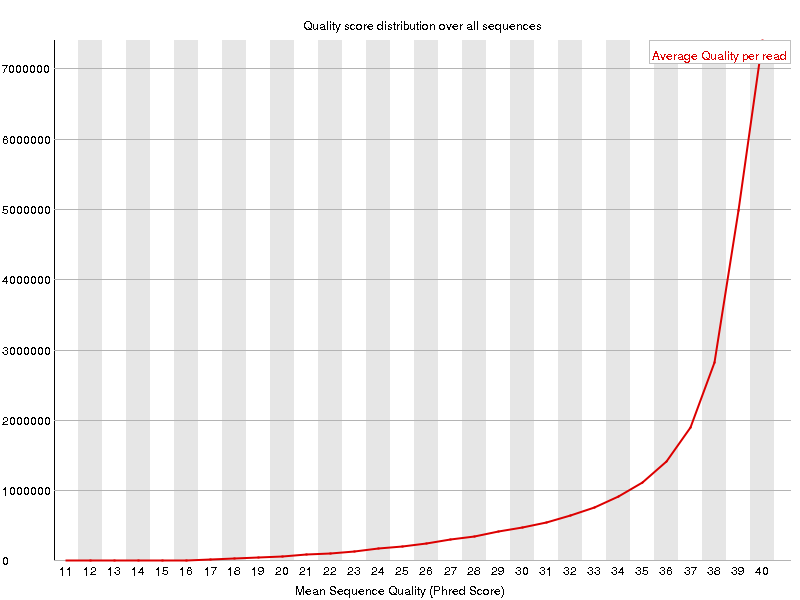

Supplement: Supplementary file 1 [file Data_Sheet_1.ZIP › 02_FastQC/B3_R2_fastqc/per_sequence_quality.png]

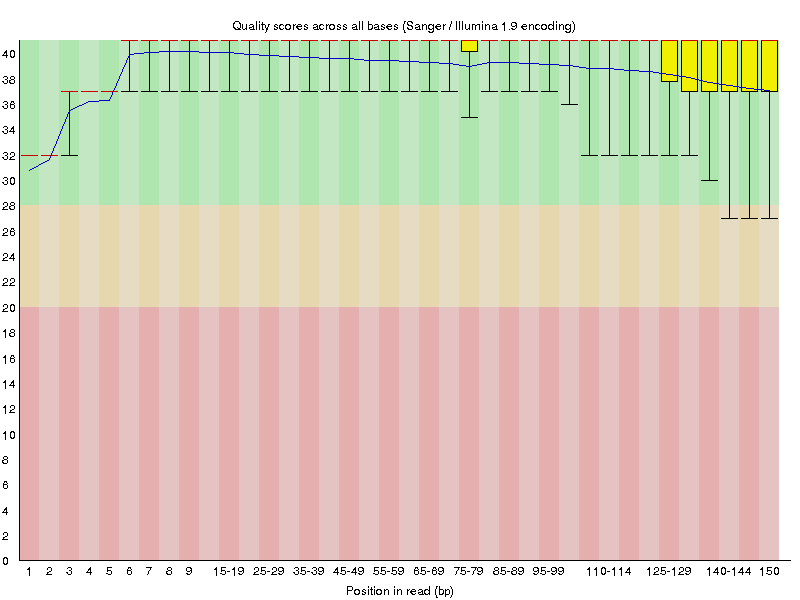

Supplement: Supplementary file 1 [file Data_Sheet_1.ZIP › 02_FastQC/C1_R1_fastqc/per_base_quality.png]

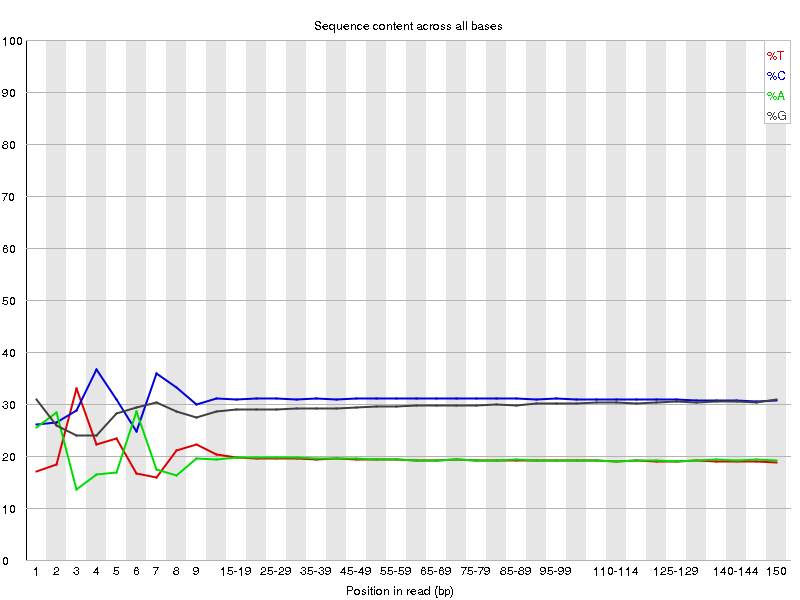

Supplement: Supplementary file 1 [file Data_Sheet_1.ZIP › 02_FastQC/C1_R1_fastqc/per_base_sequence_content.png]

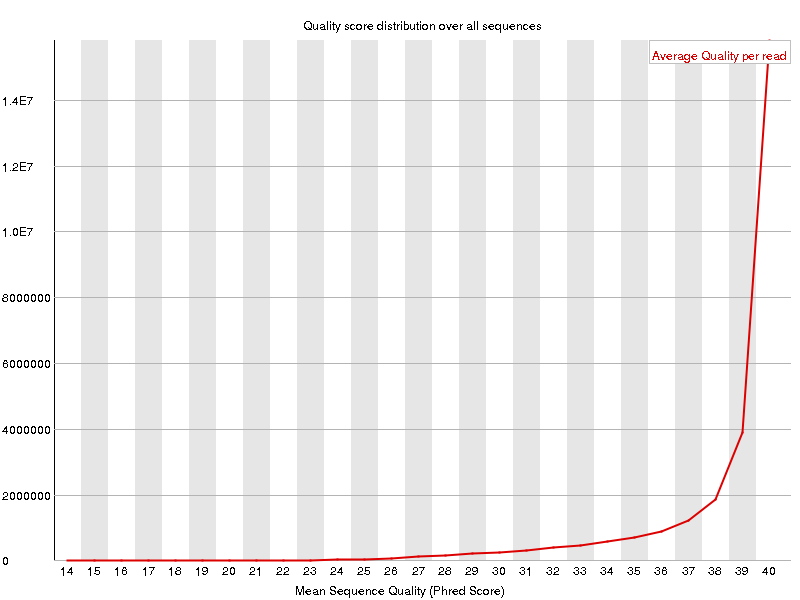

Supplement: Supplementary file 1 [file Data_Sheet_1.ZIP › 02_FastQC/C1_R1_fastqc/per_sequence_quality.png]

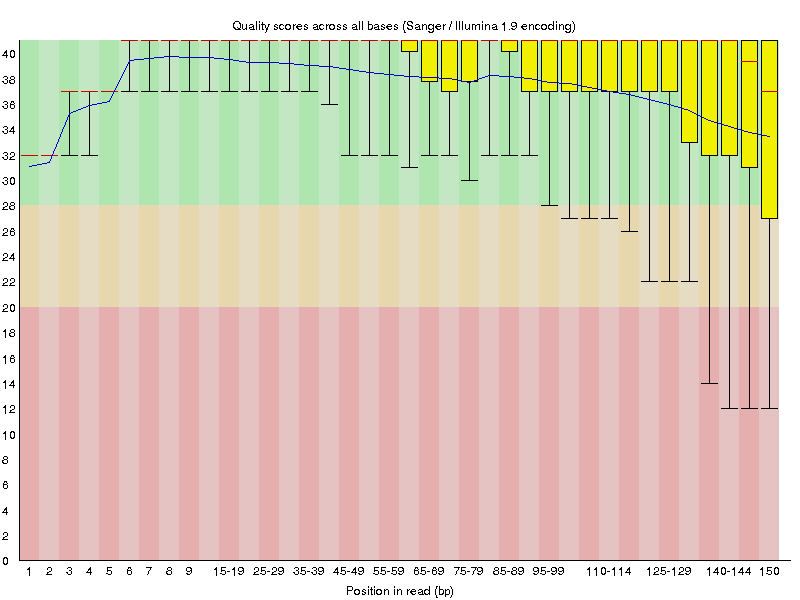

Supplement: Supplementary file 1 [file Data_Sheet_1.ZIP › 02_FastQC/C1_R2_fastqc/per_base_quality.png]

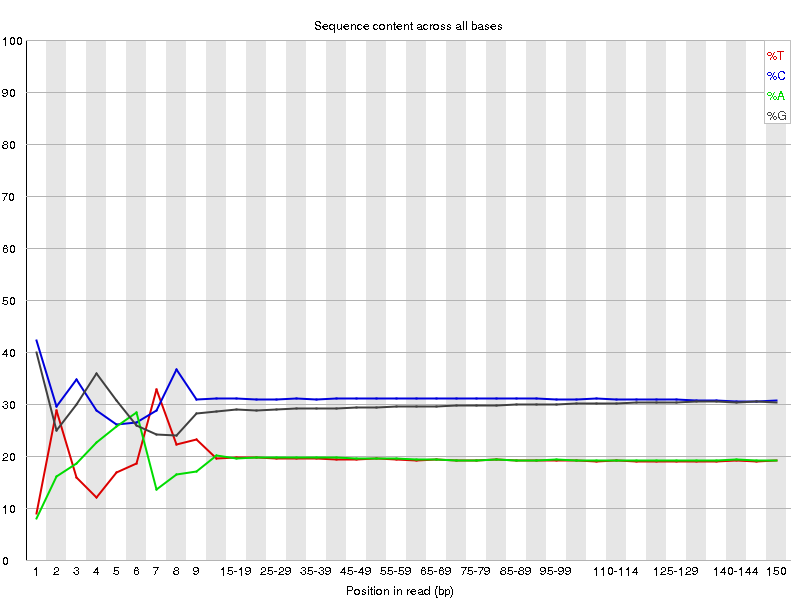

Supplement: Supplementary file 1 [file Data_Sheet_1.ZIP › 02_FastQC/C1_R2_fastqc/per_base_sequence_content.png]

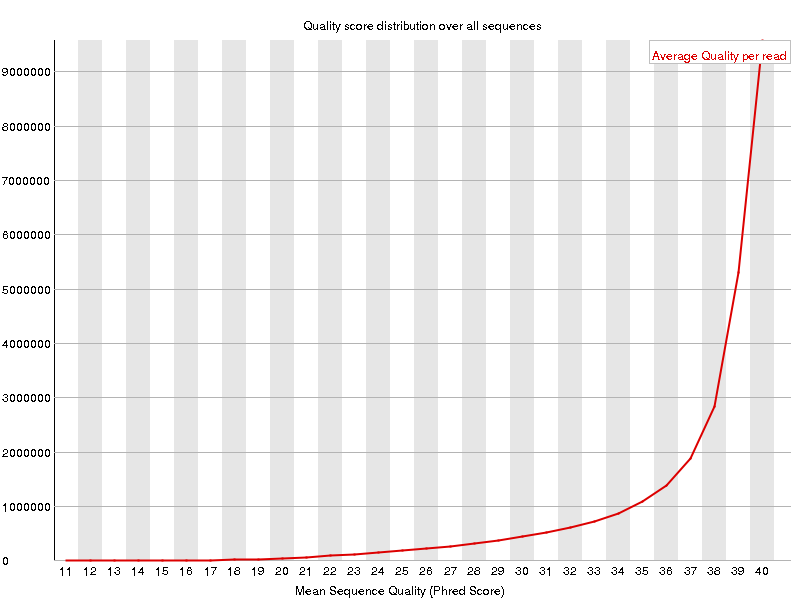

Supplement: Supplementary file 1 [file Data_Sheet_1.ZIP › 02_FastQC/C1_R2_fastqc/per_sequence_quality.png]

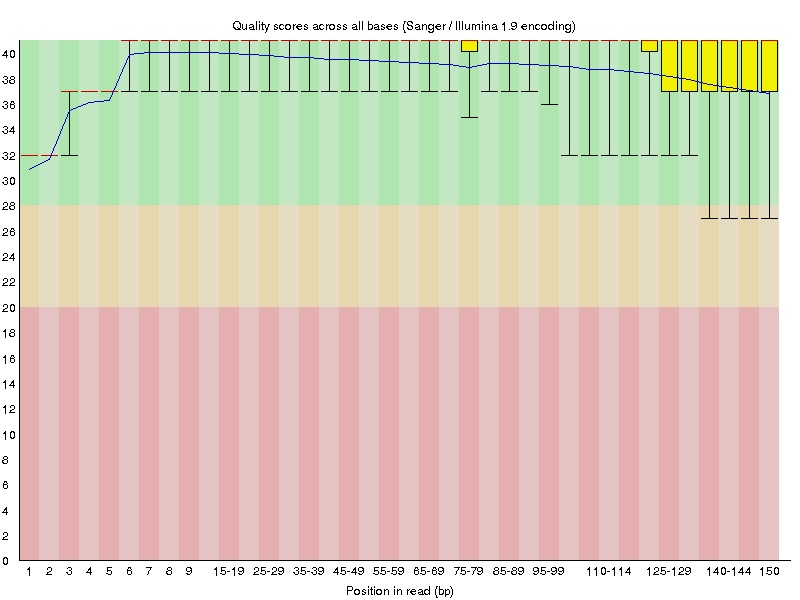

Supplement: Supplementary file 1 [file Data_Sheet_1.ZIP › 02_FastQC/C2_R1_fastqc/per_base_quality.png]

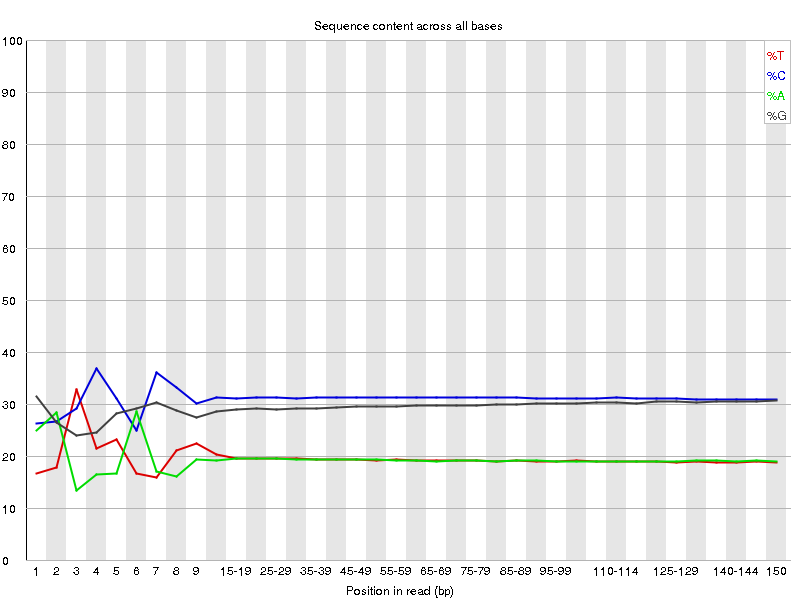

Supplement: Supplementary file 1 [file Data_Sheet_1.ZIP › 02_FastQC/C2_R1_fastqc/per_base_sequence_content.png]

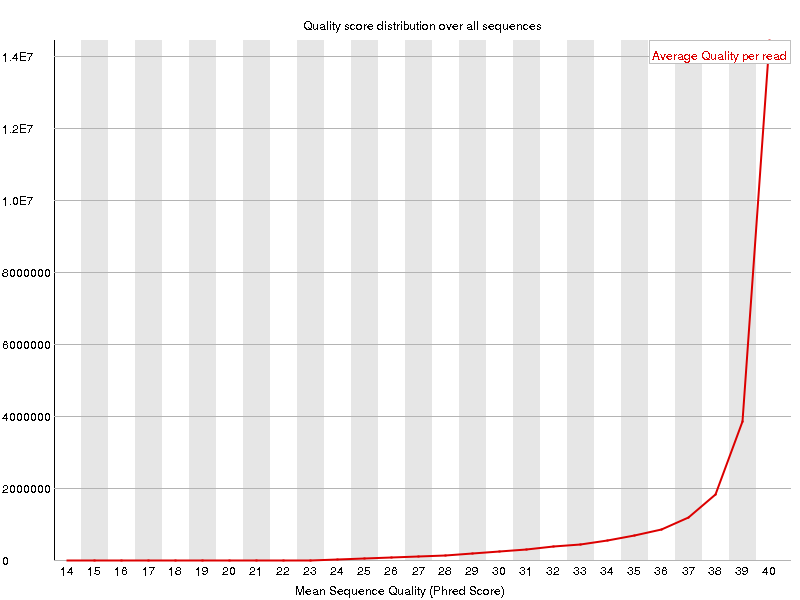

Supplement: Supplementary file 1 [file Data_Sheet_1.ZIP › 02_FastQC/C2_R1_fastqc/per_sequence_quality.png]

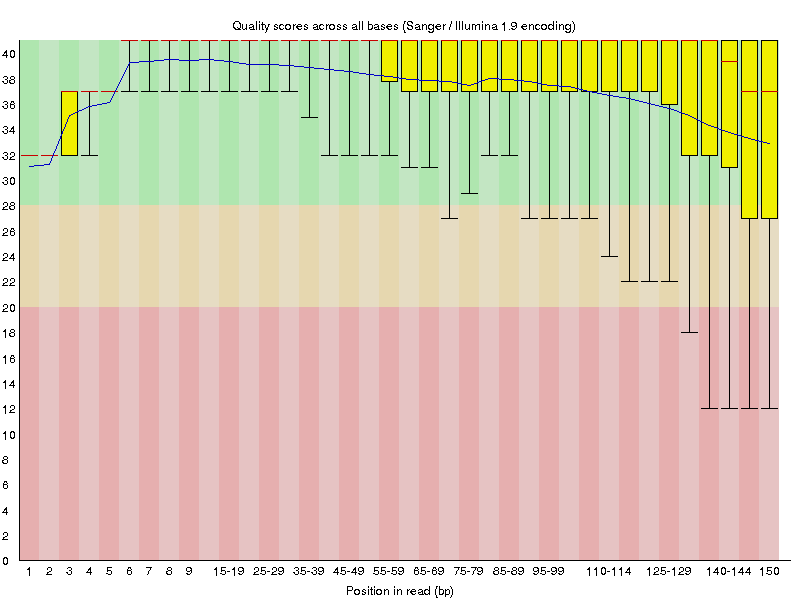

Supplement: Supplementary file 1 [file Data_Sheet_1.ZIP › 02_FastQC/C2_R2_fastqc/per_base_quality.png]

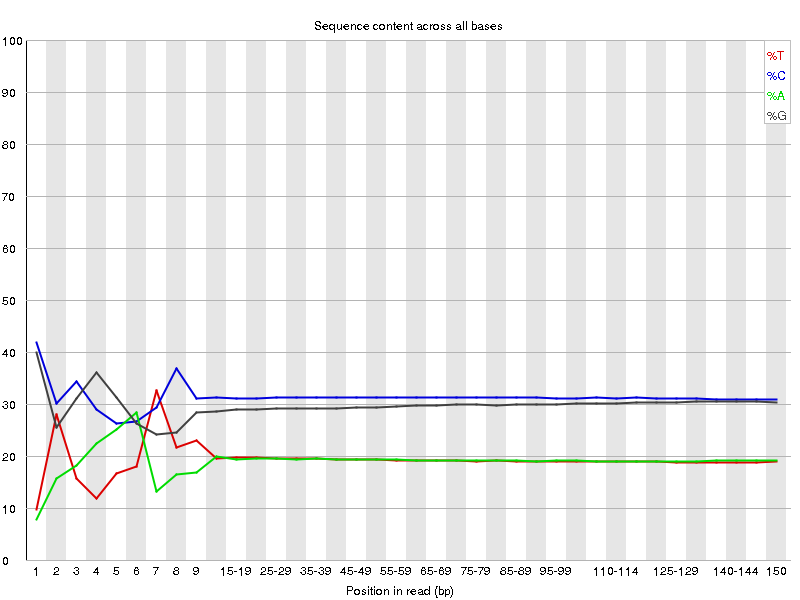

Supplement: Supplementary file 1 [file Data_Sheet_1.ZIP › 02_FastQC/C2_R2_fastqc/per_base_sequence_content.png]

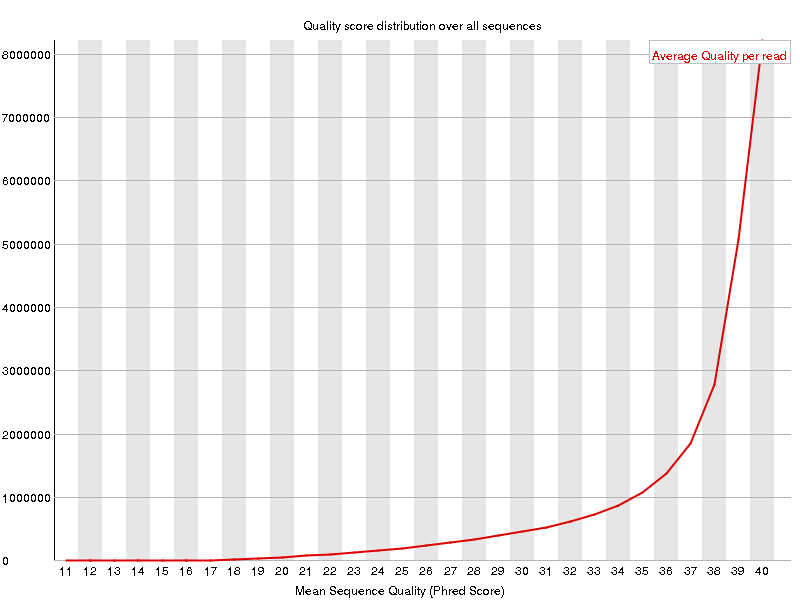

Supplement: Supplementary file 1 [file Data_Sheet_1.ZIP › 02_FastQC/C2_R2_fastqc/per_sequence_quality.png]

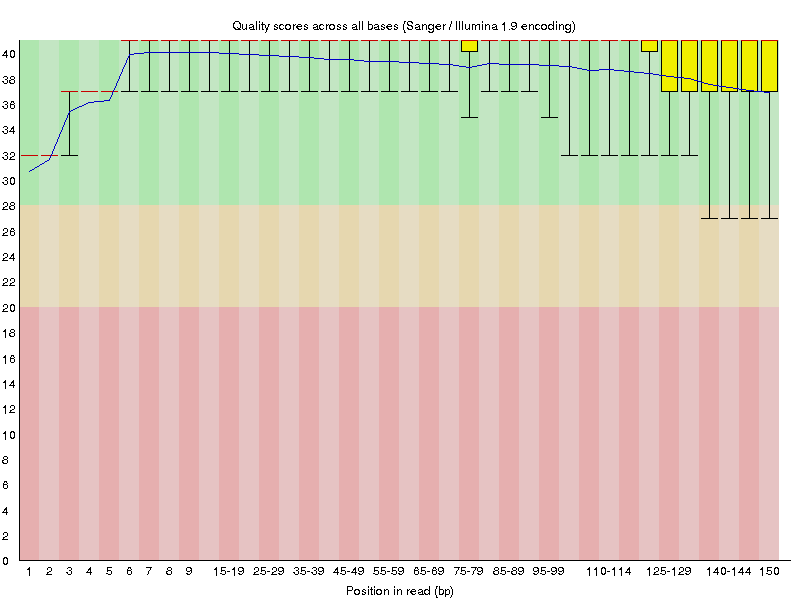

Supplement: Supplementary file 1 [file Data_Sheet_1.ZIP › 02_FastQC/C3_R1_fastqc/per_base_quality.png]

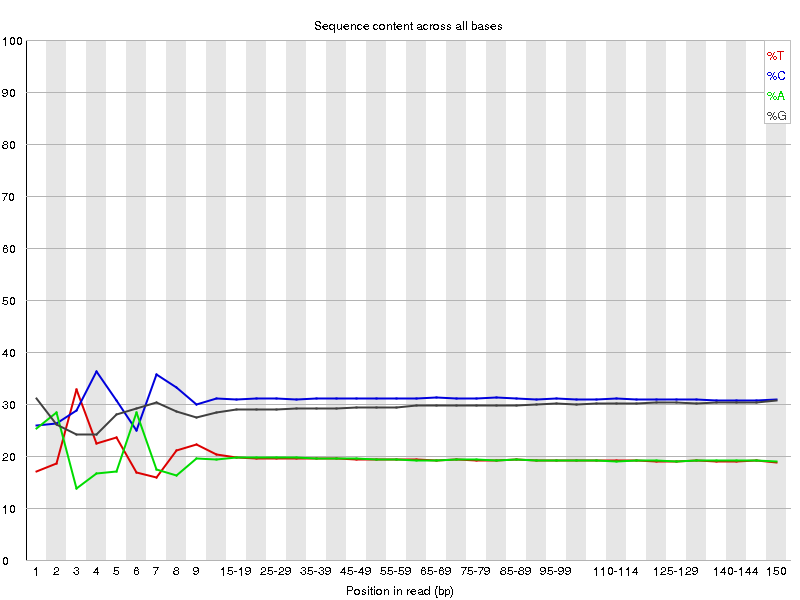

Supplement: Supplementary file 1 [file Data_Sheet_1.ZIP › 02_FastQC/C3_R1_fastqc/per_base_sequence_content.png]

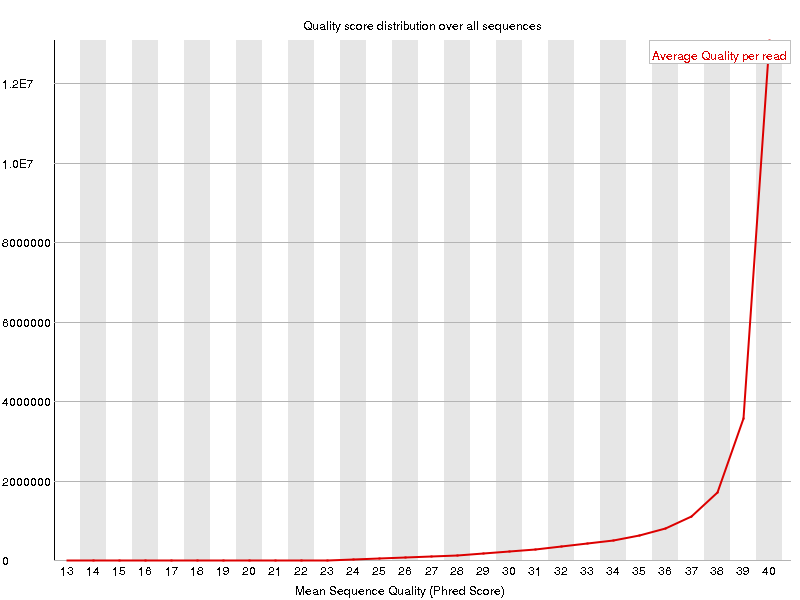

Supplement: Supplementary file 1 [file Data_Sheet_1.ZIP › 02_FastQC/C3_R1_fastqc/per_sequence_quality.png]

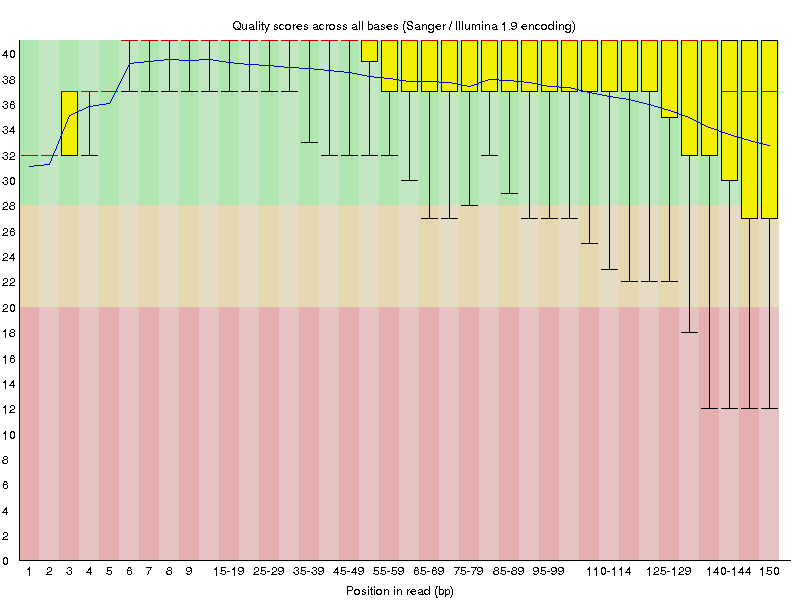

Supplement: Supplementary file 1 [file Data_Sheet_1.ZIP › 02_FastQC/C3_R2_fastqc/per_base_quality.png]

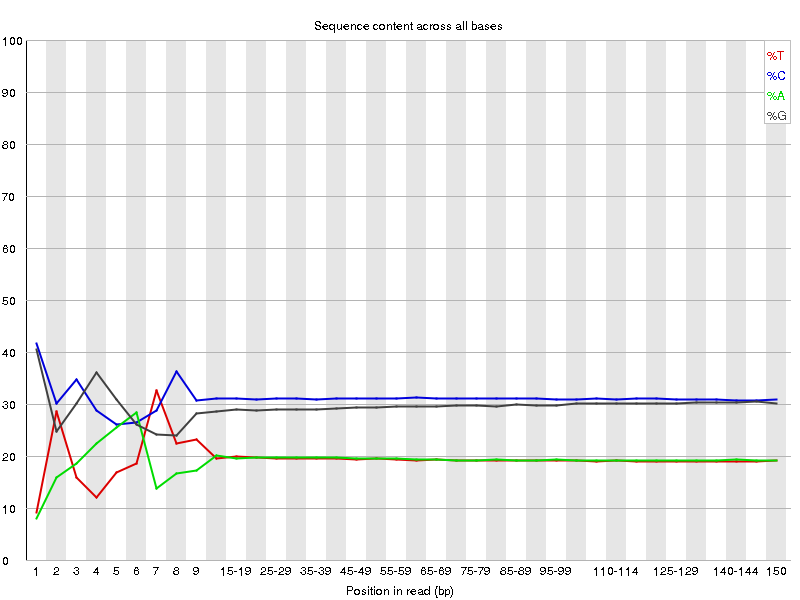

Supplement: Supplementary file 1 [file Data_Sheet_1.ZIP › 02_FastQC/C3_R2_fastqc/per_base_sequence_content.png]

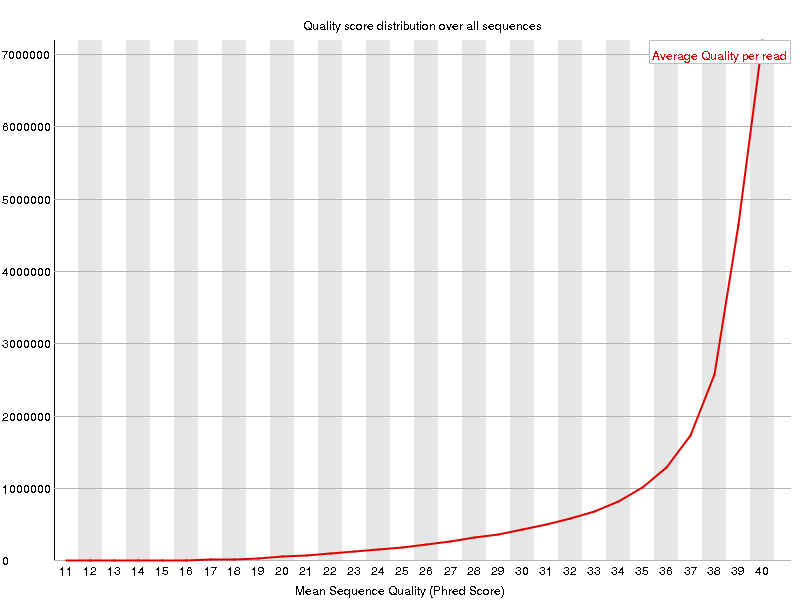

Supplement: Supplementary file 1 [file Data_Sheet_1.ZIP › 02_FastQC/C3_R2_fastqc/per_sequence_quality.png]

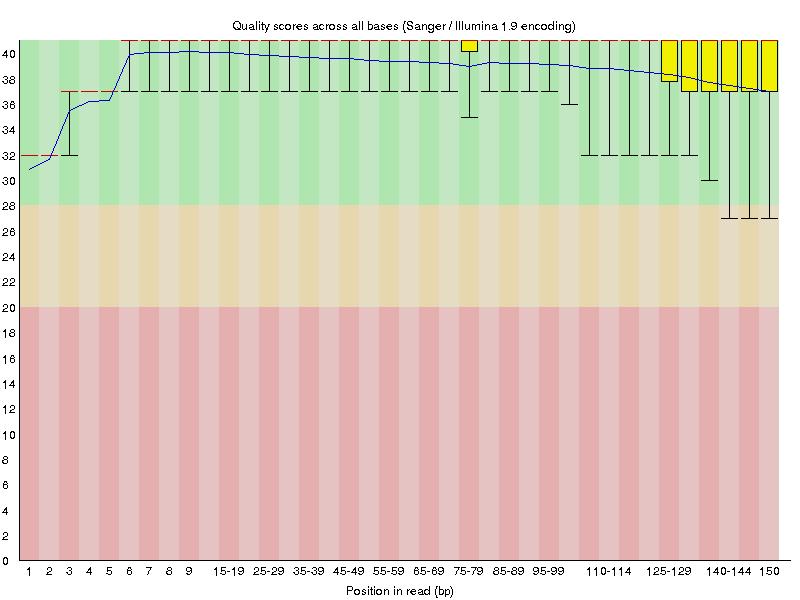

Supplement: Supplementary file 1 [file Data_Sheet_1.ZIP › 02_FastQC/D1_R1_fastqc/per_base_quality.png]

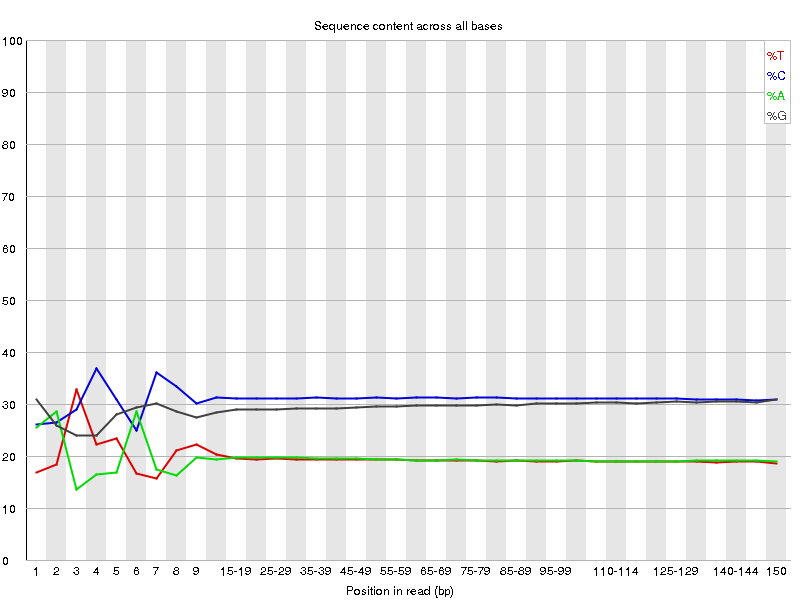

Supplement: Supplementary file 1 [file Data_Sheet_1.ZIP › 02_FastQC/D1_R1_fastqc/per_base_sequence_content.png]

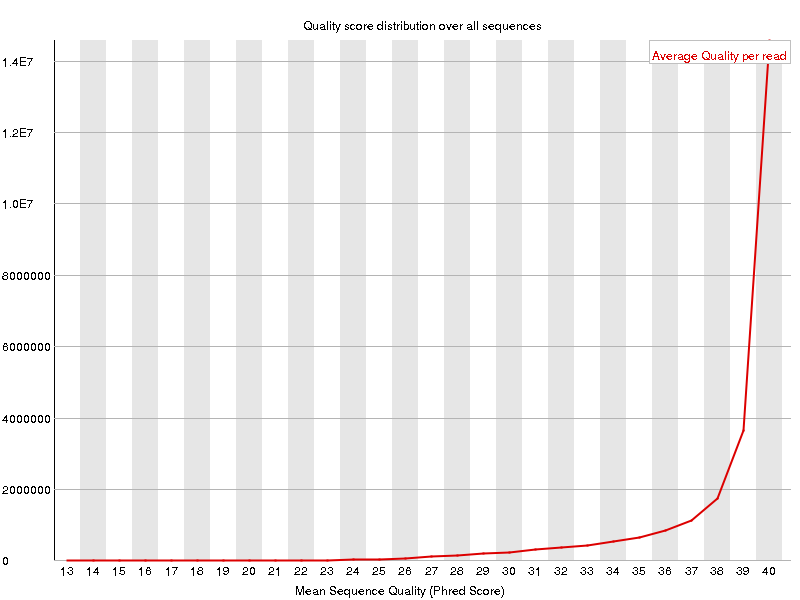

Supplement: Supplementary file 1 [file Data_Sheet_1.ZIP › 02_FastQC/D1_R1_fastqc/per_sequence_quality.png]

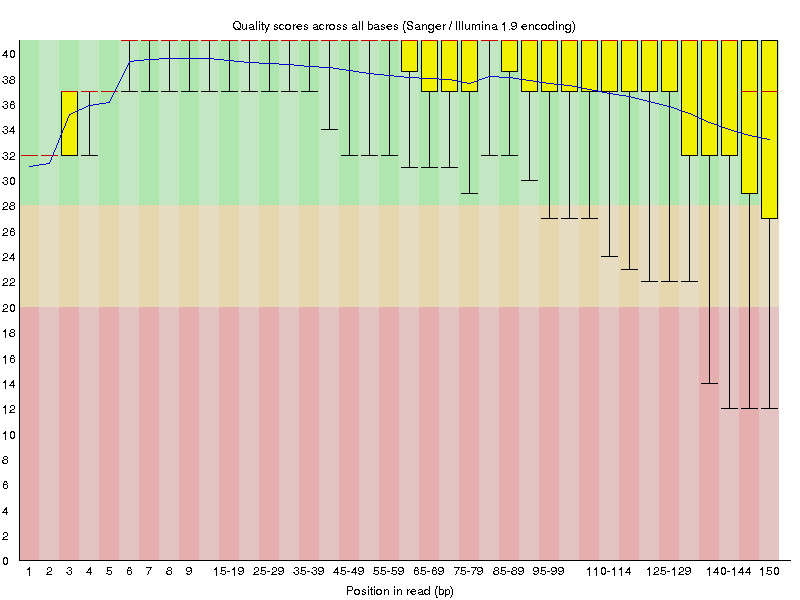

Supplement: Supplementary file 1 [file Data_Sheet_1.ZIP › 02_FastQC/D1_R2_fastqc/per_base_quality.png]

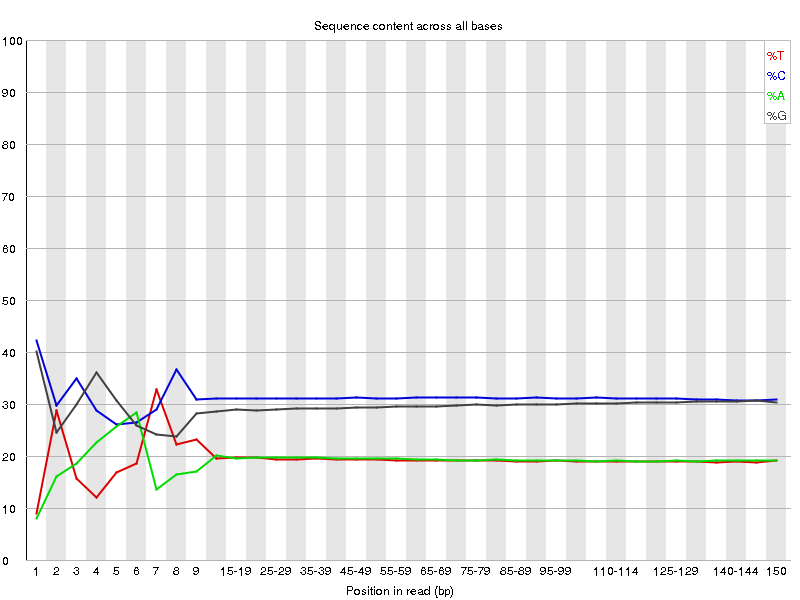

Supplement: Supplementary file 1 [file Data_Sheet_1.ZIP › 02_FastQC/D1_R2_fastqc/per_base_sequence_content.png]

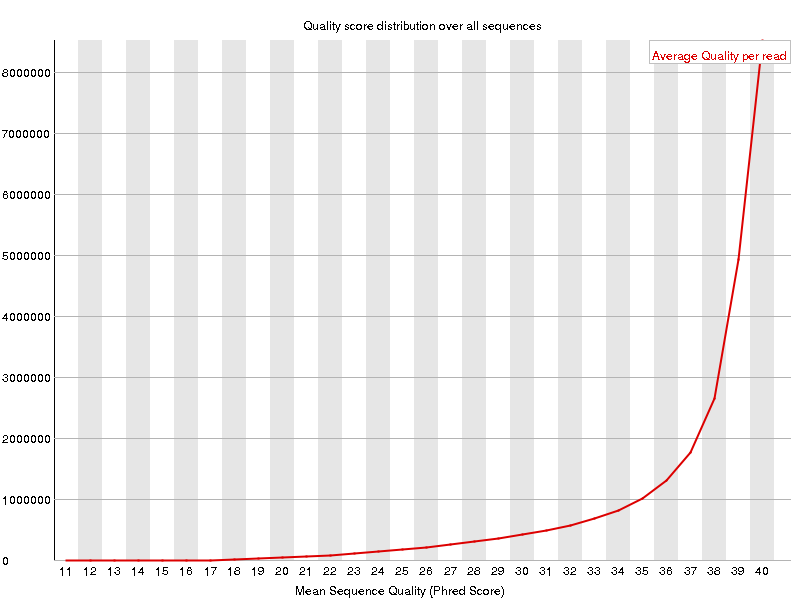

Supplement: Supplementary file 1 [file Data_Sheet_1.ZIP › 02_FastQC/D1_R2_fastqc/per_sequence_quality.png]

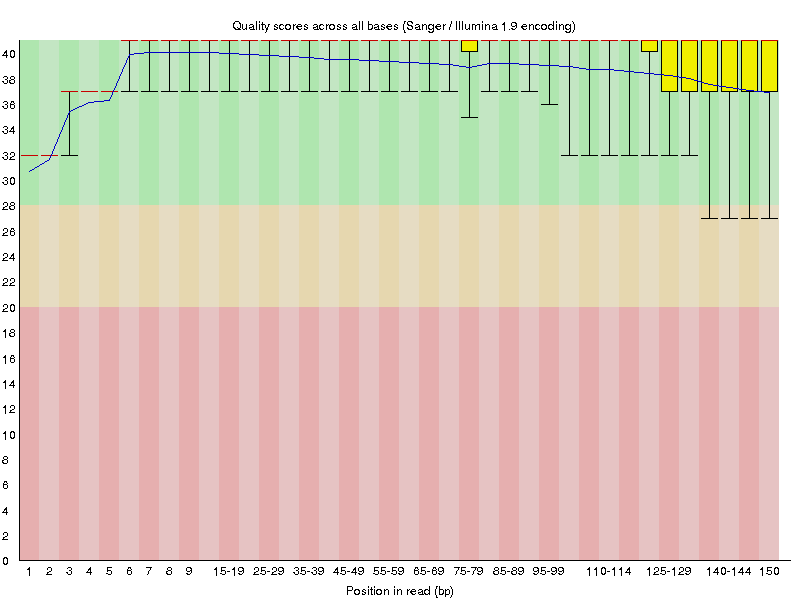

Supplement: Supplementary file 1 [file Data_Sheet_1.ZIP › 02_FastQC/D2_R1_fastqc/per_base_quality.png]

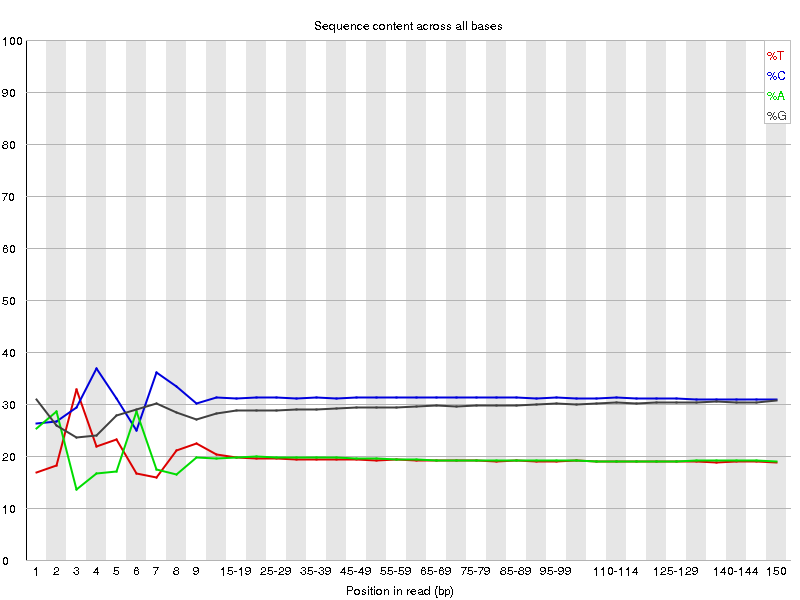

Supplement: Supplementary file 1 [file Data_Sheet_1.ZIP › 02_FastQC/D2_R1_fastqc/per_base_sequence_content.png]

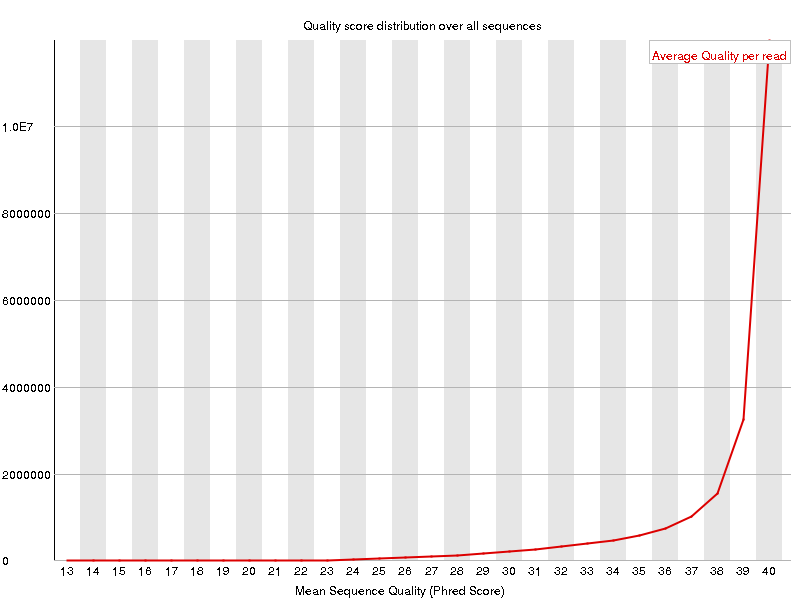

Supplement: Supplementary file 1 [file Data_Sheet_1.ZIP › 02_FastQC/D2_R1_fastqc/per_sequence_quality.png]

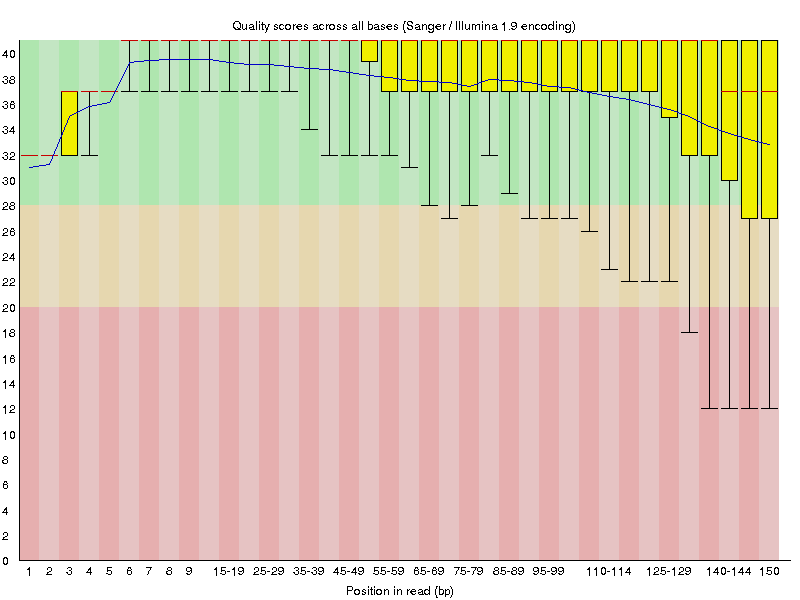

Supplement: Supplementary file 1 [file Data_Sheet_1.ZIP › 02_FastQC/D2_R2_fastqc/per_base_quality.png]

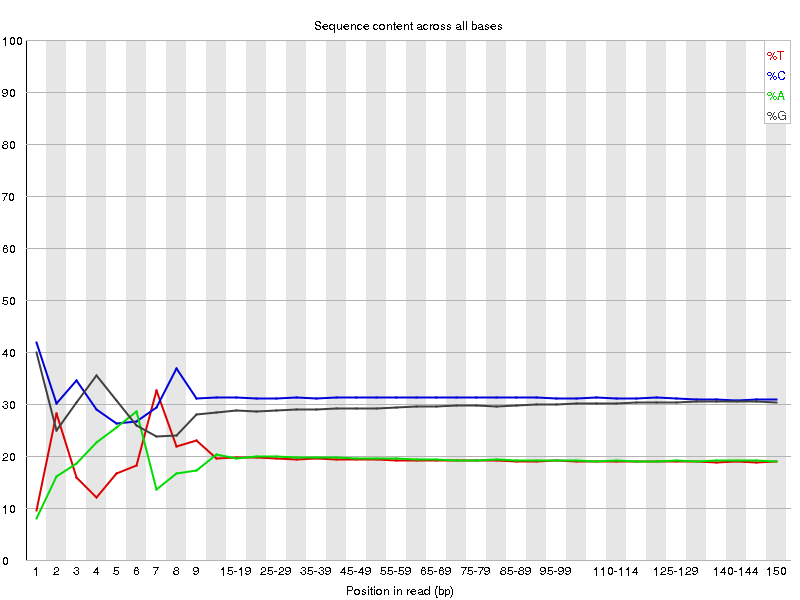

Supplement: Supplementary file 1 [file Data_Sheet_1.ZIP › 02_FastQC/D2_R2_fastqc/per_base_sequence_content.png]

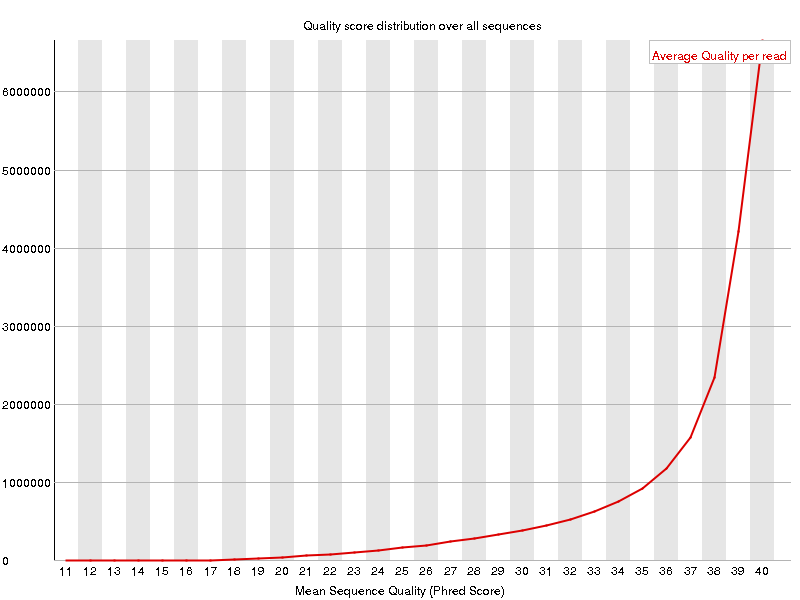

Supplement: Supplementary file 1 [file Data_Sheet_1.ZIP › 02_FastQC/D2_R2_fastqc/per_sequence_quality.png]

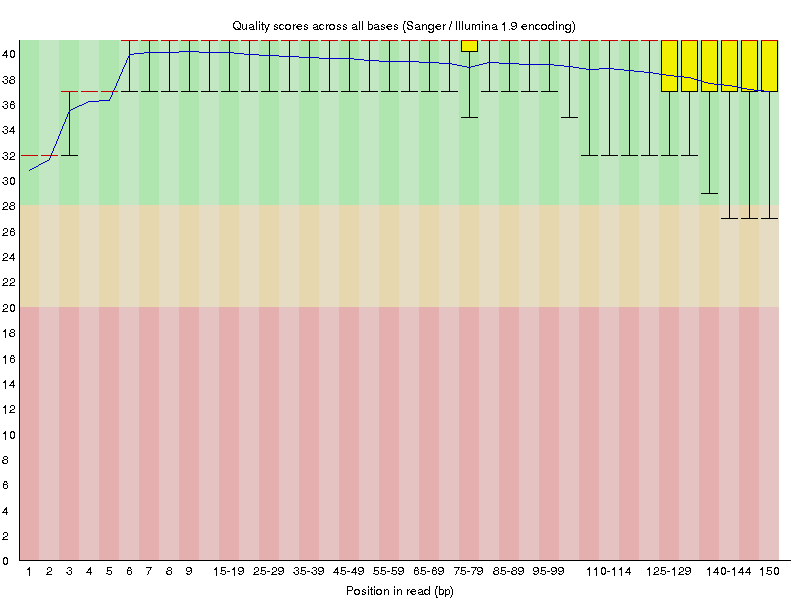

Supplement: Supplementary file 1 [file Data_Sheet_1.ZIP › 02_FastQC/D3_R1_fastqc/per_base_quality.png]

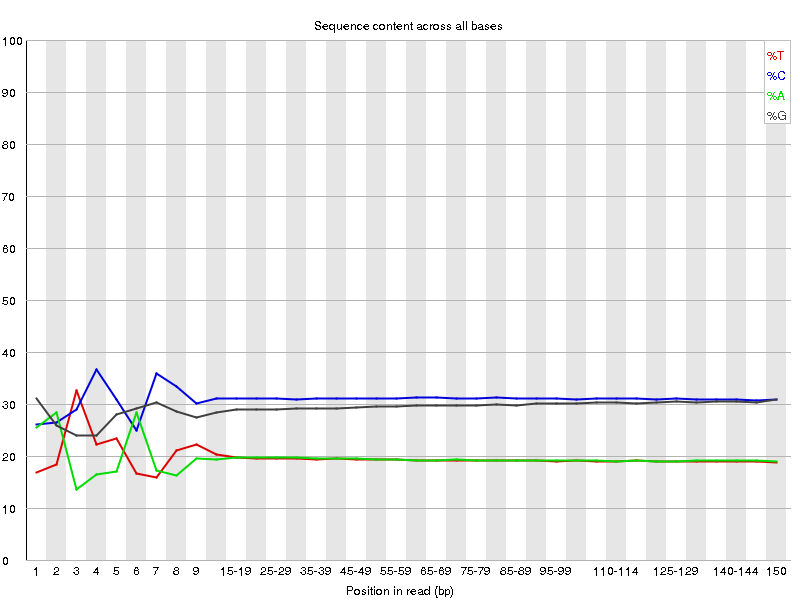

Supplement: Supplementary file 1 [file Data_Sheet_1.ZIP › 02_FastQC/D3_R1_fastqc/per_base_sequence_content.png]

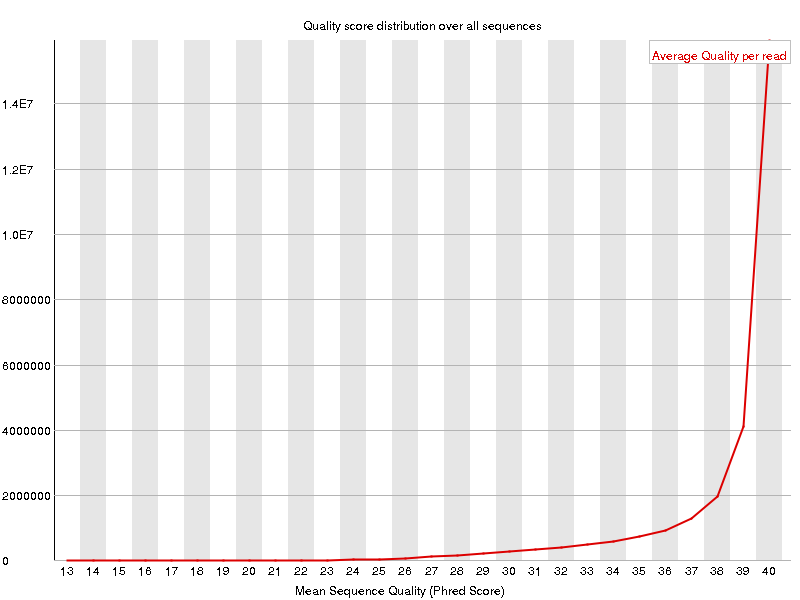

Supplement: Supplementary file 1 [file Data_Sheet_1.ZIP › 02_FastQC/D3_R1_fastqc/per_sequence_quality.png]

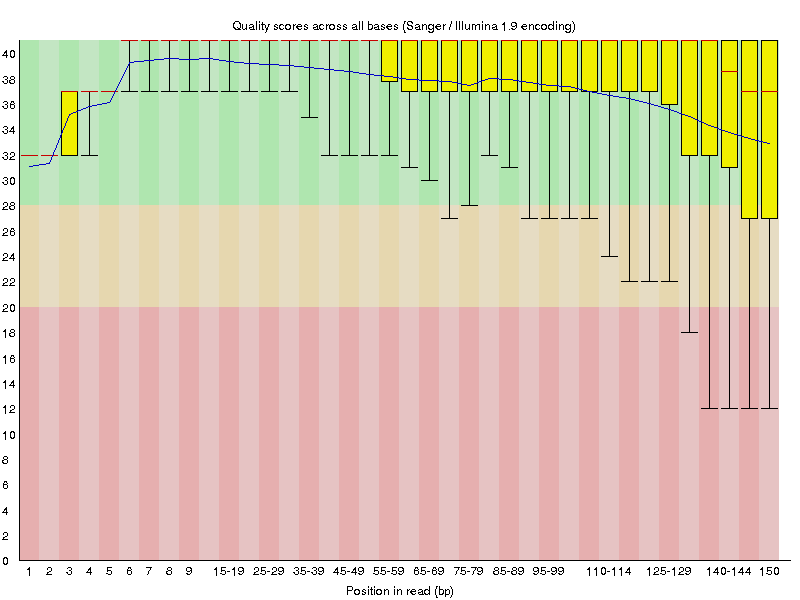

Supplement: Supplementary file 1 [file Data_Sheet_1.ZIP › 02_FastQC/D3_R2_fastqc/per_base_quality.png]

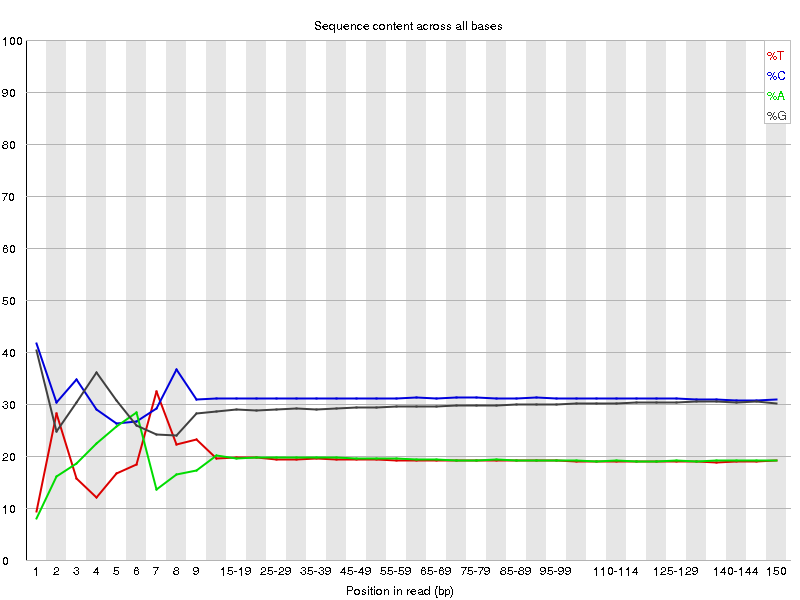

Supplement: Supplementary file 1 [file Data_Sheet_1.ZIP › 02_FastQC/D3_R2_fastqc/per_base_sequence_content.png]

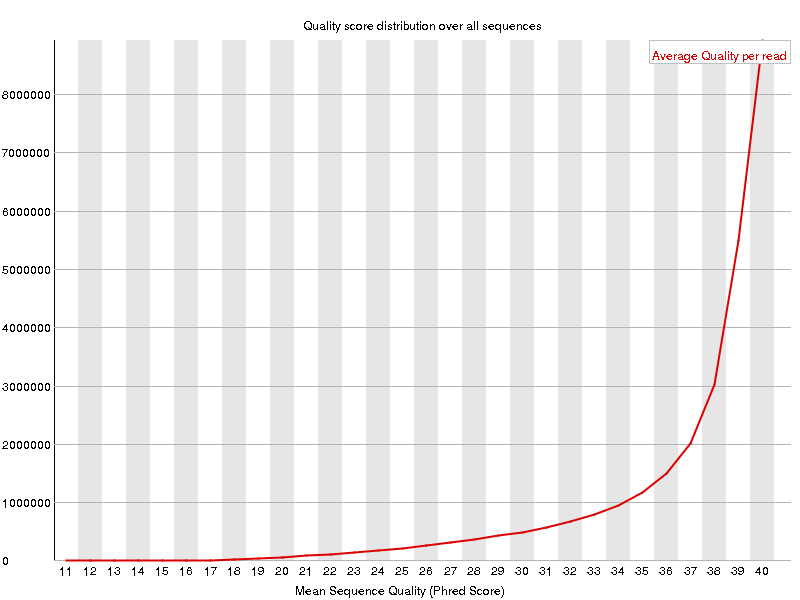

Supplement: Supplementary file 1 [file Data_Sheet_1.ZIP › 02_FastQC/D3_R2_fastqc/per_sequence_quality.png]

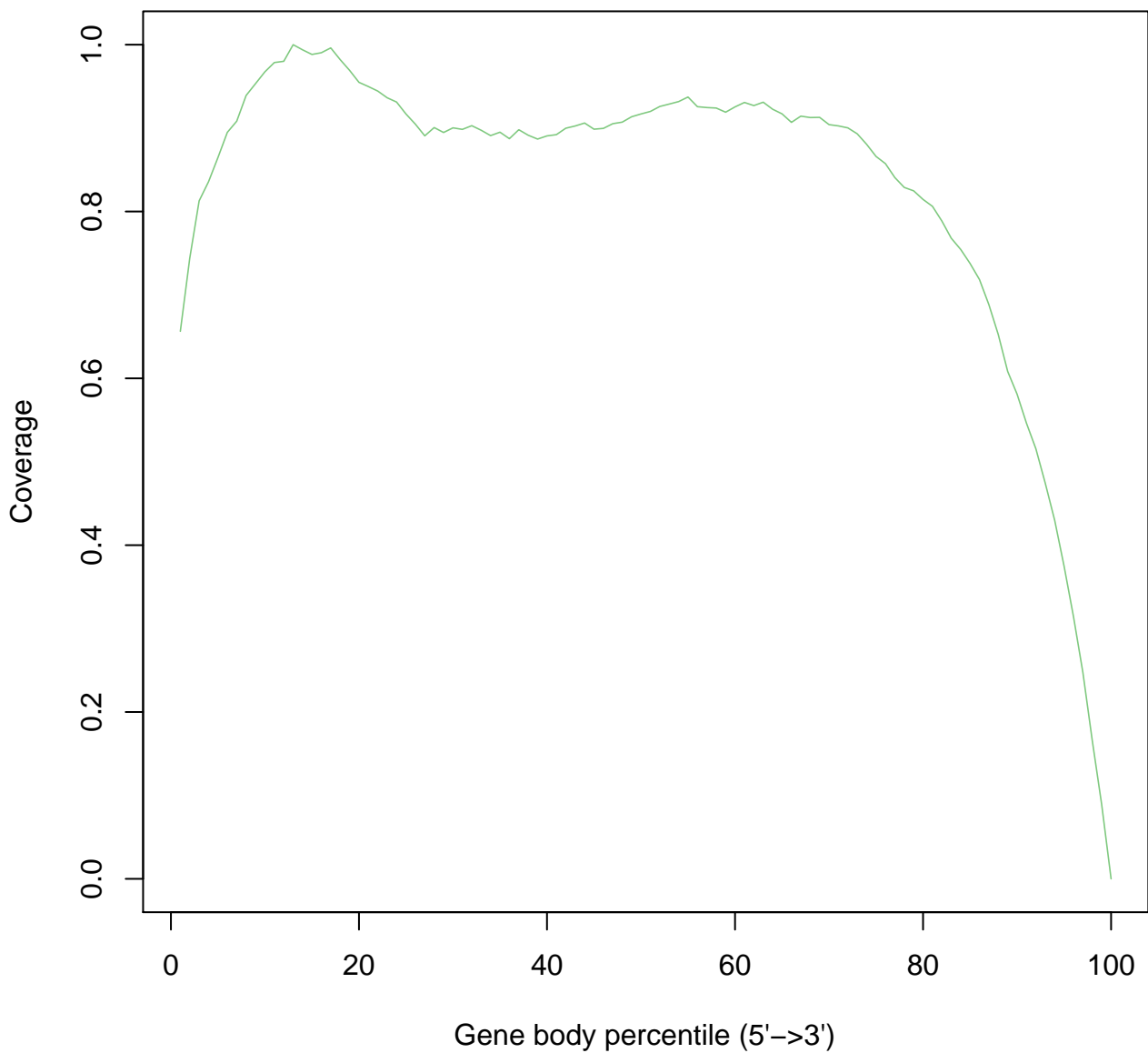

Supplement: Supplementary file 1 [file Data_Sheet_1.ZIP › 04_MapQC/genebody_coverage/A1.geneBodyCoverage.curves.pdf]

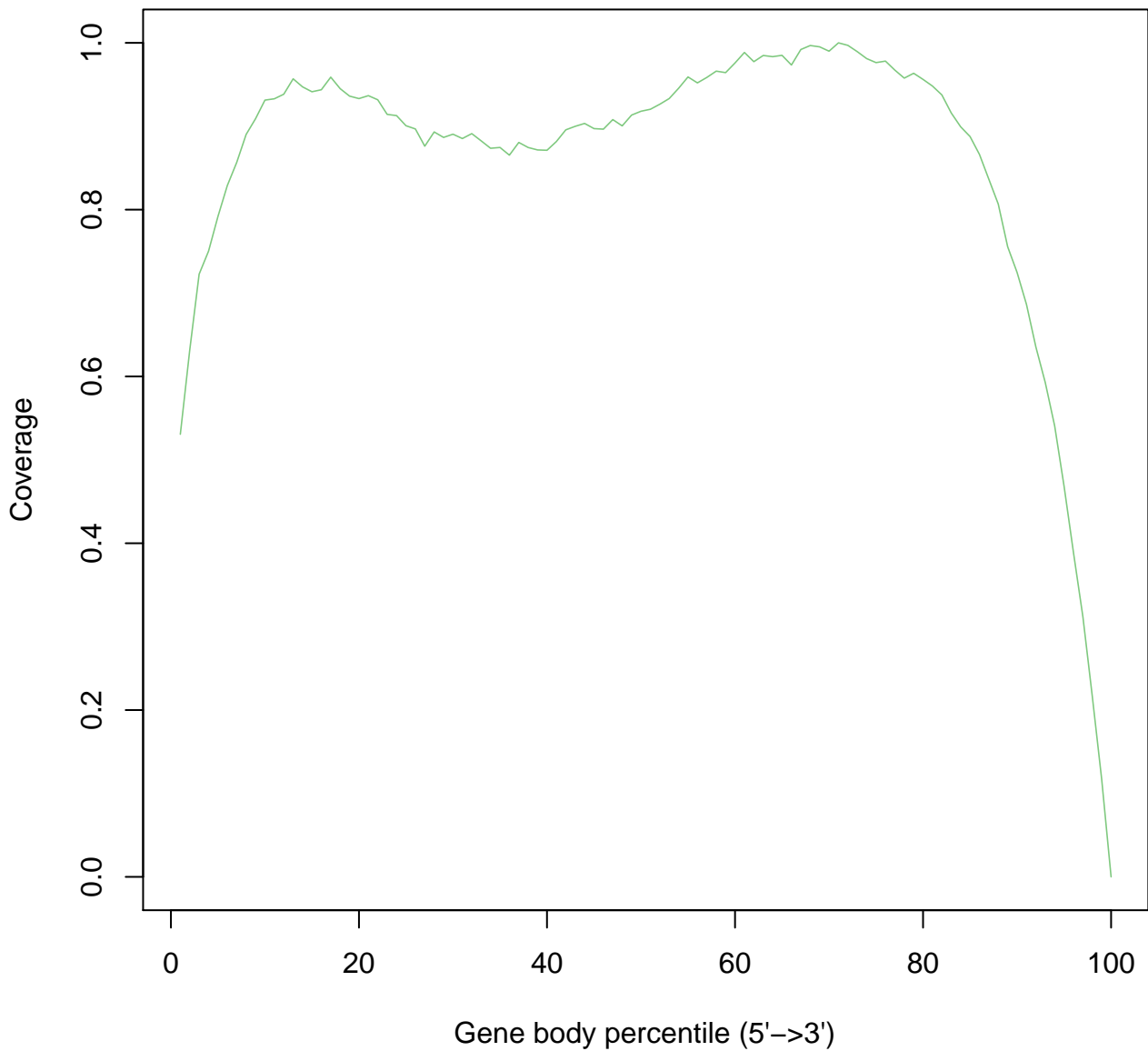

Supplement: Supplementary file 1 [file Data_Sheet_1.ZIP › 04_MapQC/genebody_coverage/A2.geneBodyCoverage.curves.pdf]

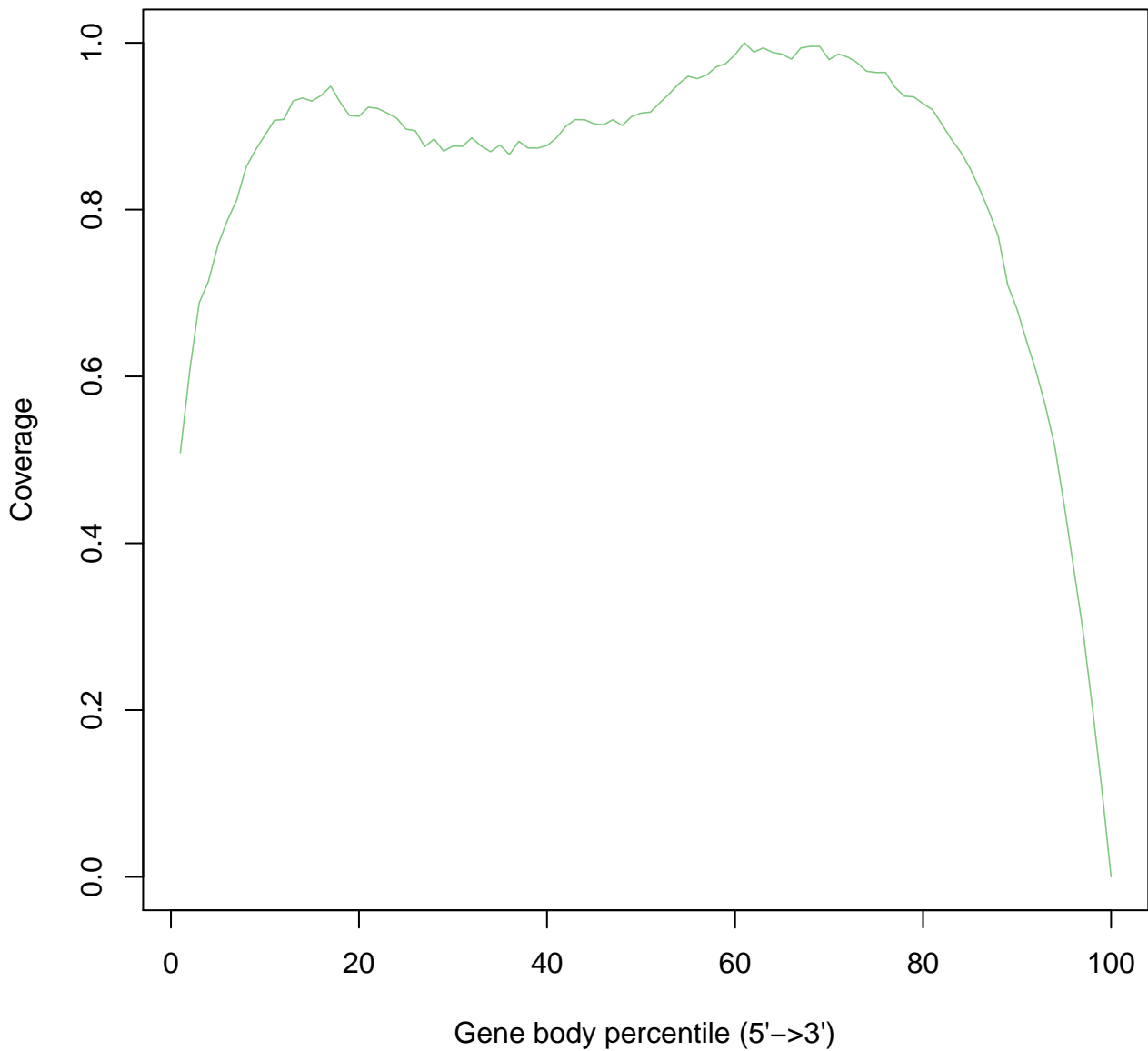

Supplement: Supplementary file 1 [file Data_Sheet_1.ZIP › 04_MapQC/genebody_coverage/A3.geneBodyCoverage.curves.pdf]

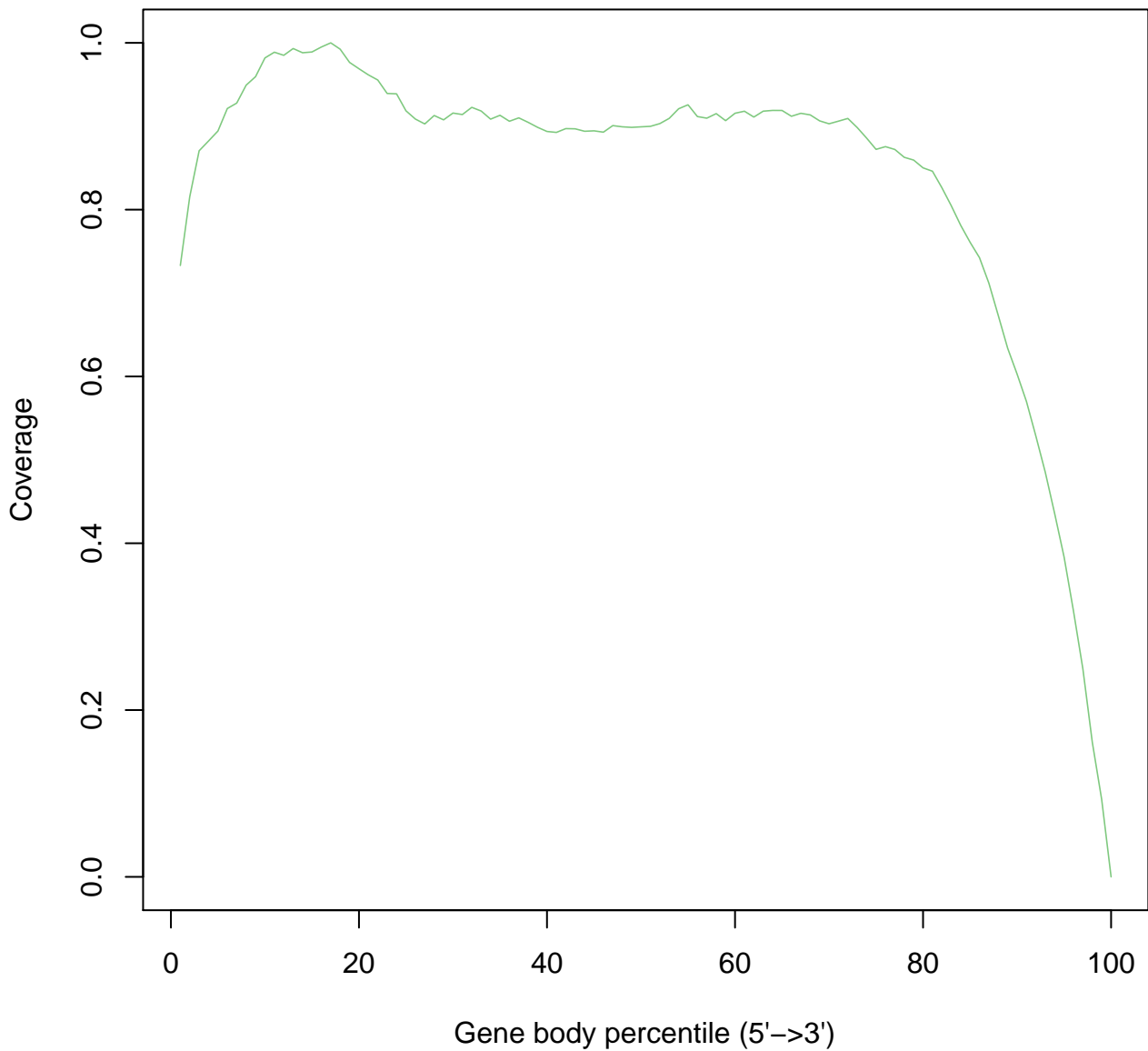

Supplement: Supplementary file 1 [file Data_Sheet_1.ZIP › 04_MapQC/genebody_coverage/B1.geneBodyCoverage.curves.pdf]

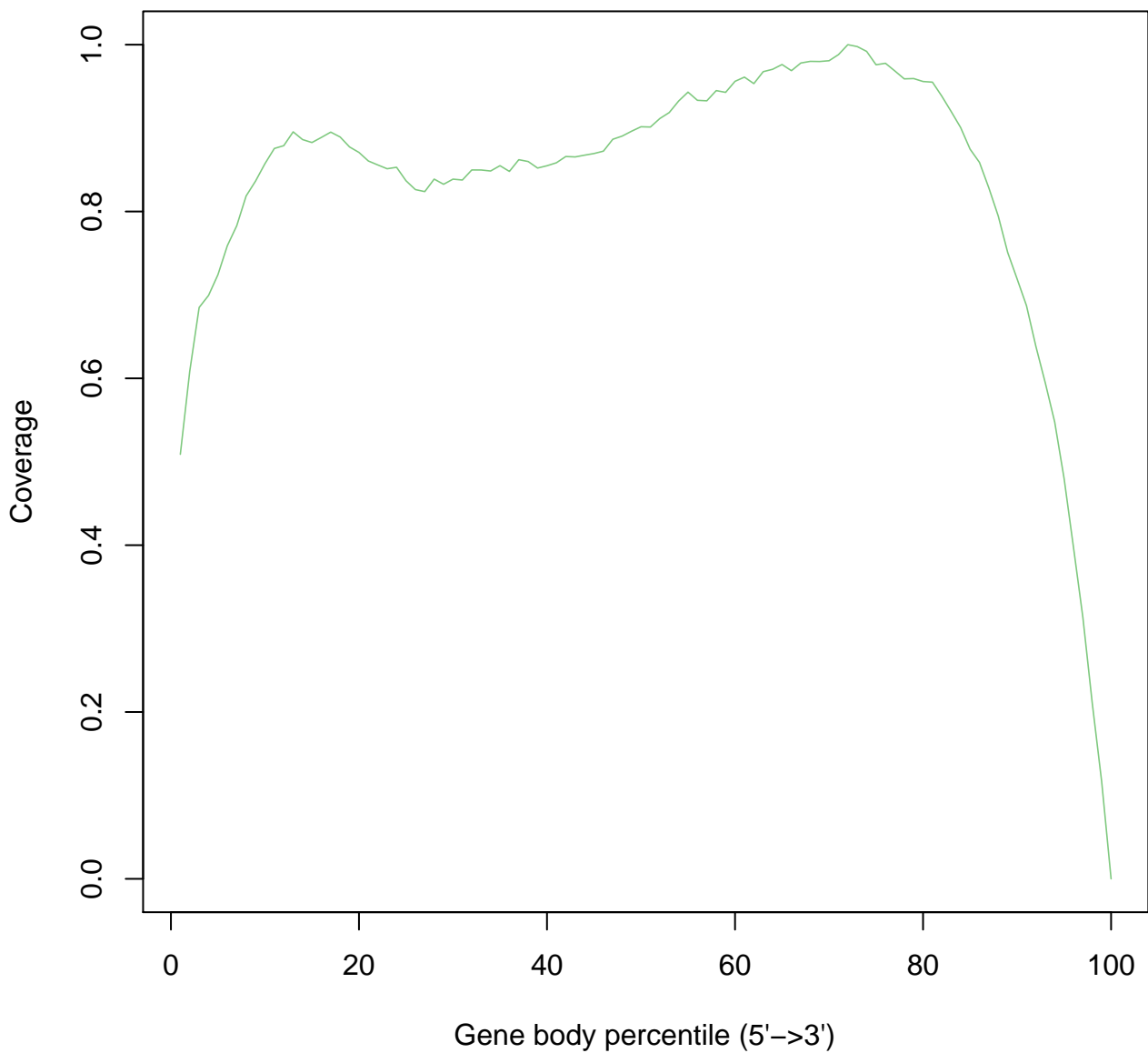

Supplement: Supplementary file 1 [file Data_Sheet_1.ZIP › 04_MapQC/genebody_coverage/B2.geneBodyCoverage.curves.pdf]

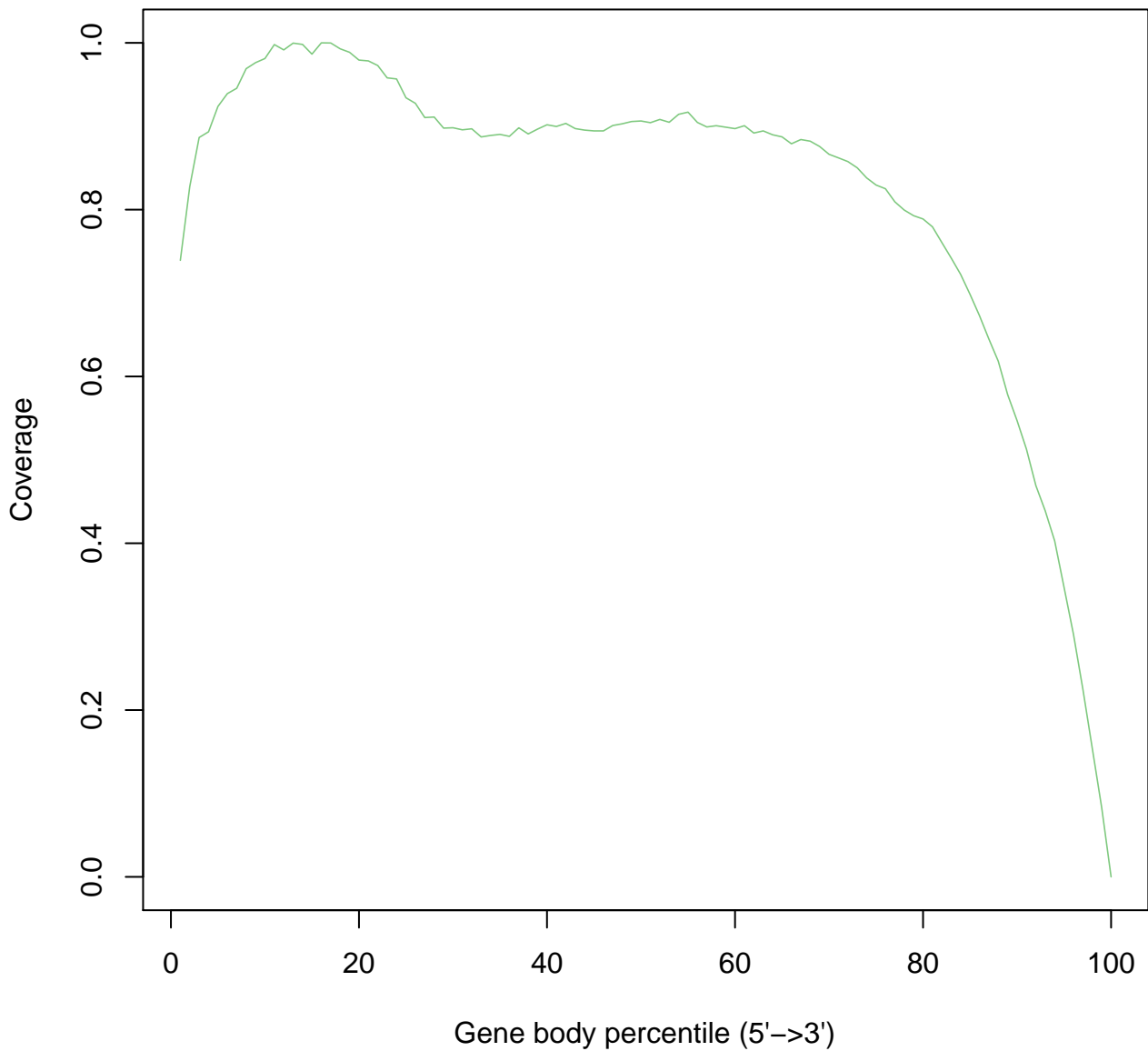

Supplement: Supplementary file 1 [file Data_Sheet_1.ZIP › 04_MapQC/genebody_coverage/B3.geneBodyCoverage.curves.pdf]

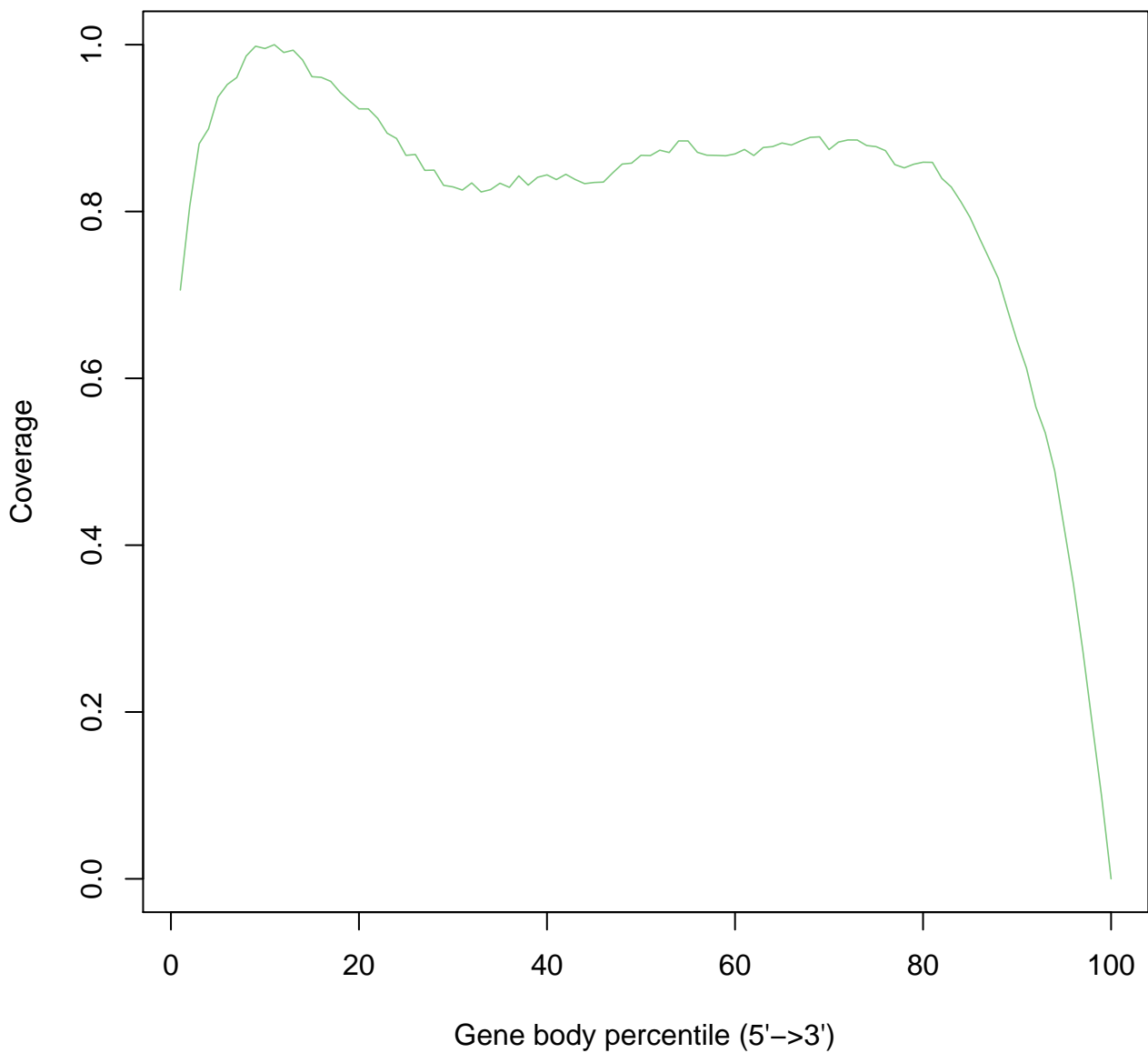

Supplement: Supplementary file 1 [file Data_Sheet_1.ZIP › 04_MapQC/genebody_coverage/C1.geneBodyCoverage.curves.pdf]

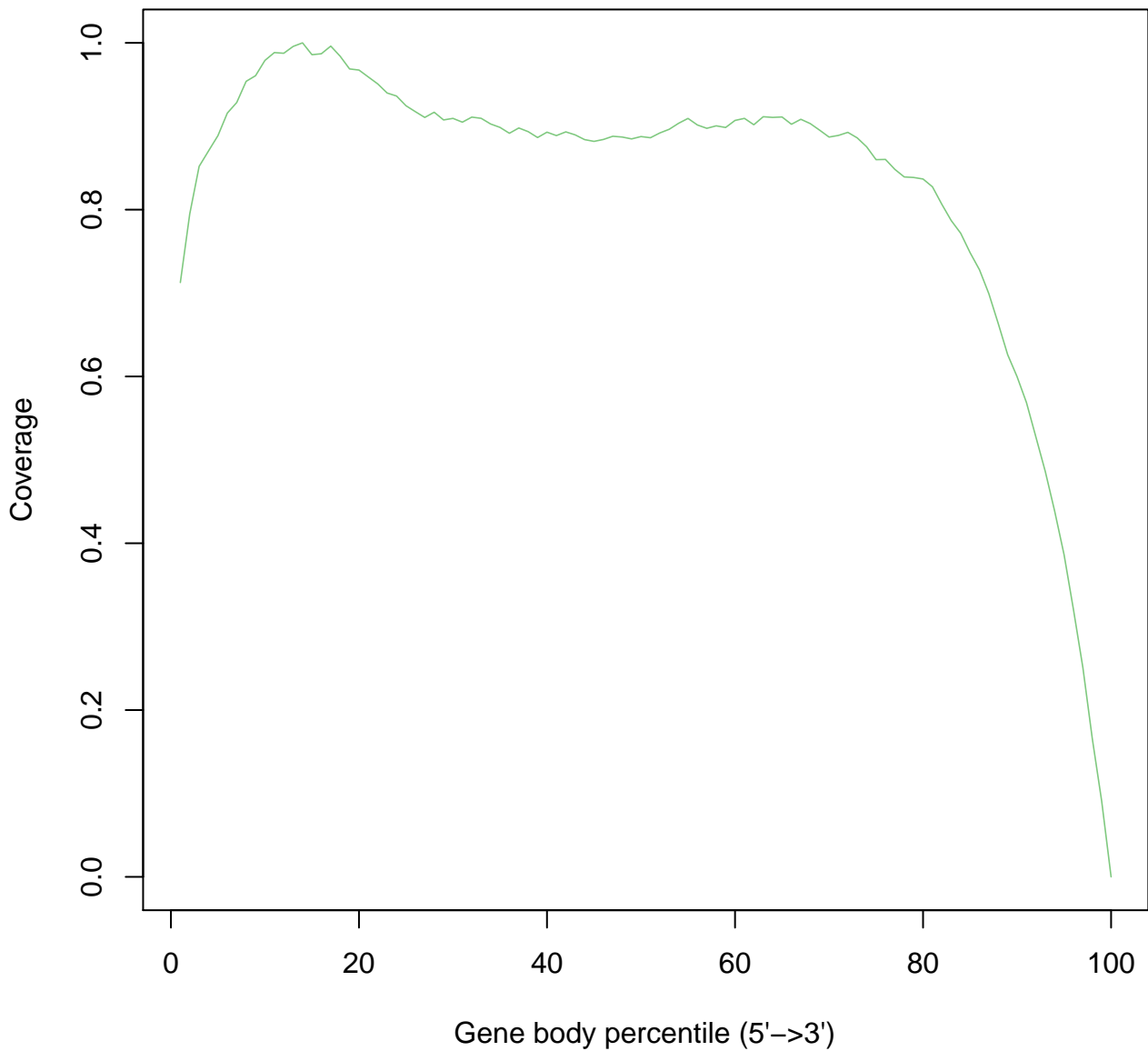

Supplement: Supplementary file 1 [file Data_Sheet_1.ZIP › 04_MapQC/genebody_coverage/C2.geneBodyCoverage.curves.pdf]

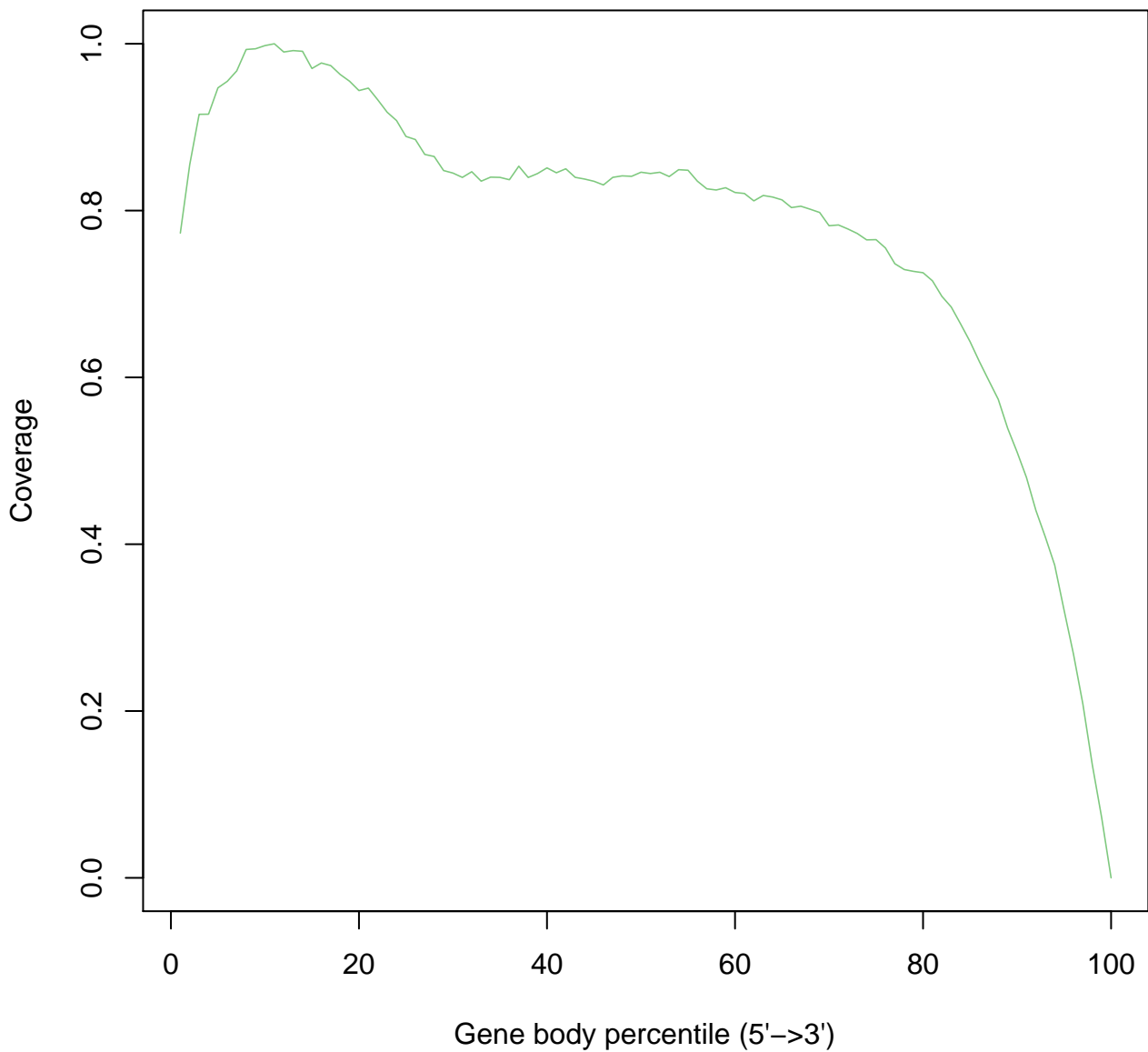

Supplement: Supplementary file 1 [file Data_Sheet_1.ZIP › 04_MapQC/genebody_coverage/C3.geneBodyCoverage.curves.pdf]

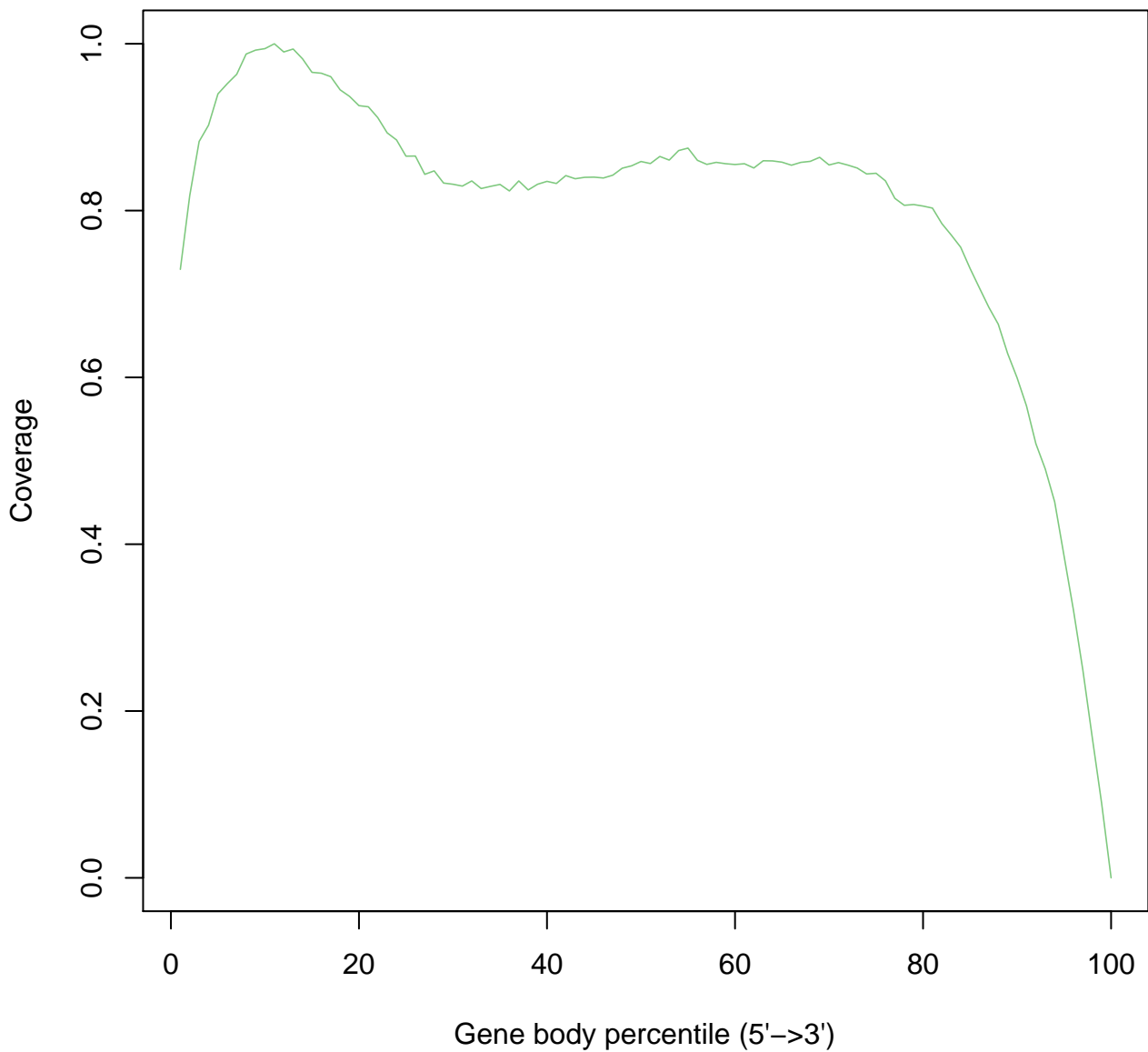

Supplement: Supplementary file 1 [file Data_Sheet_1.ZIP › 04_MapQC/genebody_coverage/D1.geneBodyCoverage.curves.pdf]

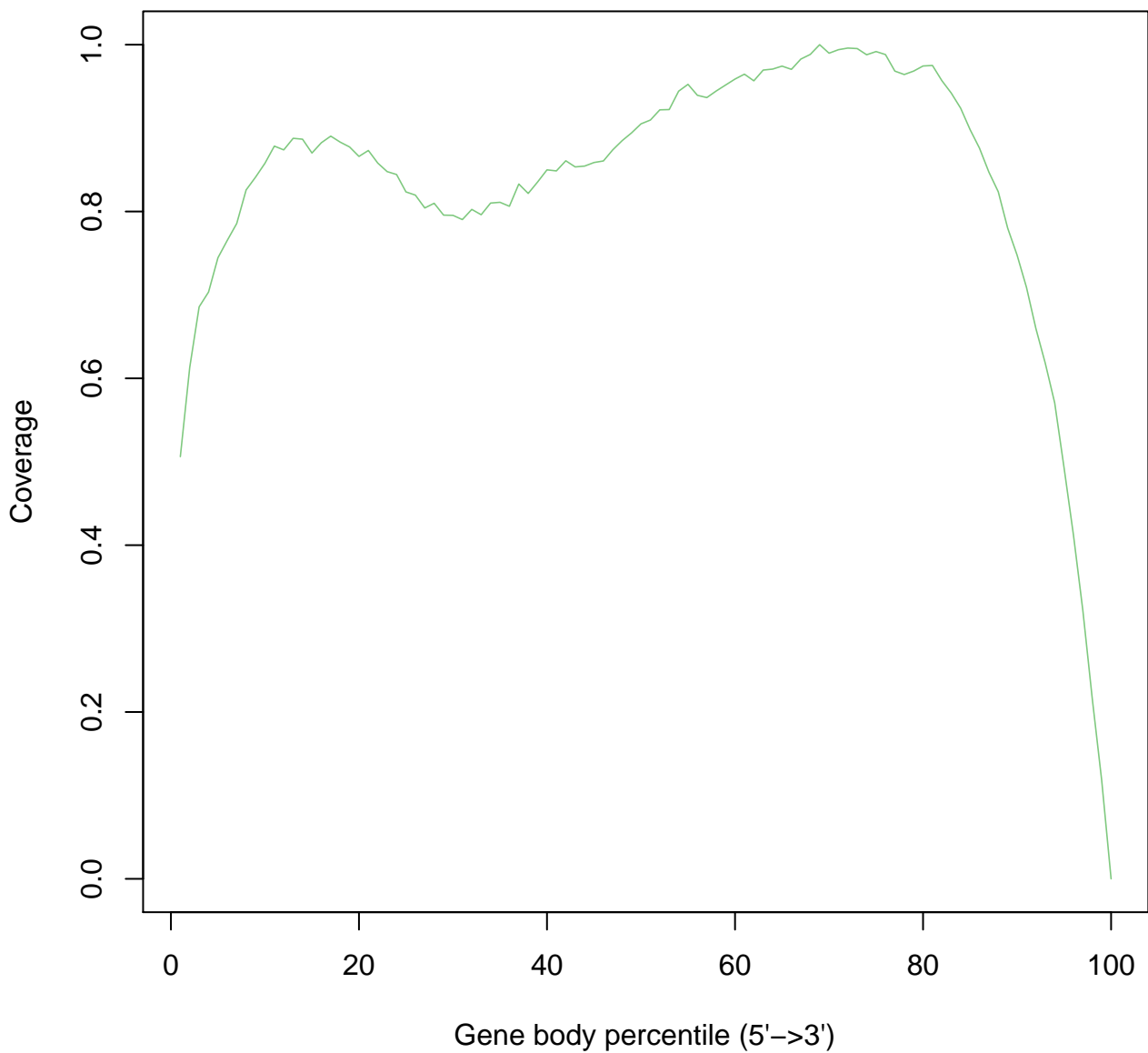

Supplement: Supplementary file 1 [file Data_Sheet_1.ZIP › 04_MapQC/genebody_coverage/D2.geneBodyCoverage.curves.pdf]

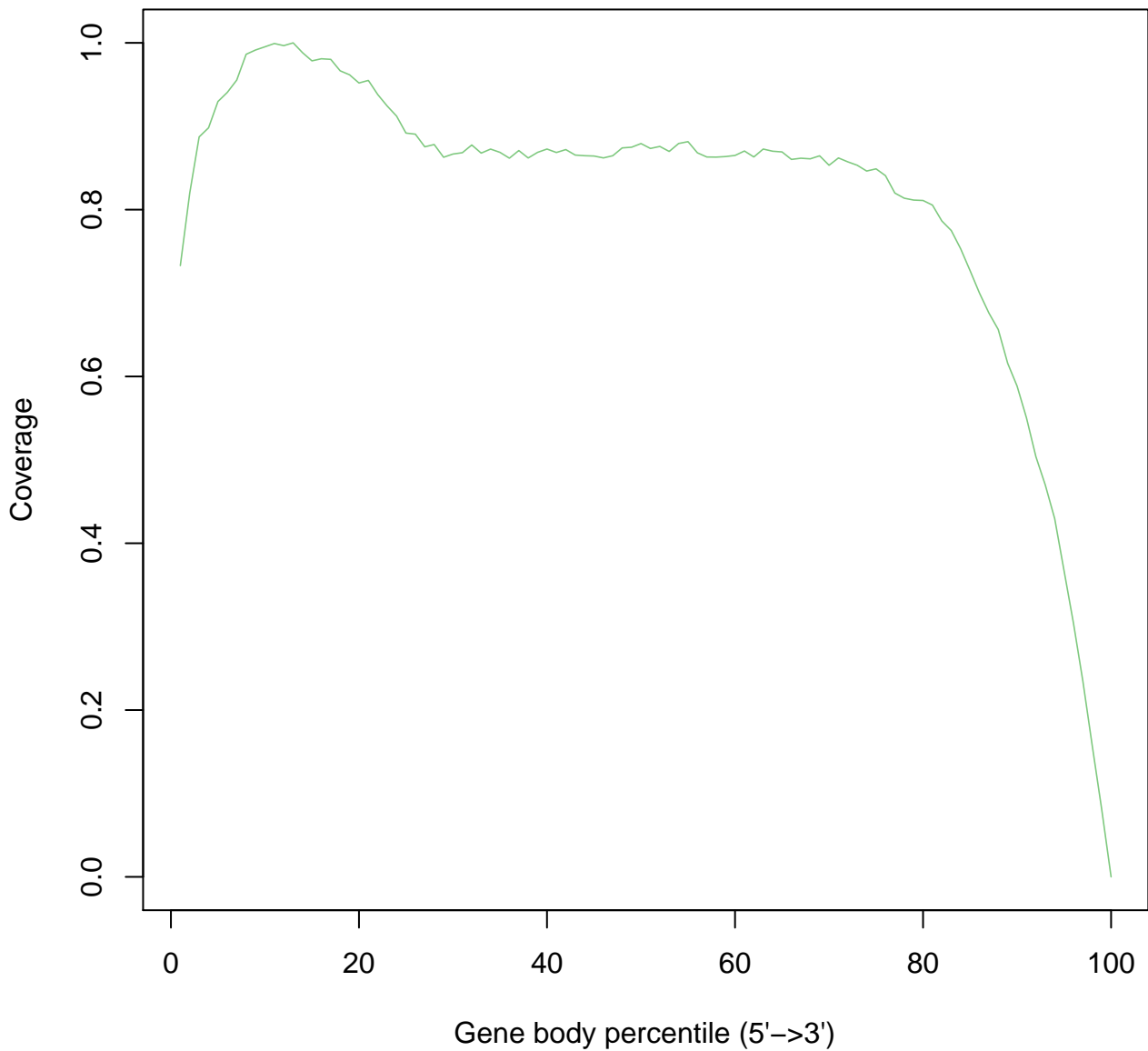

Supplement: Supplementary file 1 [file Data_Sheet_1.ZIP › 04_MapQC/genebody_coverage/D3.geneBodyCoverage.curves.pdf]

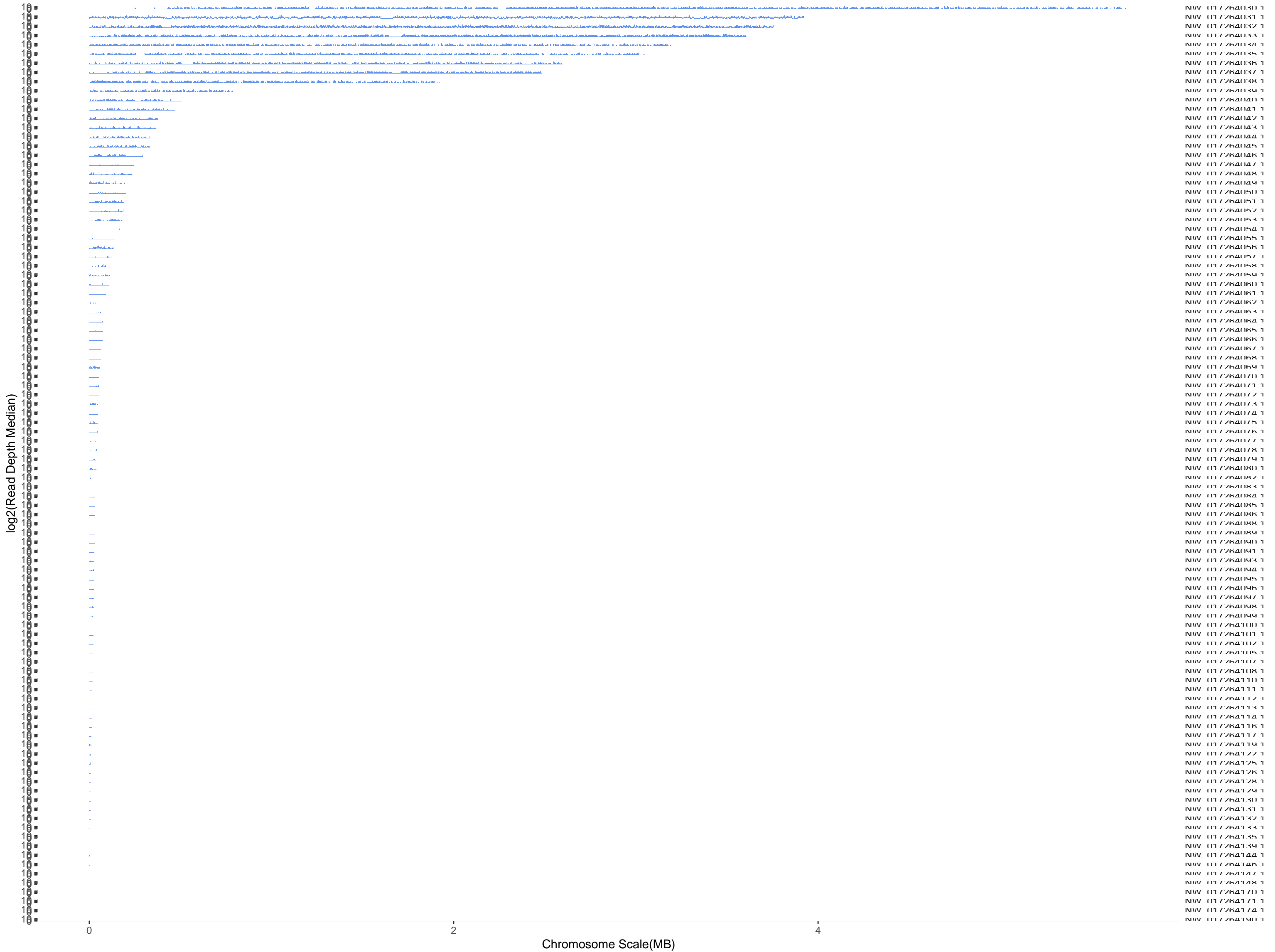

Supplement: Supplementary file 1 [file Data_Sheet_1.ZIP › 04_MapQC/mappedReadsDepth/A1.mapped_reads_depth.pdf]

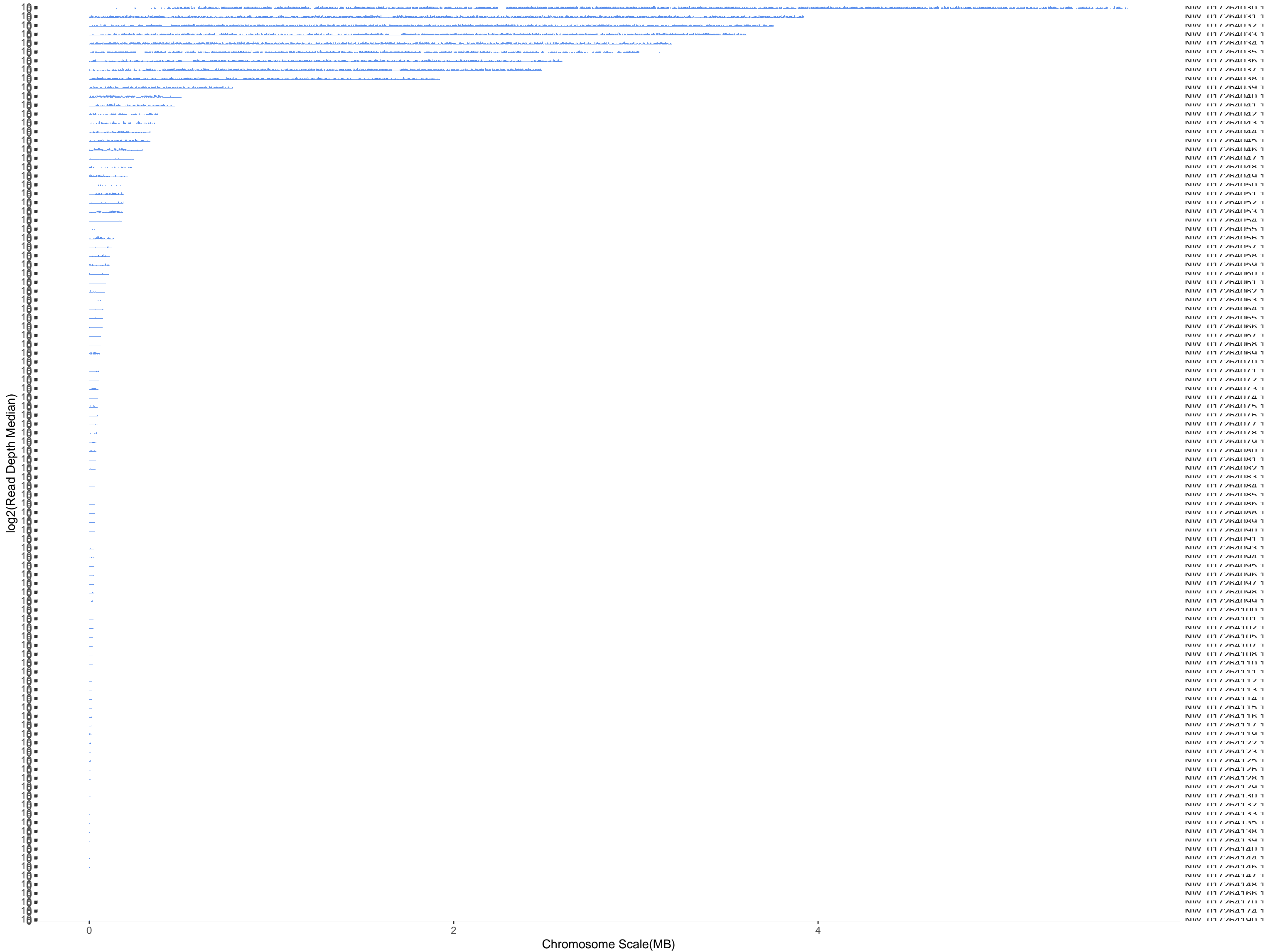

Supplement: Supplementary file 1 [file Data_Sheet_1.ZIP › 04_MapQC/mappedReadsDepth/A2.mapped_reads_depth.pdf]

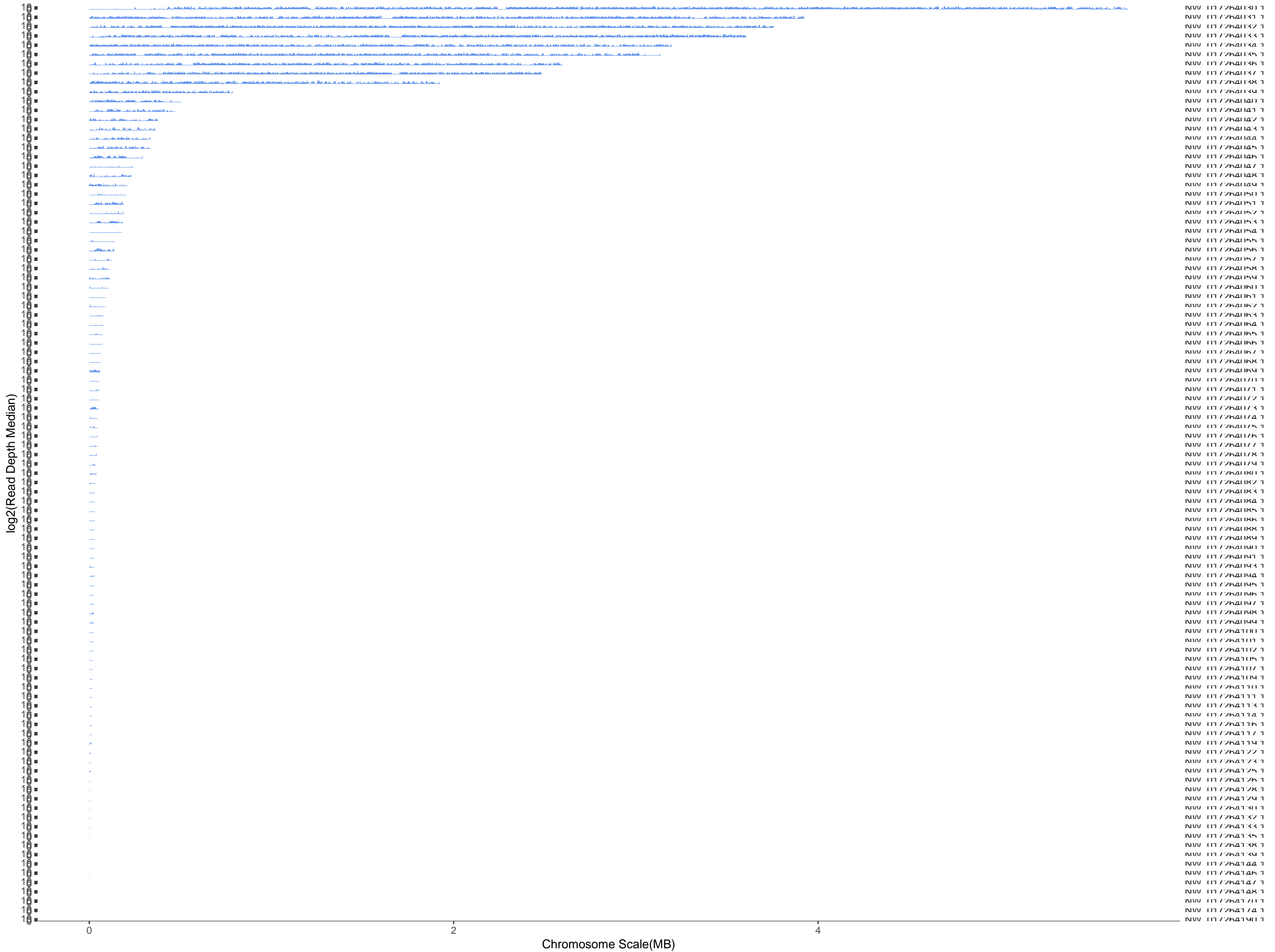

Supplement: Supplementary file 1 [file Data_Sheet_1.ZIP › 04_MapQC/mappedReadsDepth/A3.mapped_reads_depth.pdf]

0

2

4

Chromosome Scale(MB)

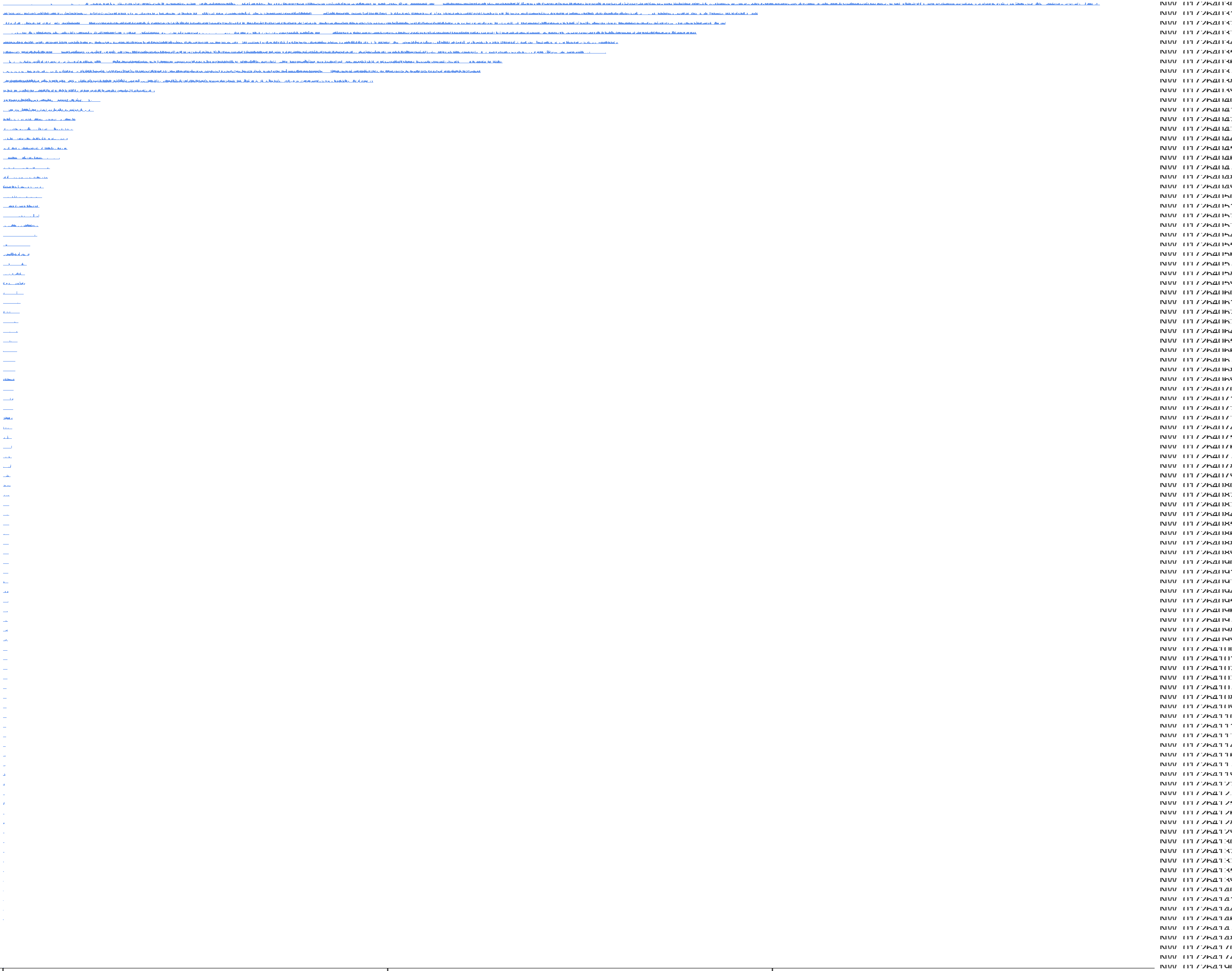

Supplement: Supplementary file 1 [file Data_Sheet_1.ZIP › 04_MapQC/mappedReadsDepth/B1.mapped_reads_depth.pdf]

0

2

4

Chromosome Scale(MB)

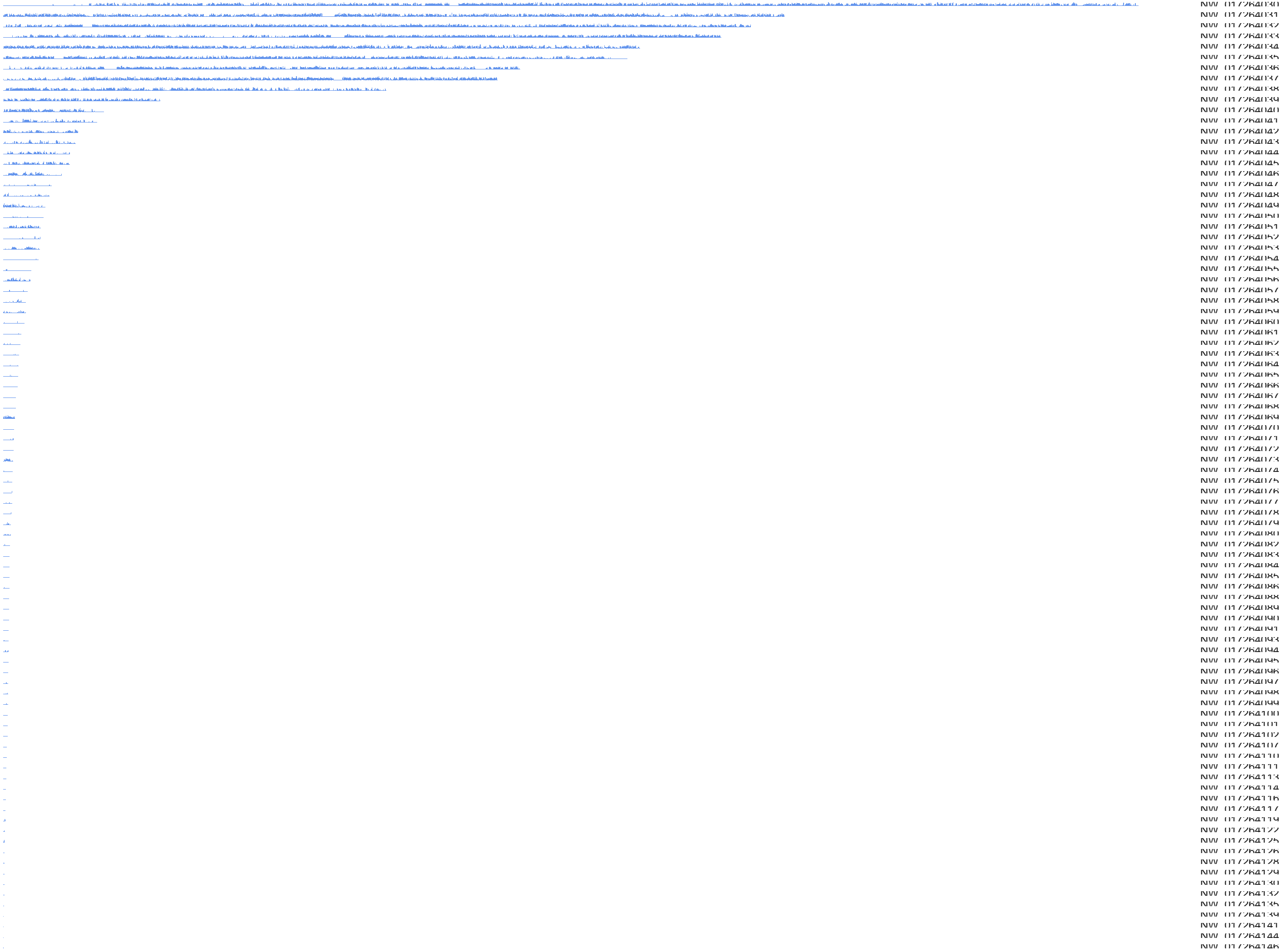

Supplement: Supplementary file 1 [file Data_Sheet_1.ZIP › 04_MapQC/mappedReadsDepth/B2.mapped_reads_depth.pdf]

0

2

4

Chromosome Scale(MB)

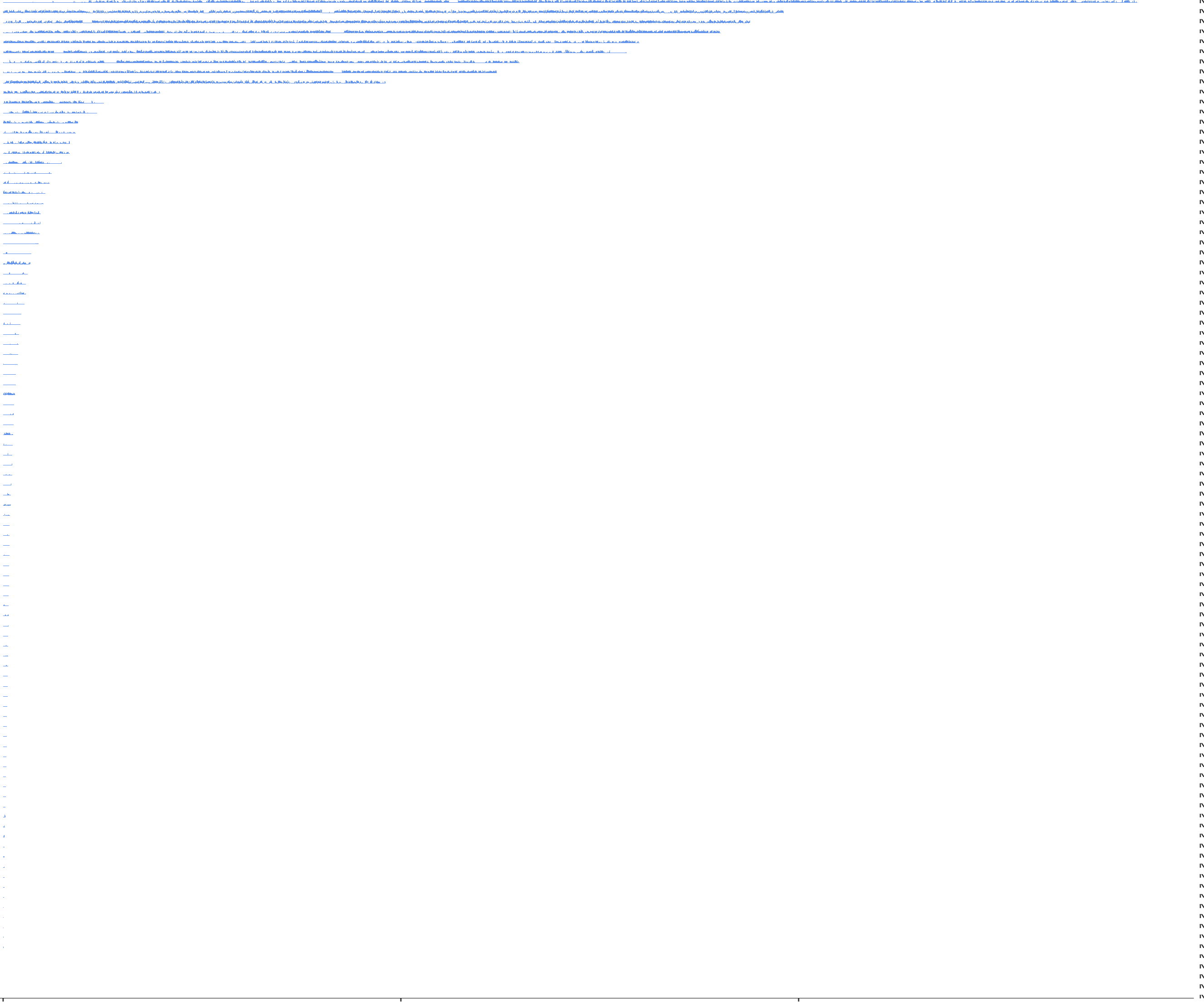

Supplement: Supplementary file 1 [file Data_Sheet_1.ZIP › 04_MapQC/mappedReadsDepth/B3.mapped_reads_depth.pdf]

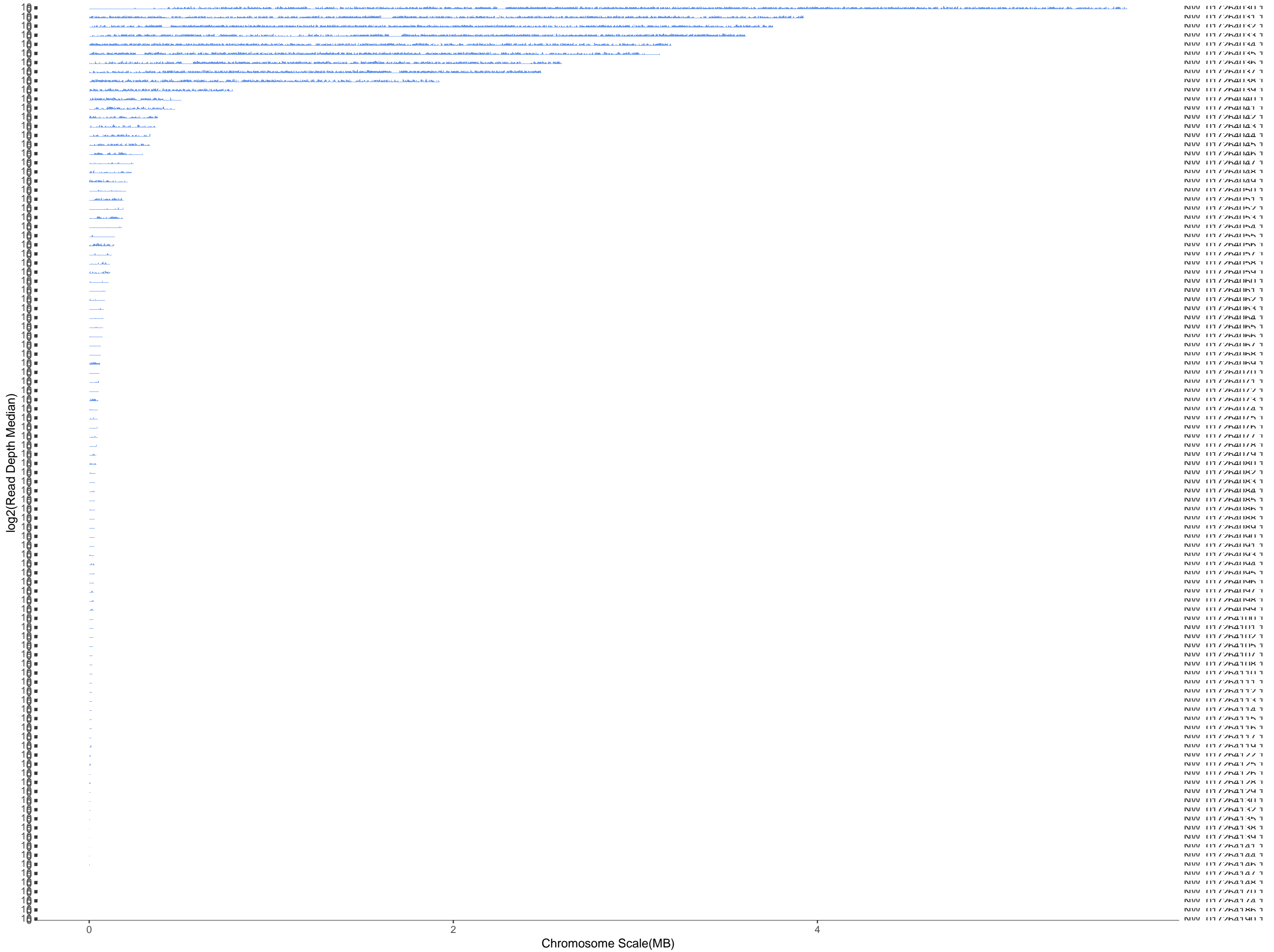

Supplement: Supplementary file 1 [file Data_Sheet_1.ZIP › 04_MapQC/mappedReadsDepth/C1.mapped_reads_depth.pdf]

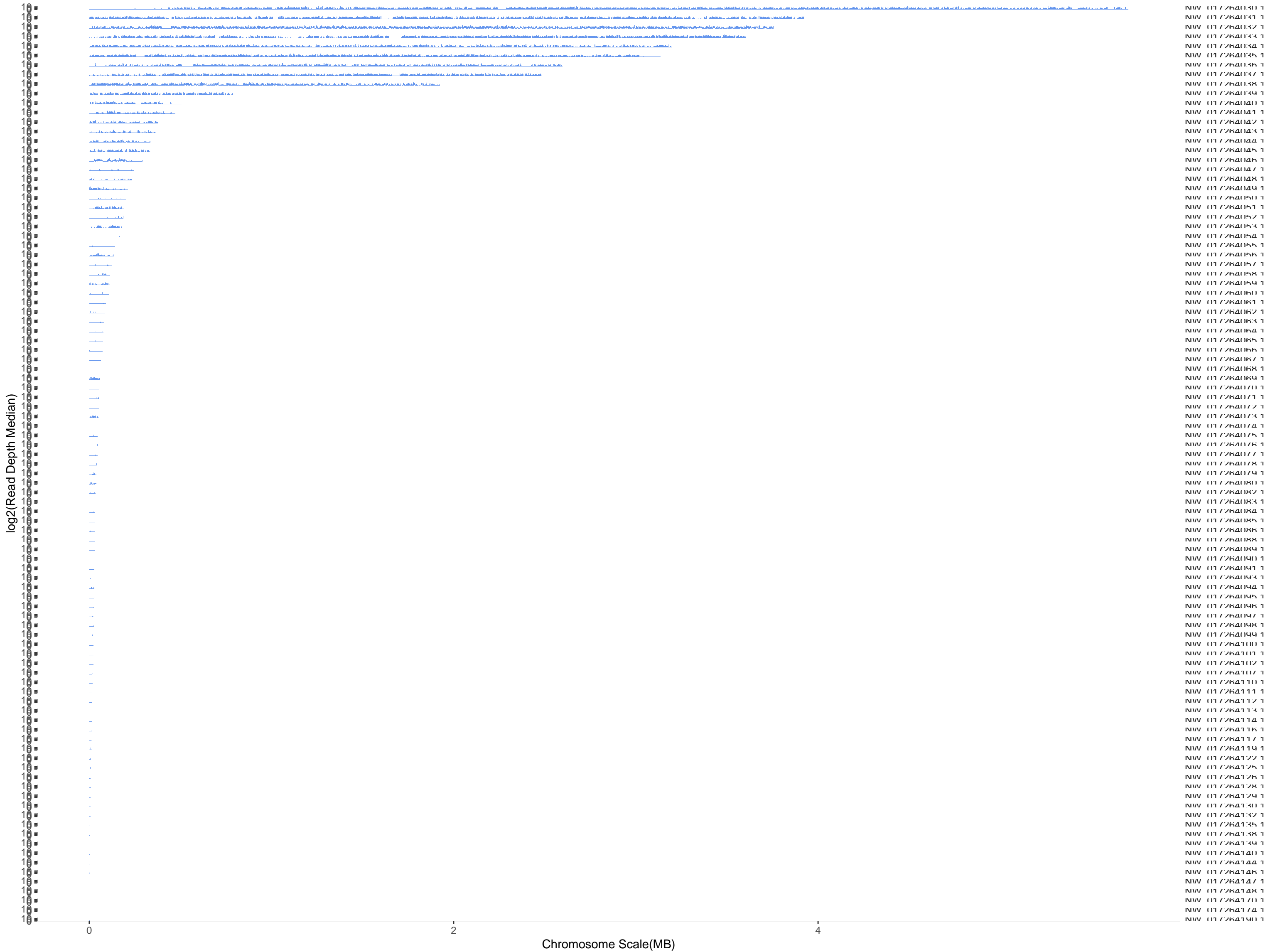

Supplement: Supplementary file 1 [file Data_Sheet_1.ZIP › 04_MapQC/mappedReadsDepth/C2.mapped_reads_depth.pdf]

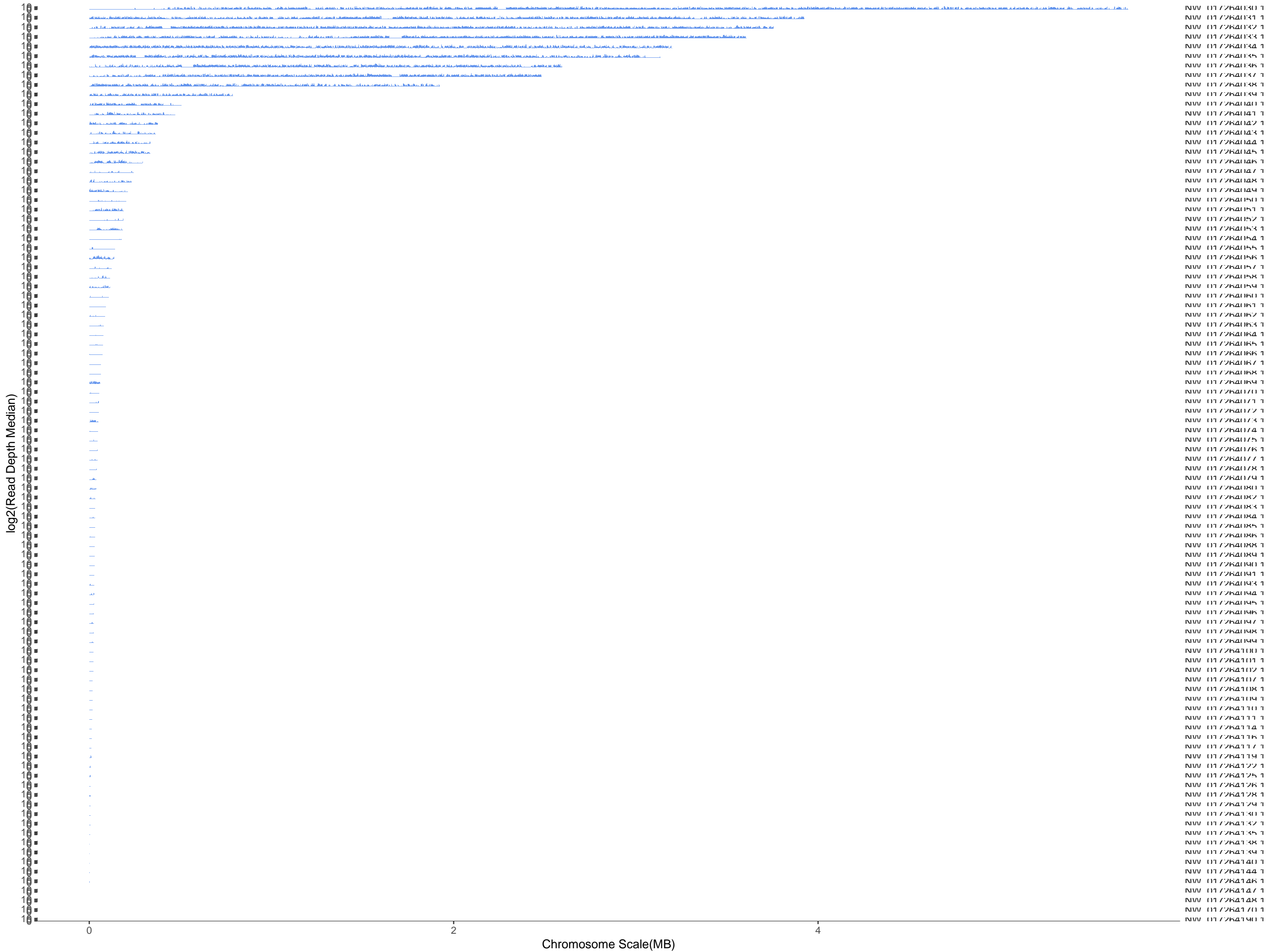

Supplement: Supplementary file 1 [file Data_Sheet_1.ZIP › 04_MapQC/mappedReadsDepth/C3.mapped_reads_depth.pdf]

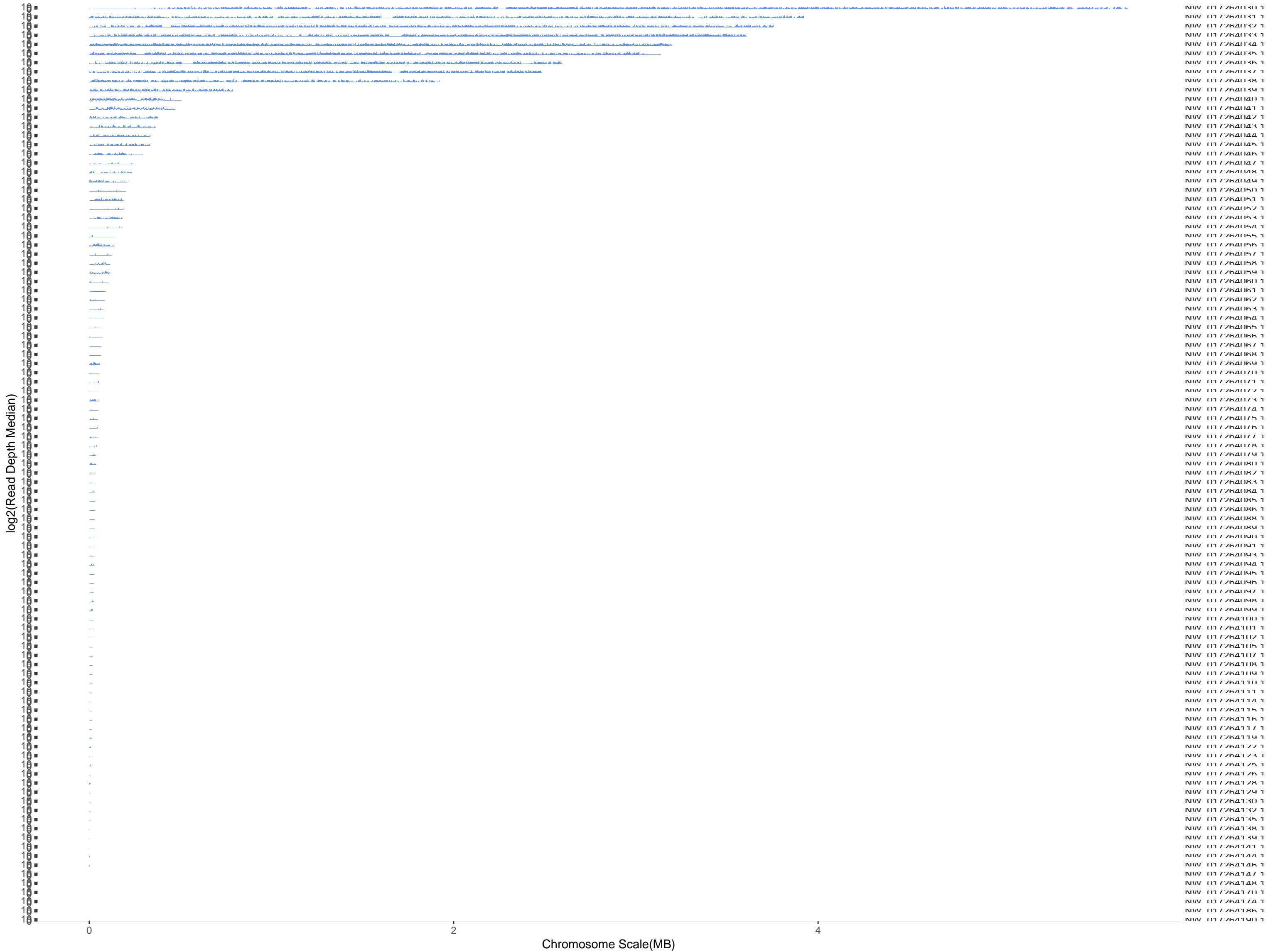

Supplement: Supplementary file 1 [file Data_Sheet_1.ZIP › 04_MapQC/mappedReadsDepth/D1.mapped_reads_depth.pdf]

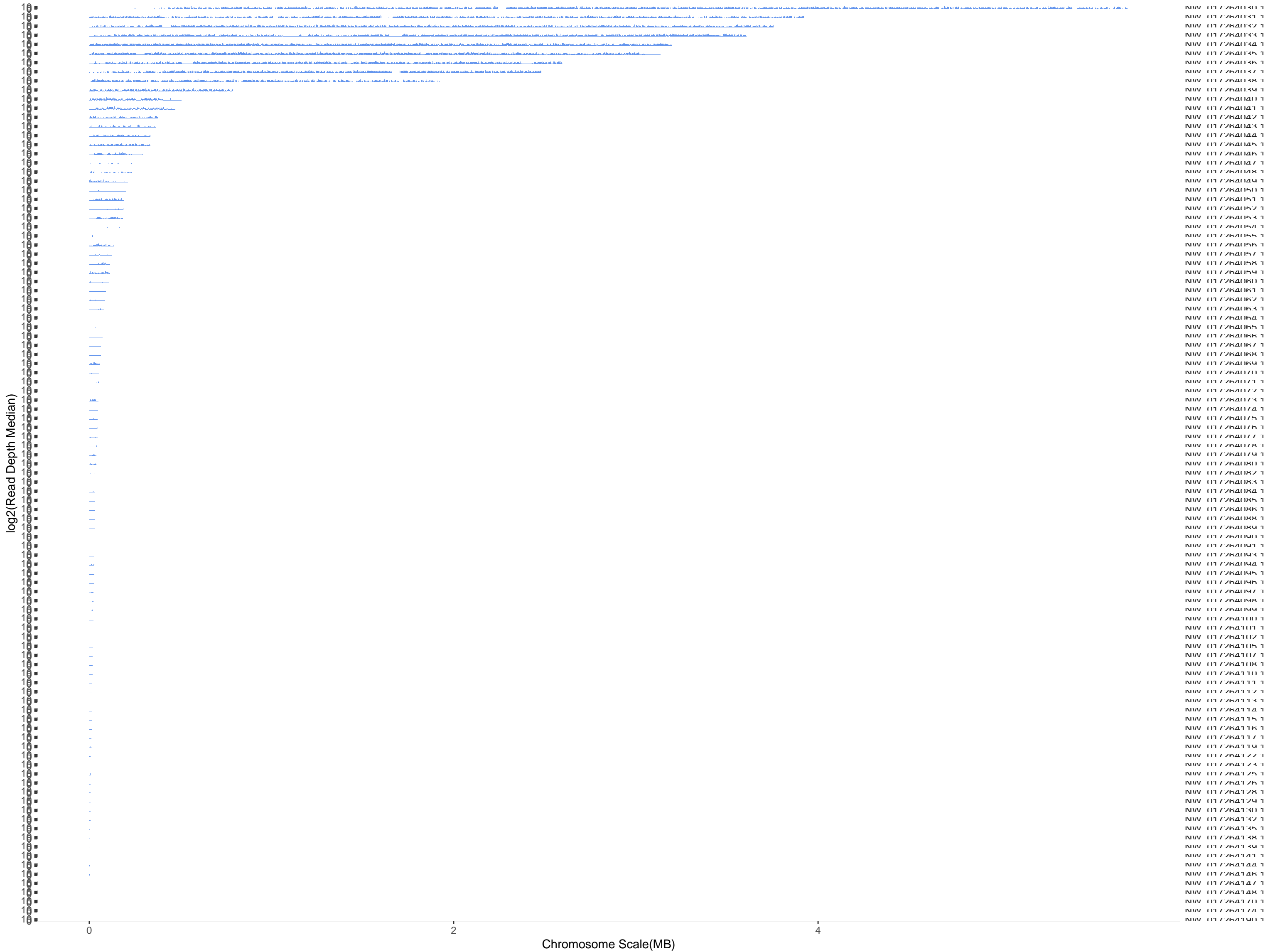

Supplement: Supplementary file 1 [file Data_Sheet_1.ZIP › 04_MapQC/mappedReadsDepth/D2.mapped_reads_depth.pdf]

0

2

4

Chromosome Scale(MB)

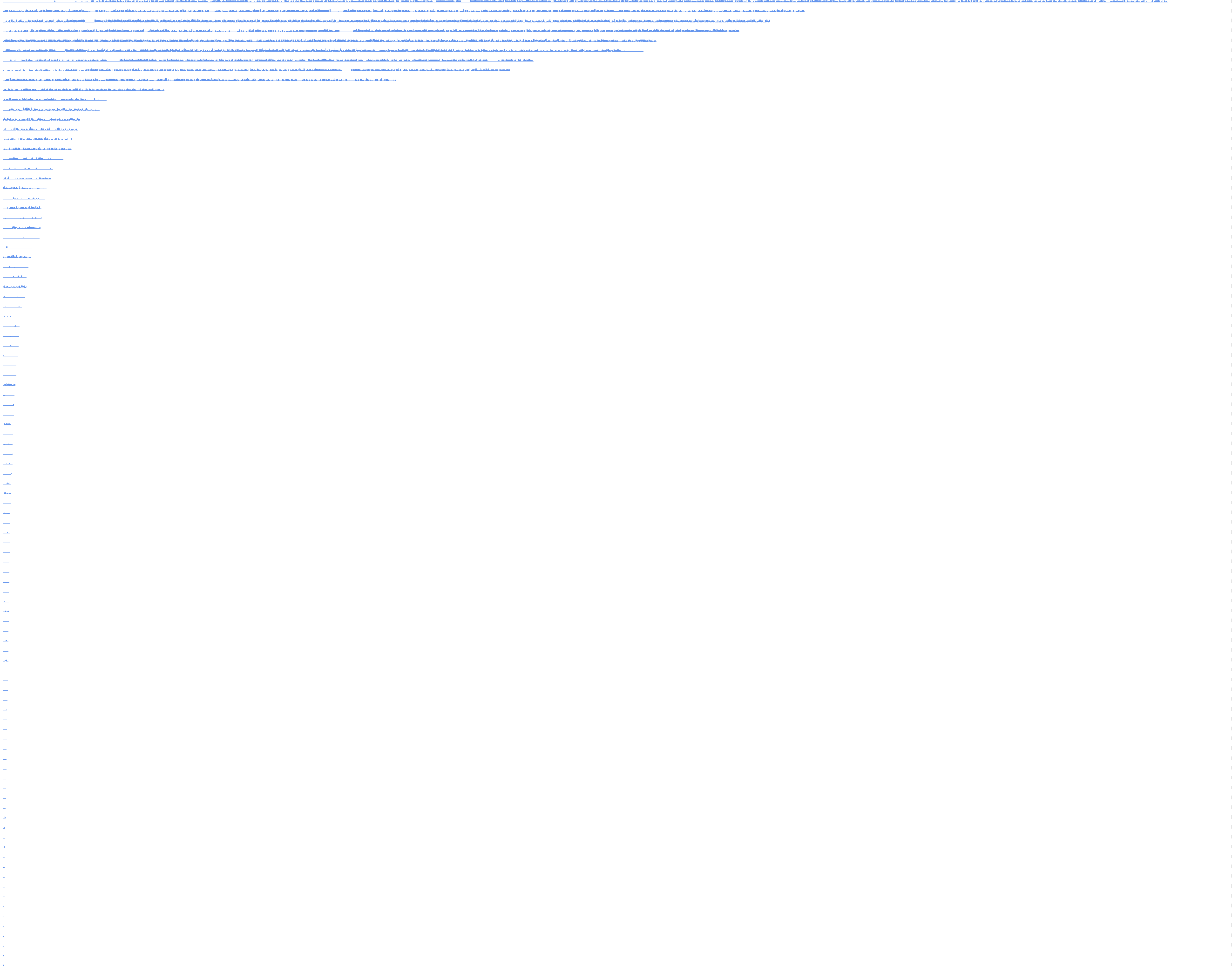

Supplement: Supplementary file 1 [file Data_Sheet_1.ZIP › 04_MapQC/mappedReadsDepth/D3.mapped_reads_depth.pdf]

# Read Distribution

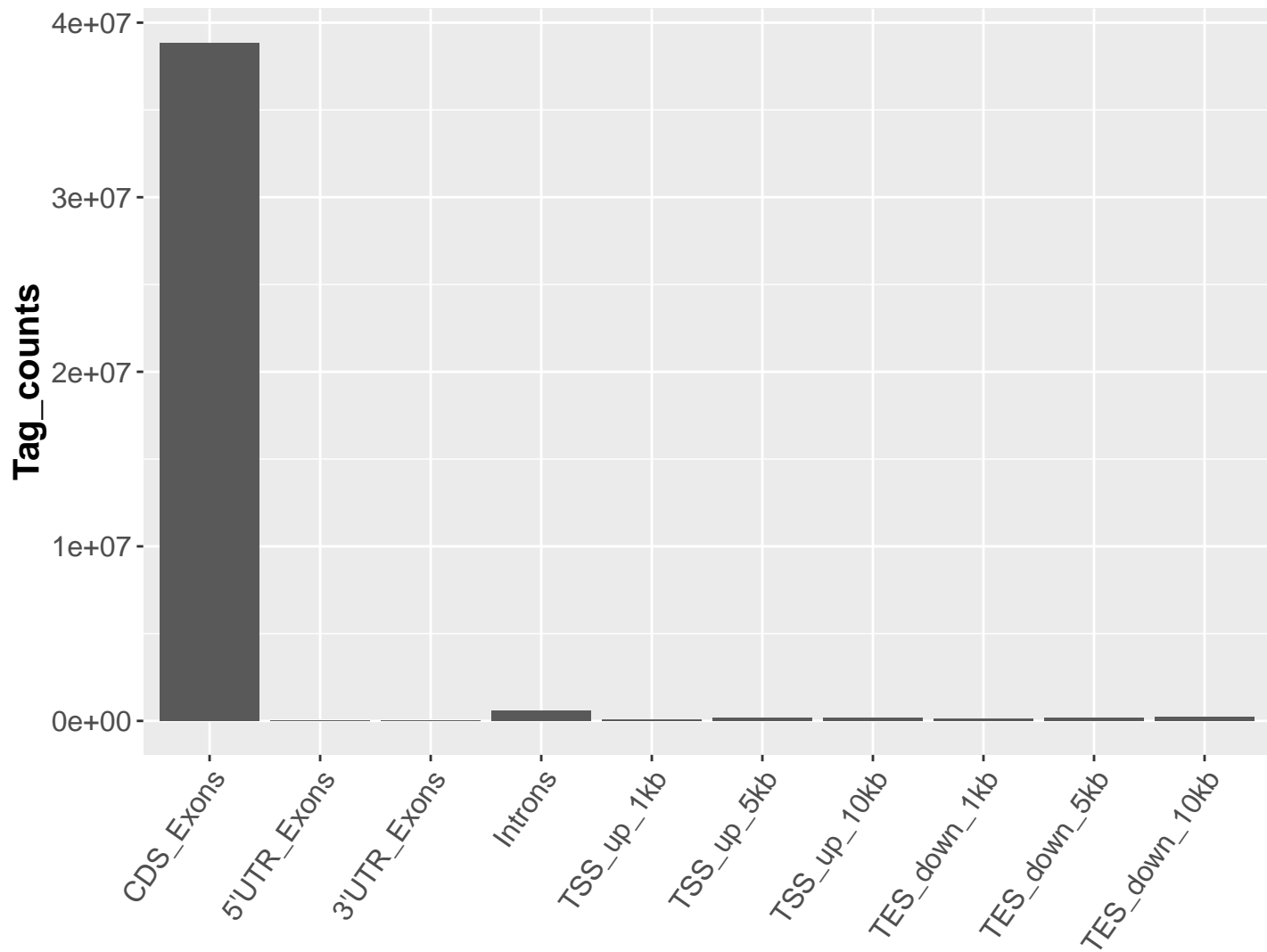

Supplement: Supplementary file 1 [file Data_Sheet_1.ZIP › 04_MapQC/read_distribution/A1.read_distribution.pdf]

# Read Distribution

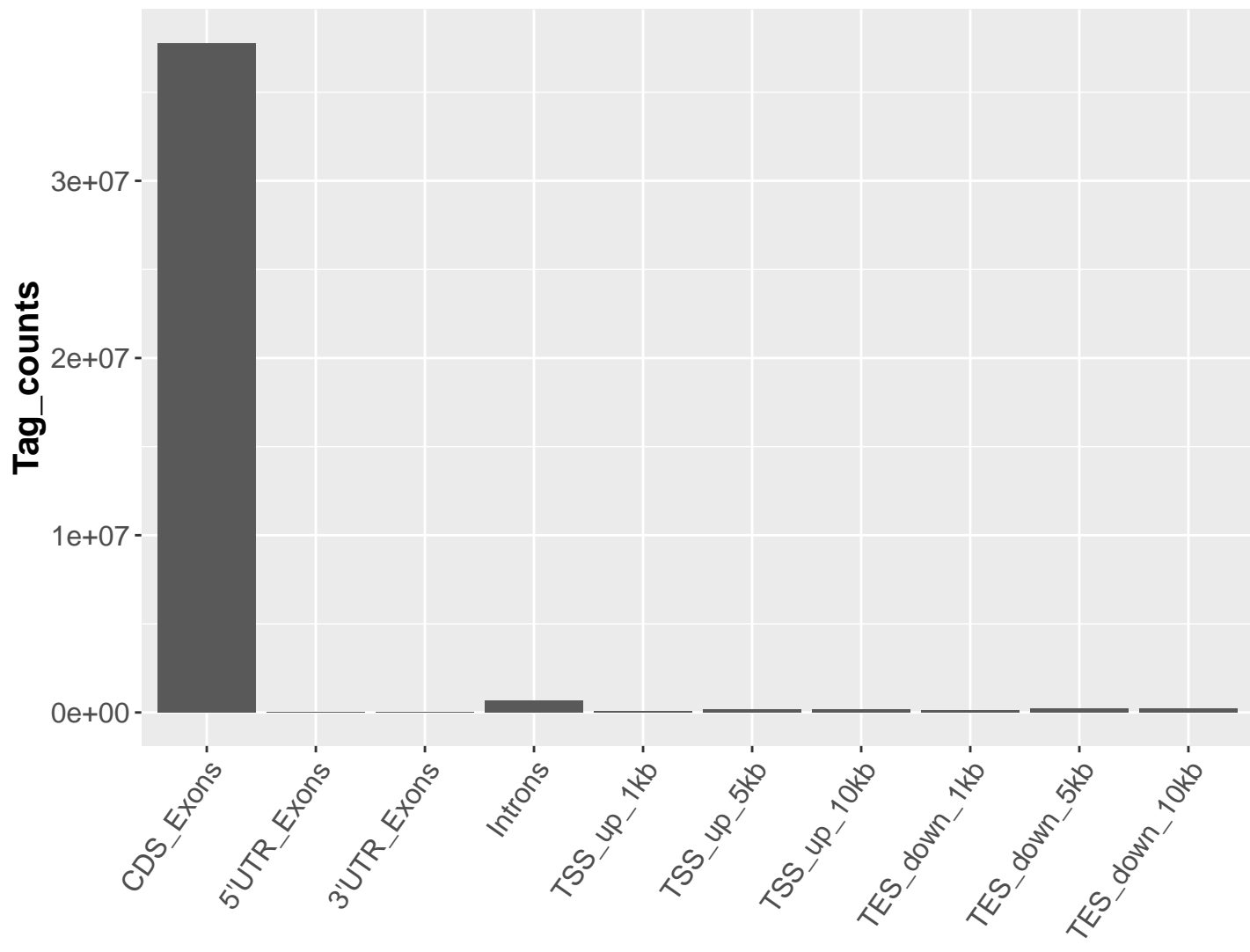

Supplement: Supplementary file 1 [file Data_Sheet_1.ZIP › 04_MapQC/read_distribution/A2.read_distribution.pdf]

# Read Distribution

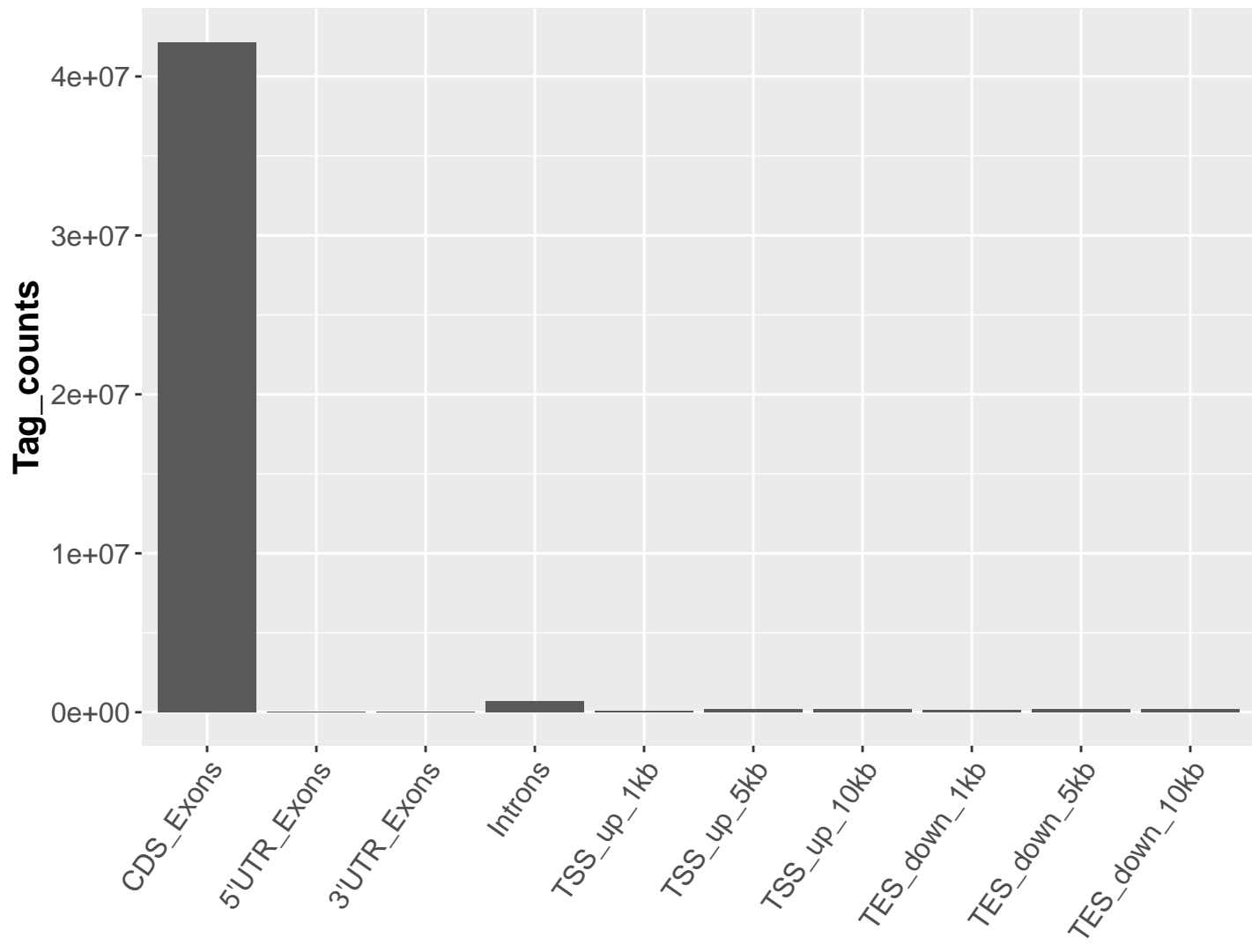

Supplement: Supplementary file 1 [file Data_Sheet_1.ZIP › 04_MapQC/read_distribution/A3.read_distribution.pdf]

# Read Distribution

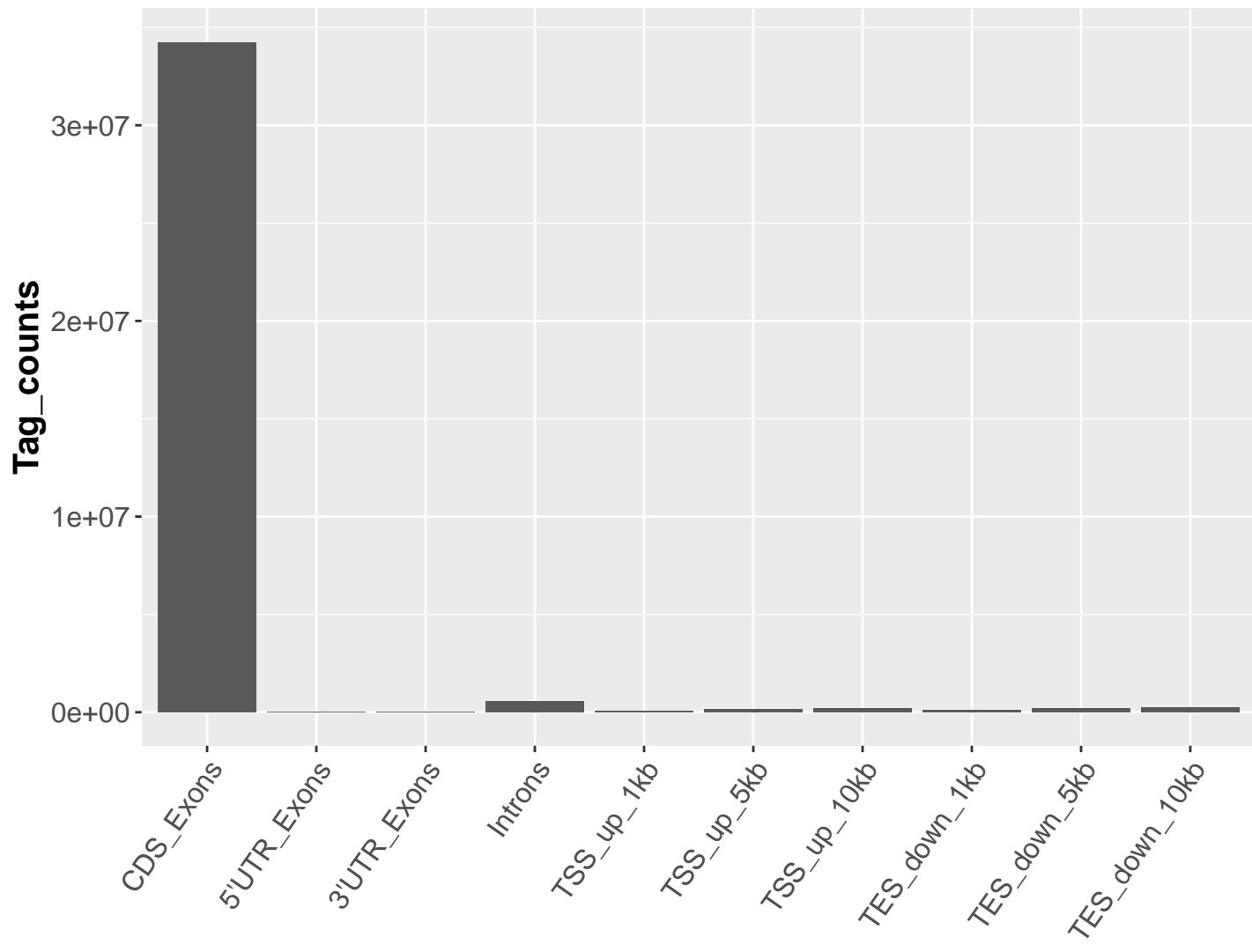

Supplement: Supplementary file 1 [file Data_Sheet_1.ZIP › 04_MapQC/read_distribution/B1.read_distribution.pdf]
