# Supplementary material for: Functional characterization of CreA, ZnT, and MTase as key regulators of cadmium resistance in Paecilomyces lilacinus
Source: Front Microbiol. 2026 May 14;17:1792636. doi: 10.3389/fmicb.2026.1792636 (PMC13218079; doi:10.3389/fmicb.2026.1792636)
Supplement: Supplementary file 2 [file Table_1.DOCX]

| Gene | Name | correlation coefficient  (RT-PCR and RNA-seq | Primer Sequence (5',-3') | Length (bp) |
| --- | --- | --- | --- | --- |
| internal reference gene | β-tubulin | / | F-TGGCAGACCATCTCTGGCGAGCACG  R-GCCTCGTTGAAGTAGACGCTCAT | 126 |
| VFPFJ_04632 | Membrane zinc transporter(ZnT) | 0.891 | F-CCAGCTCACGGCCATCTTTA  R-CCAGACCGAGACCCTCAAAC | 149 |
| VFPFJ_08427 | Ferric reductase transmembrane component 4(Fet4p) | 0.771 | F-CGCGAACCTTCCGCTAGTAT  R-CGATAGTCGCAATCCACCGA | 113 |
| VFPFJ_01627 | Cinnamoyl-CoA reductase(CCR) | 0.672 | F-GTCAACTGAGCTCCTCGCTT  R-GCTGGATTGCTCCTCTCCTG | 131 |
| VFPFJ_05026 | 6-phosphogluconate dehydro genase nad-binding protein(G6PD) | 0.814 | F-GCGTCGTAGTCATCTGCCTT  R-CTTGGGTGTACCGGTTGTGA | 113 |
| VFPFJ_00181 | Superoxide dismutase(SOD) | 0.791 | F-GTCGCTGTCCTCCGTGGTGA  R-GCTTCGCGTTGGCGTCGTT | 118 |
| VFPFJ_07659 | Mitochondrial peroxiredoxin PRX1 | 0.769 | F-CACTCCTGTCTGCACCACCG  R-ACCTGCCCACCAGTCACCTC | 153 |
| VFPFJ_03043 | Epimerase/hydratase | 0.668 | F-AAGAGTACAAGGATGCGGGC  R-CTTGATGAGCTCGGCGTACA | 125 |
| VFPFJ_11308 | Methyltransferase(MTase) | 0.886 | F-ATGCCGAGGAACTCGTGAAG  R-CGGTTCGGGTAGATCAGCTC | 148 |
| VFPFJ_10637 | Aldehyde reductase(AKRlAl) | 0.783 | F-GGTCATCCCTCCAGTTGTCG  R-CGCATCGTTCCAAGTCTCCT | 163 |
| VFPFJ_00707 | Cycloheximide resistance protein(CHX) | 0.685 | F-GAAGCGCACGTACATTGGTC  R-CAGGACGTAGATGGACAGGC | 151 |
| VFPFJ_02045 | Glutathione-dependentformaldehyde activating enzyme protein | 0.787 | F-CGCTCCTGTGCTCGGTGGTA R-CGCAGATGTGGTTGTGGCTGAC | 213 |
| VFPFJ_04681 | Gibberellin 20-oxidase(GA20oX) | 0.689 | F-TCACGCTTCTGTTCCAGGAC  R-ACCATTTCCGTGGGACCATC | 112 |
| VFPFJ_05623 | Cysteine synthase B(CSaseB) | 0.709 | F-GCGAGCCTTTCTGAGCAATA  R-CCGACTCCAAAGCCATCC | 155 |
| VFPFJ_10442 | Major facilitator superfamily transporter(MFS） | 0.687 | F-AGTCATCAGCCGCTACATCG  R-CCAGCCATCGTTGGTGTACT | 118 |
| VFPFJ_01055 | DNA-binding protein CreA(CreA) | 0.884 | F-ATGGCTAGCGCCGCCGTCACCGTCC  R-GATGCTCAAGGCGGTGGAA | 128 |
| VFPFJ_07405 | Glucose-methanol-choline  oxidoreductase(GMC） | 0.765 | F -AAGCCCAACGTCACCATCAT  R -GCGAGTCAACCTGGACTTCA | 244 |
| VFPFJ_04703 | Basic-leucine zipper transcription factor(bZIP） | 0.794 | F-ATCCCAGCCAACACGCTAAA  R-AAGAGACACTGGAGGCGTTG | 161 |
| VFPFJ_04710 | Nucleoside-diphosphate-sugar epimerase protein(NDse) | 0.801 | F-CGATACACGCGCAGTCTACA  R-GACGAAGGAGGACAGCGATT | 142 |
| VFPFJ_09620 | NAD(P)-binding protein | 0.764 | F-TTCCAACGACCGTCTCAAGG  R-GCAGCTCCTCAATGAAGGGT | 173 |
| VFPFJ_10184 | 2OG-Fe(II)oxygenase | 0.698 | F-AGTCATCAGCCGCTACATCG  R-CCAGCCATCGTTGGTGTACT | 118 |

Supplementary Table 1
