# Supplementary material for: Functional characterization of CreA, ZnT, and MTase as key regulators of cadmium resistance in Paecilomyces lilacinus
Source: Front Microbiol. 2026 May 14;17:1792636. doi: 10.3389/fmicb.2026.1792636 (PMC13218079; doi:10.3389/fmicb.2026.1792636)
Supplement: Supplementary file 1 [file Data_Sheet_1.ZIP › mRNA/3_Enrichment/D_vs_A/topGO_MF_top10.pdf]

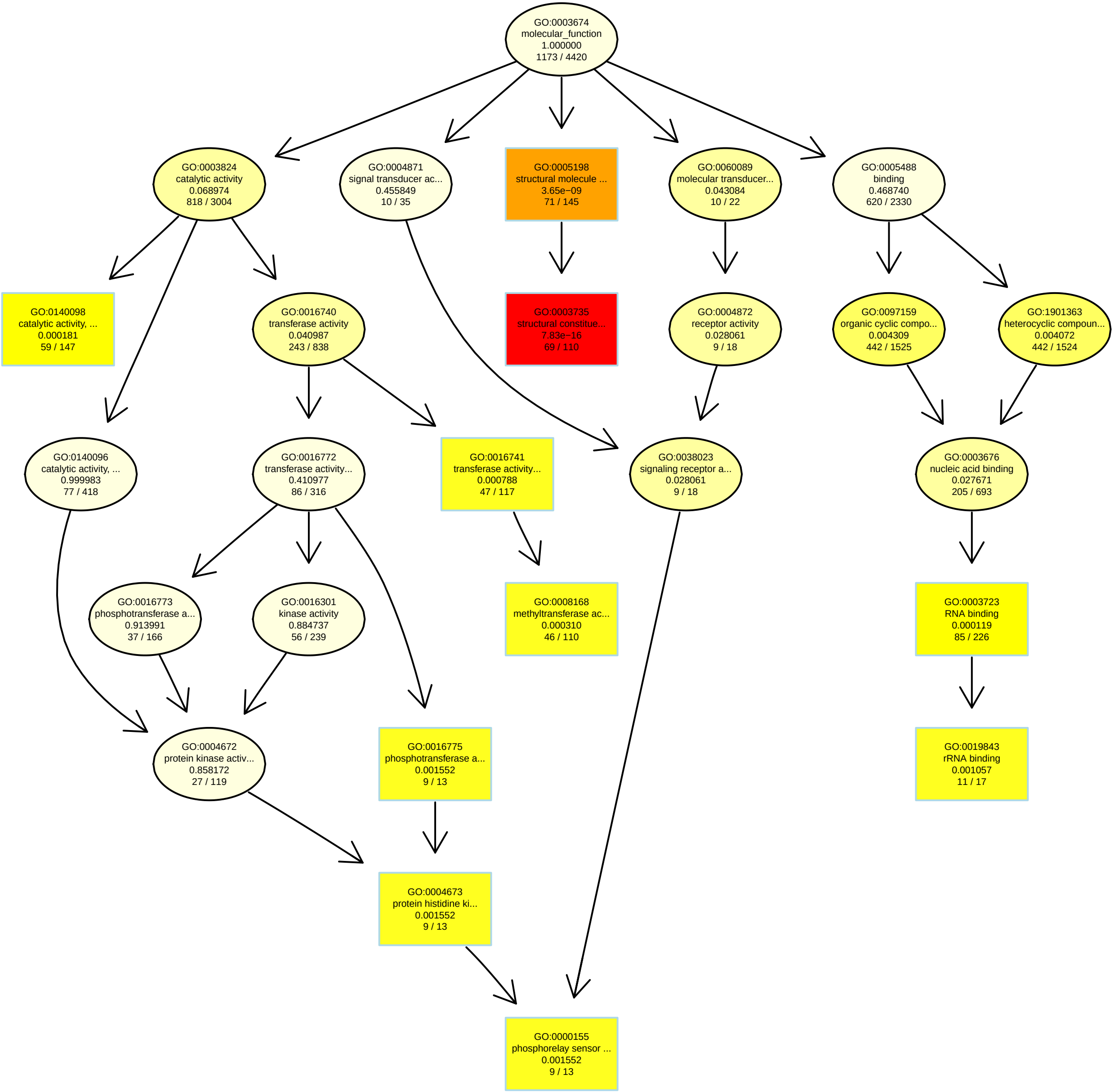

GO:0003674
molecular_function
1.000000
1173 / 4420
GO:0003824
catalytic activity
0.068974
818 / 3004
GO:0004871
signal transducer ac...
0.455849
10 / 35
GO:0005198
structural molecule ...
3.65e−09
71 / 145
GO:0060089
molecular transducer...
0.043084
10 / 22
GO:0005488
binding
0.468740
620 / 2330
GO:0140098
catalytic activity, ...
0.000181
59 / 147
GO:0016740
transferase activity
0.040987
243 / 838
GO:0003735
structural constitue...
7.83e−16
69 / 110
GO:0004872
receptor activity
0.028061
9 / 18
GO:0097159
organic cyclic compo...
0.004309
442 / 1525
GO:1901363
heterocyclic compoun...
0.004072
442 / 1524
GO:0140096
catalytic activity, ...
0.999983
77 / 418
GO:0016772
transferase activity...
0.410977
86 / 316
GO:0016741
transferase activity...
0.000788
47 / 117
GO:0038023
signaling receptor a...
0.028061
9 / 18
GO:0003676
nucleic acid binding
0.027671
205 / 693
GO:0016773
phosphotransferase a...
0.913991
37 / 166
GO:0016301
kinase activity
0.884737
56 / 239
GO:0008168
methyltransferase ac...
0.000310
46 / 110
GO:0003723
RNA binding
0.000119
85 / 226
GO:0004672
protein kinase activ...
0.858172
27 / 119
GO:0016775
phosphotransferase a...
0.001552
9 / 13
GO:0019843
rRNA binding
0.001057
11 / 17
GO:0004673
protein histidine ki...
0.001552
9 / 13
GO:0000155
phosphorelay sensor ...
0.001552
9 / 13
