# Supplementary material for: Functional characterization of CreA, ZnT, and MTase as key regulators of cadmium resistance in Paecilomyces lilacinus
Source: Front Microbiol. 2026 May 14;17:1792636. doi: 10.3389/fmicb.2026.1792636 (PMC13218079; doi:10.3389/fmicb.2026.1792636)
Supplement: Supplementary file 1 [file Data_Sheet_1.ZIP › mRNA/4_TFs_family/TF.count.pdf]

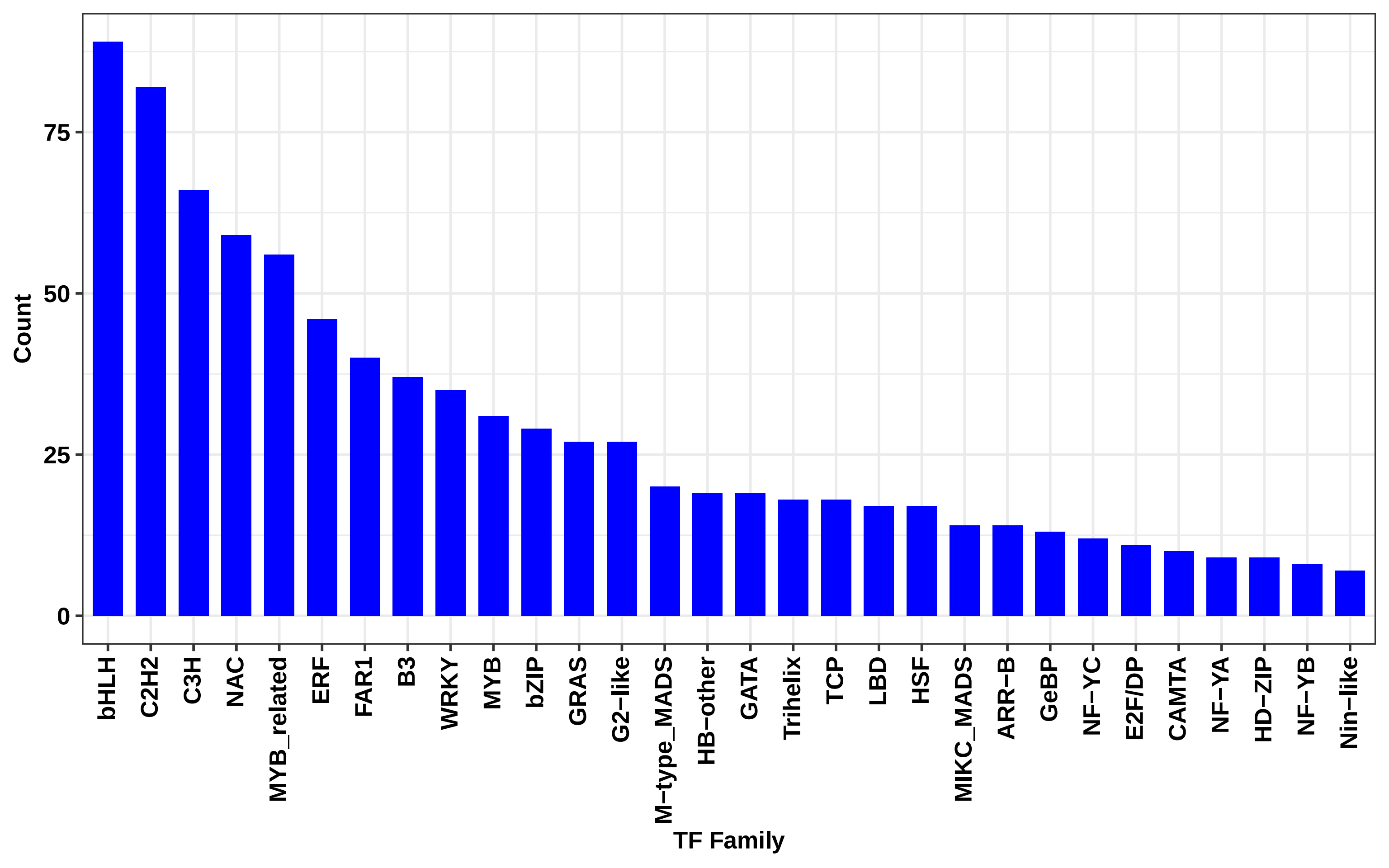
Count
0
25
50
75
bHLH
C2H2
C3H
NAC
MYB_related
ERF
FAR1
B3
WRKY
MYB
bZIP
GRAS
G2−like
M−type_MADS
HB−other
GATA
Trihelix
TCP
LBD
HSF
MIKC_MADS
ARR−B
GeBP
NF−YC
E2F/DP
CAMTA
NF−YA
HD−ZIP
NF−YB
Nin−like
TF Family
