# Supplementary material for: Functional characterization of CreA, ZnT, and MTase as key regulators of cadmium resistance in Paecilomyces lilacinus
Source: Front Microbiol. 2026 May 14;17:1792636. doi: 10.3389/fmicb.2026.1792636 (PMC13218079; doi:10.3389/fmicb.2026.1792636)
Supplement: Supplementary file 1 [file Data_Sheet_1.ZIP › mRNA/5_PPI/D_vs_A.PPI.pdf]

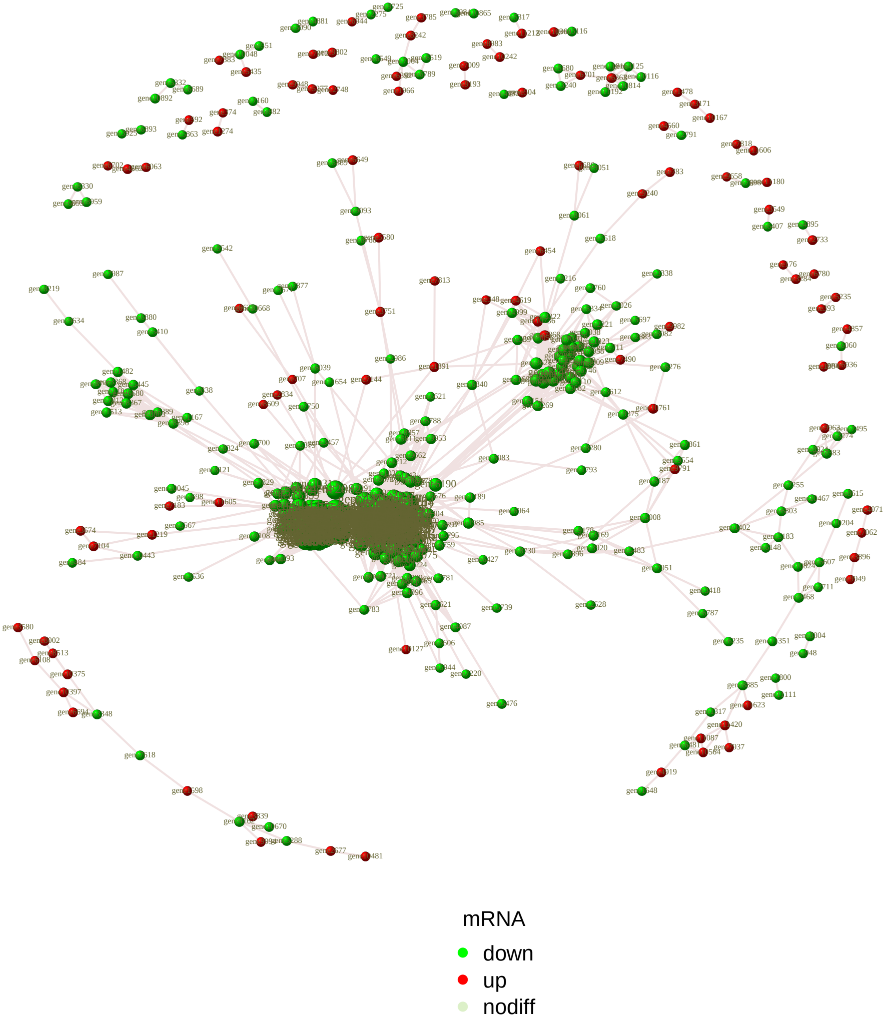
mRNA
down
up
nodiff
